# Asymmetric synthesis of γ-branched amines via rhodium-catalyzed reductive amination

**DOI:** 10.1038/s41467-018-03535-y

**Published:** 2018-03-22

**Authors:** Zhao Wu, Summer D. Laffoon, Kami L. Hull

**Affiliations:** Department of Chemistry, University of Illinois at Urbana-Champaign, 600 South Mathews Avenue, Urbana, IL 61801 USA

## Abstract

Amines bearing γ-stereocenters are highly important structural motifs in many biologically active compounds. However, reported enantioselective syntheses of these molecules are indirect and often require multiple steps. Herein, we report a general asymmetric route for the one-pot synthesis of chiral γ-branched amines through the highly enantioselective isomerization of allylamines, followed by enamine exchange and subsequent chemoselective reduction. This protocol is suitable for establishing various tertiary stereocenters, including those containing dialkyl, diaryl, cyclic, trifluoromethyl, difluoromethyl, and silyl substituents, which allows for a rapid and modular synthesis of many chiral γ-branched amines. To demonstrate the synthetic utility, Terikalant and Tolterodine are synthesized using this method with high levels of enantioselectivity.

## Abstract (web summary)

Biologically active compounds often contain a chiral centre in proximity of amine groups. Here, the authors developed a strategy involving asymmetric isomerization of allylic amines, enamine exchange and chemoselective reduction for the one-pot highly enantioselective synthesis of gamma-branched amines.

## Introduction

**Fig. 1:**
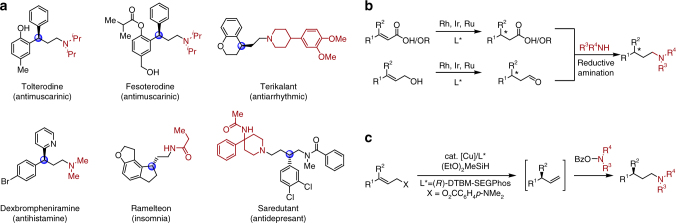
Significance and syntheses of chiral γ-branched amines. **a** Biologically active molecules containing chiral γ-branched amine moiety. **b** Asymmetric hydrogenation or isomerization followed by reductive amination for the multistep synthesis. **c** Direct synthesis via a Cu–H catalyzed relay hydroamination reaction

Aliphatic amines with adjacent stereocenters are prevalent in natural products and pharmaceuticals and are often key contributors to their potent biological activity^1^. In particular, enantiopure γ-branched amines represent an important subclass of bioactive amines, including many pharmaceutical agents (Fig. 1a). Despite the generality of this structure, the direct synthesis of chiral, γ-branched amines remains underdeveloped compared to the well-established methods for constructing α-branched and β-branched amines^2–5^, as well as distal stereocenters to other function groups such as ketones^6^, aldehydes^7–10^, and amides^11^.

Known catalytic approaches to install this subunit often require multistep synthetic sequences via chiral, β-branched carbonyl intermediates, which can hinder the rapid generation of compound libraries for high throughput screening in medicinal chemistry. For example, transition metal-catalyzed asymmetric hydrogenation of α,β-unsaturated acids or esters^12–14^ affords the enantiopure β-branched carbonyl intermediates, followed by a reductive amination to install the desired chiral γ-branched amines (Fig. 1b). However, varying substituents at the newly introduced stereocenters, such as aryl vs. alkyl, acyclic vs. cyclic, or carbon atom vs. heteroatom, often requires different metal/ligand scaffolds to achieve high enantioselectivity^12–14^. The redox neutral isomerization of allylic amines^15,16^ or alcohols^17–19^ provides a solution to this problem; however, current methods suffer from very limited substrate scope^9–13^. To the best of our knowledge, there is only one reported method for the direct synthesis of chiral γ-branched amines (Fig. 1c). Buchwald et al. have shown that 3,3-disubstituted allylic esters can undergo an enantioselective hydrocupration followed by β-alkoxide elimination and subsequent anti-Markovnikov hydroamination of the intermediate terminal olefin to afford γ-branched amines in one step^20^. Although this method demonstrates high enantioselectivity under a ligand-controlled hydrocupration of allylic esters, the preparation of electrophilic amines requires additional synthetic operations and limits the substrate scope to secondary alkyl amines^20^.

**Fig. 2:**
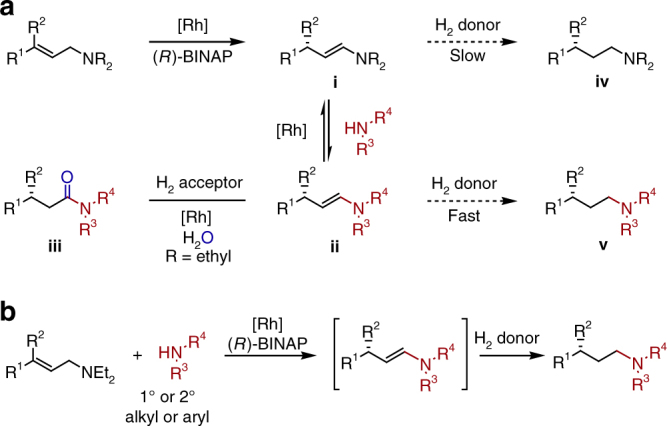
Design of a tandem asymmetric isomerization—enamine exchange—reduction process. **a** The chemoselectivity is determined by the relative reduction rate of intermediates i and ii. When R = ethyl, exclusive formation of β-branched amide iii is observed in the presence of hydrogen acceptor. **b** One-pot synthesis of chiral γ-branched amine from allylic amine, exogenous amine nucleophile, and hydrogen donor

Our group has recently published a one-step synthesis of chiral, β-branched amides via Rh-catalyzed enantioselective isomerization of allylic amines, followed by enamine exchange, and subsequent oxidation^21^. The slow oxidation of the more sterically hindered diethyl enamine (i, when R = ethyl, Fig. 2a) compared to facile oxidation of enamine (ii) leads to exclusive formation of the desired amide product (iii). Based on this report, we proposed that chiral enamine intermediate (ii) could instead be reduced to afford the valuable enantiopure γ-branched amine (v).

We report herein a nucleophilic amination of allylic amines with exogenous amine nucleophiles to afford chiral, γ-branched amines via a transfer hydrogenation (Fig. 2b). Both primary and secondary alkyl/aryl amines are effective nucleophiles, coupling with allylic diethylamine precursors to afford various γ-branched amine products with excellent enantioselectivities in a two-step one-pot manner.

## Results

### Reaction discovery and optimization

To establish a method for the selective conversion of allylic amines to enantiopure γ-branched amines, we began our investigation by examining a variety of hydrogen donors in the reductive amination of geranyl diethylamine (**1a**) with morpholine (**2b**) under slightly modified conditions from our previous report^21^. Compared to the oxidative process, the reduction is more challenging as the hydrogenated starting material (Fig. 2a, iv) was often observed as the major byproduct in the amidation reaction^21^. Therefore, an appropriate selection of a hydrogen donor and starting material (R group) to allow for the rapid and chemoselective reduction of intermediate (ii) was the key challenge in our investigation. No conversion of **1a** was observed in the presence of H_2_ donors, presumably due to protonation of the basic allylic nitrogen atom or coordination to the cationic catalyst, thereby impeding the initiation of the 1,3-hydride shift^15,16^. Sequential addition of the hydrogen donor after the isomerization/enamine exchange step led to higher conversion of starting material, with HCO_2_H showing superior reactivity and selectivity (Table 1, entries 1–4). Increasing the equivalency of amine nucleophile improved the ratio of **3a**/**3a**’, but did not increase the yield of the desired product **3a** (Table 1, entries 4–6). Different allylic amine precursors (**1b**–**d**) were then tested to compare both chemoselectivity and enantioselectivity (Table 1, entries 7–9). Elevated temperature was required to achieve high conversion for these substrates. Less sterically hindered dimethylamino substrate **1b** afforded poor chemoselectivity and high enantioselectivity; however, bulkier allylic diisopropylamine **1c** showed greater chemoselectivity but poor enantioselectivity. Secondary amine precursor **1d** was less reactive and selective under these conditions.

**Table 1:** Selected optimization of reductive amination of allylic amines^a^


Entry	1	R, R’	T (°C)	X	Hydrogen donor	Yield **3a** (%)^b^	Yield **3a’** (%)^b^
1	**1a**	Et, Et	40	1.2	^*i*^PrOH	<1 ^c^	5
2	**1a**	Et, Et	40	1.2	MeOH	<1 ^c^	2
3	**1a**	Et, Et	40	1.2	HCO_2_NH_4_	12	20
4	**1a**	Et, Et	40	1.2	HCOOH	88 (96.2:3.8 er)	10
5	**1a**	Et, Et	60	2.0	HCOOH	87	8
6	**1a**	Et, Et	60	3.0	HCOOH	88	5
7	**1b**	Me, Me	80	1.2	HCOOH	53 (96.4:3.6 er)	43
8	**1c**	*i*-Pr, *i*-Pr	80	1.2	HCOOH	80 (77.6:22.4 er)	<1
9	**1d**	Cy, H	80	1.2	HCOOH	44	28

er: enantiometric ratio

^a^General reaction conditions: geranyl amine (**1**) (0.12 mmol, 1.0 equiv, *E*/*Z* = 97.5:2.5), morpholine (**2a**), hydrogen donor (3.0 equiv), THF (1.2 M). The absolute configuration of **3a** was assigned by analogy

^b^In situ yield determined by GC or NMR analysis

^c^Enamine of **3a** was observed as the major product

### Substrate scope

**Fig. 3:**
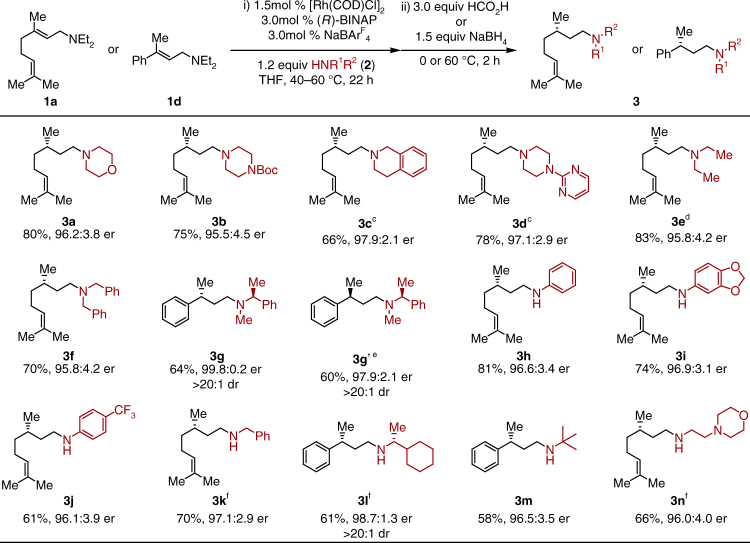
Scope of amine nucleophiles for the reductive amination of allylamine. a: General reaction conditions: **1a** (0.24 mmol, 1.0 equiv, *E*/*Z* = 97.5:2.5, 40 °C for 1st step) or **1b** (0.24 mmol, 1.0 equiv, *E*/*Z* > 99:1, 60 °C for 1st step) nucleophile **2** (1.2 equiv), hydrogen donor (3.0 equiv), THF (1.2 M). b: For **3a**–**3g**, HCO_2_H used as H_2_ donor at 60 °C for 2nd step; For **3h**–**3n**, NaBH_4_ (1.5 equiv) used as reductant at 0 °C to rt for 2nd step. c: **2c** and **2d** added together with HCO_2_H. d: No nucleophile added. e: (*S*)-BINAP used. f: **2k**, **2l**, and **2n** added after isomerization. See supplementary methods for details. dr diastereomeric ratio

With these optimized conditions in hand, the amine nucleophile scope was investigated (Fig. 3). Secondary cyclic amines such as morpholine (**3a**), Boc-protected piperazine (**3b**), tetrahydroisoquinoline (**3c**), and 2-(piperazin-1-yl) pyrimidine (**3d**) all gave similarly excellent yields and enantiometric ratios. Without the addition of amine, **3e** could be obtained in high yield and er. Surprisingly, more sterically hindered acyclic dialkyl amines **3f** and **3g** (compared to diethylamine) were effective nucleophiles in this reaction, indicating that the volatility of the resulting diethylamine byproduct is likely playing a larger role than steric hindrance in determining the chemoselectivity (*vide infra*). Enantiopure α-branched amine **2g** afforded the desired product **3g** and **3g’** with high er (>97:3) and dr (>20:1), demonstrating that the isomerization is not affected by the chirality of the nucleophile, but is instead controlled by the ligand. Importantly, no racemization of the chiral amine nucleophile occurred under the reaction conditions.

Under slightly modified conditions, primary aryl and alkyl amines were coupled with allylic diethylamine electrophiles to afford the chiral secondary amines, respectively. In these cases, NaBH_4_ proved to be a superior reductant than HCO_2_H. Both electron rich (**3i**) and poor (**3j**) anilines afforded the desired chiral amines with identically excellent enantiomeric ratios. In the presence of primary alkyl amines (with the exception of ^t^BuNH_2_ **2m**), the isomerization of allylic diethylamine was completely prohibited; therefore, a sequential addition of nucleophile was required to reach high yields. Primary alkyl amines, α to 1°(**3k**), 2°(**3l**), and 3° (**3m**) carbons, all afforded desired products with moderate to good yields and excellent enantioselectivity. A nucleophile containing a tethered tertiary nitrogen atom (**3n**) was well tolerated.

**Fig. 4:**
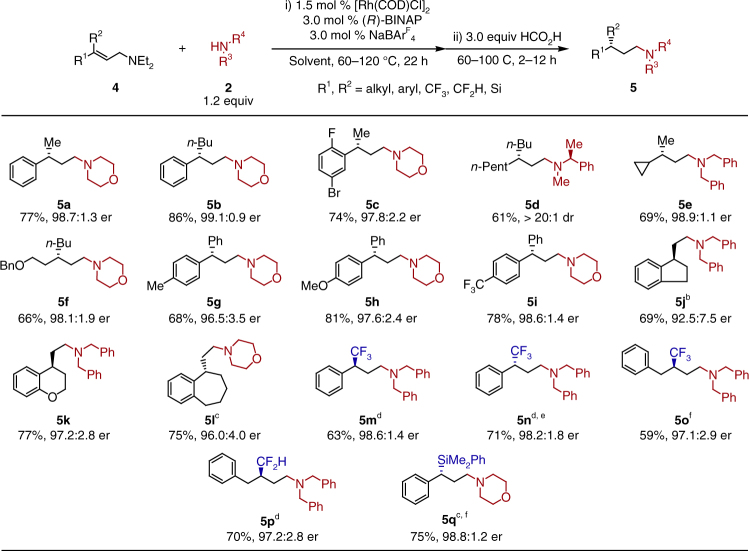
Scope of allylamine. a: General reaction conditions: allylic diethylamine **4** (0.24 mmol, 1.0 equiv, *E*/*Z* > 99:1 unless otherwise noted), nucleophile **2** (1.2 equiv), HCO_2_H (3.0 equiv), THF (1.2 M). b: Substrate *E*/*Z* = 96.7:3.3. c: Substrate *Z*/*E* > 99:1. d: 1,4-dioxane used. e: Substrate *Z*/*E* = 95.6:4.4. f: Toluene used. See supplementary methods for details. The absolute configuration of product is determined by alkene configuration

A survey of 3,3-disubstituted allylic amine electrophiles revealed that a wide variety of tertiary stereocenters can be installed under these reductive amination conditions (Fig. 4). Several 3,3-aryl,alkyl allylic diethylamines (**5a–c**) were tested and all afforded products with good yields and enantioselectivities. An *ortho* substituent on the aryl ring (**5c**) has no effect on the enantioselectivity of the isomerization, and the standard reaction conditions were amenable to aryl bromides, with no proteodebromination byproducts observed. The use of β,β-dialkyl allylic diethylamine (**5d–f**) was successful, enabling the highly enantioselective synthesis of γ-dialkyl amines, even with minimally differentiated substituents (**5d**, *n*Pent vs. *n*Bu). When more challenging 3,3-diaryl allylic diethylamines (**5g–i**) were subjected to the reaction conditions, amine products bearing γ-diaryl stereocenters, a common moiety in pharmaceutical agents, can be formed with excellent enantioselectivity^22–25^. Substrates bearing electron-rich (**5h**) and electron-poor (**5i**) aryl substituents afforded good yields and excellent enantiomeric ratios. This method can be used to set stereocenters containing sterically and electronically similar phenyl and *para*-tolyl groups with excellent selectivity (**5 g**, 96.5:3.5 er). Chiral γ-cyclic amines containing five-membered, six-membered, and seven-membered rings (**5j-l**) can be obtained as well with high enantioselectivity under identical conditions^26,27^.

Due to the superior reactivity and broad substrate tolerance of this catalyst, we sought to further develop this method for the construction of highly valuable stereocenters containing CF_3_, CF_2_H, and SiR_3_ substituents (Fig. 4). In order to effect suitable conversion, modification of the reaction solvent and increased temperatures were required. This may be attributed to the difficult isomerization of the more hindered allylic amines. Under these new conditions, difficult to synthesize enantiopure γ-trifluromethylated (**5m–o)** and difluoromethylated (**5p**) amines can be accessed with moderate to good yields and excellent enantioselectivities^28,29^. It is worth noting that the (*Z*)-CF_3_ allylic amine (**5n**) was slightly more reactive under these conditions compared to the (*E*)-isomer (**5m**), as higher conversion was observed for **5n**. Phenyldimethylsilyl substituted allylic diethylamines (**5q**) afforded good yields and enantioselectivities under these conditions as well. It is noteworthy that the chiral silyl group can be installed, as this can be converted to a range of functionalities^30^.

### Synthetic application

**Fig. 5:**
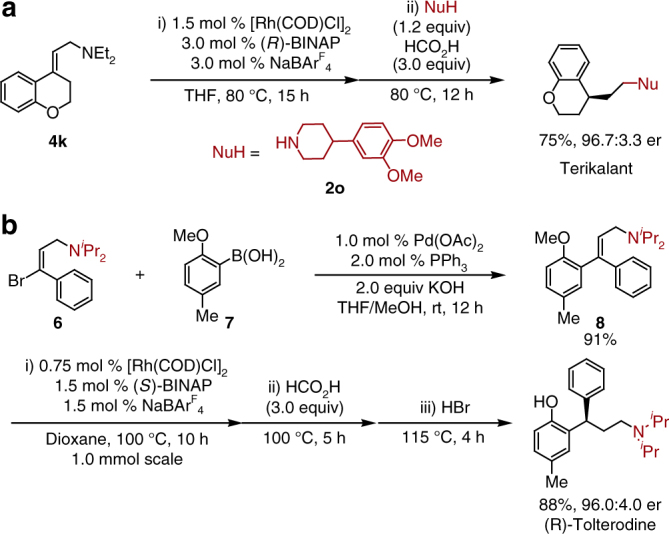
Synthetic application of rhodium-catalyzed asymmetric reductive amination of allylamines. **a** Enantioselective synthesis of Terikalant from allylic diethylamine **4k**. **b** Enantioselective and modular synthesis of (*R*)-Tolterodine from vinyl bromide **6**. See supplementary methods for details

This methodology was applied in the enantioselective syntheses of biologically active Terikalant and Tolterodine as illustrated in Fig. 5. Substrate **4k** and nucleophile **2o** were prepared according to literature procedures. The presence of **2o** proved to inhibit the isomerization of allylic amine **4k**. Therefore, the addition of nucleophile along with formic acid after the isomerization step was found effective, giving 75% yield as well as excellent er (96.7:3.3) for Terikalant (Fig. 5a), a significant improvement over the current synthesis utilizing chiral resolution^31^. A highly enantioselective synthesis of (*R*)-Tolterodine was then demonstrated in Fig. 5b^32–34^. The (*E*)-vinyl bromide **6**, prepared from *trans*-cinnamyl chloride^35^, was coupled with aryl boronic acid **7** to afford the diastereopure (*E*)-allylic amine **8** in 91% yield. A sequential addition of catalyst, hydrogen donor, and strong acid afforded the desired (*R*)-Tolterodine in 88% overall yield and 96.0:4.0 er. Although diisopropylamine was not a sufficient nucleophile to perform the enamine exchange with the diethyl enamine, the isomerization of allylic diisopropylamine **8** also proceeds in a highly enantioselective fashion. It is worth noting that the reaction was carried out on the 1.0 mmol scale with half the catalyst loading compared to the aminations performed on the smaller scale. Compared to state-of-the-art Tolterodine synthesis, which requires the *ortho*-hydroxyl substituent to direct the asymmetric hydrogenation^34^, our method allows for a modular and rapid synthesis of Tolterodine derivatives, including those without the *ortho*-hydroxyl functionality (**5g–i**).

### Control experiments and mechanistic discussion

**Fig. 6:**
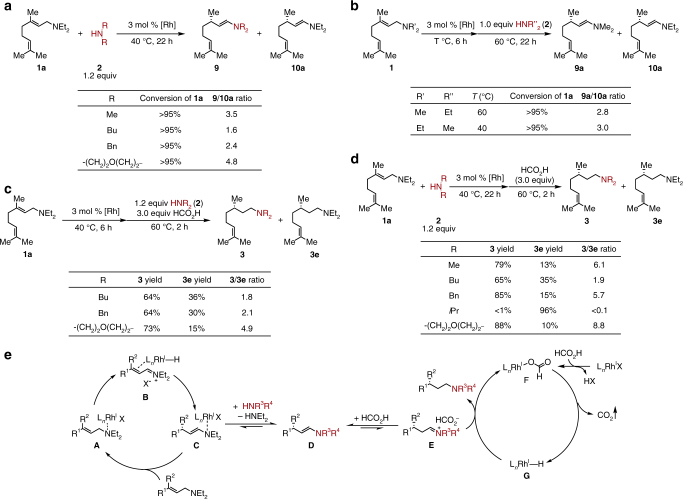
Control experiments and proposed catalytic cycles. **a** Selectivity of the enamine exchange step. **b** Thermodynamic equilibrium for the enamine exchange. **c** Selectivity of the transfer hydrogenation step (simultaneous addition of amine and hydrogen donor). **d** Chemoselectivity for various secondary amine nucleophiles under standard conditions. **e** Proposed catalytic cycles: enantioselective isomerization and transfer hydrogenation, X = BAr_4_^F^

To gain insight into the overall selectivity of this tandem process, a series of control reactions were carried out under optimized conditions (Fig. 6a–d). The selectivity of the enamine exchange step was first investigated. In general, less sterically hindered amine nucleophiles (compared to diethylamine) led to higher selectivity of desired product enamine **9** over the diethylenamine **10a** (Fig. 6a). For sterically similar dibutylamine and dibenzylamine, **9** was found to be the major product, presumably due to a combination of the relative amine volatilities, stoichiometry of the reaction, and enamine stability. When equimolar amounts of nucleophile and substrate were subjected to the reaction conditions, similar product distributions were observed regardless of the permutation of allylic amine versus nucleophile (Fig. 6b). This implies that the exchanging product distribution is controlled by a thermodynamic equilibrium under standard reaction conditions. When the nucleophiles and hydrogen donor were added simultaneously into the reaction after the isomerization step (Fig. 6c), the observed selectivities are similar to those shown in Fig. 6a, indicating that the exchange step is faster than the reduction. Finally, various secondary amine nucleophiles were studied under standard conditions (Fig. 6d). Higher selectivities were observed compared to those in Fig. 6a, implying that the reduction of desired enamine intermediates is faster than the diethylenamine **10a**. Therefore, the chemoselectivity of this two-step one-pot reaction comes from both steps, favoring the desired product. A proposed mechanism is shown in Fig. 6e: the basic nitrogen atom of the allylic amine substrate coordinates to the cationic rhodium to form **A**, followed by β-hydride elimination and re-insertion of in situ generated conjugated iminium **B** to afford the chiral enamine **C**. A thermally controlled enamine exchange leads to **D**, which then undergoes subsequent transfer hydrogenation upon addition of formic acid. A rhodium-mediated transfer hydrogenation mechanism is proposed, as lower conversion was observed in the absence of metal catalyst when investigating the reduction of pre-made enamine (see Supplementary Figure 5). An in situ formed rhodium formate species **F** can undergo decarboxylation to generate Rh hydride species **G**^36,37^. Subsequent iminium **E** inserts into Rh–H **G** to give the desired chiral γ-branched amine and regenerate rhodium formate **F**.

## Discussion

We have developed conditions for a highly enantioselective, modular synthesis of chiral γ-branched amines. This method enables a rapid assembly of various stereocenters as well as amine functionalities via a tandem isomerization–enamine exchange transfer hydrogenation process. Stereocenters bearing diaryl, cyclic, fluoroalkyl, and silyl substituents are established using same catalyst under similar conditions.

## Methods

### Direct asymmetric synthesis of γ-branched amines

[Rh(COD)Cl]_2_ (2.0 mg, 1.5 mol %), (*R*)-BINAP (4.5 mg, 3.0 mol %), NaBAr_4_^F^ (6.4 mg, 3.0 mol %), and THF (0.2 mL) were added to a oven-dried 4 mL vial equipped with a stir bar in the glove box under nitrogen atmosphere. To the vial was added sequentially allylic diethylamine (**1**, 0.24 mmol, 1.0 equiv), and secondary amine (**2**, 0.29 mmol, 1.2 equiv). The resulting solution was allowed to stir for 22 h at 40 °C (unless otherwise noted). After 22 h, formic acid (0.36 mmol, 3.0 equiv) was added into reaction vial via syringe and the reaction was allowed to stir for another 2 h at 60 °C (unless otherwise noted). The crude reaction was quenched by the addition of DCM, concentrated in vacuo and then purified by basic alumina chromatography to afford the desired product **3**.

### Data availability

All data is available from the corresponding authors upon reasonable request.

## Electronic supplementary material

Supplementary Information(PDF 8095 kb)
