# Supplementary material for: Asymmetric synthesis of γ-branched amines via rhodium-catalyzed reductive amination
Source: Nat Commun. 2018 Mar 22;9:1185. doi: 10.1038/s41467-018-03535-y (PMC5864842; doi:10.1038/s41467-018-03535-y)
Supplement: Supplementary file 1 — Supplementary Information(PDF 8095 kb) [file 41467_2018_3535_MOESM1_ESM.pdf]

# Asymmetric Synthesis of γ-Branched Amines *via* Rhodium-Catalyzed Reductive Amination

Wu et al.

## Supplementary methods

**General Experimental Information:** All reactions were carried out in flame-dried (or oven-dried at 140 °C for at least 2 h) glassware under an atmosphere of nitrogen unless otherwise indicated. Nitrogen was dried using a drying tube equipped with Drierite™ unless otherwise noted. Air- and moisture-sensitive reagents were handled in a nitrogen-filled glovebox (working oxygen level ~ 0.1 ppm). Column chromatography was performed with 1) basic aluminium oxide from ACROS Organics (50-200 µm, 60 A), Brockmann I grade, activated upon addition of certain amount of water according to the substrates, dry loading of activated aluminium oxide was applied followed by flush with eluent to get rid of air bubbles; 2) silica gel from Grace Davison Discovery Sciences (35-75 µm) with a column mixed as a slurry with the eluent and was packed, rinsed, and run under air pressure. Analytical thin-layer chromatography (TLC) was performed on precoated glass silica gel plates (by EMD Chemicals Inc.) with F-254 indicator. Visualization was either by short wave (254 nm) ultraviolet light, or by staining with potassium permanganate followed by brief heating on a hot plate or by a heat gun. Distillations were performed using a 3 cm short-path column under reduced pressure or by using a Hickman still at ambient pressure.

**Instrumentation:** $^{1}$H NMR and $^{13}$C NMR were recorded on a Varian Unity 400/500 MHz (100/125 MHz respectively for $^{13}$C) or a VXR-500 MHz spectrometer. Spectra were referenced using either $CDCl_3$ or $C_6D_6$ as solvents (unless otherwise noted) with the residual solvent peak as the internal standard ($^{1}$H NMR: δ 7.26 ppm, $^{13}$C NMR: δ 77.00 ppm for $CDCl_3$ and $^{1}$H NMR: δ 7.15 ppm, $^{13}$C NMR: δ 128.60 ppm for $C_6D_6$). Chemical shifts were reported in parts per million and multiplicities are as indicated: s (singlet,) d (doublet,) t (triplet,) q (quartet,) p (pentet,) m (multiplet,) and br (broad). Coupling constants, ***J***, are reported in Hertz and integration is provided, along with assignments, as indicated. Analysis by Gas Chromatography-Mass Spectrometry (GC-MS) was performed using a Shimadzu GC-2010 Plus Gas chromatograph fitted with a Shimadzu GCMS-QP2010 SE mass spectrometer using electron impact (EI) ionization after analytes traveled through a SHRXI–5MS- 30m x 0.25 mm x 0.25 µm column using a helium carrier gas. Data are reported in the form of m/z (intensity relative to base peak = 100). Gas Chromatography (GC) was performed on a Shimadzu GC-2010 Plus gas chromatograph with SHRXI–MS- 15m x 0.25 mm x 0.25 µm column with nitrogen carrier gas and a flame ionization detector (FID). Enantiomeric ratios were measured on Shimadzu Prominence HLPC system with SPD-M20A UV/VIS Photodiode array detector using Chiralpak IA-3, IB-3, IC-3, ID-3 or Chiralcel OJ-H columns. Low-resolution Mass Spectrometry and High Resolution Mass Spectrometry were performed in the Department of Chemistry at University of Illinois at Urbana-Champaign. The glove box, MBraun LABmaster sp, was maintained under nitrogen atmosphere. Melting points were recorded on a Thomas Hoover capillary melting point apparatus and are uncorrected.

**Materials:** Solvents used for extraction and column chromatography were reagent grade and used as received. Reaction solvents tetrahydrofuran (Fisher, unstabilized HPLC ACS grade), diethyl ether (Fisher, BHT stabilized ACS grade), methylene chloride (Fisher, unstabilized HPLC grade), dimethoxyethane (Fisher, certified ACS), toluene (Fisher, optima ACS grade), 1,4-dioxane (Fisher, certified ACS), acetonitrile (Fisher, HPLC grade), and hexanes (Fisher, ACS HPLC grade) were dried on a Pure Process Technology Glass Contour Solvent Purification System using activated Stainless Steel columns while following manufacture's recommendations for solvent preparation and dispensation unless otherwise noted. All amines were distilled and degassed by the freeze-pump-thaw method, and were stored under an atmosphere of nitrogen in glove box before use. All starting allylic diethylamine materials were distilled before use (often gave better reactivity after distillation).

## General procedure for trisubstituted allylic amine synthesis

Allylic diethylamine substrates **1a-1e, 4a, 4b, 4d, 4f, 4i, 4k** were synthesized according to our previous report. The $^{1}H$ and $^{13}C$ NMRs are marched with literature.[1]

Allylic diethylamine substrates **4c** and **4e** were synthesized by following method, modified from our previous report.[1]

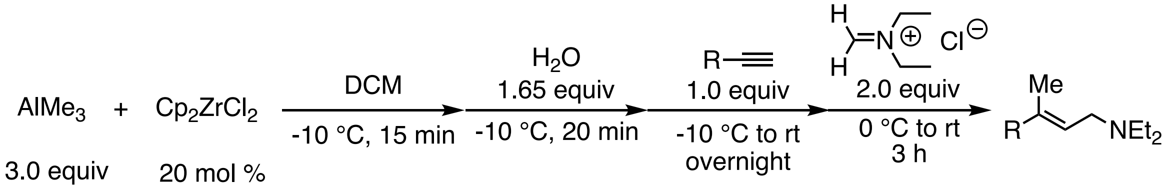


**Procedure**: To a dry 100 mL schlenk flask was charged with a stir bar and 0.292 g $Cp_2ZrCl_2$ (1 mmol, 20 mmol %), purged with nitrogen followed by the addition of 25 mL DCM. Cooled to -10 °C, 7.5 mL 2 M $AlMe_3$/hexanes solution (15 mmol, 3.0 equiv) was added slowly. The reaction was allowed to stir at -10 °C for 15 min followed by the slow addition of 168 μL $H_2O$ (8.2 mmol, 1.65 equiv). The resµting mixture was stirred vigorously at -10 °C for 20 min then added the alkyne (5 mmol, 1.0 equiv). The reaction flask was then was then warmed up to rt and stir overnight. A solution of the iminium chloride salt (10 mmol, 2 equiv) in 5 mL dry DCM was added slowly to the flask at 0 °C, then reaction was warmed up to rt and stir for another 3 hrs . The reaction is quenched by careful addition of 2 M NaOH solution at 0 °C, then filtered through ceilite and washed with warm DCM. The resulting mixture was then extract by DCM three time and combined organic layers were dried by $Na_2SO_4$, concentrated ***in vacuo***, and distilled under vacuum to afford desired allylic diethylamines.

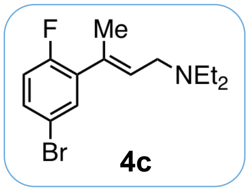


**(*E*)-3-(5-bromo-2-fluorophenyl)-*N,N*-diethylbut-2-en-1-amine (4c)**, prepared according to previously described procedure in 60% yield.

**$^{1}$H NMR** (500 MHz, $CDCl_3$) δ: 7.36 (dd, J = 6.8, 2.6 Hz, 1H), 7.30 (ddd, J = 8.7, 4.3, 2.6 Hz, 1H), 6.90 (dd, J = 10.2, 8.7 Hz, 1H), 5.73 (t, J = 6.6 Hz, 1H), 3.25 (d, J = 6.6 Hz, 2H), 2.58 (q, J = 7.2 Hz, 4H), 2.01 (s, 3H), 1.07 (t, J = 7.1 Hz, 6H).

**$^{13}$C NMR** (125 MHz, $CDCl_3$) δ: 159.06 (d, J = 247.5 Hz), 134.53 (d, J = 15.7 Hz), 132.48 (d, J = 4.6 Hz), 132.32 , 131.08 (d, J = 8.4 Hz), 130.28 , 117.57 (d, J = 24.6 Hz), 116.46 (d, J = 3.4 Hz), 50.92 , 47.20 , 17.20 (d, J = 3.8 Hz), 12.09.

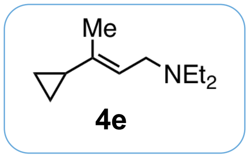


**(*E*)-3-cyclopropyl-*N,N*-diethylbut-2-en-1-amine (4e)**, prepared according to previously described procedure in 78% yield.

**$^{1}$H NMR** (500 MHz, $CDCl_3$) δ: 5.29 (t, ***J*** = 6.8 Hz, 3H), 3.05 (d, ***J*** = 6.8 Hz, 2H), 2.49 (q, ***J*** = 7.2 Hz, 4H), 1.54 (s, 3H), 1.42 – 1.33 (m, 1H), 1.02 (t, ***J*** = 7.2 Hz, 6H), 0.57 – 0.51 (m, 2H), 0.46 – 0.42 (m, 2H).

**$^{13}$C NMR** (125 MHz, $CDCl_3$) δ: 138.44, 120.19, 50.66, 46.81, 19.01, 14.54, 11.91, 4.61.

Allylic diethylamine substrate **4g** was synthesized by following method[2] and the starting vinyl bromide was synthesized according to our previous report[1] and literature[2].

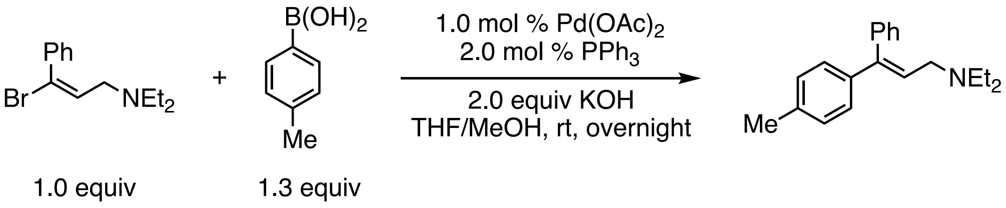


**Procedure**: To a 50 ml round bottom flask was charged with a stir bar and 11 mg $Pd(OAc)_2$ (0.050 mmol, 1.0 mol %), 26 mg $PPh_3$ (0.10 mmol, 2.0 mol %), 0.560 g KOH (10 mmol, 2.0 equiv), starting material vinyl bromide (1.34g, 5 mmol, 1.0 equiv) ,0.880 g 4-methyl boronic acid (6.5 mmol, 1.3 equiv) and 5 mL THF and 5 mL MeOH. The reaction was stirred at rt overnight followed by dilution with EtOAc, and washed by 1 N NaOH solution and brine. The organic layer was then dried over $MgSO_4$, concentrated ***in vacuo***, purified by $Al_2O_3$ column chromatography: 200 g $Al_2O_3$ + 12 g $H_2O$, 30 : 1 hexanes/ EtOAc with 0.5% MeOH as eluent.

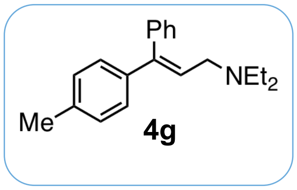


**(*E*)-*N,N*-diethyl-3-phenyl-3-(p-tolyl)prop-2-en-1-amine (4g)**, prepared according to previously described procedure in 70% yield.

**$^1$H NMR** (500 MHz, $CDCl_3$) δ: 7.40 – 7.34 (m, 2H), 7.33 – 7.28 (m, 1H), 7.20 – 7.12 (m, 4H), 7.11 – 7.05 (m, 2H), 6.19 (t, J = 6.7 Hz, 1H), 3.15 (d, J = 6.7 Hz, 2H), 2.52 (q, J = 7.1 Hz, 4H), 2.32 (s, 3H), 0.96 (t, J = 7.1 Hz, 6H).

**$^{13}$C NMR** (125 MHz, $CDCl_3$) δ: 143.23, 140.11, 139.65, 137.01, 129.97, 128.96, 128.21, 127.27, 127.15, 126.55, 51.86, 47.13, 21.20, 11.96.

The cyclic allylic diethylamine substrates **4j and 4l** were synthesized by following method[3] and the starting diethyl (2-(diethylamino)-2-oxoethyl) phosphonate was synthesized according to our previous report[1] and literature.[3]

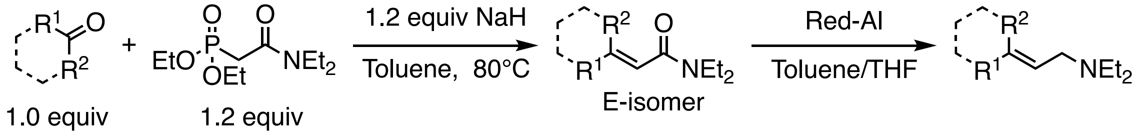


**Olefination**: A dry 100mL round-bottom flask was charged with a stir bar and 0.48g NaH (60 wt %, 12 mmol, 1.2 equiv), purged with nitrogen followed by the addition of 15 mL toluene. Cooled to 0 °C, diethyl (2-(diethylamino)-2-oxoethyl)phosphonate was added dropwise (2.8 mL, 12 mmol, 1.2 equiv). The reaction was allowed to stir at 0 °C for 30 min until the solution become clear. Ketone was added dropwise (10 mmol, 1.0 equiv) to the reaction over 5 min, then warmed up to 80 °C, stirring overnight. The reaction was quenched with sat. $NH_4Cl$ solution, and the aqueous layer was extracted with DCM three times. The combined organic layers were dried over $MgSO_4$, and purified by silica column chromatography.

**Reduction**: To a dry 20 mL round-bottom flask was charged with a stir bar, purged with $N_2$ three times, followed by the addition of unsaturated amide (4.0 mmol), dry THF (3 mL) and dry toluene (6 mL, V(tol)/V(THF)=2). The flask was then cooled in ice bath, and added RedAl solution (2.0 equiv, 3.5 M) dropwised. The reaction was allowed to stir at 0 °C for 2 hours then warmed up to rt for another 4 hours. The reaction crude was cooled in ice bath and quenched by the addition of 10 mL 5 M NaOH solution and 20 mL toluene. After stirring for 30 minutes, the crude was transferred to a separatory funnel. Organic layer was separated, washed by 5 M NaOH solution twice, dried over $MgSO_4$, concentrated ***in vacuo*** and further purified by $Al_2O_3$ column chromatography.

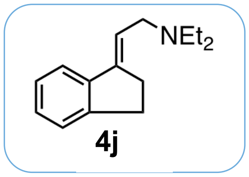


**(*E*)-2-(2,3-dihydro-1H-inden-1-ylidene)-*N,N*-diethylethan-1-amine** (**4j**), prepared according to previously described procedure at 25% overall yield.

**$^1$H NMR** (500 MHz, $CDCl_3$) δ: 7.51 – 7.44 (m, 1H), 7.25 – 7.23 (m, 1H), 7.21 – 7.14 (m, 2H), 6.05 (ddd, J = 7.0, 4.3, 2.6 Hz, 1H), 3.25 (d, J = 6.8 Hz, 2H), 3.06 – 2.90 (m, 2H), 2.80 – 2.71 (m, 2H), 2.58 (q, J = 7.2 Hz, 4H), 1.07 (t, J = 7.2 Hz, 6H).

**$^{13}$C NMR** (125 MHz, $CDCl_3$) δ: 146.06, 144.18, 141.52, 127.87, 126.57, 125.38, 120.33, 116.74, 52.33, 47.08, 30.28, 28.12, 12.04.

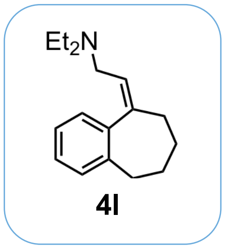


**(*Z*)-*N,N*-diethyl-2-(6,7,8,9-tetrahydro-5H-benzo[7]annulen-5-ylidene)ethan-1-amine** (**4l**), prepared according to previously described procedure at 46% overall yield.

**$^1$H NMR** (500 MHz, $CDCl_3$) δ: 7.17 – 7.09 (m, 3H), 7.02 – 6.97 (m, 1H), 5.64 (t, J = 6.8 Hz, 1H), 3.00 (d, J = 6.8 Hz, 2H), 2.77 – 2.67 (m, 2H), 2.47 (q, J = 7.1 Hz, 4H), 2.33 – 2.24 (m, 2H), 1.85 (p, J = 5.9 Hz, 2H), 1.72 – 1.61 (m, 2H), 0.92 (t, J = 7.1 Hz, 6H).

**$^{13}$C NMR** (125 MHz, $CDCl_3$) δ: 145.62, 141.73, 141.18, 129.12, 128.98, 126.92, 125.59, 124.99, 51.08, 46.81, 38.10, 36.63, 33.30, 27.87, 11.76.ppm.

The (***E***)-selective β-$CF_3$ or $CF_2H$ substituted allylic diethylamine substrates **4m, 4o,** and **4p** were synthesized by following method.[4]

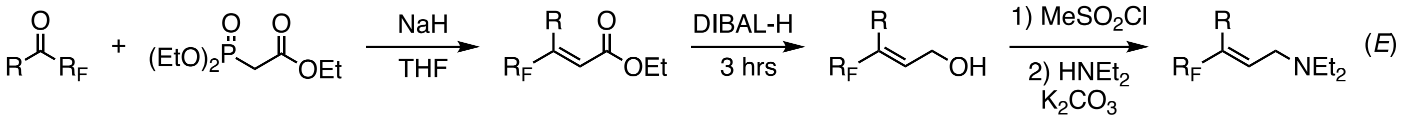


**Olefination**: A dry 100mL round-bottom flask was charged with a stir bar and 0.60 g NaH (60 wt %, 15 mmol, 1.5 equiv), purged with nitrogen followed by the addition of 30 mL THF. Cooled to 0 °C, ethyl 2-(diethoxyphosphoryl)acetate was added dropwise (3.0 mL, 15 mmol, 1.5 equiv). The reaction was allowed to stir at 0 °C for 30 min until the solution become clear. Fluoroakyl ketone was added dropwise (10 mmol, 1.0 equiv) to the reaction over 5 min, then warmed up to 50 °C, stirring overnight. The reaction was quenched with sat. $NH_4Cl$ solution, and the aqueous layer was extracted with DCM

three times. The combined organic layers were dried over $MgSO_4$, concentrated ***in vacuo*** and purified by silica column chromatography to affored (E)-$R_F$-substituted allylic ester. (Yields: 60% to 80% for desired isomer)

**Reduction:** To a dry 250 mL round-bottom flask was charged with a stir bar, purged with $N_2$ three times, followed by the addition of unsaturated ester (4.8 mmol), dry THF (24 mL). The flask was then cooled in ice bath, then added DIBAL-H solution (2.5 equiv, 1 M in hexanes) dropwise. The reaction was allowed to stir at 0 °C for 2 hours then quenched by the addition of 10 mL sat. Rochelle salt solution. After stirring at rt overnight, the crude was extracted with $Et_2O$ three times, combined organic lay dried over $MgSO_4$, concentrated ***in vacuo*** and used for next step without further purification.

**Chlorination**: To a dry 50 mL round-bottom flask was charged with a stir bar, purged with $N_2$ three times, followed by the addition of allylic alcohol (4.6 mmol), dry DCM (20 mL), and 1.9 mL $Et_3N$ (13.8 mmol, 3.0 equiv). The flask was then cooled in ice bath, then added $MeSO_2Cl$ (13.8 mmol, 3.0 equiv) dropwise. The reaction was allowed to stir at 0 °C for 5 hours followed by the addition of another 2.0 equiv of $MeSO_2Cl$. The resulting mixture was then warmed up to rt, and stirred overnight. The reaction crude was diluted in DCM, washed sequentially with 1 N HCl solution, sat. $NaHCO_3$ solution and brine. The organic layer was then dried over $MgSO_4$, concentrated ***in vacuo*** and purified by silica column chromatography to affored the corresponding allylic chloride. (Yield: 85% to 95%, two steps)

**$S_N$2**: To a dry 50 mL round-bottom flask was charged with a stir bar, allylic chloride (4.0 mmol), $HNEt_2$ (6.0 mmol, 1.5 equiv), $K_2CO_3$ (10 mmol, 2.5 equiv), and 22 mL acetone. The reaction mixture was then refluxed under $N_2$ at 70 °C overnight. The reaction crude was then filtered through celite, concentrated ***in vacuo*** to remove solvent, re-diluted in $Et_2O$, extracted with 1 N HCl three time. The aqueous layer was then basified by the addition of 3 N NaOH solution, (pH>11) and extracted with DCM three times. The combined DCM layers were $MgSO_4$, concentrated ***in vacuo*** and distilled under vacuum to afford the desired allylic amines (Yields: 82% to 88%)

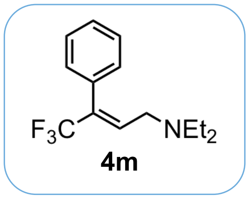


**(*E*)-*N,N*-diethyl-4,4,4-trifluoro-3-phenylbut-2-en-1-amine (4m)**, prepared according to previously described procedure.

**¹H NMR** (500 MHz, $CDCl_3$) δ: 7.44 – 7.36 (m, 3H), 7.25 – 7.21 (m, 2H), 6.55 (ddt, J = 6.6, 5.0, 1.6 Hz, 1H), 3.13 – 2.94 (m, 2H), 2.46 (q, J = 7.1 Hz, 4H), 0.95 (t, J = 7.1 Hz, 6H).

**¹³C NMR** (125 MHz, $CDCl_3$) δ: 134.83 (q, J = 5.3 Hz), 132.38 (q, J = 29.7 Hz), 132.04, 129.52, 128.57, 128.40, 123.22 (q, J = 273.2 Hz), 50.39, 47.12, 11.81.

**¹⁹F NMR** (471 MHz, $CDCl_3$) δ -65.91 (d, J = 1.9 Hz).

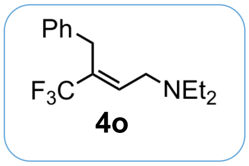


**(*E*)-3-benzyl-*N,N*-diethyl-4,4,4-trifluorobut-2-en-1-amine (4o)**, prepared according to previously described procedure.

**¹H NMR** (500 MHz, $CDCl_3$) δ: 7.32 – 7.27 (m, 2H), 7.24 – 7.20 (m, 1H), 7.19 – 7.16 (m, 2H), 6.45 (t, J = 6.2 Hz, 1H), 3.60 (s, 2H), 3.18 (dq, J = 5.1, 2.4 Hz, 2H), 2.49 (q, J = 7.1 Hz, 4H), 1.00 (t, J = 7.1 Hz, 6H).

**¹³C NMR** (125 MHz, $CDCl_3$) δ: 137.92, 135.13 (q, J = 5.8 Hz), 129.40 (q, J = 28.6 Hz), 128.77, 128.33, 126.71, 124.30 (q, J = 273.4 Hz), 50.52, 47.45, 31.89, 12.11.

$^{19}$**F NMR** (471 MHz, $CDCl_3$) δ: -67.01 (d, J = 2.2 Hz).

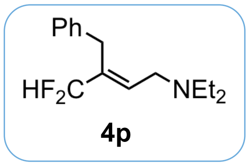

**(*E*)-3-benzyl-*N,N*-diethyl-4,4-difluorobut-2-en-1-amine (4p)**, prepared according to previously described procedure.

$^{1}$**H NMR** (500 MHz, $CDCl_3$) δ: 7.31 – 7.26 (m, 2H), 7.22 – 7.17 (m, 3H), 6.10 – 6.05 (m, 1H), 5.99 (t, J = 56.1 Hz, 1H), 3.57 (s, 2H), 3.17 (dt, J = 6.8, 3.7 Hz, 2H), 2.49 (q, J = 7.1 Hz, 3H), 1.00 (t, J = 7.2 Hz, 6H).

$^{13}$**C NMR** (125 MHz, $CDCl_3$) δ: 138.58, 134.22 (t, J = 9.9 Hz), 133.73 (t, J = 20.5 Hz), 128.63, 128.51, 126.41, 117.00 (t, J = 237.6 Hz), 50.37, 47.25, 31.10 (t, J = 1.8 Hz), 11.96.

$^{19}$**F NMR** (471 MHz, $CDCl_3$) δ: -114.38 (d, J = 55.8 Hz).

The (***Z***)-selective β-$CF_3$ substituted allylic diethylamine substrates **4n** was synthesized by following method.[5]

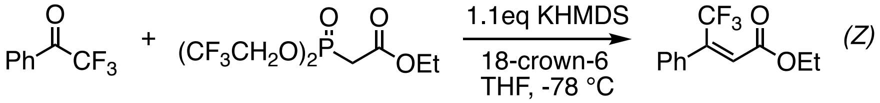


**Olefination**: A dry 50 mL round-bottom flask was charged with a stir bar and 1.76 g KHMDS (8.8 mmol, 1.1 equiv) and 2.56 g 18-crown-6 (9.6 mmol, 1.2 equiv) purged with nitrogen followed by the addition of 15 mL THF. Cooled to -78 °C, ethyl 2-(bis(2,2,2-trifluoroethoxy)phosphoryl)acetate was added dropwise (8.8 mmol, 1.1 equiv). The reaction was allowed to stir at -78 °C for 45 min followed by the addition of trifluoroacetophenone (8.0 mmol, 1.0 equiv) to the reaction, stirred at -78 °C for another 3 h then warmed up to rt, quenched with sat. $NH_4Cl$ solution, and the aqueous layer was extracted with DCM three times. The combined organic layers were dried over $MgSO_4$, concentrated ***in vacuo*** and purified by silica column chromatography to affored (Z)-$R_F$-substituted allylic ester at 58% yield.

**Reduction**, **Chlorination, and SN2** were carried out under same conditions as described above.

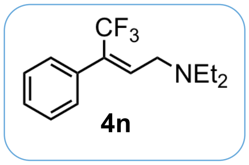

**(*Z*)-*N,N*-diethyl-4,4,4-trifluoro-3-phenylbut-2-en-1-amine (4n)**, prepared according to previously described procedure. Purity: Z/E=22:1.

$^{1}$**H NMR** (500 MHz, $CDCl_3$) δ: δ 7.42 – 7.30 (m, 5H), 6.19 (td, J = 6.2, 0.9 Hz, 1H), 3.49 (dq, J = 5.8, 2.8 Hz, 2H), 2.58 (q, J = 7.1 Hz, 4H), 1.07 (t, J = 7.1 Hz, 6H).

$^{13}$**C NMR** (125 MHz, $CDCl_3$) δ: 140.93 (q, J = 2.8 Hz), 136.28 (q, J = 1.8 Hz), 132.15 (q, J = 30.5 Hz), 128.41, 128.29, 128.15, 124.03 (q, J = 275.7 Hz), 51.29 (q, J = 2.4 Hz), 47.49, 12.09.

$^{19}$**F NMR** (471 MHz, $CDCl_3$) δ: -57.30 (d, J = 3.1 Hz).

β-Silyl substituted allylic diethylamine substrate **4q** was synthesized by following method, modified from literature.[6]

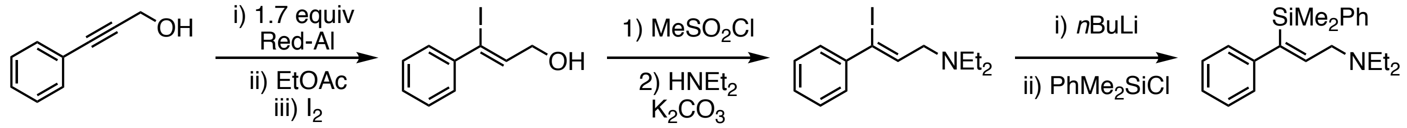


**Hydroalumination**:[6] To a dry 100 mL schlenk flask was charged with a stir bar, purged with $N_2$ three times, followed by the addition of dry THF (30 mL) and 2.8 mL RedAl solution (8.5 mmol, 1.7 equiv). The flask was then cooled in ice bath, then added 5 mL THF solution of 3-phenyl-2-propyn-1-ol (5.0 mmol, 1.0 equiv) dropwise. The reaction was allowed to warm up to rt and stir for 4 hours. Then, the reaction flask was then cooled to $^{-}$10 °C followed by the slow addition of 2.0 mL EtOAc to quench excess Red-Al then stirred at -10 °C for another 15 min. The resulting mixture was then cooled to $^{-}$78 °C, followed by the addition of $I_2$ (10 mmol, 2.0 equiv) in one portion under nitrogen flow. The reaction crude was then allowed to stir at $^{-}$78 °C for another hour before being quenched by 15 mL sat. Rochelle salt solution and 25 mL sat. $Na_2S_2O_3$ solution at 0 °C. The biphasic mixture was then stirred vigorously at rt overnight, and extracted by $Et_2O$ three times. The combined organic layer was then dried over $MgSO_4$, concentrated ***in vacuo*** and used for next step without further purification.

**Chlorination and $SN_2$** were carried out under same conditions as described above.

**Vinyl silane synthesis**: To a dry 200 mL schlenk flask was charged with a stir bar, purged with $N_2$ three times, followed by the addition of dry THF (25 mL) and starting vinyl iodine (5.0 mmol, 1.0 equiv). The flask was then cooled to $^{-}$78 °C, followed by the slow addition of nBuLi (12 mmol, 2.4 equiv) over 10 min. The resulting crude was allowed to stir at $^{-}$78 °C for another 30 min, before the addition of chloro(dimethyl)phenylsilane (15 mmol, 3.0 equiv). The resulting mixture was allowed to stir at $^{-}$78 °C for another 2 hours followed by being quenched with sat. $NaHCO_3$ solution, extracted by $Et_2O$ three times. The combined organic layer was then dried over $MgSO_4$, concentrated ***in vacuo*** and further purified by $Al_2O_3$ column chromatography.

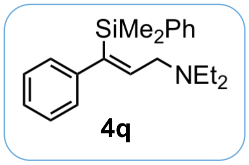


**(*Z*)-3-(dimethyl(phenyl)silyl)-*N,N*-diethyl-3-phenylprop-2-en-1-amine (4q)**, prepared according to previously described procedure.

**$^1$H NMR** (500 MHz, $CDCl_3$) δ: δ 7.62 – 7.55 (m, 2H), 7.35 (dd, J = 4.8, 1.9 Hz, 3H), 7.29 – 7.23 (m, 2H), 7.21 – 7.14 (m, 1H), 7.12 – 7.05 (m, 2H), 6.33 (t, J = 6.3 Hz, 1H), 3.10 (d, J = 6.3 Hz, 2H), 2.40 (q, J = 7.1 Hz, 4H), 0.91 (t, J = 7.1 Hz, 6H), 0.34 (s, 6H).

**$^{13}$C NMR** (125 MHz, $CDCl_3$) δ: 146.66, 146.50, 142.37, 139.34, 133.99, 129.11, 128.02, 127.97, 127.72, 125.73, 54.79, 46.89, 12.04, -0.34.

**General procedure for Rh-catalyzed reductive amination of allylic diethylamine with secondary amine nucleophiles (General procedure A)**

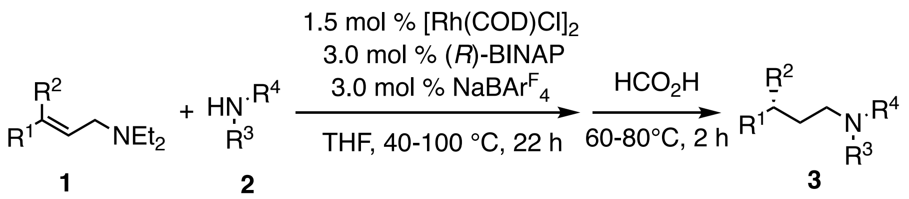


**General procedure A**: $[Rh(COD)Cl]_2$ (2.0 mg, 0.0036 mmol, 1.5 mol %), (***R***)-BINAP (4.5 mg, 0.0072 mmol, 3.0 mol %), $NaBAr_4^F$ (6.4 mg, 0.0072 mmol, 3.0 mol %), and THF (0.2 mL) were added to a 4 mL vial equipped with a stir bar in the glove box under nitrogen atmosphere. To the vial was added sequentially allylic diethylamine (**1**, 0.24 mmol, 1.0 equiv), and secondary amine (**2**, 0.29 mmol, 1.2 equiv). The resulting solution was allowed to stir for 22 h at 40 °C (unless otherwise noted). After 22 h, formic acid (0.36 mmol, 3.0 equiv) was added into reaction vial via syringe and the reaction was allowed to stir for another 2 h at 60 °C (unless otherwise noted). The reaction crude was quenched by the addition of DCM, concentrated ***in vacuo*** and then purified by basic alumina chromatography to afford the desired product **3**.

**General procedure for Rh-catalyzed reductive amination of allylic diethylamine with aryl amine nucleophiles (General procedure B)**

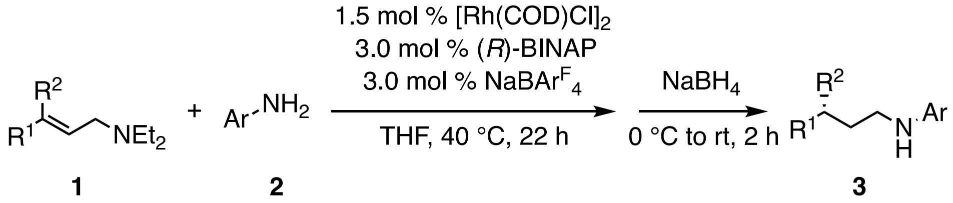


**General procedure B**: $[Rh(COD)Cl]_2$ (2.0 mg, 0.0036 mmol, 1.5 mol %), (***R***)-BINAP (4.5 mg, 0.0072 mmol, 3.0 mol %), $NaBAr_4^F$ (6.4 mg, 0.0072 mmol, 3.0 mol %), and THF (0.2 mL) were added to a 4 mL vial equipped with a stir bar in the glove box under nitrogen atmosphere. To the vial was added sequentially allylic diethylamine (**1**, 0.24 mmol, 1.0 equiv), and aryl amine (**2**, 0.29 mmol, 1.2 equiv). The resulting solution was allowed to stir for 22 h at 40 °C (unless otherwise noted). After 22 h, the reaction vial was cooled to 0 °C followed by the addition of $NaBH_4$ (0.18 mmol, 1.5 equiv) and 1.0 ml MeOH. The resulting mixture was allowed to stir at 0 °C for 1 h then warmed up to rt for another 1 h. The crude reaction was quenched by the addition of DCM, concentrated ***in vacuo*** and then re-dissolved in DCM, washed with sat. $NaHCO_3$ solution. The organic layer was dried over $MgSO_4$, concentrated ***in vacuo***, and purified by silica gel chromatography to afford the desired product **3**.

**General procedure for Rh-catalyzed reductive amination of allylic diethylamine with primary alkyl amine nucleophiles (General procedure C)**

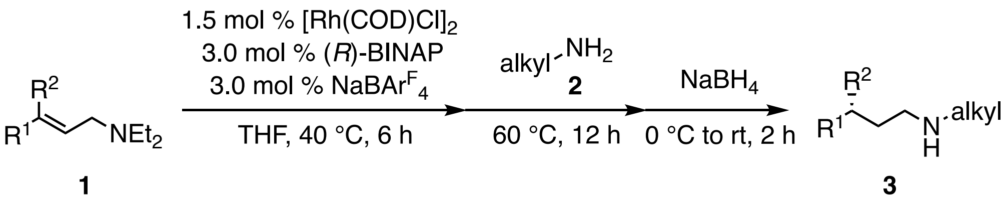

**General procedure C**: $[Rh(COD)Cl]_2$ (2.0 mg, 0.0036 mmol, 1.5 mol %), (***R***)-BINAP (4.5 mg, 0.0072 mmol, 3.0 mol %), $NaBAr_4^F$ (6.4 mg, 0.0072 mmol, 3.0 mol %), THF (0.2 mL), and allylic diethylamine (**1**, 0.24 mmol, 1.0 equiv) were added to a 4 mL vial equipped with a stir bar in the glove box under nitrogen atmosphere. The resulting solution was allowed to stir for 6 h at 40 °C (unless otherwise noted), followed by the addition of primary alkyl amine (**2**, 0.29 mmol, 1.2 equiv) then continued stirring at 60 °C for another 12 h. After 12 h, the reaction vial was cooled to 0 °C followed by the addition of $NaBH_4$ (0.18 mmol, 1.5 equiv) and 1.0 ml MeOH. The resulting mixture was allowed to stir at 0 °C for 1 h then warmed up to rt for another 1 h. The reaction crude was then quenched by the addition of DCM, concentrated ***in vacuo*** and then re-dissolved in DCM, washed with sat. $NaHCO_3$ solution. The organic layer was dried over $MgSO_4$, concentrated ***in vacuo***, and purified by basic alumina chromatography to afford the desired product **3**.

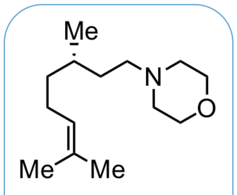

**3a**

**(*S*)-4-(3,7-dimethyloct-6-en-1-yl)morpholine (3a, Figure 3):** Prepared according to General procedure A from geranyl diethyl amine (**1a**) with morpholine (**2a**) in 80% isolated yield.

**Column Chromatography Condition:** 100 g $Al_2O_3$ + 9 g $H_2O$, 30 : 1 hexanes/ EtOAc with 0.5% MeOH to 15 : 1 hexanes/ EtOAc with 1.0% MeOH as gradient eluent.

**$^1$H NMR** (500 MHz, $CDCl_3$) δ: 5.09 (t, J = 7.1 Hz, 1H), 3.71 (t, J = 4.7 Hz, 4H), 2.49 – 2.39 (m, 4H), 2.40 – 2.26 (m, 2H), 2.08 – 1.87 (m, 2H), 1.68 (s, 3H), 1.60 (s, 3H), 1.52 (ddt, J = 12.5, 10.3, 5.5 Hz, 1H), 1.48 – 1.40 (m, 1H), 1.37 – 1.27 (m, 2H), 1.17 (m, 1H), 0.89 (d, J = 6.6 Hz, 3H).

**$^{13}$C NMR** (125 MHz, $CDCl_3$) δ: 131.33, 124.93, 67.19, 57.41, 54.07, 37.37, 33.72, 31.19, 25.86, 25.62, 19.86, 17.80.

**HRMS** (ESI-TOF) ***m/z***: [M+H$^+$] calculated for $C_{14}H_{28}NO$, 226.2171; found, 226.2175.

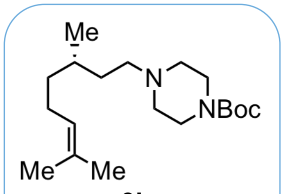

**3b**

**tert-butyl (*S*)-4-(3,7-dimethyloct-6-en-1-yl)piperazine-1-carboxylate (3b, Figure 3):** Prepared according to General procedure A from geranyl diethyl amine (**1a**) with ***tert***-butyl piperazine-1-carboxylate (**2b**) in 75% isolated yield.

**Column Chromatography Condition:** 100 g $Al_2O_3$ + 9 g $H_2O$, 30 : 1 hexanes/ EtOAc with 0.5% MeOH as eluent.

**$^1$H NMR** (500 MHz, $CDCl_3$) δ: 5.08 (t, J = 7.0 Hz, 1H), 3.43 (m, 4H), 2.46 – 2.22 (m, 6H), 2.09 – 1.85 (m, 2H), 1.68 (s, 3H), 1.59 (s, 3H), 1.55 – 1.49 (m, 2H), 1.45 (s, 9H), 1.31 (m, 2H), 1.22 – 1.10 (m, 1H), 0.88 (d, J = 6.5 Hz, 3H).

**$^{13}$C NMR** (125 MHz, $CDCl_3$) δ: 154.91, 131.34, 124.91, 79.68, 56.98, 53.31, 37.35, 33.94, 31.20, 28.58, 25.86, 25.61, 19.84, 17.80.

**HRMS** (ESI-TOF) ***m/z***: [M+H$^+$] calculated for $C_{19}H_{37}N_2O_2$, 325.2855; found, 325.2850.

Nucleophiles **2c** and **2d** were observed to slow down the isomerization of allylic amine **1a**, therefore the addition of **2c** or **2d** together with formic acid led to increased conversion of **1a**.

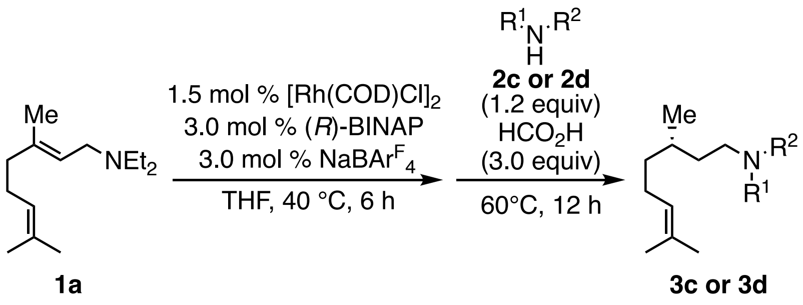


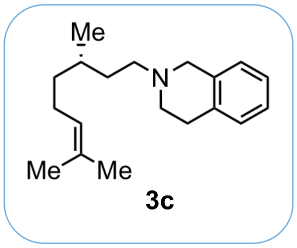


**(*S*)-2-(3,7-dimethyloct-6-en-1-yl)-1,2,3,4-tetrahydroisoquinoline (3c, Figure 3):** Prepared according to modified General procedure A from geranyl diethyl amine (**1a**) with 1,2,3,4-tetrahydroisoquinoline (**2c**) in 66% isolated yield.

**Column Chromatography Condition:** 100 g $Al_2O_3$ + 6 g $H_2O$, 50 : 1 hexanes/ EtOAc with 0.5% MeOH to 30 : 1 hexanes/ EtOAc with 1.0% MeOH as gradient eluent.

**$^1$H NMR** (500 MHz, $CDCl_3$) δ: 7.16 – 7.07 (m, 3H), 7.05 – 6.97 (m, 1H), 5.11 (t, J = 6.9 Hz, 1H), 3.63 (s, 2H), 2.91 (t, J = 6.0 Hz, 2H), 2.73 (td, J = 6.0, 3.1 Hz, 2H), 2.52 (dt, J = 9.5, 5.5 Hz, 2H), 2.10 – 1.88 (m, 2H), 1.69 (s, 3H), 1.67 – 1.63 (m, 1H), 1.61 (s, 3H), 1.55 – 1.48 (m, 1H), 1.46 – 1.32 (m, 2H), 1.24 – 1.15 (m, 1H), 0.93 (d, J = 6.6 Hz, 3H)..

**$^{13}$C NMR** (125 MHz, $CDCl_3$) δ: 135.09, 134.53, 131.30, 128.76, 126.73, 126.17, 125.66, 125.00, 56.73, 56.49, 51.25, 37.44, 34.39, 31.27, 29.30, 25.88, 25.66, 19.91, 17.82.

**HRMS** (ESI-TOF) ***m/z***: [M+H$^+$] calculated for $C_{19}H_{30}N$, 272.2378; found, 272.2377.

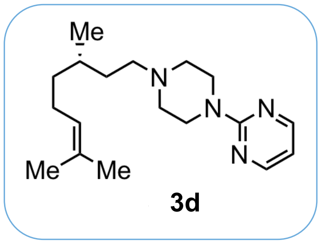


**(*S*)-2-(4-(3,7-dimethyloct-6-en-1-yl)piperazin-1-yl)pyrimidine (3d, Figure 3):** Prepared according to modified General procedure A from geranyl diethyl amine (**1a**) with 2-(piperazin-1-yl)pyrimidine (**2d**) in 83% isolated yield.

**Column Chromatography Condition:** 100 g $Al_2O_3$ + 9 g $H_2O$, 30 : 1 hexanes/ EtOAc with 0.5% MeOH to 15 : 1 hexanes/ EtOAc with 1.0% MeOH as gradient eluent.

**$^1$H NMR** (500 MHz, $CDCl_3$) δ: 8.30 (d, J = 4.7 Hz, 2H), 6.47 (t, J = 4.7 Hz, 1H), 5.09 (t, J = 7.0 Hz, 1H), 3.92 – 3.76 (br, 4H), 2.54 – 2.45 (br, 4H), 2.44 – 2.30 (m, 2H), 2.09 – 1.87 (m, 2H), 1.68 (s, 3H), 1.60 (s, 3H), 1.58 – 1.53 (m, 1H), 1.51 – 1.43 (m, 1H), 1.39 – 1.29 (m, 2H), 1.22 – 1.13 (m, 1H), 0.90 (d, J = 6.6 Hz, 3H).

**$^{13}$C NMR** (125 MHz, $CDCl_3$) δ: 161.83, 157.83, 131.34, 124.93, 190.91 57.10, 53.41, 43.84, 37.37, 34.01, 31.27, 25.87, 25.63, 19.87, 17.81.

**HRMS** (ESI-TOF) ***m/z***: [M+H$^+$] calculated for $C_{18}H_{31}N_4$, 303.2549; found, 303.2549.

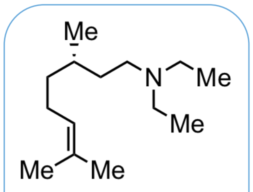
3e

**(S)-N,N-diethyl-3,7-dimethyloct-6-en-1-amine (3e, Figure 3):** Prepared according to General procedure A from geranyl diethyl amine (**1a**) without any nucleophilic amine added in 83% isolated yield.

**Column Chromatography Condition:** 100 g $Al_2O_3$ + 9 g $H_2O$, 30 : 1 hexanes/ EtOAc with 0.5% MeOH as eluent.

**$^1$H NMR** (500 MHz, $CDCl_3$) δ: 5.09 (t, J = 7.1 Hz, 1H), 2.51 (q, J = 7.1, 4H), 2.46 – 2.36 (m, 2H), 1.97 (qq, J = 14.5, 7.1 Hz, 2H), 1.67 (d, J = 1.6 Hz, 3H), 1.59 (s, 3H), 1.53 – 1.38 (m, 2H), 1.36 – 1.21 (m, 2H), 1.16 (m, 1H), 1.01 (t, J = 7.1 Hz, 6H), 0.88 (d, J = 6.5 Hz, 3H).

**$^{13}$C NMR** (125 MHz, $CDCl_3$) δ: 131.21, 125.05, 50.97, 47.05, 37.44, 34.01, 31.31, 25.87, 25.66, 19.90, 17.77, 11.84.

**HRMS** (ESI-TOF) ***m/z***: [M+H$^+$] calculated for $C_{14}H_{30}N$, 212.2378; found, 212.2385.

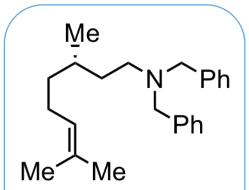
3f

**(S)-N,N-dibenzyl-3,7-dimethyloct-6-en-1-amine (3f, Figure 3):** Prepared according to General procedure A from geranyl diethyl amine (**1a**) with dibenzylamine (**2f**) in 70% isolated yield.

**Column Chromatography Condition:** silica gel, 20 : 1 hexanes/ EtOAc as eluent.

**$^1$H NMR** (500 MHz, $CDCl_3$) δ: δ 7.39 – 7.34 (m, 4H), 7.33 – 7.28 (m, 4H), 7.24 – 7.18 (m, 2H), 5.06 (tq, J = 7.1, 1.4 Hz, 1H), 3.58 (d, J = 13.7 Hz, 2H), 3.50 (d, J = 13.7 Hz, 2H), 2.43 (t, J = 7.3 Hz, 2H), 2.04 – 1.83 (m, 2H), 1.67 (brs, 3H), 1.57 (brs, 4H, overlap), 1.52 – 1.42 (m, 1H), 1.37 – 1.27 (m, 1H), 1.27 – 1.17 (m, 1H), 1.13 – 1.00 (m, 1H), 0.76 (d, J = 6.5 Hz, 3H).

**$^{13}$C NMR** (125 MHz, $CDCl_3$) δ: 140.17, 131.13, 128.93, 128.24, 126.83, 125.08, 58.42, 51.44, 37.26, 34.15, 30.52, 25.87, 25.61, 19.75, 17.79.

**HRMS** (ESI-TOF) ***m/z***: [M+H$^+$] calculated for $C_{24}H_{34}N$, 336.2691; found, 336.2695.

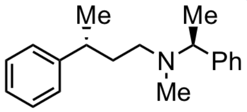
3g

**(R)-N-methyl-3-phenyl-N-((S)-1-phenylethyl)butan-1-amine (3g, Figure 3):** Prepared according to General procedure A from (***E***)-***N,N***-diethyl-3-phenylbut-2-en-1-amine (**1b**) with (***S***)-***N***-methyl-1-phenylethan-1-amine (**2g**) and (***R***)-BNIAP as ligand in 64% isolated yield.

**Column Chromatography Condition:** 100 g $Al_2O_3$ + 3 g $H_2O$, 50 : 1 hexanes/ EtOAc with 0.5% MeOH to 30 : 1 hexanes/ EtOAc with 1.0% MeOH as gradient eluent.

**$^1$H NMR** (500 MHz, $CDCl_3$) δδ 7.31 – 7.23 (m, 5H), 7.23 – 7.19 (m, 2H), 7.19 – 7.12 (m, 3H), 3.52 (q, J = 6.8 Hz, 1H), 2.70 (h, J = 7.1 Hz, 1H), 2.38 (ddd, J = 12.6, 9.4, 6.0 Hz, 1H), 2.18 (ddd, J = 12.5, 9.3, 5.3 Hz, 1H), 2.13 (s, 3H), 1.79 (dddd, J = 13.3, 9.4, 8.0, 5.3 Hz, 1H), 1.71 (ddt, J = 13.4, 9.3, 6.2 Hz, 1H), 1.30 (d, J = 6.8 Hz, 3H), 1.20 (d, J = 6.9 Hz, 3H).

**$^{13}$C NMR** (125 MHz, $CDCl_3$) δ: 147.74, 144.08, 128.42, 128.16, 127.82, 127.08, 126.77, 125.94, 63.22, 52.72, 38.47, 37.96, 35.76, 22.66, 18.24.

**HRMS** (ESI-TOF) ***m/z***: [M+H$^+$] calculated for $C_{19}H_{26}N$, 268.2065; found, 268.2073.

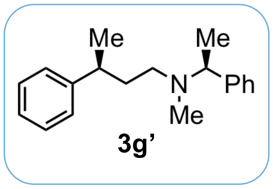


**(*S*)-*N*-methyl-3-phenyl-*N*-((*S*)-1-phenylethyl)butan-1-amine (3g', Figure 3):** Prepared according to General procedure A from (***E***)-***N,N***-diethyl-3-phenylbut-2-en-1-amine (**1b**) with (***S***)-***N***-methyl-1-phenylethan-1-amine (**2g**) and (***S***)-BNIAP as ligand in 60% isolated yield.

**Column Chromatography Condition:** 100 g $Al_2O_3$ + 3 g $H_2O$, 50 : 1 hexanes/ EtOAc with 0.5% MeOH to 30 : 1 hexanes/ EtOAc with 1.0% MeOH as gradient eluent.

**$^1$H NMR** (500 MHz, $CDCl_3$) δ: 7.30 – 7.26 (m, 3H), 7.25 – 7.18 (m, 4H), 7.17 – 7.11 (m, 3H), 3.49 (q, J = 6.7 Hz, 1H), 2.70 (h, J = 7.0 Hz, 1H), 2.36 – 2.20 (m, 2H), 2.12 (s, 3H), 1.81 – 1.67 (m, 2H), 1.26 (d, J = 6.8 Hz, 3H), 1.17 (d, J = 7.0 Hz, 3H).

**$^{13}$C NMR** (125 MHz, $CDCl_3$) δ: 147.77, 144.23, 128.41, 128.19, 127.80, 127.07, 126.79, 125.93, 63.34, 52.58, 38.60, 37.79, 35.65, 22.48, 18.55.

**HRMS** (ESI-TOF) ***m/z***: [M+H$^+$] calculated for $C_{19}H_{26}N$, 268.2065; found, 268.2066.

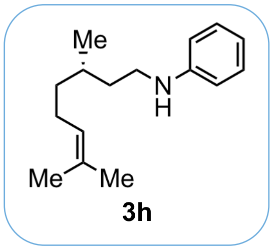


**(*S*)-*N*-(3,7-dimethyloct-6-en-1-yl)aniline (3h, Figure 3):** Prepared according to General procedure B from geranyl diethyl amine (**1a**) with aniline (**2h**) in 81% isolated yield.

**Column Chromatography Condition:** silica gel, 50 : 1 hexanes/ EtOAc as eluent.

**$^1$H NMR** (500 MHz, $CDCl_3$) δ: 7.22 – 7.11 (m, 2H), 6.75 – 6.66 (m, 1H), 6.64 – 6.58 (m, 2H), 5.11 (t, J = 7.0 Hz, 1H), 3.60 (brs, 1H), 3.26 – 3.00 (m, 2H), 2.12 – 1.91 (m, 2H), 1.70 (s, 3H), 1.68 – 1.63 (m, 1H), 1.61 (s, 3H), 1.59 – 1.54 (m, 1H), 1.49 – 1.34 (m, 2H), 1.28 – 1.17 (m, 1H), 0.95 (d, J = 6.6 Hz, 3H).

**$^{13}$C NMR** (125 MHz, $CDCl_3$) δ: 148.64, 131.49, 129.36, 124.79, 117.27, 112.87, 42.12, 37.24, 36.84, 30.58, 25.88, 25.62, 19.75, 17.83.

**HRMS** (ESI-TOF) ***m/z***: [M+H$^+$] calculated for $C_{16}H_{26}N$, 232.2065; found, 232.2064.

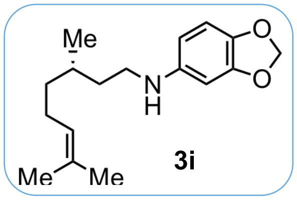


**(*S*)-*N*-(3,7-dimethyloct-6-en-1-yl)benzo[d][1,3]dioxol-5-amine (3i, Figure 3):** Prepared according to General procedure B from geranyl diethyl amine (**1a**) with benzo[***d***] [1,3]dioxol-5-amine aniline (**2i**) in 74% isolated yield.

**Column Chromatography Condition:** silica gel, 30 : 1 hexanes/ EtOAc as eluent.

**$^1$H NMR** (500 MHz, $CDCl_3$) δ: 6.65 (d, J = 8.2 Hz, 1H), 6.24 (d, J = 2.3 Hz, 1H), 6.04 (dd, J = 8.3, 2.3 Hz, 1H), 5.85 (s, 2H), 5.12 (t, J = 7.0 Hz, 1H), 3.35 (brs, 1H), 3.15 – 2.91 (m, 2H), 2.13 – 1.88 (m, 2H), 1.69 (s, 3H), 1.66 – 1.62 (m, 1H), 1.61 (s, 3H), 1.57 – 1.50 (m, 1H), 1.46 – 1.31 (m, 2H), 1.28 – 1.14 (m, 1H), 0.94 (d, J = 6.5 Hz, 3H).

**$^{13}$C NMR** (125 MHz, $CDCl_3$) δ: 148.46, 144.52, 139.55, 131.49, 124.78, 108.75, 104.44, 100.65, 96.00, 43.15, 37.24, 36.85, 30.58, 25.88, 25.61, 19.75, 17.83.

**HRMS** (ESI-TOF) ***m/z***: [M+H$^+$] calculated for $C_{17}H_{26}NO_2$, 276.1964; found, 276.1961.

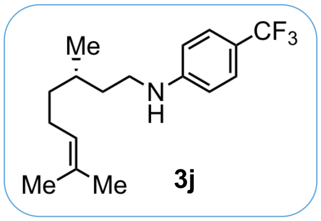

**(*S*)-*N*-(3,7-dimethyloct-6-en-1-yl)-4-(trifluoromethyl)aniline (3j, Figure 3):** Prepared according to General procedure B from geranyl diethyl amine (**1a**) with 4-trifluoromethyl aniline (**2j**) in 61% isolated yield (as a mixture of 12:1 desired product and hydrogenated product).

**Column Chromatography Condition:** silica gel, 99 : 1 hexanes/ EtOAc as eluent.

**$^{1}$H NMR** (500 MHz, $CDCl_3$) δ: δ 7.32 (d, J = 8.6 Hz, 2H), 6.51 (d, J = 8.4 Hz, 2H), 5.05 – 4.99 (m, 1H), 3.87 (brs, 1H), 3.21 – 2.84 (m, 2H), 2.06 – 1.80 (m, 2H), 1.62 (d, J = 1.3 Hz, 3H), 1.60 – 1.55 (m, 1H), 1.53 (d, J = 1.4 Hz, 3H), 1.51 – 1.45 (m, 1H), 1.44 – 1.35 (m, 1H), 1.34 – 1.25 (m, 1H), 1.17 – 1.10 (m, 1H), 0.88 (d, J = 6.6 Hz, 3H)..

**$^{13}$C NMR** (125 MHz, $CDCl_3$) δ: 151.04, 131.72, 126.82 (q, J = 3.8 Hz), 125.28 (q, J = 270.2 Hz), 124.75, 118.72 (q, J = 32.7 Hz), 111.92, 41.76, 37.26, 36.63, 30.59, 25.98, 25.69, 19.80, 17.93.

**$^{19}$F NMR** (471 MHz, $CDCl_3$) δ: -61.30.

**HRMS** (ESI-TOF) ***m/z***: [M+H$^+$] calculated for $C_{17}H_{25}NF_3$, 300.1939; found, 300.1947.

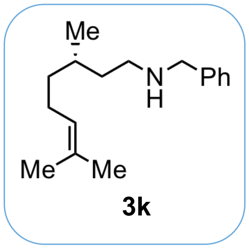

**(*S*)-*N*-benzyl-3,7-dimethyloct-6-en-1-amine (3k, Figure 3):** Prepared according to General procedure C from geranyl diethyl amine (**1a**) with benzylamine (**2k**) in 70% isolated yield.

**Column Chromatography Condition:** 100 g $Al_2O_3$ + 9 g $H_2O$, 20 : 1 hexanes/ EtOAc with 0.5% MeOH to 10 : 1 hexanes/ EtOAc with 1.0% MeOH as gradient eluent.

**$^{1}$H NMR** (500 MHz, $CDCl_3$) δ: 7.34 – 7.30 (m, 4H), 7.26 – 7.21 (m, 1H), 5.09 (dddd, J = 7.1, 5.7, 2.9, 1.4 Hz, 1H), 3.79 (s, 2H), 2.72 – 2.58 (m, 2H), 2.06 – 1.88 (m, 2H), 1.68 (d, J = 1.3 Hz, 3H), 1.59 (s, 3H), 1.56 – 1.44 (m, 2H), 1.39 – 1.28 (m, 2H), 1.21 – 1.10 (m, 1H), 0.88 (d, J = 6.5 Hz, 3H).

**$^{13}$C NMR** (125 MHz, $CDCl_3$) δ: 140.70, 131.31, 128.51, 128.25, 127.00, 124.97, 54.35, 47.60, 37.43, 37.38, 30.77, 25.87, 25.64, 19.78, 17.80.

**HRMS** (ESI-TOF) ***m/z***: [M+H$^+$] calculated for $C_{17}H_{28}N$, 246.2222; found, 246.2228.

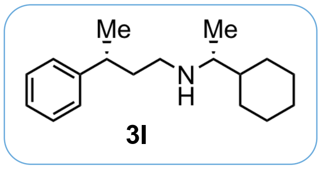

**(*R*)-*N*-((*R*)-1-cyclohexylethyl)-3-phenylbutan-1-amine (3l, Figure 3):** Prepared according to General procedure C from (***E***)-***N,N***-diethyl-3-phenylbut-2-en-1-amine (**1b**) with (***R***)-1-cyclohexylethan-1-amine (**2l**) in 61% isolated yield.

**Column Chromatography Condition:** 100 g $Al_2O_3$ + 9 g $H_2O$, 30 : 1 hexanes/ EtOAc with 0.5% MeOH to 15 : 1 hexanes/ EtOAc with 1.0% MeOH as gradient eluent.

**$^{1}$H NMR** (500 MHz, $CDCl_3$) δ: δ 7.32 – 7.27 (m, 2H), 7.22 – 7.15 (m, 3H), 2.77 (h, J = 7.1 Hz, 1H), 2.63 – 2.53 (m, 1H), 2.42 – 2.29 (m, 2H), 1.80 – 1.68 (m, 4H), 1.67 – 1.57 (m, 3H), 1.25 (d, J = 6.9 Hz, 4H, overlap), 1.20 – 1.04 (m, 4H), 0.99 – 0.92 (m, 1H), 0.91 (d, J = 6.4 Hz, 3H).

**$^{13}$C NMR** (125 MHz, $CDCl_3$) δ: 147.49, 128.49, 127.07, 126.06, 57.93, 46.03, 43.12, 39.00, 38.35, 30.07, 28.09, 26.92, 26.80, 26.66, 22.74, 16.87.

**HRMS** (ESI-TOF) ***m/z***: [M+H⁺] calculated for $C_{18}H_{30}N$, 260.2378; found, 260.2381.

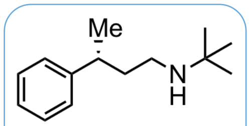

**3m**

**(*R*)-*N*-(tert-butyl)-3-phenylbutan-1-amine (3m, Figure 3):** Prepared according to General procedure B from (***E***)-***N,N***-diethyl-3-phenylbut-2-en-1-amine (**1b**) with t-butylamine (**2m**) in 58% isolated yield.

**Column Chromatography Condition:** silical gel, 30 : 1 hexanes/ EtOAc to 10 : 1 hexanes/ EtOAc as gradient eluent.

**¹H NMR** (500 MHz, $CDCl_3$) δ: 7.39 – 7.30 (m, 3H), 7.26 – 7.19 (m, 2H), 2.84 (h, J = 7.0 Hz, 1H), 2.62 – 2.43 (m, 2H), 1.87 – 1.75 (m, 2H), 1.31 (d, J = 6.9 Hz, 3H), 1.08 (s, 9H).

**¹³C NMR** (125 MHz, $CDCl_3$) δ: 147.38, 128.47, 127.07, 126.06, 50.34, 40.91, 39.60, 38.34, 29.16, 22.80.

**HRMS** (ESI-TOF) ***m/z***: [M+H⁺] calculated for $C_{14}H_{24}N$, 206.1909; found, 206.1913.

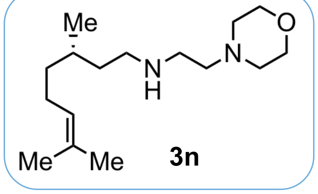

**3n**

**(*S*)-3,7-dimethyl-N-(2-morpholinoethyl)oct-6-en-1-amine (3n, Figure 3):** Prepared according to General procedure C from geranyl diethyl amine (**1a**) with 2-morpholinoethan-1-amine (**2n**) in 66% isolated yield.

**Purification:** No column chromatography needed. Reaction crude was concentrated to remove solvent then re-dissolve in $Et_2O$ followed by an acid/base extraction to afford the desired product **3n**.

**¹H NMR** (500 MHz, $CDCl_3$) δ: δ 5.09 (ddt, J = 8.9, 7.2, 1.6 Hz, 1H), 3.93 – 3.48 (m, 4H), 2.71 (t, J = 6.2 Hz, 2H), 2.62 (dddd, J = 20.9, 11.4, 10.4, 5.7 Hz, 2H), 2.49 (t, J = 6.1 Hz, 2H), 2.45 – 2.40 (m, 4H), 1.96 (m, 2H), 1.81 (brs, 1H), 1.67 (d, J = 1.6 Hz, 3H), 1.59 (s, 3H), 1.57 – 1.44 (m, 2H), 1.40 – 1.28 (m, 2H), 1.18 – 1.11 (m, 1H), 0.88 (d, J = 6.4 Hz, 3H).

**¹³C NMR** (125 MHz, $CDCl_3$) δ: 131.32, 124.92, 67.18, 58.42, 53.91, 48.14, 46.35, 37.36, 37.32, 30.80, 25.86, 25.64, 19.74, 17.79.

**HRMS** (ESI-TOF) ***m/z***: [M+H⁺] calculated for $C_{16}H_{33}N_2O$, 269.2593; found, 269.2593.

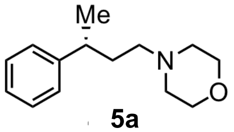

**5a**

**(*R*)-4-(3-phenylbutyl)morpholine (5a, Figure 4):** Prepared according to General procedure A from (***E***)-***N,N***-diethyl-3-phenylbut-2-en-1-amine (**4a**) with morpholine (**2a**) in 77% isolated yield.

**Column Chromatography Condition:** 100 g $Al_2O_3$ + 9 g $H_2O$, 30 : 1 hexanes/ EtOAc with 0.5% MeOH to 15 : 1 hexanes/ EtOAc with 1.0% MeOH as gradient eluent.

**¹H NMR** (500 MHz, $CDCl_3$) δ: 7.35 – 7.27 (m, 2H), 7.22 – 7.15 (m, 3H), 3.69 (t, J = 4.7 Hz, 4H), 2.75 (h, J = 7.1 Hz, 1H), 2.47 – 2.33 (m, 4H), 2.27 (ddd, J = 12.1, 8.5, 6.5 Hz, 1H), 2.19 (ddd, J = 12.1, 8.6, 6.6 Hz, 1H), 1.83 – 1.73 (m, 2H), 1.26 (d, J = 6.9 Hz, 3H).

**¹³C NMR** (125 MHz, $CDCl_3$) δ: 147.30, 128.51, 127.08, 126.13, 67.18, 57.45, 53.94, 38.24, 35.18, 22.64.

**HRMS** (ESI-TOF) ***m/z***: [M+H⁺] calculated for $C_{14}H_{22}NO$, 220.1701; found, 220.1706.

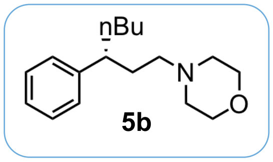


**(*R*)-4-(3-phenylheptyl)morpholine (5b, Figure 4):** Prepared according to General procedure A from (***E***)-***N,N***-diethyl-3-phenylhept-2-en-1-amine (**4b**) with morpholine (**2a**) in 86% isolated yield.

**Column Chromatography Condition:** 100 g $Al_2O_3$ + 9 g $H_2O$, 30 : 1 hexanes/ EtOAc with 0.5% MeOH to 15 : 1 hexanes/ EtOAc with 1.0% MeOH as gradient eluent.

**$^1$H NMR** (500 MHz, $CDCl_3$) δ: 7.31 – 7.25 (m, 2H), 7.21 – 7.16 (m, 1H), 7.15 – 7.10 (m, 2H), 3.68 (t, J = 4.7 Hz, 4H), 2.53 (tt, J = 9.7, 5.4 Hz, 1H), 2.43 – 2.29 (m, 4H), 2.21 (ddd, J = 12.1, 10.2, 5.8 Hz, 1H), 2.10 (ddd, J = 12.1, 10.2, 4.9 Hz, 1H), 1.85 (ddt, J = 13.1, 10.5, 5.4 Hz, 1H), 1.76 – 1.68 (m, 1H), 1.67 – 1.50 (m, 2H), 1.38 – 1.00 (m, 4H), 0.82 (t, J = 7.2 Hz, 3H).

**$^{13}$C NMR** (125 MHz, $CDCl_3$) δ: 145.76, 128.41, 127.73, 126.08, 67.17, 57.46, 53.94, 44.27, 36.96, 33.75, 29.89, 22.88, 14.15.

**HRMS** (ESI-TOF) ***m/z***: [M+H$^+$] calculated for $C_{17}H_{28}NO$, 262.2171; found, 262.2177.

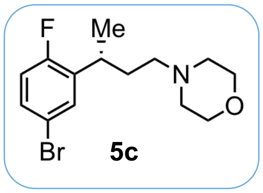


**(*R*)-4-(3-(5-bromo-2-fluorophenyl)butyl)morpholine (5c, Figure 4):** Prepared according to General procedure A from (***E***)-3-(5-bromo-2-fluorophenyl)-***N,N***-diethylbut-2-en-1-amine (**4c**) with morpholine (**2a**) in 74% isolated yield.

**Column Chromatography Condition:** 100 g $Al_2O_3$ + 9 g $H_2O$, 50 : 1 hexanes/ EtOAc with 0.5% MeOH to 15 : 1 hexanes/ EtOAc with 1.0% MeOH as gradient eluent.

**$^1$H NMR** (500 MHz, $CDCl_3$) δ: 7.32 (dd, J = 6.5, 2.5 Hz, 1H), 7.29 – 7.22 (m, 1H), 6.88 (dd, J = 9.9, 8.6 Hz, 1H), 3.68 (t, J = 4.7 Hz, 4H), 3.08 (h, J = 7.0 Hz, 1H), 2.44 – 2.34 (m, 4H), 2.29 (ddd, J = 12.3, 9.2, 6.0 Hz, 1H), 2.22 (ddd, J = 12.2, 9.3, 5.9 Hz, 1H), 1.86 – 1.68 (m, 2H), 1.25 (d, J = 6.9 Hz, 3H).

**$^{13}$C NMR** (125 MHz, $CDCl_3$) δ: 159.90 (d, J = 245.2 Hz), 136.23 (d, J = 16.3 Hz), 131.19 (d, J = 5.4 Hz), 130.34 (d, J = 8.4 Hz), 117.31 (d, J = 24.8 Hz), 116.79 (d, J = 3.2 Hz), 67.11, 57.06, 53.87, 33.80, 31.15, 31.14, 20.93.

**HRMS** (ESI-TOF) ***m/z***: [M+H$^+$] calculated for $C_{14}H_{20}NOBrF$, 316.0712; found, 316.0716.

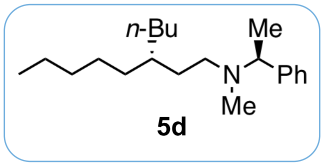


**(*S*)-3-butyl-N-methyl-N-((S)-1-phenylethyl)octan-1-amine (5d, Figure 4):** Prepared according to General procedure A from (***E***)-3-butyl-***N,N***-diethyloct-2-en-1-amine (**4d**) with (***S***)-***N***-methyl-1-phenylethan-1-amine (**2g**) in 61% isolated yield. $[\alpha]_D^{23}$ = -21.09 (*c* = 1.05)

**Column Chromatography Condition:** 100 g $Al_2O_3$ + 3 g $H_2O$, 50 : 1 hexanes/ EtOAc with 0.5% MeOH as eluent.

**$^1$H NMR** (500 MHz, $CDCl_3$) δ: 7.32 – 7.29 (m, 4H), 7.24 – 7.20 (m, 1H), 3.55 (q, J = 6.7 Hz, 1H), 2.40 (ddd, J = 12.5, 9.8, 6.0 Hz, 1H), 2.29 – 2.20 (m, 1H), 2.18 (s, 3H), 1.45 – 1.38 (m, 2H), 1.36 (d, J = 6.8 Hz, 3H), 1.32 – 1.22 (m, 6H), 1.22 – 1.10 (m, 9H), 0.87 (t, J = 7.1 Hz, 6H).

**$^{13}$C NMR** (125 MHz, $CDCl_3$) δ: 144.35, 128.19, 127.82, 126.81, 63.55, 52.39, 38.79, 35.89, 33.82, 33.60, 32.47, 31.03, 28.96, 26.41, 23.25, 22.85, 18.91, 14.29, 14.28.

**HRMS** (ESI-TOF) ***m/z***: [M+H+] calculated for $C_{21}H_{38}N$, 304.3004; found, 304.3006.

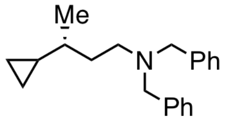

5e

**(*R*)-*N,N*-dibenzyl-3-cyclopropylbutan-1-amine (5e, Figure 4):** Prepared according to General procedure A from (***E***)-3-cyclopropyl-***N,N***-diethylbut-2-en-1-amine (**4e**) with dibenzylamine (**2f**) in 69% isolated yield.

**Column Chromatography Condition:** silica gel, 30 : 1 hexanes/ EtOAc as eluent.

**$^1$H NMR** (500 MHz, $CDCl_3$) δ: 7.37 (d, J = 7.1 Hz, 4H), 7.31 (dd, J = 8.4, 6.7 Hz, 4H), 7.25 – 7.19 (m, 2H), 3.62 (d, J = 13.6 Hz, 2H), 3.51 (d, J = 13.6 Hz, 2H), 2.54 (ddd, J = 12.8, 9.0, 6.4 Hz, 1H), 2.46 (ddd, J = 12.7, 9.1, 5.1 Hz, 1H), 1.74 (ddt, J = 12.7, 9.2, 6.0 Hz, 1H), 1.54 – 1.39 (m, 1H), 0.84 (d, J = 6.6 Hz, 3H), 0.79 – 0.64 (m, 1H), 0.50 – 0.38 (m, 1H), 0.36 – 0.28 (m, 2H), 0.02 – -0.05 (m, 2H).

**$^{13}$C NMR** (125 MHz, $CDCl_3$) δ: 140.14, 128.98, 128.23, 126.83, 58.36, 51.49, 36.71, 34.59, 19.89, 18.35, 4.49, 3.23.

**HRMS** (ESI-TOF) ***m/z***: [M+H+] calculated for $C_{21}H_{28}N$, 294.2222; found, 294.2220.

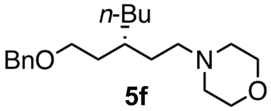

5f

**(*R*)-4-(3-(2-(benzyloxy)ethyl)heptyl)morpholine (5f, Figure 4):** Prepared according to General procedure A from (***E***)-3-(2-(benzyloxy)ethyl)-***N,N***-diethylhept-2-en-1-amine (**4f**) with morpholine (**2a**) in 66% isolated yield.

**Column Chromatography Condition:** 100 g $Al_2O_3$ + 6 g $H_2O$, 15 : 1 hexanes/ EtOAc with 0.5% MeOH as eluent.

**$^1$H NMR** (500 MHz, $CDCl_3$) δ: 7.35 – 7.31 (m, 4H), 7.30 – 7.26 (m, 1H), 4.49 (s, 2H), 3.70 (t, J = 4.7 Hz, 4H), 3.49 (t, J = 6.9 Hz, 2H), 2.47 – 2.38 (m, 4H), 2.35 – 2.28 (m, 2H), 1.60 (qd, J = 6.8, 1.4 Hz, 2H), 1.53 – 1.49 (m, 1H), 1.48 – 1.42 (m, 2H), 1.29 – 1.23 (m, 8H), 0.88 (t, J = 6.8 Hz, 3H).

**$^{13}$C NMR** (125 MHz, $CDCl_3$) δ: 138.75, 128.48, 127.76, 127.64, 73.07, 68.70, 67.15, 57.10, 54.06, 33.93, 33.66, 33.34, 30.59, 28.84, 23.17, 14.25.

**HRMS** (ESI-TOF) ***m/z***: [M+H+] calculated for $C_{20}H_{34}NO_2$, 320.2590; found, 320.2598.

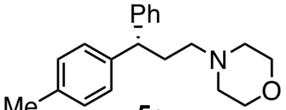

5g

**(*S*)-4-(3-phenyl-3-(p-tolyl)propyl)morpholine (5g, Figure 4):** Prepared according to General procedure A from (***E***)-***N,N***-diethyl-3-phenyl-3-(p-tolyl)prop-2-en-1-amine (**4g**) with morpholine (**2a**) in 66% isolated yield.

**Column Chromatography Condition:** 100 g $Al_2O_3$ + 9 g $H_2O$, 30 : 1 hexanes/ EtOAc with 0.5% MeOH to 15 : 1 hexanes/ EtOAc with 1.0% MeOH as gradient eluent.

**$^1$H NMR** (500 MHz, $CDCl_3$) δ: 7.31 – 7.26 (m, 3H), 7.25 – 7.24 (m, 1H), 7.21 – 7.14 (m, 3H), 7.12 – 7.08 (m, 2H), 3.98 (t, J = 7.4 Hz, 1H), 3.77 – 3.68 (m, 4H), 2.46 – 2.37 (m, 4H), 2.32 (s, 3H), 2.30 – 2.27 (m, 2H), 2.27 – 2.20 (m, 2H).

**$^{13}$C NMR** (125 MHz, $CDCl_3$) δ: 145.19, 141.90, 135.81, 129.29, 128.57, 127.90, 127.81, 126.23, 67.19, 57.46, 53.94, 48.74, 32.61, 21.12.

**HRMS** (ESI-TOF) ***m/z***: [M+H$^+$] calculated for $C_{20}H_{26}NO$, 296.2014; found, 296.2006.

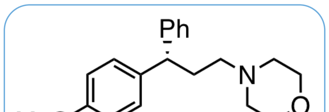

**5h**

**(*S*)-4-(3-(4-methoxyphenyl)-3-phenylpropyl)morpholine (5h, Figure 4):** Prepared according to General procedure A from (***E***)-***N,N***-diethyl-3-(4-methoxyphenyl)-3-phenylprop-2-en-1-amine (**4h**) with morpholine (**2a**) in 81% isolated yield.

**Column Chromatography Condition:** 100 g $Al_2O_3$ + 8 g $H_2O$, 10 : 1 hexanes/ EtOAc with 1.0% MeOH as eluent.

**$^{1}$H NMR** (500 MHz, $CDCl_3$) δ: 7.29 – 7.26 (m, 1H), 7.25 – 7.21 (m, 3H), 7.20 – 7.13 (m, 3H), 6.89 – 6.76 (m, 2H), 3.95 (t, J = 7.6 Hz, 1H), 3.77 (s, 3H), 3.71 (t, J = 4.7 Hz, 5H), 2.49 – 2.36 (m, 4H), 2.31 – 2.24 (m, 2H), 2.24 – 2.15 (m, 2H).

**$^{13}$C NMR** (125 MHz, $CDCl_3$) δ: 158.06, 145.32, 137.04, 128.85, 128.57, 127.85, 126.22, 113.96, 67.18, 57.45, 55.35, 53.94, 48.27, 32.74.

**HRMS** (ESI-TOF) ***m/z***: [M+H$^+$] calculated for $C_{20}H_{26}NO_2$, 312.1964; found, 312.1960.

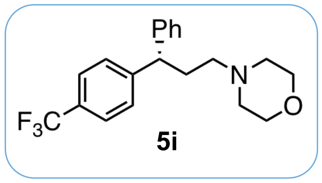

**5i**

**(*S*)-4-(3-phenyl-3-(4-(trifluoromethyl)phenyl)propyl)morpholine (5i, Figure 4):** Prepared according to General procedure A from (***E***)-***N,N***-diethyl-3-phenyl-3-(4-(trifluoromethyl)phenyl)prop-2-en-1-amine (**4i**) with morpholine (**2a**) in 78% isolated yield.

**Column Chromatography Condition:** 100 g $Al_2O_3$ + 9 g $H_2O$, 30 : 1 hexanes/ EtOAc with 0.5% MeOH to 15 : 1 hexanes/ EtOAc with 1.0% MeOH as gradient eluent.

**$^{1}$H NMR** (500 MHz, $CDCl_3$) δ: 7.53 (d, J = 7.9 Hz, 2H), 7.36 (d, J = 8.0 Hz, 2H), 7.33 – 7.27 (m, 2H), 7.25 – 7.18 (m, 3H), 4.17 – 4.03 (m, 1H), 3.71 (t, J = 4.7 Hz, 4H), 2.50 – 2.34 (m, 4H), 2.32 – 2.16 (m, 4H).

**$^{13}$C NMR** (125 MHz, $CDCl_3$) δ: 149.05, 143.86, 128.80, 128.65 (q, J = 32.4 Hz), 128.31, 127.94, 126.74, 125.56 (q, J = 3.8 Hz), 124.36 (q, J = 271.8 Hz), 67.16, 57.02, 53.90, 48.81, 32.34, 29.85.

**$^{19}$F NMR** (471 MHz, $CDCl_3$) δ: -62.75.

**HRMS** (ESI-TOF) ***m/z***: [M+H$^+$] calculated for $C_{20}H_{23}NOF_3$, 350.1732; found, 350.1729.

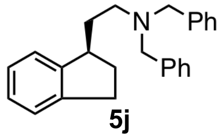

**5j**

**(*R*)-*N,N*-dibenzyl-2-(2,3-dihydro-1H-inden-1-yl)ethan-1-amine (5j, Figure 4):** Prepared according to General procedure A from (***E***)-2-(2,3-dihydro-1H-inden-1-ylidene)-***N,N***-diethylethan-1-amine (**4j**) with dibenzylamine (**2f**) in 69% isolated yield.

**Column Chromatography Condition:** silica gel, 99 : 1 hexanes/ EtOAc as eluent.

**$^{1}$H NMR** (500 MHz, $CDCl_3$) δ: 7.42 – 7.36 (m, 4H), 7.32 (t, J = 7.5 Hz, 4H), 7.24 (t, J = 7.4 Hz, 2H), 7.21 – 7.17 (m, 1H), 7.15 – 7.08 (m, 2H), 7.07 – 7.03 (m, 1H), 3.69 (d, J = 13.5 Hz, 2H), 3.52 (d, J = 13.6 Hz, 2H), 3.16 (ddd, J = 12.0, 9.9, 6.0

Hz, 1H), 2.84 (ddd, J = 15.8, 8.6, 4.5 Hz, 1H), 2.75 (dt, J = 15.9, 8.1 Hz, 1H), 2.61 (dd, J = 12.9, 7.6 Hz, 1H), 2.55 (ddt, J = 13.0, 8.4, 4.4 Hz, 1H), 2.16 – 2.07 (m, 1H), 2.03 (dtt, J = 12.4, 7.9, 4.1 Hz, 1H), 1.61 – 1.55 (m, 1H), 1.47 (dq, J = 12.4, 8.0 Hz, 1H).

**$^{13}$C NMR** (125 MHz, $CDCl_3$) δ: 147.77, 144.07, 140.03, 129.06, 128.33, 126.97, 126.32, 126.14, 124.50, 123.59, 58.58, 51.70, 42.74, 32.64, 32.22, 31.52.

**HRMS** (ESI-TOF) ***m/z***: [M+H$^+$] calculated for $C_{25}H_{28}N$, 342.2222; found, 342.2221.

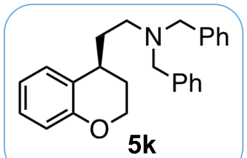


5k

**(*S*)-*N,N*-dibenzyl-2-(chroman-4-yl)ethan-1-amine (5k, Figure 4):** Prepared according to General procedure A from (***E***)-2-(chroman-4-ylidene)-***N,N***-diethylethan-1-amine (**4k**) with dibenzylamine (**2f**) in 77% isolated yield.

**Column Chromatography Condition:** silica gel, 30 : 1 hexanes/ EtOAc as eluent.

**$^1$H NMR** (500 MHz, $CDCl_3$) δ: 7.39 (d, J = 7.1 Hz, 4H), 7.33 (dd, J = 8.4, 6.7 Hz, 4H), 7.28 – 7.23 (m, 2H), 7.11 – 7.03 (m, 1H), 7.02 – 6.94 (m, 1H), 6.83 – 6.74 (m, 2H), 4.05 (dd, J = 6.5, 4.3 Hz, 2H), 3.74 (d, J = 13.5 Hz, 2H), 3.48 (d, J = 13.5 Hz, 2H), 2.94 (dq, J = 9.9, 5.0 Hz, 1H), 2.61 (dt, J = 12.9, 7.6 Hz, 1H), 2.51 (ddd, J = 12.7, 7.3, 4.7 Hz, 1H), 2.06 (dtd, J = 14.0, 7.7, 4.1 Hz, 1H), 1.84 – 1.70 (m, 1H), 1.62 (dddd, J = 14.2, 10.0, 7.0, 4.6 Hz, 1H), 1.51 – 1.40 (m, 1H).

**$^{13}$C NMR** (125 MHz, $CDCl_3$) δ: 154.64, 139.89, 129.12, 129.08, 128.38, 127.24, 127.07, 126.96, 120.24, 116.83, 63.54, 58.75, 50.54, 34.08, 31.06, 26.58.

**HRMS** (ESI-TOF) ***m/z***: [M+H$^+$] calculated for $C_{25}H_{28}NO$, 358.2171; found, 358.2171.

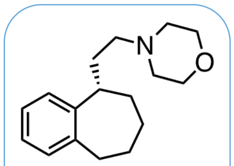


5l

**(*S*)-4-(2-(6,7,8,9-tetrahydro-5H-benzo[7]annulen-5-yl)ethyl)morpholine (5l, Figure 3):** Prepared according to General procedure A from (***Z***)-***N,N***-diethyl-2-(6,7,8,9-tetrahydro-5H-benzo[7]annulen-5-ylidene)ethan-1-amine (**4l**) with morpholine (**2a**) in 75% isolated yield.

**Column Chromatography Condition:** 100 g $Al_2O_3$ + 6 g $H_2O$, 30 : 1 hexanes/ EtOAc with 0.5% MeOH to 15 : 1 hexanes/ EtOAc with 1.0% MeOH as gradient eluent.

**$^1$H NMR** (500 MHz, Benzene-d$_6$) δ: 7.13 (dd, J = 7.5, 1.8 Hz, 1H), 7.10 (td, J = 7.2, 1.8 Hz, 1H), 7.06 (td, J = 7.1, 1.8 Hz, 1H), 7.03 (dd, J = 7.4, 1.8 Hz, 1H), 3.61 (t, J = 4.8 Hz, 4H), 2.90 (qd, J = 7.1, 2.3 Hz, 1H), 2.82 – 2.73 (m, 1H), 2.71 – 2.64 (m, 1H), 2.19 – 2.11 (m, 6H), 1.91 (dq, J = 13.8, 7.4 Hz, 1H), 1.79 – 1.44 (m, 7H).

**$^{13}$C NMR** (125 MHz, $CDCl_3$) δ: 145.14, 142.59, 130.03, 128.03, 126.06, 126.04, 67.15, 57.99, 54.02, 43.20, 36.26, 33.40, 29.85, 29.72, 28.22.

**HRMS** (ESI-TOF) ***m/z***: [M+H$^+$] calculated for $C_{17}H_{26}NO$, 260.2014; found, 260.2017.

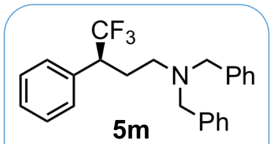

5m

**(*S*)-*N,N*-dibenzyl-4,4,4-trifluoro-3-phenylbutan-1-amine (5m, Figure 4):** Prepared according to General procedure A from (***E***)-***N,N***-diethyl-4,4,4-trifluoro-3-phenylbut-2-en-1-amine (**4m**) with dibenzylamine (**2f**) in 63% isolated yield.

**Column Chromatography Condition:** silica gel, 50 : 1 hexanes/ EtOAc as eluent.

**$^1$H NMR** (500 MHz, $CDCl_3$) δ: 7.34 – 7.27 (m, 9H), 7.25 – 7.18 (m, 4H), 7.01 (dd, J = 7.5, 1.8 Hz, 2H), 3.67 (d, J = 13.5 Hz, 2H), 3.43 – 3.34 (m, 1H), 3.32 (d, J = 13.5 Hz, 2H), 2.37 (ddd, J = 12.1, 8.6, 6.1 Hz, 1H), 2.33 – 2.27 (m, 1H), 2.27 – 2.19 (m, 1H), 2.02 – 1.88 (m, 1H).

**$^{13}$C NMR** (125 MHz, $CDCl_3$) δ: 139.41, 134.82 (q, J = 1.9 Hz), 129.15, 129.13, 128.60, 128.39, 127.97, 127.31 (q, J= 279.5 Hz), 127.09, 58.46, 50.19, 47.49 (q, J = 26.6 Hz), 26.95 (q, J = 1.7 Hz).

**$^{19}$F NMR** (471 MHz, $CDCl_3$) δ: -69.58 (d, J = 9.8 Hz).

**HRMS** (ESI-TOF) ***m/z***: [M+H$^+$] calculated for $C_{24}H_{25}NF_3$, 384.1939; found, 384.1927.

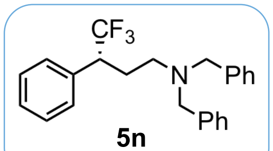

5n

**(*R*)-*N,N*-dibenzyl-4,4,4-trifluoro-3-phenylbutan-1-amine (5n, Figure 3):** Prepared according to General procedure A from (***Z***)-***N,N***-diethyl-4,4,4-trifluoro-3-phenylbut-2-en-1-amine (**4n**) with dibenzylamine (**2f**) in 71% isolated yield.

**Column Chromatography Condition:** silica gel, 50 : 1 hexanes/ EtOAc as eluent.

**$^1$H NMR** (500 MHz, $CDCl_3$) δ: δ 7.35 – 7.24 (m, 9H), 7.24 – 7.17 (m, 4H), 7.08 – 6.95 (m, 2H), 3.67 (d, J = 13.4 Hz, 2H), 3.44 – 3.34 (m, 1H), 3.32 (d, J = 13.5 Hz, 2H), 2.43 – 2.33 (m, 1H), 2.32 – 2.20 (m, 2H), 1.94 (dtd, J = 15.2, 6.8, 3.3 Hz, 1H).

**$^{13}$C NMR** (125 MHz, $CDCl_3$) δ: 139.41, 134.82 (q, J = 1.8 Hz), 129.15, 129.13, 128.60, 128.39, 127.97, 127.31 (q, J= 279.0 Hz), 127.09, 58.47, 50.19, 47.49 (q, J = 26.6 Hz), 26.96 (q, J = 1.8 Hz).

**$^{19}$F NMR** (471 MHz, $CDCl_3$) δ: -69.58 (d, J = 9.8 Hz).

**HRMS** (ESI-TOF) ***m/z***: [M+H$^+$] calculated for $C_{24}H_{25}NF_3$, 384.1939; found, 384.1952.

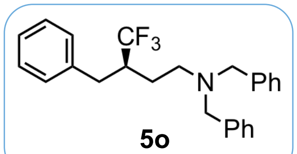

5o

**(*R*)-N,N,3-tribenzyl-4,4,4-trifluorobutan-1-amine (5o, Figure 4):** Prepared according to General procedure A from (***E***)-3-benzyl-***N,N***-diethyl-4,4,4-trifluorobut-2-en-1-amine (**4o**) with dibenzylamine (**2f**) in 59% isolated yield.

**Column Chromatography Condition:** silica gel, 50 : 1 hexanes/ EtOAc as eluent.

**$^1$H NMR** (500 MHz, $CDCl_3$) δ: 7.30 (dd, J = 8.1, 6.8 Hz, 4H), 7.26 – 7.15 (m, 9H), 7.04 – 6.99 (m, 2H), 3.54 – 3.46 (m, 2H), 3.38 (d, J = 13.6 Hz, 2H), 2.94 – 2.81 (m, 1H), 2.54 – 2.45 (m, 2H), 2.45 – 2.34 (m, 2H), 1.82 (dtd, J = 14.5, 7.3, 4.9 Hz, 1H), 1.60 (ddt, J = 13.6, 7.7, 5.6 Hz, 1H).

**$^{13}$C NMR** (125 MHz, $CDCl_3$) δ: 139.40, 138.26, 129.28, 129.02, 128.58, 128.44 (q, *J* = 280.4 Hz), 128.33, 127.05, 126.61, 58.14, 50.53, 42.39 (q, J = 24.8 Hz), 34.40 (q, J = 2.9 Hz), 24.96 (q, J = 1.8 Hz).

**$^{19}$F NMR** (471 MHz, $CDCl_3$) δ: -70.26 (d, J = 8.3 Hz).

**HRMS** (ESI-TOF) ***m/z***: [M+H$^+$] calculated for $C_{25}H_{27}NF_3$, 398.2096; found, 398.2090.

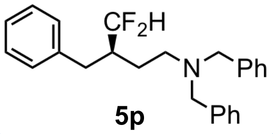


5p

**(*R*)-*N,N*,3-tribenzyl-4,4-difluorobutan-1-amine (5p, Figure 4):** Prepared according to General procedure A (***E***)-3-benzyl-***N,N***-diethyl-4,4-difluorobut-2-en-1-amine (**4p**) with dibenzyl-amine (**2f**) in 70% isolated yield.

**Column Chromatography Condition:** silica gel, 30 : 1 hexanes/ EtOAc as eluent.

**$^1$H NMR** (500 MHz, $CDCl_3$) δ: 7.34 – 7.28 (m, 7H), 7.26 – 7.22 (m, 4H), 7.21 – 7.16 (m, 1H), 7.08 – 7.02 (m, 2H), 5.54 (td, J = 56.7, 2.9 Hz, 1H), 3.57 – 3.48 (m, 2H), 3.44 (d, J = 13.4 Hz, 2H), 2.67 (dd, J = 13.9, 6.9 Hz, 1H), 2.49 – 2.47 (m, 1H), 2.45 (t, J = 6.8 Hz, 2H), 2.33 – 2.11 (m, 1H), 1.83 – 1.67 (m, 1H), 1.53 – 1.46 (m, 1H).

**$^{13}$C NMR** (125 MHz, $CDCl_3$) δ: 139.60, 139.00, 129.28, 129.11, 128.60, 128.37, 127.09, 126.42, 117.96 (t, J = 241.7 Hz), 58.37, 50.35, 41.73 (t, J = 19.1 Hz), 33.89 (dd, J = 6.2, 3.6 Hz), 24.20 (t, J = 3.9 Hz).

**$^{19}$F NMR** (471 MHz, $CDCl_3$) δ: -124.91 (ddd, J = 277.9, 56.8, 15.6 Hz), -126.24 (ddd, J = 277.8, 56.7, 17.6 Hz).

**HRMS** (ESI-TOF) ***m/z***: [M+H$^+$] calculated for $C_{25}H_{28}NF_2$, 380.2190; found, 380.2182.

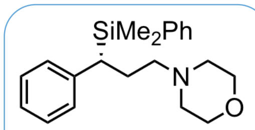


5q

**(*R*)-4-(3-(dimethyl(phenyl)silyl)-3-phenylpropyl)morpholine (5q, Figure 4):** Prepared according to General procedure A from (***Z***)-3-(dimethyl(phenyl)silyl)-***N,N***-diethyl-3-phenylprop-2-en-1-amine (**4q**) with morpholine (**2a**) in 75% isolated yield.

**Column Chromatography Condition:** 100 g $Al_2O_3$ + 9 g $H_2O$, 30 : 1 hexanes/ EtOAc with 0.5% MeOH to 15 : 1 hexanes/ EtOAc with 1.0% MeOH as gradient eluent.

**$^1$H NMR** (500 MHz, $CDCl_3$) δ: 7.42 – 7.36 (m, 2H), 7.39 – 7.28 (m, 3H), 7.23 – 7.15 (m, 2H), 7.12 – 7.05 (m, 1H), 6.98 – 6.91 (m, 2H), 3.65 (t, J = 4.7 Hz, 4H), 2.32 – 2.23 (m, 5H), 2.20 (ddd, J = 12.1, 7.9, 6.0 Hz, 1H), 2.11 (dt, J = 12.1, 7.7 Hz, 1H), 1.90 (dt, J = 8.1, 7.0 Hz, 2H), 0.25 (s, 3H), 0.16 (s, 3H).

**$^{13}$C NMR** (125 MHz, $CDCl_3$) δ: 142.71, 137.57, 134.24, 129.19, 128.20, 128.00, 127.75, 124.75, 67.11, 58.83, 53.86, 34.51, 26.45, -3.73, -5.29.

**HRMS** (ESI-TOF) ***m/z***: [M+H$^+$] calculated for $C_{21}H_{30}NOBSi$, 340.2097; found, 340.2091.

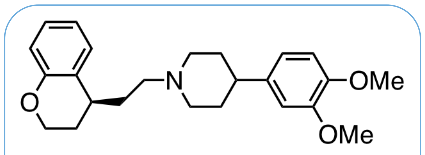


*Terikalant*

***(S)*-1-(2-(chroman-4-yl)ethyl)-4-(3,4-dimethoxyphenyl)piperidine (Terikanlant, Scheme 2):** Prepared according to General procedure A from (***E***)-2-(chroman-4-ylidene)-***N,N***-diethylethan-1-amine (**4k**) with 4-(3,4-dimethoxyphenyl) piperidine[7] in 75% isolated yield.

**Column Chromatography Condition:** 100 g $Al_2O_3$ + 6 g $H_2O$, 12 : 1 hexanes/ EtOAc with 0.5% MeOH to 6 : 1 hexanes/ EtOAc with 1.0% MeOH as gradient eluent.

**$^1$H NMR** (500 MHz, $CDCl_3$) δ: 7.20 (d, J = 7.6 Hz, 1H), 7.13 (ddd, J = 8.7, 7.4, 1.7 Hz, 1H), 6.90 (td, J = 7.4, 1.3 Hz, 1H), 6.87 – 6.80 (m, 4H), 4.31 – 4.19 (m, 2H), 3.91 (s, 3H), 3.90 (s, 3H), 3.23 – 3.06 (m, 2H), 2.94 (dq, J = 10.2, 5.3 Hz, 1H), 2.55 (t, J = 7.7 Hz, 2H), 2.49 (dt, J = 11.7, 4.2 Hz, 1H), 2.21 – 2.03 (m, 4H), 1.95 – 1.75 (m, 6H).

**$^{13}$C NMR** (125 MHz, $CDCl_3$) δ: 154.61, 148.92, 147.42, 139.23, 129.22, 127.47, 126.34, 120.27, 118.66, 116.95, 111.25, 110.24, 63.62, 56.71, 56.03, 55.91, 54.85, 54.43, 42.50, 33.91, 33.89, 33.85, 32.19, 27.24.

**HRMS** (ESI-TOF) ***m/z***: [M+H$^+$] calculated for $C_{24}H_{32}NO_3$, 382.2382; found, 382.2375.

**Enantioselective Synthesis of (*R*)-Tolterodine**

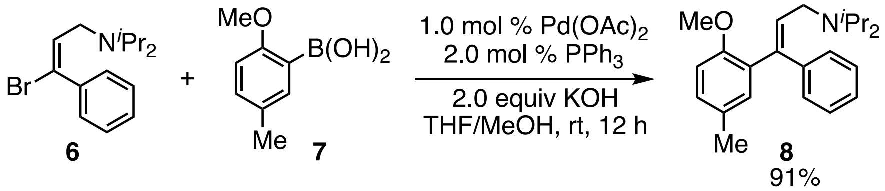


**Vinyl bromide 6** was prepared from ***trans***-cinnamyl chloride according to literature.[2]

**Suzuki coupling**: To a oven-dried 100 ml round bottom flask was charged with a stir bar, purged with $N_2$ three times then added 11 mg $Pd(OAc)_2$ (0.050 mmol, 1.0 mol %), 26 mg $PPh_3$ (0.10 mmol, 2.0 mol %), 0.560 g KOH (10 mmol, 2.0 equiv), starting material vinyl bromide (1.48g, 5 mmol, 1.0 equiv) ,0.996 g (2-methoxy-5-methylphenyl)boronic acid **7**(6.5 mmol, 1.3 equiv) and 20 mL THF and 20 mL MeOH. The reaction was stirred at rt overnight followed by dilution with EtOAc, and washed by 1 N NaOH solution and brine. **Acid-base extraction**: the organic layer was concentrated ***in vacuo,*** re-dissolved in $Et_2O$, and extracted with 3 N HCl solution three times. The resulting acidic aqueous layer was then basified by the addition of 5N NaOH solution until the pH > 11, followed by the extraction with DCM. The combined organic layers was then dried over $MgSO_4$, concentrated ***in vacuo***, purified by **$Al_2O_3$ column chromatography**: 200 g $Al_2O_3$ + 8 g $H_2O$, 50 : 1 hexanes/ EtOAc with 0.5% MeOH as eluent to afford allylic amine **8** in 91% isolated yield. For **1n**: **$^1$H NMR** (500 MHz, $CDCl_3$) δ: 7.30 – 7.26 (m, 2H), 7.24 – 7.19 (m, 1H), 7.18 – 7.14 (m, 2H), 7.06 – 7.00 (m, 2H), 6.72 (d, J = 8.0 Hz, 1H), 5.89 (t, J = 6.4 Hz, 1H), 3.51 (s, 3H), 3.28 (d, J = 6.4 Hz, 2H), 3.06 (p, J = 6.5 Hz, 2H), 2.29 (t, J = 0.8 Hz, 3H), 0.96 (d, J = 6.6 Hz, 12H). **$^{13}$C NMR** (125 MHz, $CDCl_3$) δ: 155.24, 141.08, 138.59, 134.18, 133.49, 131.64, 129.79, 129.20, 128.69, 127.55, 126.42, 111.95, 55.99, 48.96, 43.99, 20.96, 20.62. The geometry of double bond was confirmed by NOE experiment (See **Supplementary Figure 64** for details).

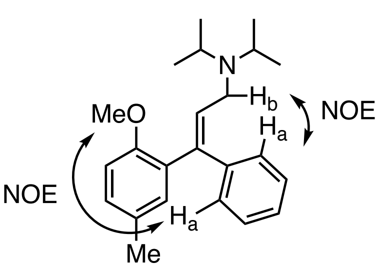


8

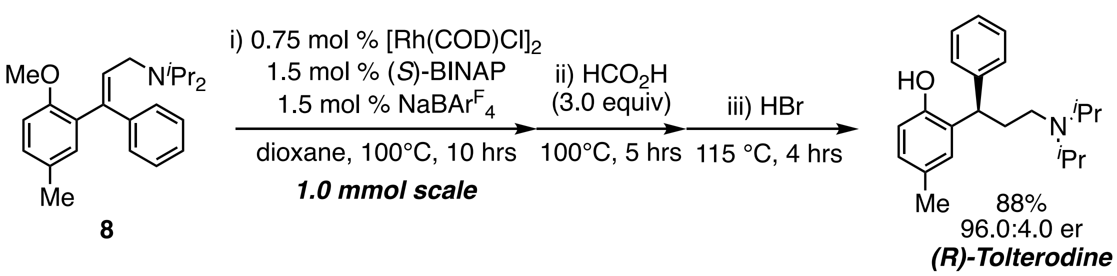


**Tolterodine synthesis**: $[Rh(COD)Cl]_2$ (4.0 mg, 0.75 mol %), (***S***)-BINAP (9.6 mg, 1.5 mol %), $NaBAr_4^F$ (12.8 mg, 1.5 mol %), and 1,4-dioxane (0.8 mL) were added to a 20 mL vial equipped with a stir bar in the glove box under nitrogen atmosphere. To the vial was added allylic diisopropylamine (**8**, 1.0 mmol, 1.0 equiv). The resulting solution was allowed to stir for 10 h at 100 °C. After 10 h, formic acid (3.0 mmol, 3.0 equiv) was added into reaction vial via syringe and the reaction was allowed to stir for another 5 h at 100 °C. The reaction crude was then diluted in DCM, filtered through basic alumina, and concentrated ***in vacuo*** (to get rid of 1,4-dioxane solvent). The residue was then transferred into another 20 mL vial, followed by the addition of HBr solution (2.2 mL, 13.2 equiv) and HOAc (2.0 mL), and allowed to stir at 115 °C for 4 h. After 4 h, the reaction crude was then diluted in water, extracted with EtOAc three times. Combined organic layers were washed with 1 N NaOH solution three times. The pH of last basic wash was verified to be >10. The organic layer was washed with brine, dried over $MgSO_4$, concentrated ***in vacuo*** and then purified by basic alumina chromatography to afford the desired product **(*R*)-Tolterodine** in 88% isolated yield.

**Column Chromatography Condition:** 100 g $Al_2O_3$ + 5 g $H_2O$, 15 : 1 hexanes/ EtOAc with 0.5% MeOH to 8 : 1 hexanes/ EtOAc with 1.0% MeOH as gradient eluent.

**$^1$H NMR** (500 MHz, $CDCl_3$) δ: 10.33 (brs, 1H), 7.33 (d, J = 4.3 Hz, 4H), 7.23 (h, J = 4.3 Hz, 1H), 6.85 (dd, J = 8.2, 2.1 Hz, 1H), 6.80 (d, J = 8.1 Hz, 1H), 6.53 (d, J = 2.5 Hz, 1H), 4.49 (dd, J = 11.3, 4.0 Hz, 1H), 3.23 (p, J = 6.7 Hz, 2H), 2.73 (dt, J = 12.7, 3.6 Hz, 1H), 2.50 – 2.25 (m, 2H), 2.12 (s, 3H), 2.10 – 2.03 (m, 1H), 1.13 (d, J = 6.7 Hz, 6H), 1.08 (d, J = 6.6 Hz, 6H).

**$^{13}$C NMR** (125 MHz, $CDCl_3$) δ: 153.34, 144.88, 132.55, 129.53, 128.78, 128.66, 128.42, 127.88, 126.28, 118.32, 48.03, 42.21, 39.46, 33.37, 20.91, 20.10, 19.69.

**HRMS** (ESI-TOF) ***m/z***: [M+H$^+$] calculated for $C_{22}H_{32}NO$, 326.2484; found, 326.2489.

**Determination of enantiomeric ratio of product 3m.**

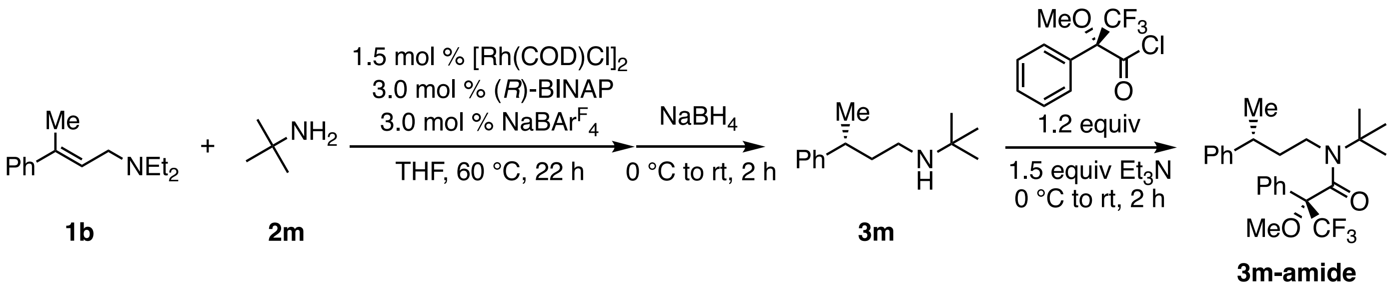


**Procedure:** Product 3bm was prepared according to the General Procedure B, followed by the amidation with (***R***)-3,3,3-trifluoro-2-methoxy-2-phenylpropanoyl chloride to afford the desired amide product **3m-amide**. The corresponding **rac-3m-amide** was prepared using same method but with (±)-BINAP as ligand.

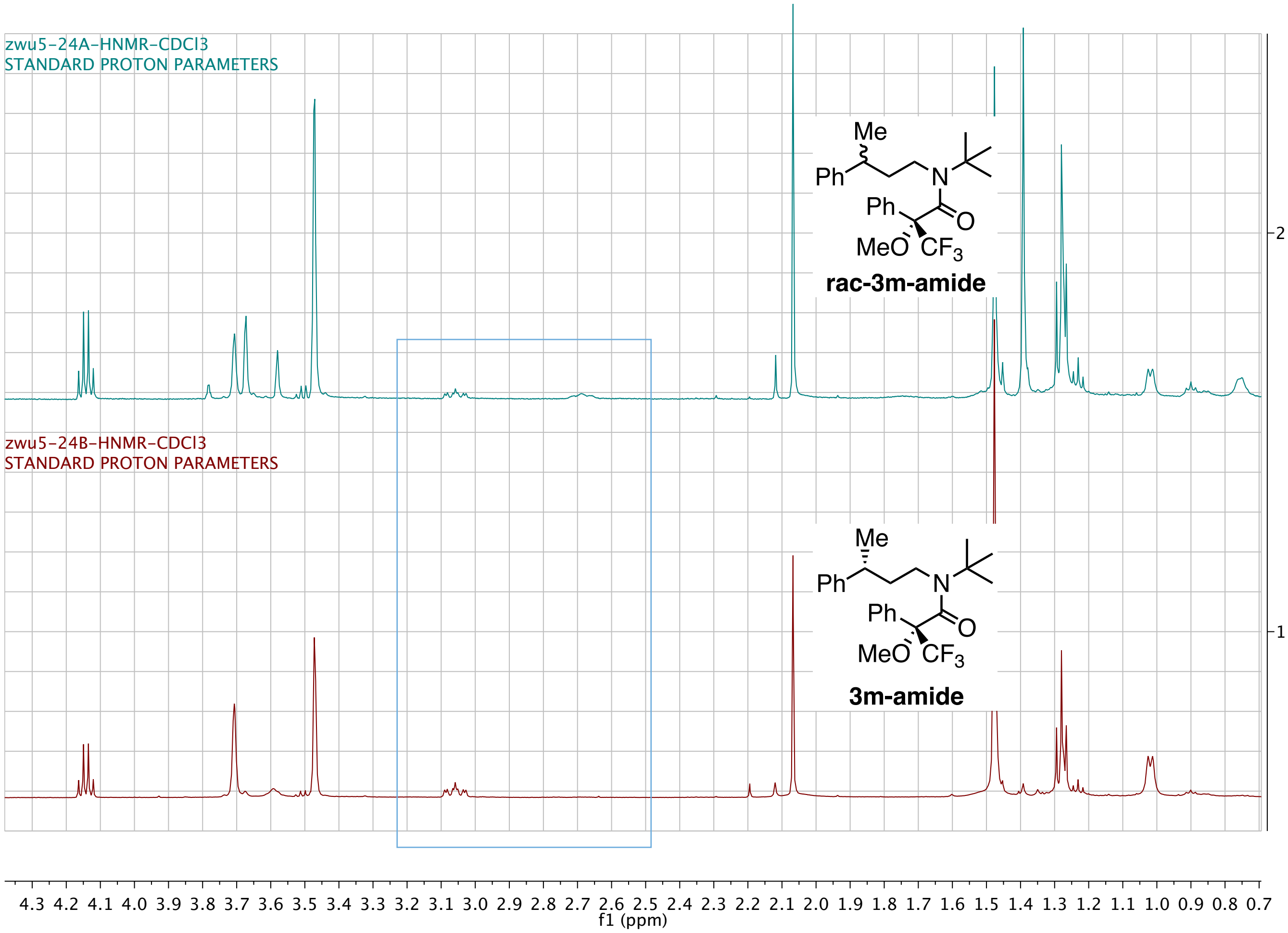


**Supplementary Figure 1.** Crude $^{1}$H NMR of **rac-3m-amide** and **3m-amide**

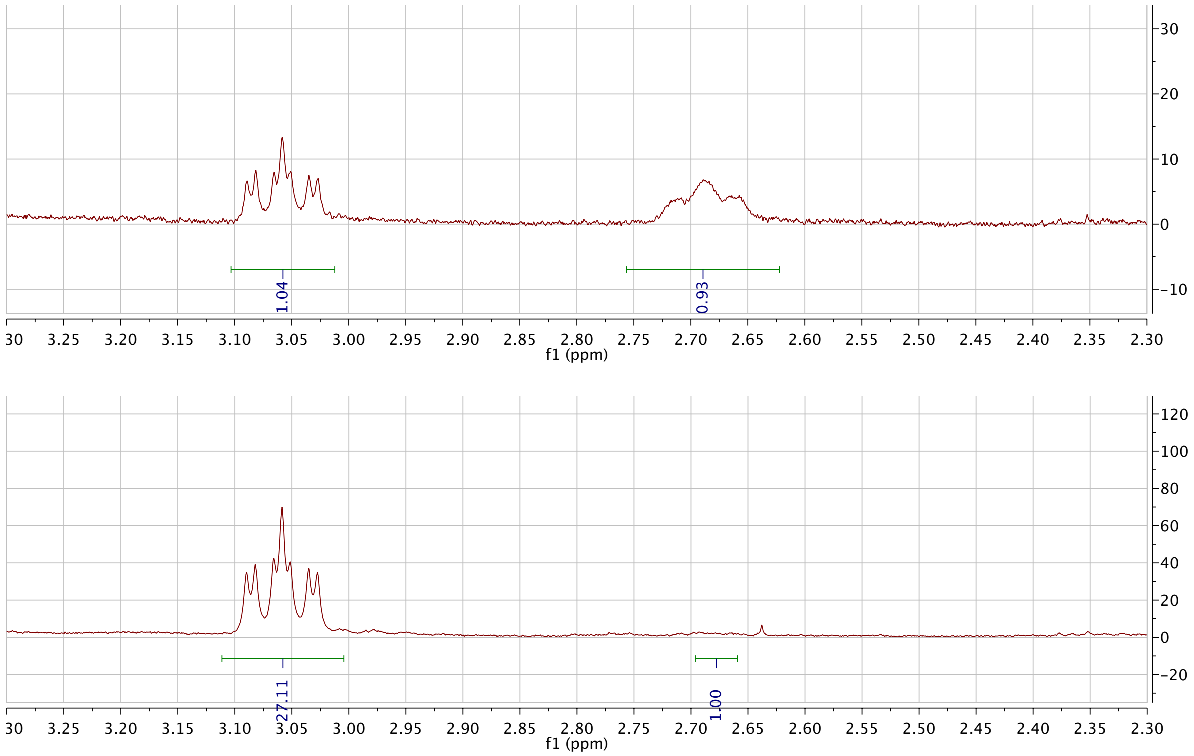


**Supplementary Figure 2.** Zoom-in of Supplementary Figure 1

for **3m-amide** dr=27.11:1, therefore, the er of 3bm = 96.5 : 3.5. $[\alpha]_D^{23}$ = -16.29 ($c$ = 0.81).

## Determination of enantiomeric ratio of product 3n.

**3n-amide** and **rac-3n-amide** were prepared as described above.

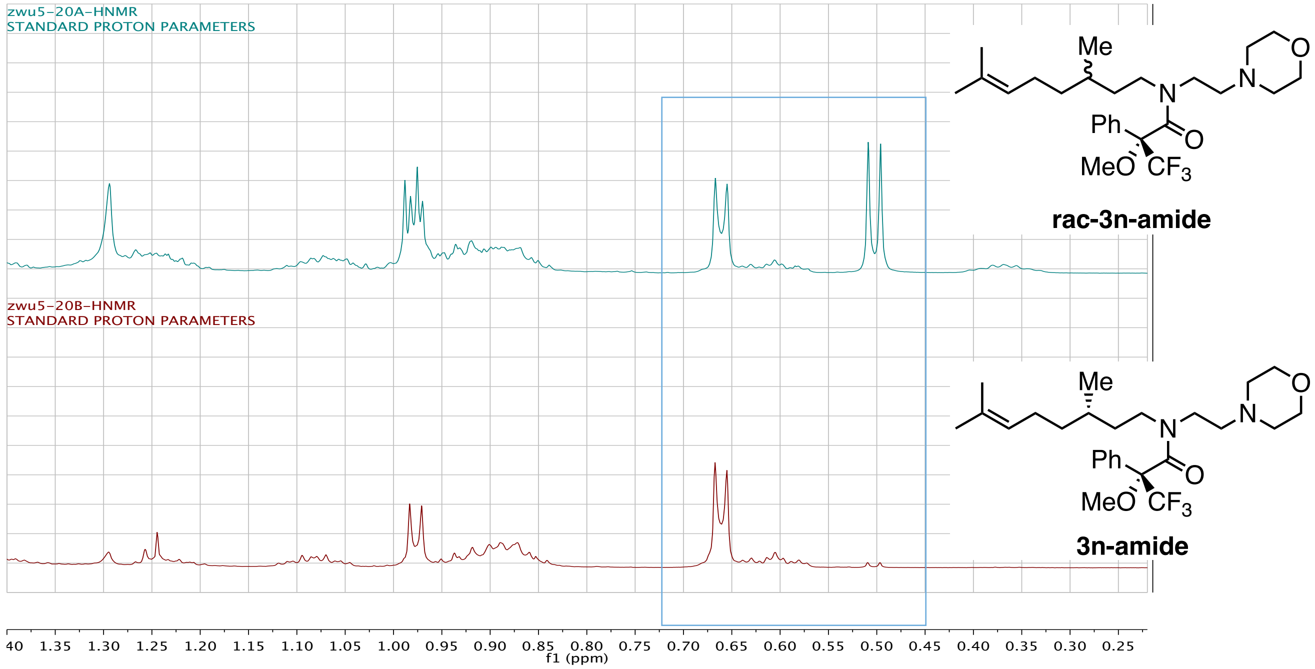


**Supplementary Figure 3.** Crude $^1$H NMR of **rac-3n-amide** and **3n-amide**

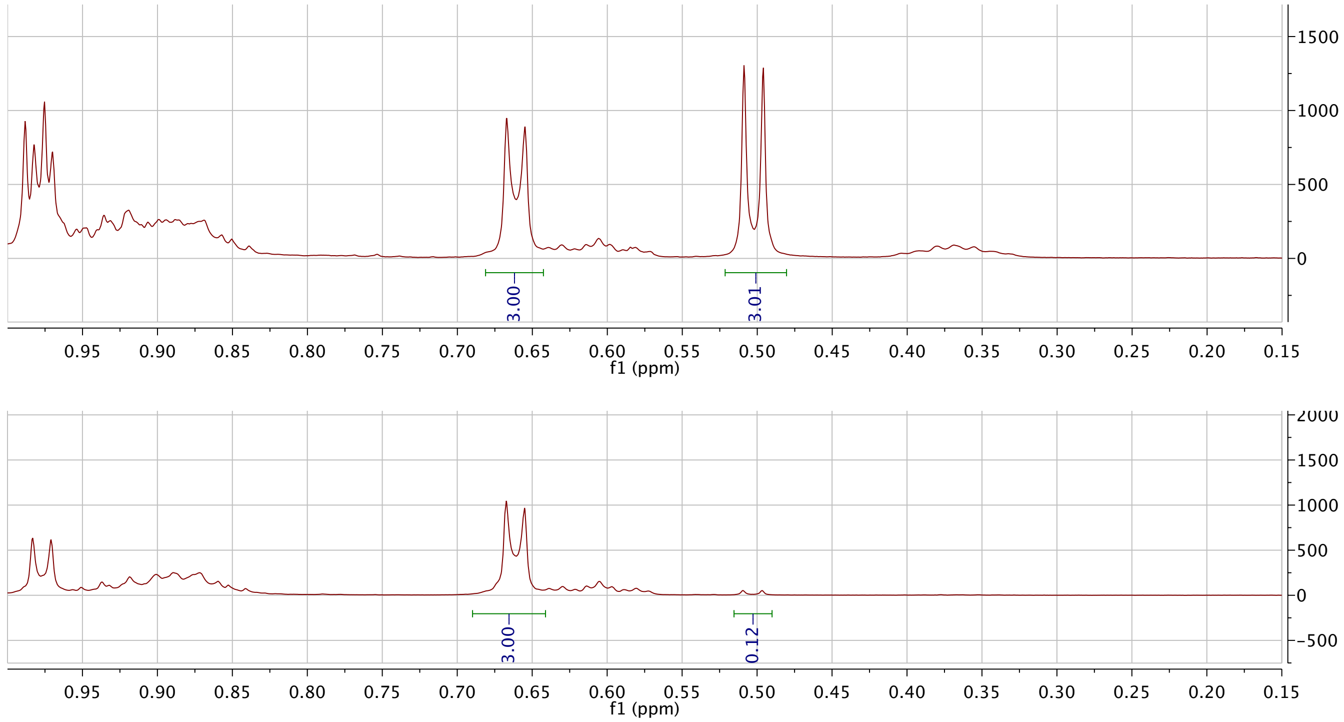

**Supplementary Figure 4.** Zoom-in of Supplementary Figure 3

for **3n-amide** dr=25.0:1, therefore, the er of 3bm = 96.2 : 3.8. $[\alpha]_D^{23}$ = +2.39 ($c$ = 1.98)

## Control Experiment of Enamine Reduction

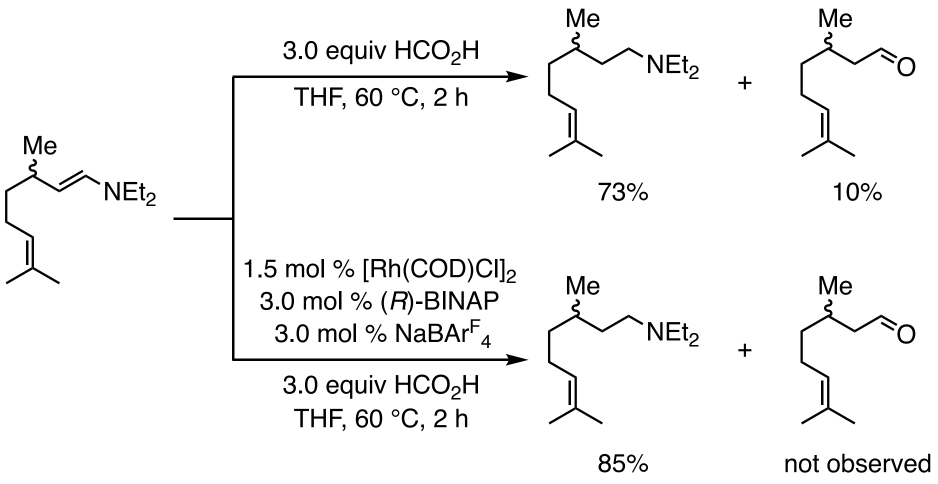


**Supplementary Figure 5.** Control experiments

**Procedure:** A pre-made geranyl diethyl enamine was subjected to reduction conditions with and without the rhodium catalyst as shown above. After 2 hours, the reaction crude was concentrated under vacuum, and analyzed using NMR spectroscopy in $CDCl_3$.

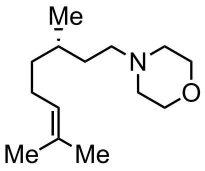


**3a**

25% (95% hexanes, 5% EtOH, 0.2%TFA, 0.1%DEA), 75% hexanes, 1.0 mL/min, CHIRALPAK® IA3

er = 96.2:3.8

$[\alpha]_D^{23}$ = +5.26 ($c$ = 1.03)

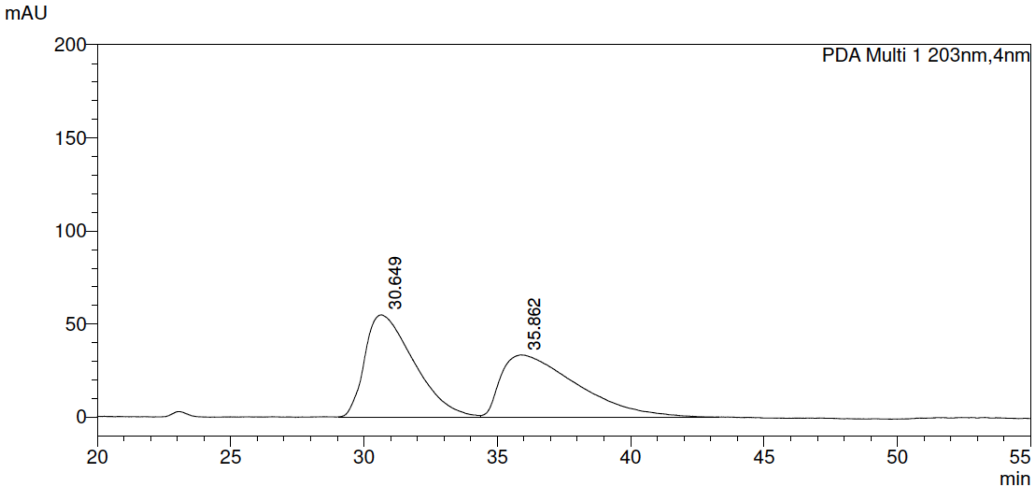


PDA Ch1 203nm

| Peak# | Ret. Time | Area | Height | Area% |
|---|---|---|---|---|
| 1 | 30.649 | 7045334 | 54875 | 51.550 |
| 2 | 35.862 | 6621600 | 33379 | 48.450 |
| Total | | 13666934 | 88254 | 100.000 |

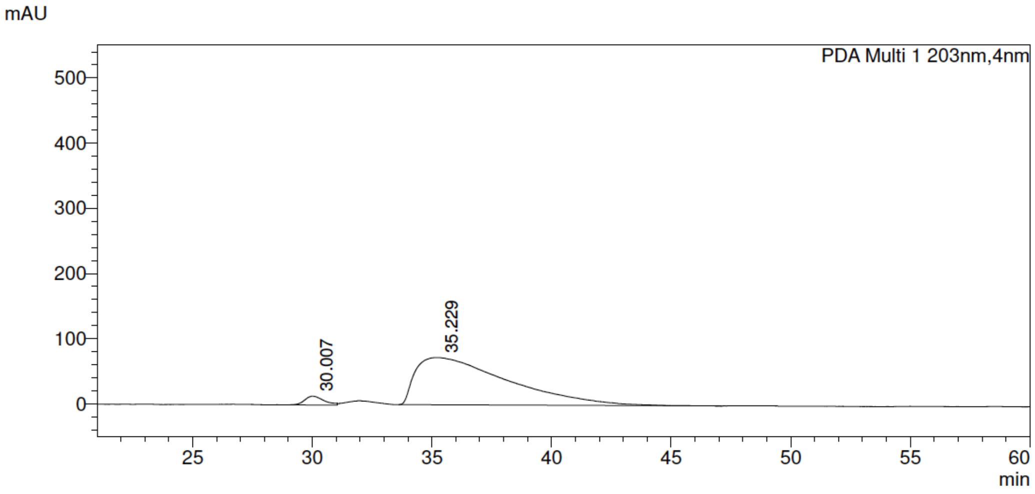


PDA Ch1 203nm

| Peak# | Ret. Time | Area | Height | Conc. | Area% |
|---|---|---|---|---|---|
| 1 | 30.007 | 760599 | 13701 | 0.000 | 3.751 |
| 2 | 35.229 | 19514962 | 72457 | 0.000 | 96.249 |
| Total | | 20275561 | 86158 | | 100.000 |

**Supplementary Figure 6.** HPLC spectra for racemic and chiral **3a**

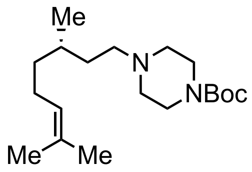


**3b**

10% (95% hexanes, 5% EtOH, 0.2%TFA, 0.1%DEA), 90% hexanes, 0.8 mL/min, CHIRALCEL® OJ-H

er = 95.5:4.5

$[\alpha]_D^{23} = +0.96$ ($c$ = 1.06)

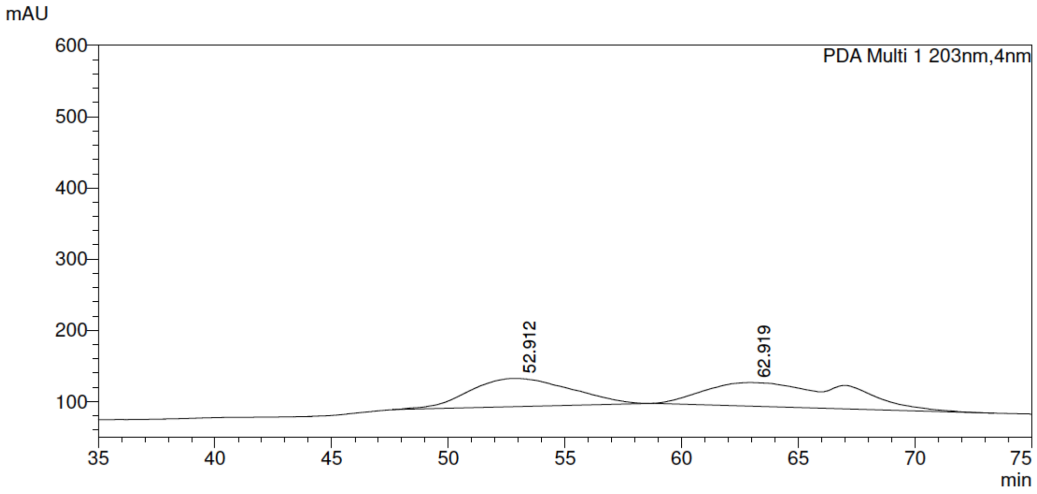


PDA Ch1 203nm

| Peak# | Ret. Time | Area | Height | Area% |
|---|---|---|---|---|
| 1 | 52.912 | 12004307 | 39668 | 44.341 |
| 2 | 62.919 | 15068254 | 33358 | 55.659 |
| Total | | 27072561 | 73026 | 100.000 |

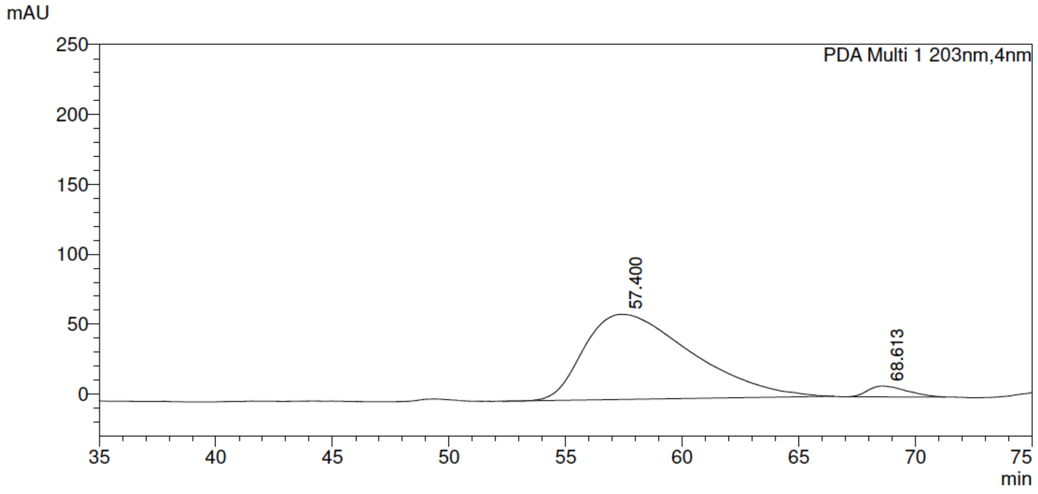


PDA Ch1 203nm

| Peak# | Ret. Time | Area | Height | Area% |
|---|---|---|---|---|
| 1 | 57.400 | 19593618 | 60894 | 95.537 |
| 2 | 68.613 | 915277 | 7851 | 4.463 |
| Total | | 20508895 | 68745 | 100.000 |

**Supplementary Figure 7.** HPLC spectra for racemic and chiral **3b**

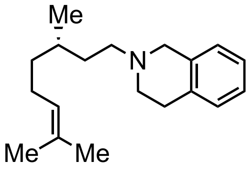


**3c**

15% (95% hexanes, 5% EtOH, 0.2%TFA, 0.1%DEA), 85% hexanes, 0.5 mL/min, CHIRALPAK® IA3

er = 97.9:2.1

$[\alpha]_D^{23}$ = +4.99 ($c$ = 1.22)

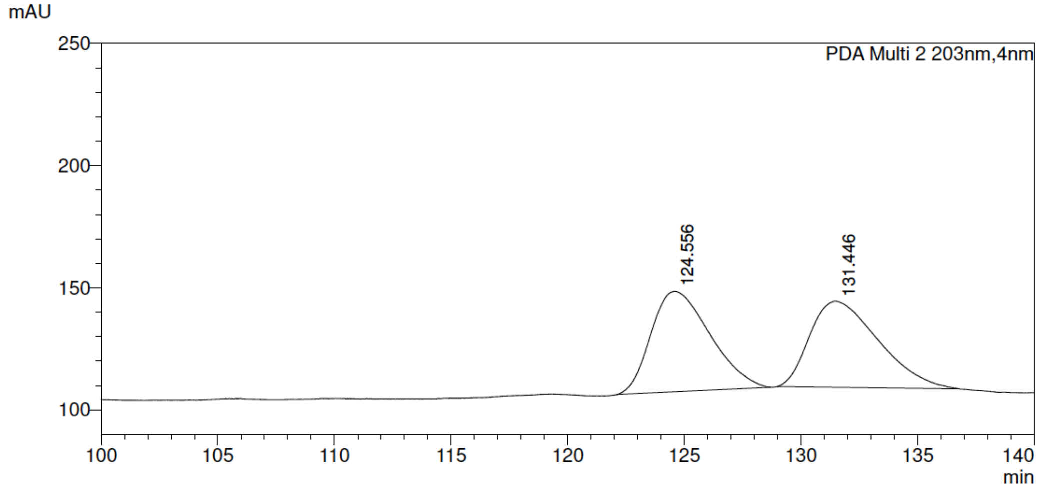


PDA Ch2 203nm

| Peak# | Ret. Time | Area | Height | Area% |
|---|---|---|---|---|
| 1 | 124.556 | 7224948 | 41143 | 50.072 |
| 2 | 131.446 | 7204093 | 35251 | 49.928 |
| Total | | 14429041 | 76394 | 100.000 |

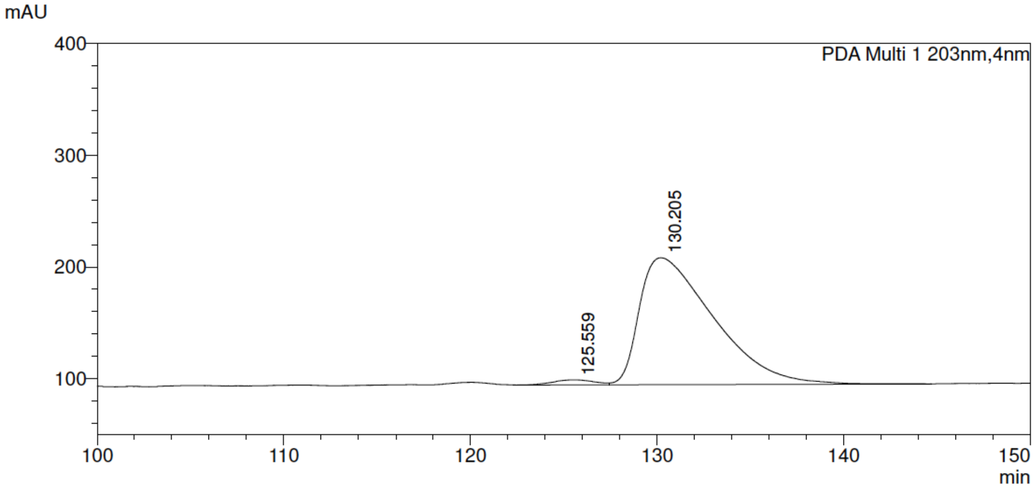


PDA Ch1 203nm

| Peak# | Ret. Time | Area | Height | Area% |
|---|---|---|---|---|
| 1 | 125.559 | 683713 | 4457 | 2.133 |
| 2 | 130.205 | 31377640 | 113789 | 97.867 |
| Total | | 32061352 | 118247 | 100.000 |

**Supplementary Figure 7.** HPLC spectra for racemic and chiral **3c**

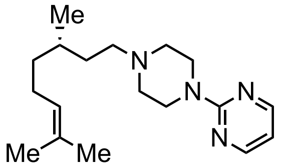


**3d**

35% (95% hexanes, 5% EtOH, 0.2%TFA, 0.1%DEA), 65% hexanes, 0.8 mL/min, CHIRALCEL® OJ-H

er = 97.1:2.9

$[\alpha]_D^{23} = +6.54$ ($c$ = 1.24)

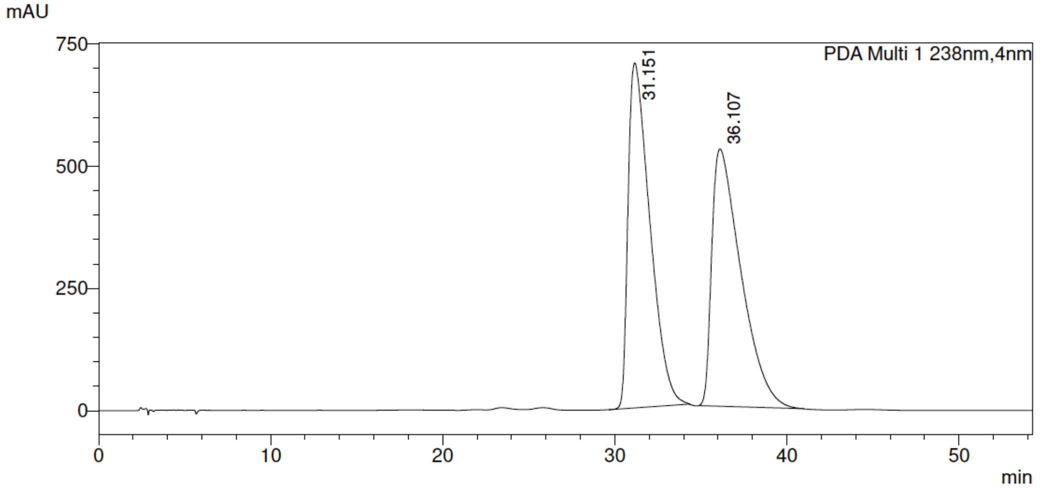


PDA Ch1 238nm

| Peak# | Ret. Time | Area | Height | Area% |
|---|---|---|---|---|
| 1 | 31.151 | 62093344 | 705329 | 50.009 |
| 2 | 36.107 | 62071201 | 526124 | 49.991 |
| Total | | 124164545 | 1231453 | 100.000 |

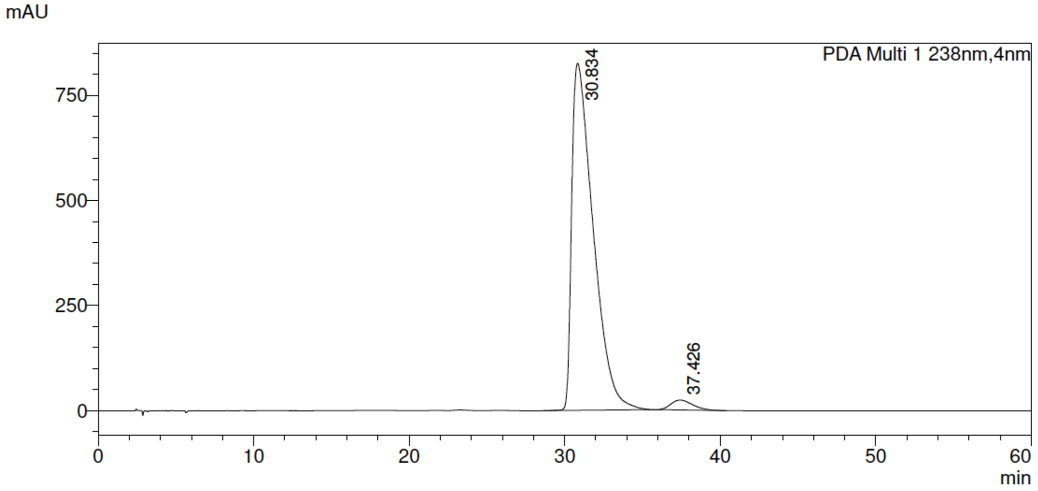


PDA Ch1 238nm

| Peak# | Ret. Time | Area | Height | Area% |
|---|---|---|---|---|
| 1 | 30.834 | 79102148 | 824737 | 97.129 |
| 2 | 37.426 | 2338378 | 23773 | 2.871 |
| Total | | 81440526 | 848509 | 100.000 |

**Supplementary Figure 8.** HPLC spectra for racemic and chiral **3d**

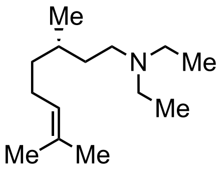


**3e**

25% (95% hexanes, 5% EtOH, 0.2%TFA, 0.1%DEA), 75% hexanes, 0.8 mL/min, CHIRALCEL® OJ-H

er = 95.8:4.2

$[\alpha]_D^{23}$ = +5.90 ($c$ = 1.5.0)

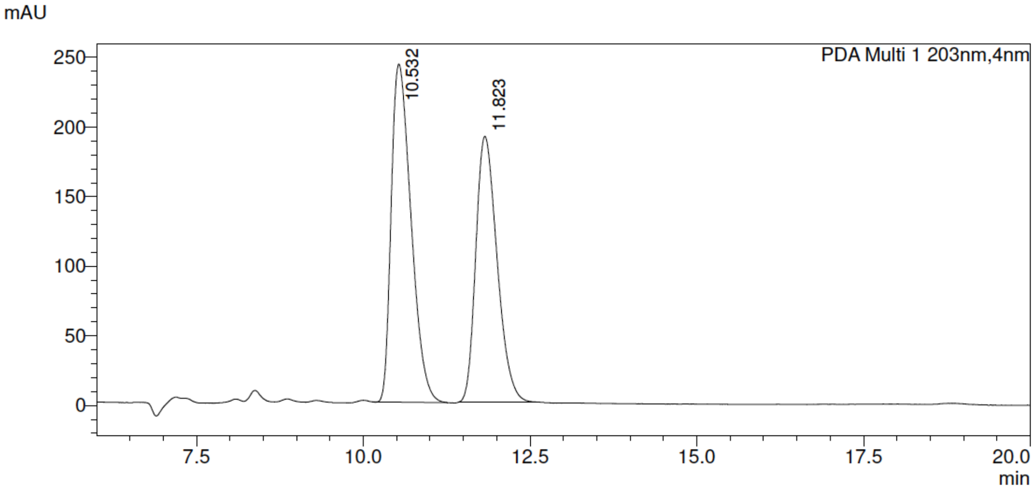


PDA Ch1 203nm

| Peak# | Ret. Time | Area | Height | Area% |
|---|---|---|---|---|
| 1 | 10.532 | 5023170 | 242833 | 54.239 |
| 2 | 11.823 | 4238093 | 190933 | 45.761 |
| Total | | 9261263 | 433765 | 100.000 |

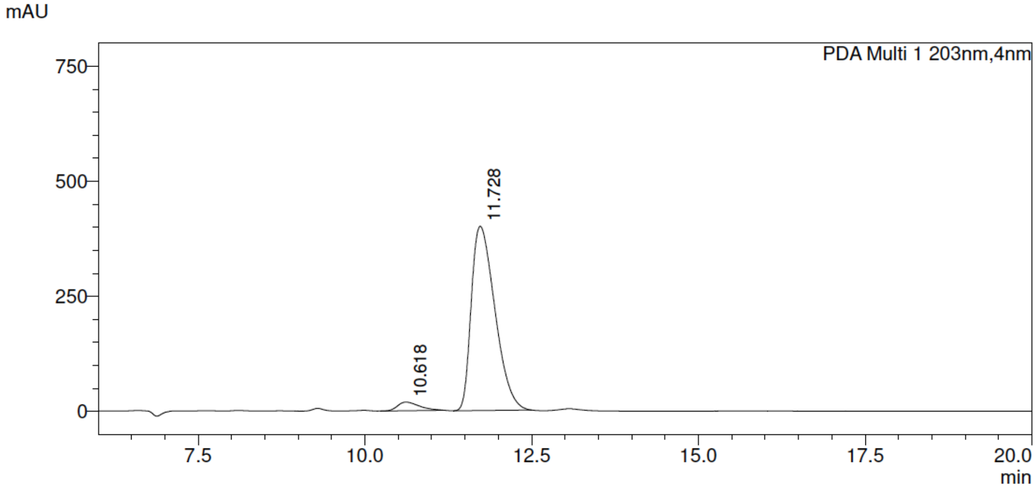


PDA Ch1 203nm

| Peak# | Ret. Time | Area | Height | Area% |
|---|---|---|---|---|
| 1 | 10.618 | 437245 | 19164 | 4.230 |
| 2 | 11.728 | 9900026 | 400551 | 95.770 |
| Total | | 10337271 | 419715 | 100.000 |

**Supplementary Figure 9.** HPLC spectra for racemic and chiral **3e**

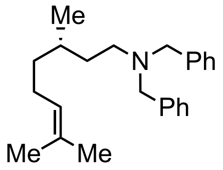


**3f**

50% (95% hexanes, 5% EtOH, 0.2%TFA, 0.1%DEA), 50% hexanes, 0.8 mL/min, CHIRALCEL® OJ-H

er = 95.8 : 4.2

$[\alpha]_D^{23}$ = -0.90 ($c$ = 1.27)

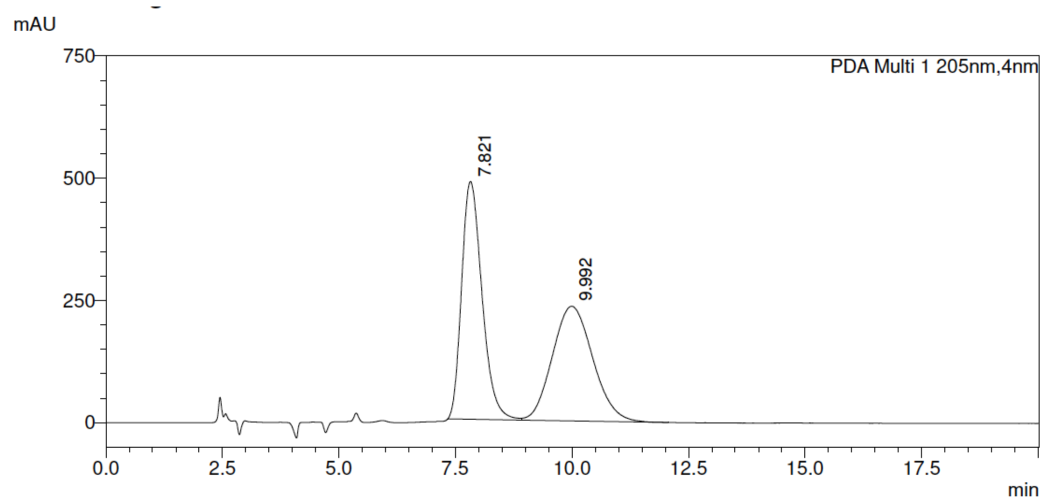


PDA Ch1 205nm

| Peak# | Ret. Time | Area | Height | Conc. |
|---|---|---|---|---|
| 1 | 7.821 | 14809676 | 486321 | 51.130 |
| 2 | 9.992 | 14155320 | 234507 | 48.870 |
| Total | | 28964995 | 720828 | |

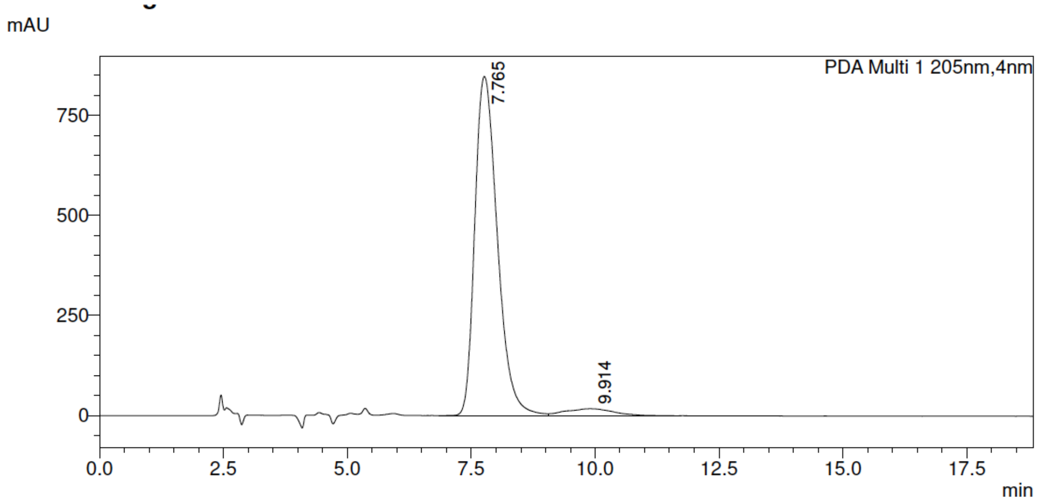


PDA Ch1 205nm

| Peak# | Ret. Time | Area | Height | Area% |
|---|---|---|---|---|
| 1 | 7.765 | 26853663 | 847280 | 95.821 |
| 2 | 9.914 | 1171290 | 17301 | 4.179 |
| Total | | 28024953 | 864581 | 100.000 |

**Supplementary Figure 10.** HPLC spectra for racemic and chiral **3f**

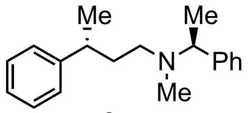


**3g**

50% (95% hexanes, 5% EtOH, 0.2%TFA, 0.1%DEA), 50% hexanes, 0.8 mL/min, CHIRALCEL® OJ-H

er = 99.8:0.2

$[\alpha]_D^{23}$ = -53.81 ($c$ = 1.41)

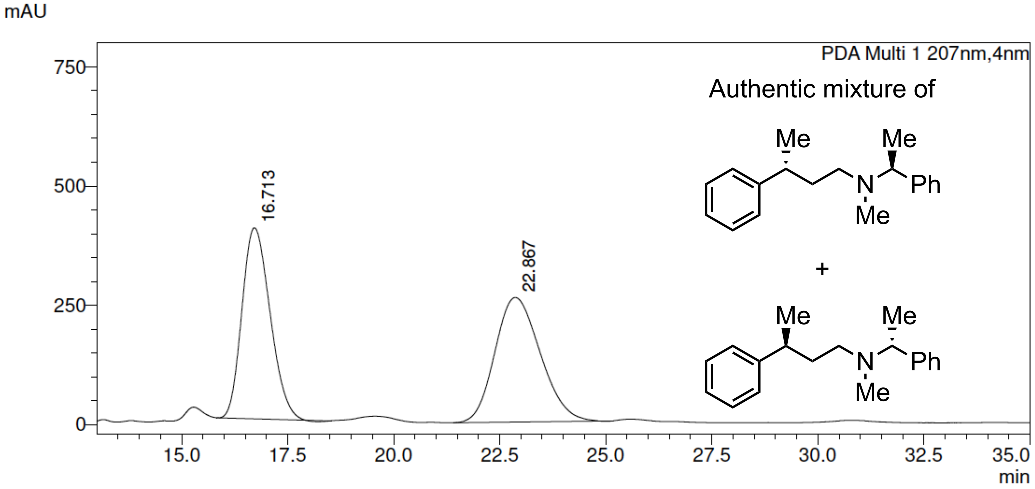


PDA Ch1 207nm

| Peak# | Ret. Time | Area | Height | Area% |
|---|---|---|---|---|
| 1 | 16.713 | 18488064 | 400696 | 48.737 |
| 2 | 22.867 | 19445966 | 261542 | 51.263 |
| Total | | 37934029 | 662238 | 100.000 |

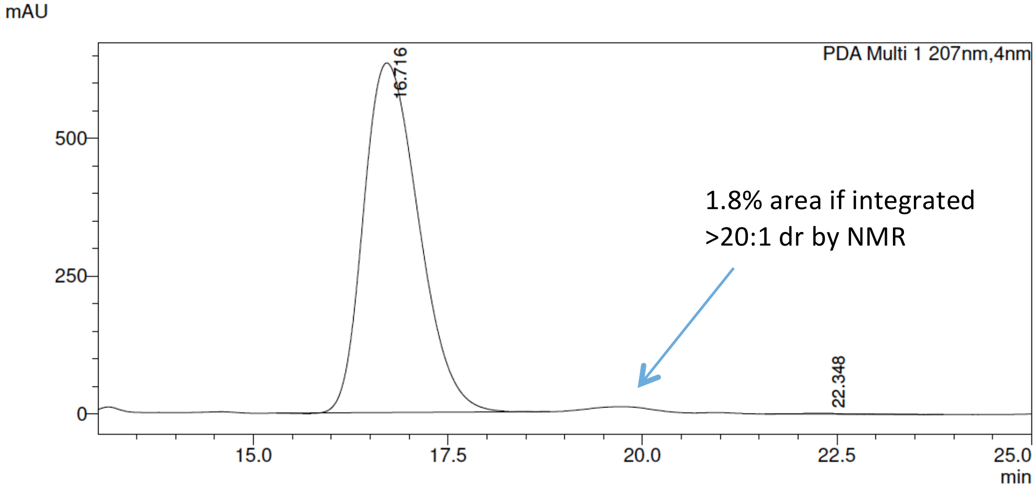


PDA Ch1 207nm

| Peak# | Ret. Time | Area | Height | Area% |
|---|---|---|---|---|
| 1 | 16.716 | 31186056 | 633830 | 99.841 |
| 2 | 22.348 | 49778 | 1370 | 0.159 |
| Total | | 31235834 | 635199 | 100.000 |

**Supplementary Figure 11.** HPLC spectra for racemic and chiral **3g**

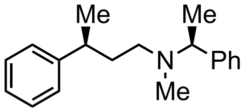

**3g'**

50% (95% hexanes, 5% EtOH, 0.3%TFA, 0.1%DEA), 50% hexanes, 0.8 mL/min, CHIRALPAK® IB3

er = 97.9:2.1

$[\alpha]_D^{23}$ = +5.06 ($c$ = 1.82)

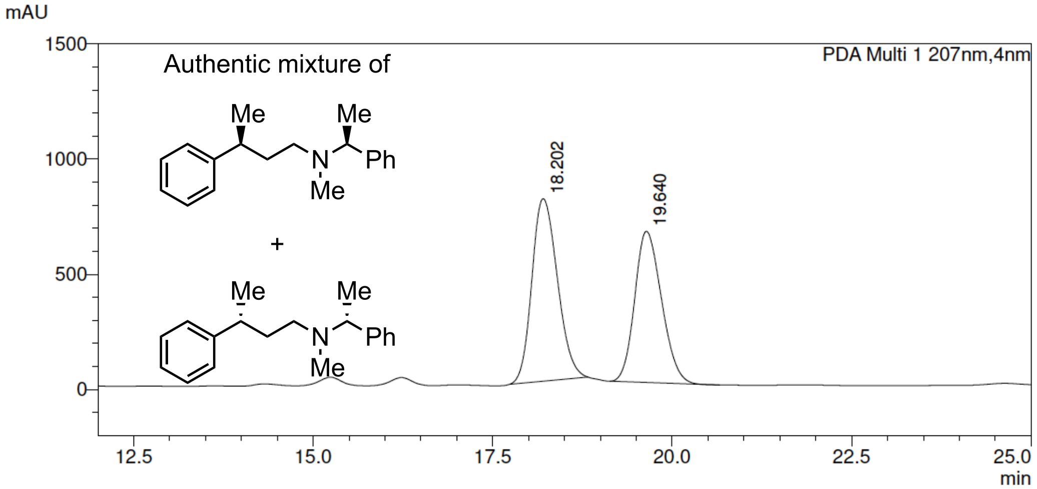


PDA Ch1 207nm

| Peak# | Ret. Time | Area | Height | Area% |
|---|---|---|---|---|
| 1 | 18.202 | 19620406 | 792191 | 53.392 |
| 2 | 19.640 | 17127349 | 656651 | 46.608 |
| Total | | 36747755 | 1448842 | 100.000 |

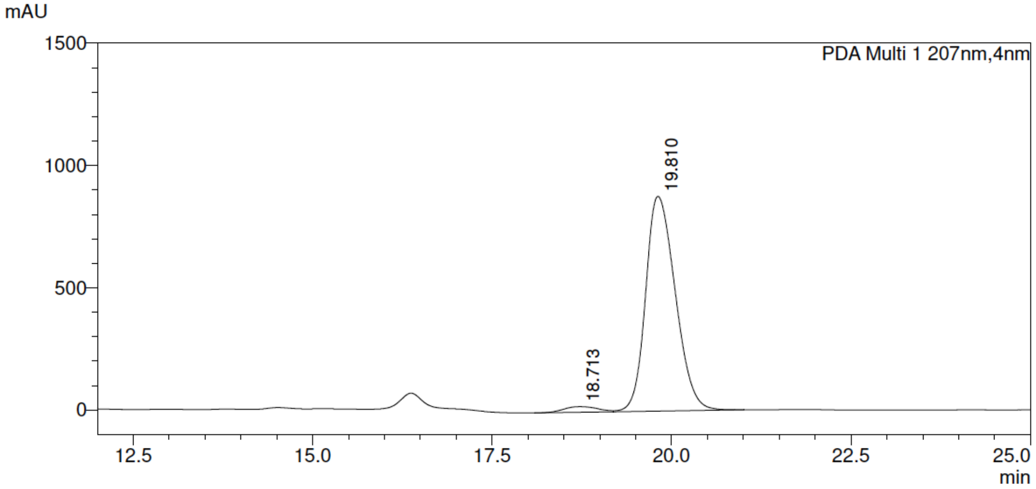


PDA Ch1 207nm

| Peak# | Ret. Time | Area | Height | Area% |
|---|---|---|---|---|
| 1 | 18.713 | 755220 | 23394 | 2.900 |
| 2 | 19.810 | 25288836 | 877926 | 97.100 |
| Total | | 26044057 | 901320 | 100.000 |

**Supplementary Figure 12.** HPLC spectra for racemic and chiral **3g'**

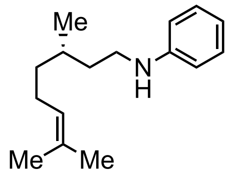

**3h**

10% (95% hexanes, 5% EtOH, 0.2%TFA, 0.1%DEA), 90% hexanes, 0.5 mL/min, CHIRALCEL® OJ-H

er = 96.6:3.4

$[\alpha]_D^{23} = +0.94$ ($c$ = 1.57)

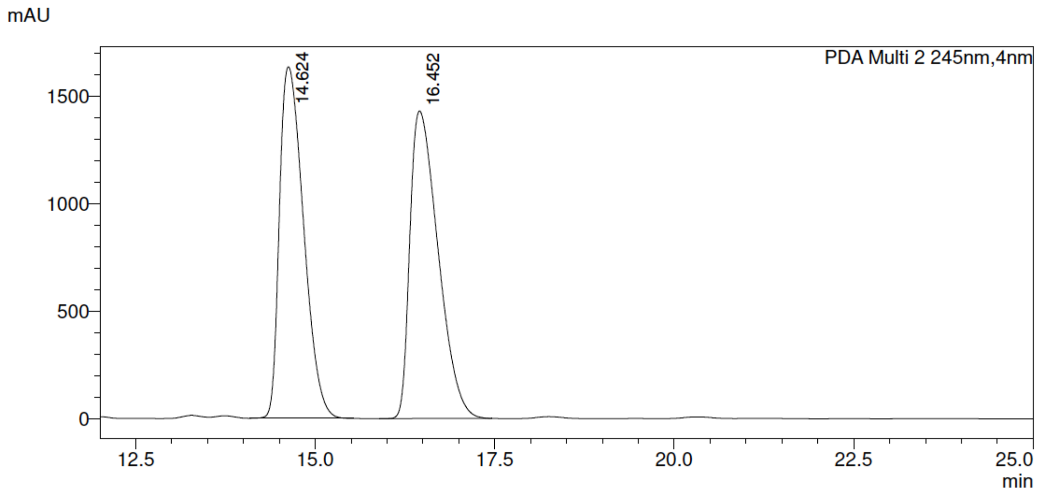


PDA Ch2 245nm

| Peak# | Ret. Time | Area | Height | Area% |
|---|---|---|---|---|
| 1 | 14.624 | 38080564 | 1633112 | 49.408 |
| 2 | 16.452 | 38992596 | 1428836 | 50.592 |
| Total | | 77073160 | 3061948 | 100.000 |

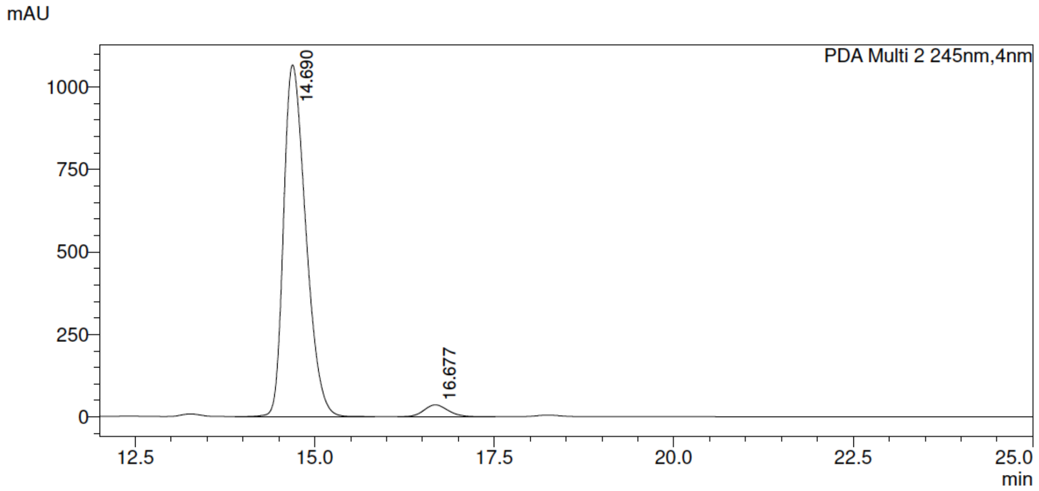


PDA Ch2 245nm

| Peak# | Ret. Time | Area | Height | Area% |
|---|---|---|---|---|
| 1 | 14.690 | 22874777 | 1065611 | 96.580 |
| 2 | 16.677 | 809936 | 35403 | 3.420 |
| Total | | 23684713 | 1101015 | 100.000 |

**Supplementary Figure 13.** HPLC spectra for racemic and chiral **3h**

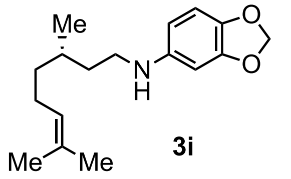

**3i**

15% (95% hexanes, 5% EtOH, 0.2%TFA, 0.1%DEA), 85% hexanes, 0.5 mL/min, CHIRALCEL® OJ-H

er = 96.9 : 3.1

$[\alpha]_D^{23} = +2.92$ ($c$ = 1.37)

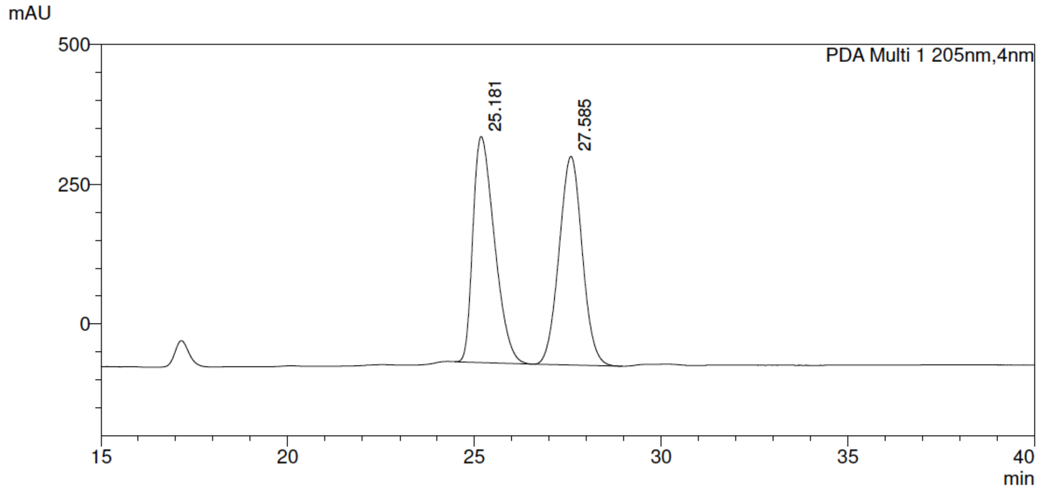


PDA Ch1 205nm

| Peak# | Ret. Time | Area | Height | Area% |
|---|---|---|---|---|
| 1 | 25.181 | 16289356 | 404945 | 50.201 |
| 2 | 27.585 | 16158952 | 373843 | 49.799 |
| Total | | 32448308 | 778787 | 100.000 |

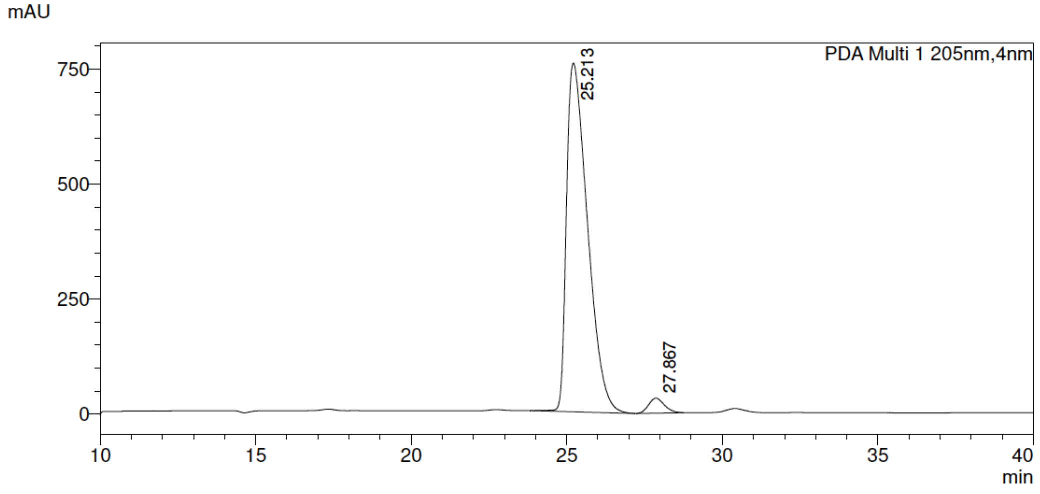


PDA Ch1 205nm

| Peak# | Ret. Time | Area | Height | Area% |
|---|---|---|---|---|
| 1 | 25.213 | 35595600 | 758232 | 96.865 |
| 2 | 27.867 | 1152161 | 32472 | 3.135 |
| Total | | 36747762 | 790703 | 100.000 |

**Supplementary Figure 14.** HPLC spectra for racemic and chiral **3i**

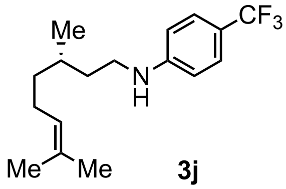


**3j**

**3j** was hydrogenated to **$H_2$-3j** for the determination of er

8% (95% hexanes, 5% EtOH, 0.2%TFA, 0.1%DEA), 92% hexanes, 0.8 mL/min, CHIRALCEL® OJ-H

er = 96.1 : 3.9, $[\alpha]_D^{23}$ = +3.89 ($c$ = 1.39)

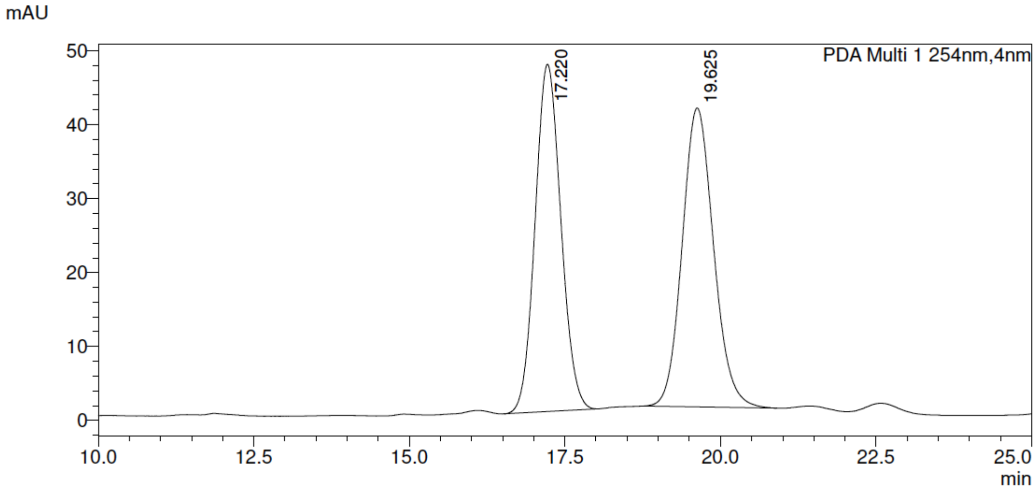


PDA Ch1 254nm

| Peak# | Ret. Time | Area | Height | Area% |
|---|---|---|---|---|
| 1 | 17.220 | 1353114 | 46965 | 48.812 |
| 2 | 19.625 | 1418984 | 40407 | 51.188 |
| Total | | 2772098 | 87372 | 100.000 |

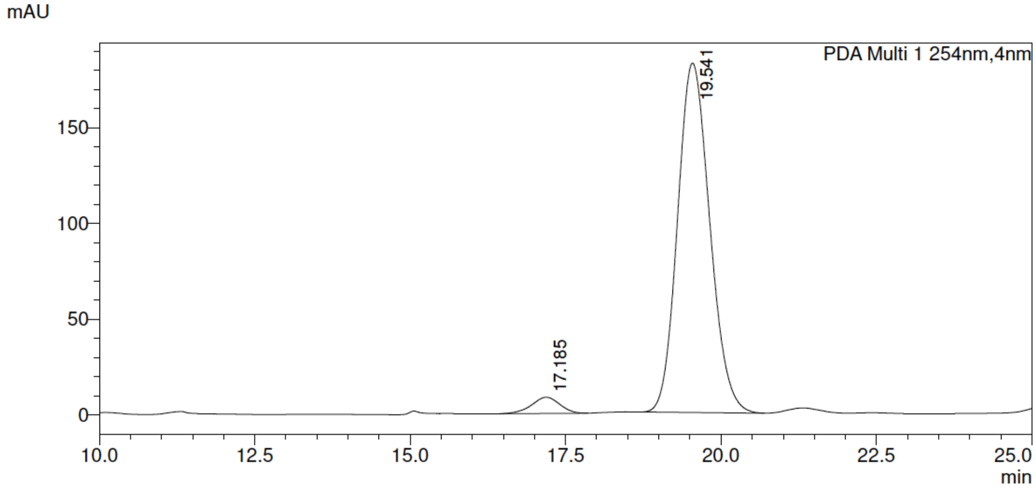


PDA Ch1 254nm

| Peak# | Ret. Time | Area | Height | Area% |
|---|---|---|---|---|
| 1 | 17.185 | 268082 | 8481 | 3.915 |
| 2 | 19.541 | 6579948 | 182416 | 96.085 |
| Total | | 6848031 | 190897 | 100.000 |

**Supplementary Figure 15.** HPLC spectra for racemic and chiral **$H_2$-3j**

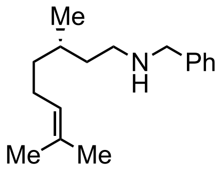


**3k**

15% (95% hexanes, 5% EtOH, 0.2%TFA, 0.1%DEA), 85% hexanes, 0.8 mL/min, CHIRALCEL® OJ-H

er = 97.1 : 2.9

$[\alpha]_D^{23} = +2.30$ ($c$ = 1.22)

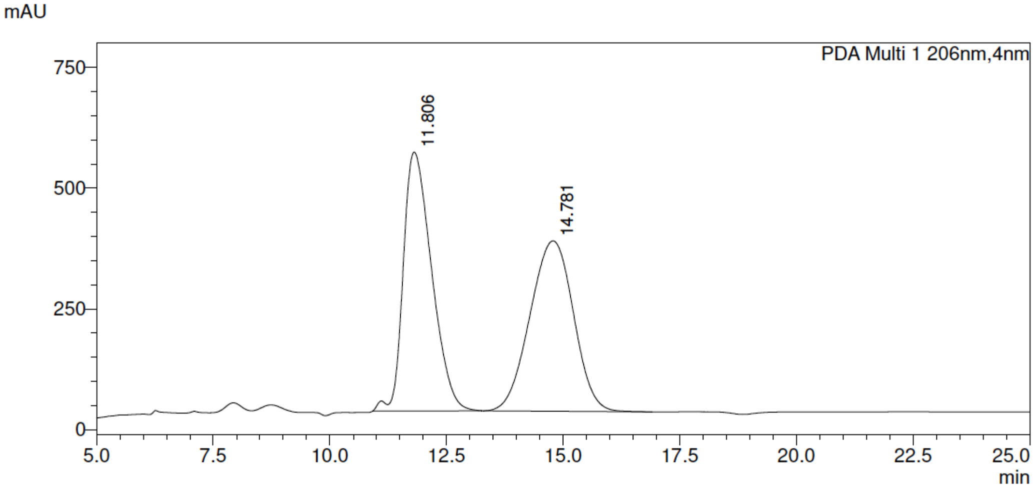


PDA Ch1 206nm

| Peak# | Ret. Time | Area | Height | Area% |
|---|---|---|---|---|
| 1 | 11.806 | 22727270 | 535977 | 49.791 |
| 2 | 14.781 | 22917725 | 352879 | 50.209 |
| Total | | 45644995 | 888856 | 100.000 |

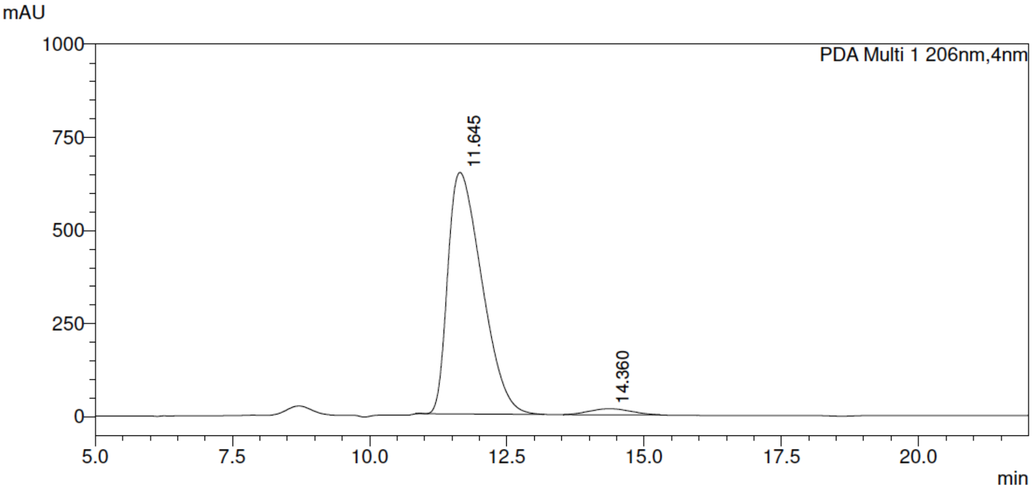


PDA Ch1 206nm

| Peak# | Ret. Time | Area | Height | Area% |
|---|---|---|---|---|
| 1 | 11.645 | 28053490 | 648611 | 97.093 |
| 2 | 14.360 | 839817 | 16129 | 2.907 |
| Total | | 28893307 | 664740 | 100.000 |

**Supplementary Figure 16.** HPLC spectra for racemic and chiral **3k**

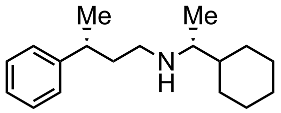

**3l**

50% (95% hexanes, 5% EtOH, 0.2%TFA, 0.1%DEA), 50% hexanes, 0.8 mL/min, CHIRALPAK® ID3

er = 98.7:1.3

$[\alpha]_D^{23}$ = -29.98 ($c$ = 1.04)

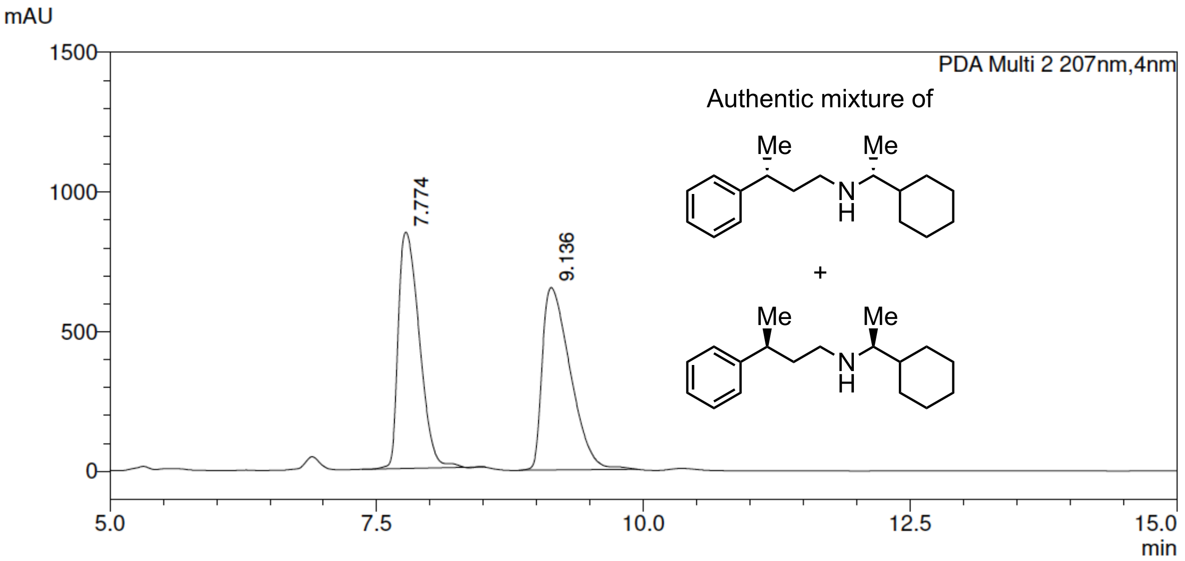


PDA Ch2 207nm

| Peak# | Ret. Time | Area | Height | Area% |
|---|---|---|---|---|
| 1 | 7.774 | 11673670 | 846410 | 49.351 |
| 2 | 9.136 | 11980896 | 653596 | 50.649 |
| Total | | 23654566 | 1500006 | 100.000 |

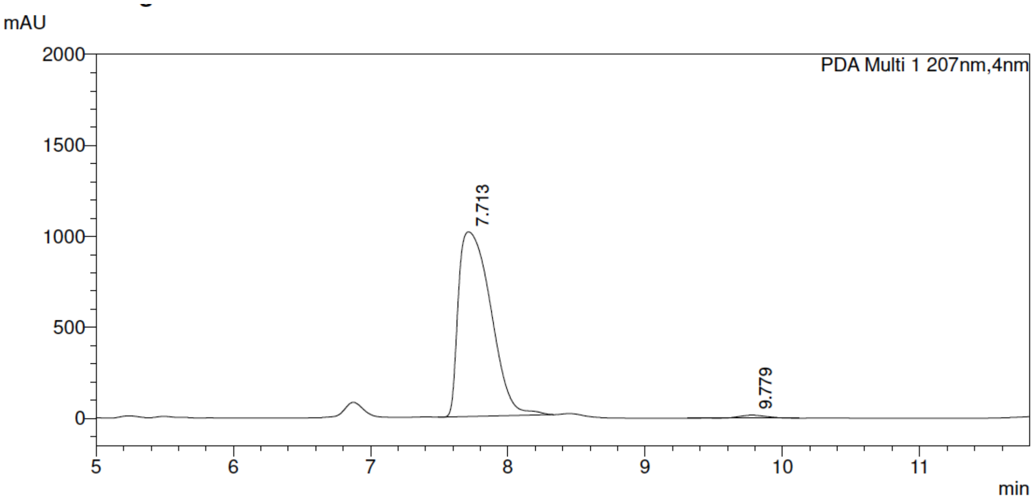


PDA Ch1 207nm

| Peak# | Ret. Time | Area | Height | Area% |
|---|---|---|---|---|
| 1 | 7.713 | 16698897 | 1014797 | 98.661 |
| 2 | 9.779 | 226615 | 16468 | 1.339 |
| Total | | 16925513 | 1031265 | 100.000 |

**Supplementary Figure 17.** HPLC spectra for racemic and chiral **3l**

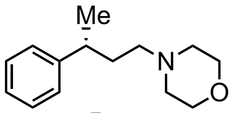


**5a**

100% (95% hexanes, 5% EtOH, 0.2%TFA, 0.1%DEA), 0.8 mL/min, CHIRALCEL® OJ-H

er = 98.7:1.3

$[\alpha]_D^{23}$ = -22.61 ($c$ = 1.15)

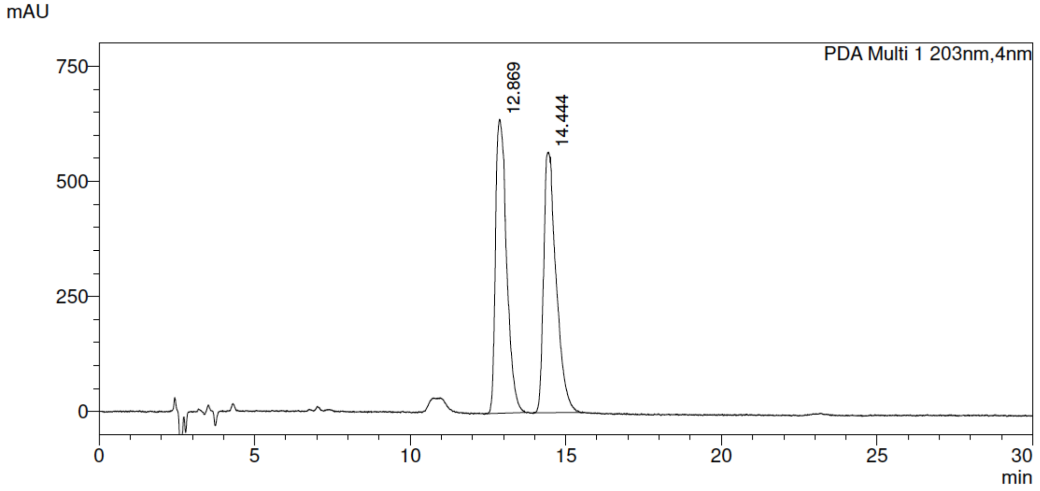


PDA Ch1 203nm

| Peak# | Ret. Time | Area | Height | Area% |
|---|---|---|---|---|
| 1 | 12.869 | 15832954 | 638093 | 49.993 |
| 2 | 14.444 | 15837223 | 566309 | 50.007 |
| Total | | 31670178 | 1204401 | 100.000 |

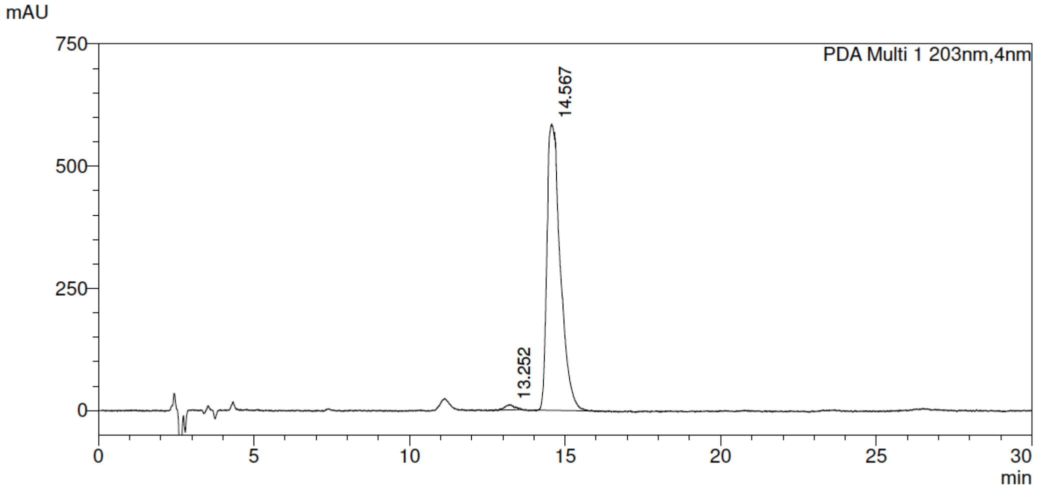


PDA Ch1 203nm

| Peak# | Ret. Time | Area | Height | Area% |
|---|---|---|---|---|
| 1 | 13.252 | 231539 | 10478 | 1.306 |
| 2 | 14.567 | 17495432 | 585933 | 98.694 |
| Total | | 17726971 | 596411 | 100.000 |

**Supplementary Figure 18.** HPLC spectra for racemic and chiral **5a**

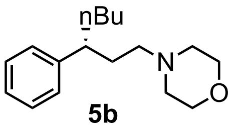


**5b**

50% (95% hexanes, 5% EtOH, 0.2%TFA, 0.1%DEA), 50% hexanes, 0.8 mL/min, CHIRALPAK® IA3

er = 99.1:0.9

$[\alpha]_D^{23}$ = -10.85 ($c$ = 2.12)

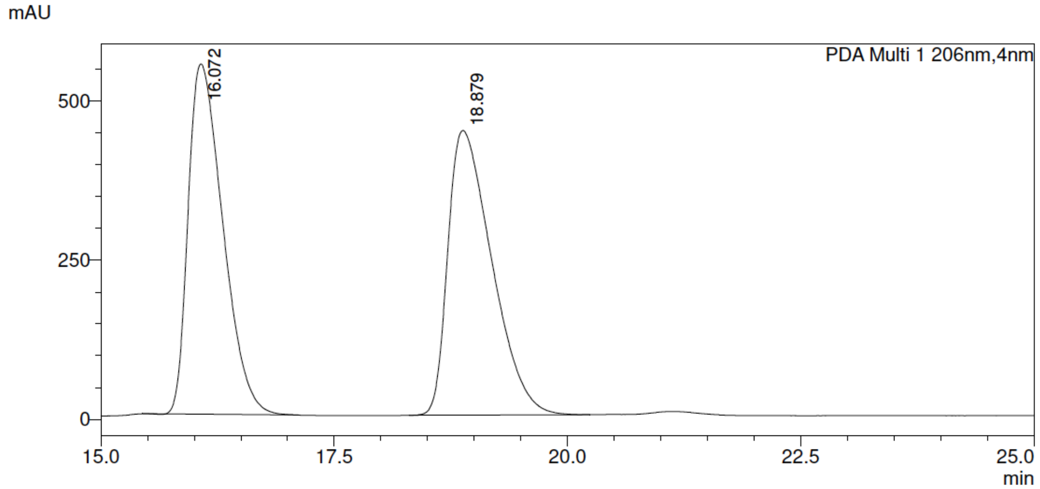


PDA Ch1 206nm

| Peak# | Ret. Time | Area | Height | Area% |
|---|---|---|---|---|
| 1 | 16.072 | 14246867 | 550302 | 49.306 |
| 2 | 18.879 | 14648200 | 447090 | 50.694 |
| Total | | 28895068 | 997392 | 100.000 |

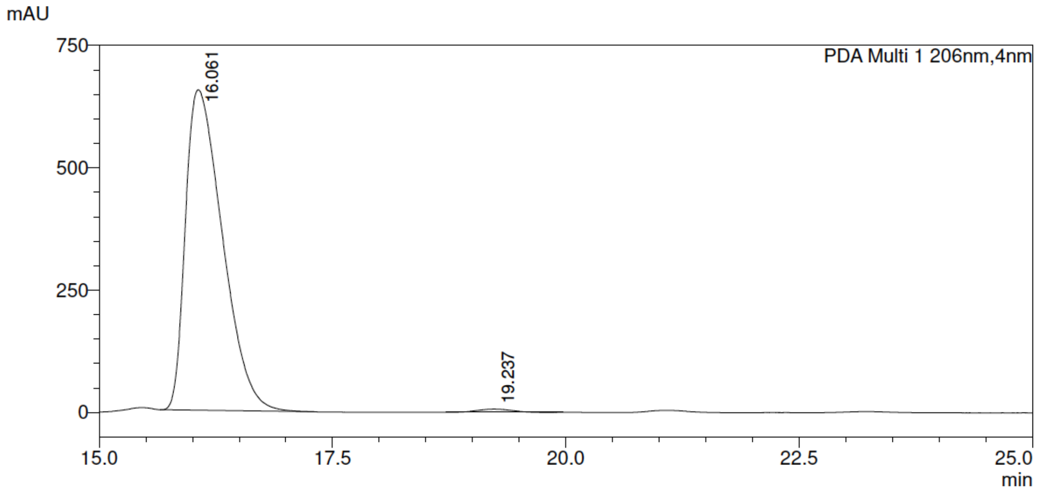


PDA Ch1 206nm

| Peak# | Ret. Time | Area | Height | Area% |
|---|---|---|---|---|
| 1 | 16.061 | 18071421 | 655107 | 99.088 |
| 2 | 19.237 | 166303 | 6740 | 0.912 |
| Total | | 18237725 | 661847 | 100.000 |

**Supplementary Figure 19.** HPLC spectra for racemic and chiral **5b**

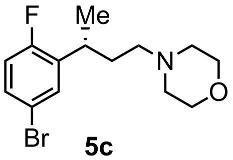

**5c**

30% (95% hexanes, 5% EtOH, 0.2%TFA, 0.1%DEA), 70% hexanes, 0.8 mL/min, CHIRALPAK® IA3

er = 97.8:2.2

$[\alpha]_D^{23}$ = -33.09 ($c$ = 1.06)

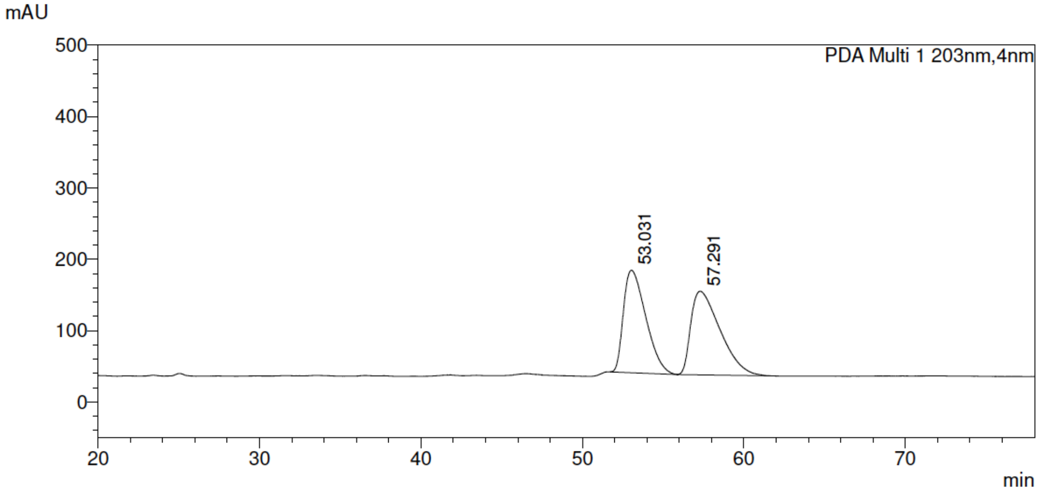


PDA Ch1 203nm

| Peak# | Ret. Time | Area | Height | Area% |
|---|---|---|---|---|
| 1 | 53.031 | 13837009 | 143708 | 48.335 |
| 2 | 57.291 | 14790355 | 117209 | 51.665 |
| Total | | 28627365 | 260917 | 100.000 |

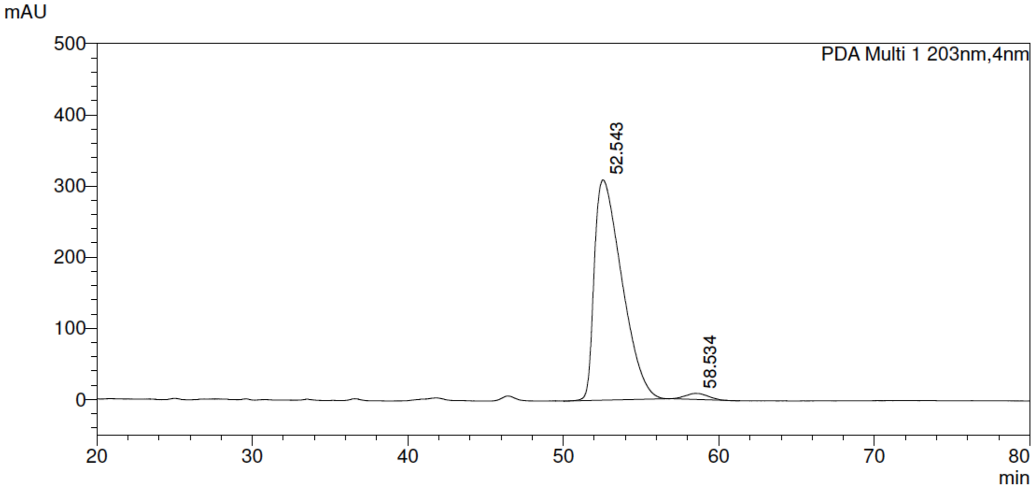


PDA Ch1 203nm

| Peak# | Ret. Time | Area | Height | Area% |
|---|---|---|---|---|
| 1 | 52.543 | 38237378 | 309568 | 97.804 |
| 2 | 58.534 | 858562 | 8644 | 2.196 |
| Total | | 39095940 | 318212 | 100.000 |

**Supplementary Figure 20.** HPLC spectra for racemic and chiral **5c**

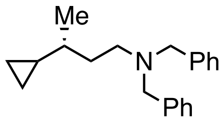

**5e**

10% (95% hexanes, 5% EtOH, 0.2%TFA, 0.1%DEA), 90% hexanes, 0.8 mL/min, CHIRALCEL® OJ-H

er = 98.9 : 1.1

$[\alpha]_D^{23} = 8.84$ ($c$ = 1.74)

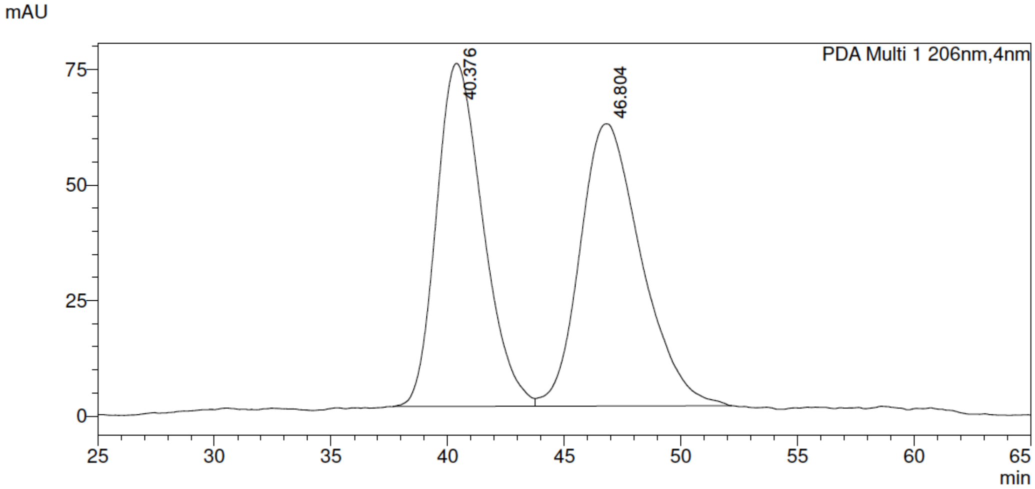


PDA Ch1 206nm

| Peak# | Ret. Time | Area | Height | Area% |
|---|---|---|---|---|
| 1 | 40.376 | 10298183 | 74278 | 47.947 |
| 2 | 46.804 | 11180038 | 61121 | 52.053 |
| Total | | 21478221 | 135400 | 100.000 |

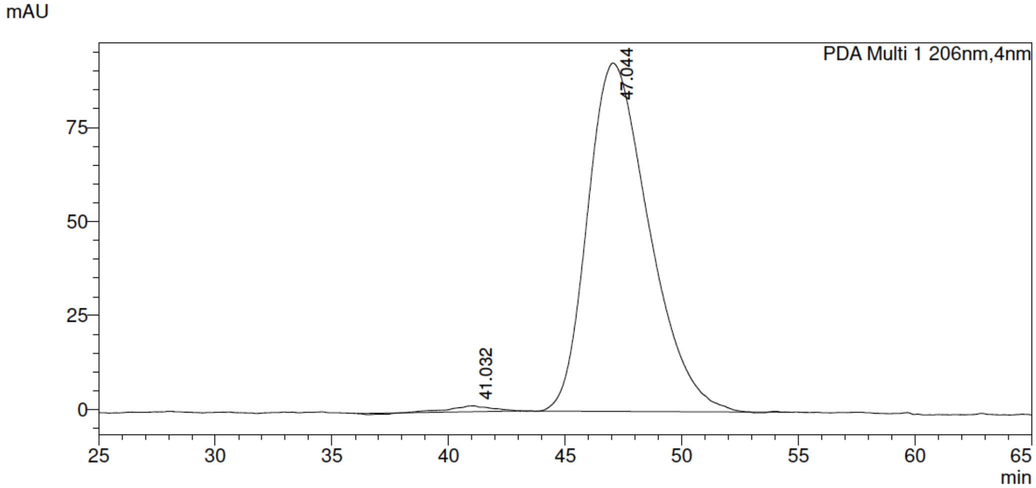


PDA Ch1 206nm

| Peak# | Ret. Time | Area | Height | Area% |
|---|---|---|---|---|
| 1 | 41.032 | 190116 | 1562 | 1.107 |
| 2 | 47.044 | 16985697 | 92714 | 98.893 |
| Total | | 17175813 | 94276 | 100.000 |

**Supplementary Figure 21.** HPLC spectra for racemic and chiral **5e**

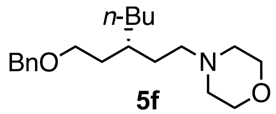


**5f**

50% (95% hexanes, 5% EtOH, 0.2%TFA, 0.1%DEA), 50% hexanes, 0.8 mL/min, CHIRALCEL® OJ-H

er = 98.1 : 1.9

$[\alpha]_D^{23} = 0.72$ ($c$ = 1.77)

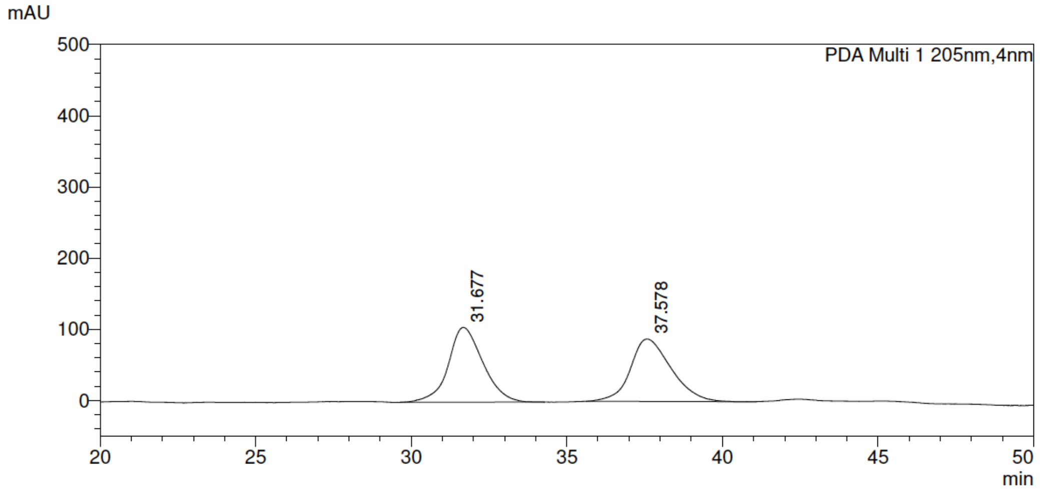


PDA Ch1 205nm

| Peak# | Ret. Time | Area | Height | Area% |
|---|---|---|---|---|
| 1 | 31.677 | 8177026 | 105089 | 50.601 |
| 2 | 37.578 | 7982873 | 87695 | 49.399 |
| Total | | 16159899 | 192784 | 100.000 |

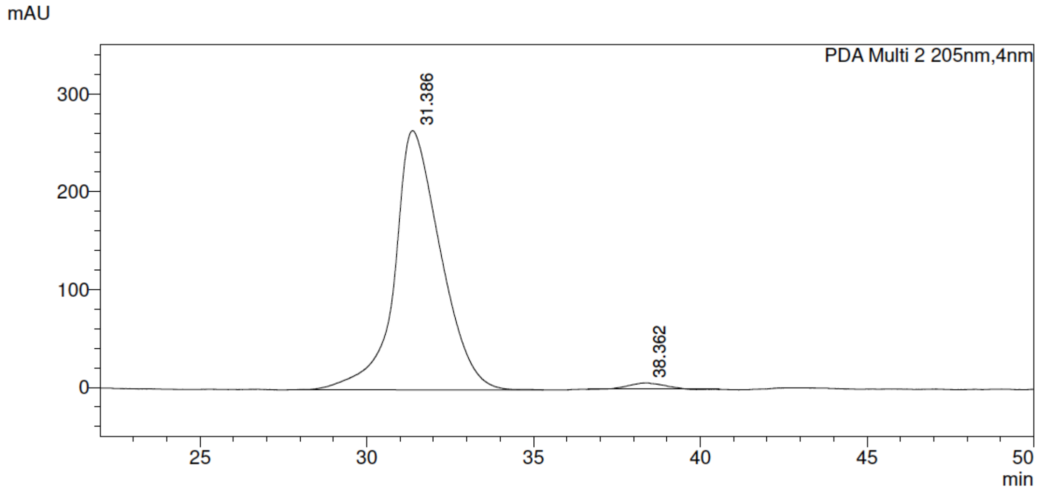


PDA Ch2 205nm

| Peak# | Ret. Time | Area | Height | Area% |
|---|---|---|---|---|
| 1 | 31.386 | 25684603 | 264916 | 98.079 |
| 2 | 38.362 | 503084 | 6450 | 1.921 |
| Total | | 26187686 | 271367 | 100.000 |

**Supplementary Figure 22.** HPLC spectra for racemic and chiral **5f**

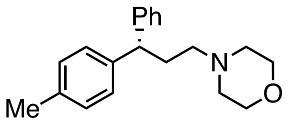

**5g**

50% (95% hexanes, 5% EtOH, 0.2%TFA, 0.1%DEA), 50% hexanes, 0.8 mL/min, CHIRALCEL® OJ-H

er = 96.5 : 3.5

$[\alpha]_D^{23} = 2.63$ ($c$ = 2.28)

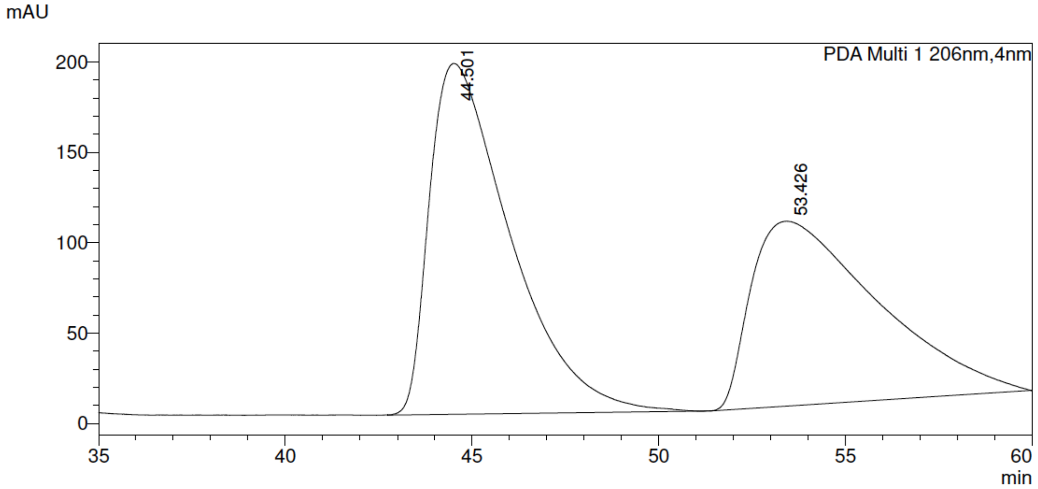

PDA Ch1 206nm

| Peak# | Ret. Time | Area | Height | Area% |
|---|---|---|---|---|
| 1 | 44.501 | 29184938 | 193973 | 54.829 |
| 2 | 53.426 | 24044256 | 102258 | 45.171 |
| Total | | 53229194 | 296230 | 100.000 |

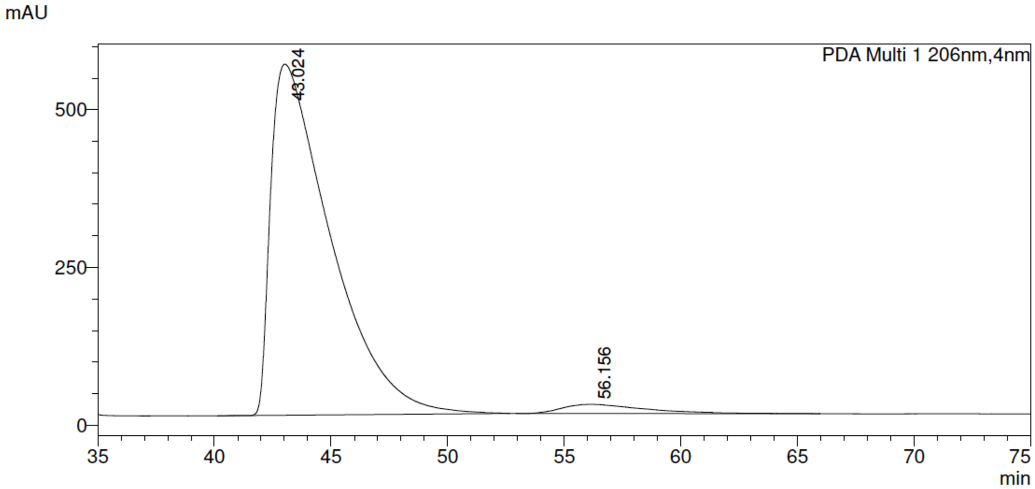

PDA Ch1 206nm

| Peak# | Ret. Time | Area | Height | Area% |
|---|---|---|---|---|
| 1 | 43.024 | 99442289 | 555842 | 96.538 |
| 2 | 56.156 | 3565794 | 14361 | 3.462 |
| Total | | 103008083 | 570203 | 100.000 |

**Supplementary Figure 23.** HPLC spectra for racemic and chiral **5g**

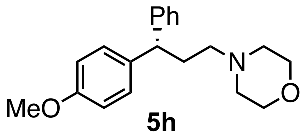


100% (95% hexanes, 5% EtOH, 0.2%TFA, 0.1%DEA), 0.8 mL/min, CHIRALCEL® OJ-H

er = 97.6 : 2.4

$[\alpha]_D^{23} = 1.49$ ($c$ = 1.05)

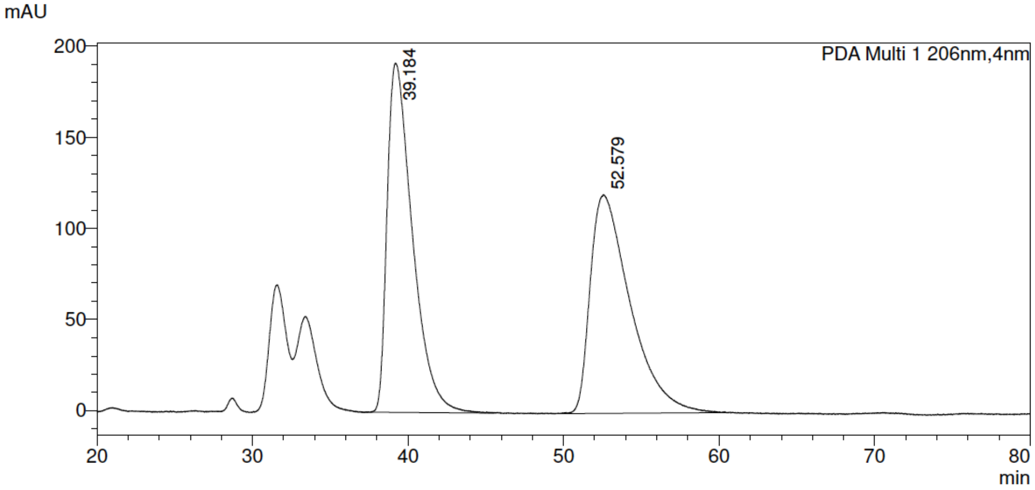


PDA Ch1 206nm

| Peak# | Ret. Time | Area | Height | Area% |
|---|---|---|---|---|
| 1 | 39.184 | 21696765 | 191679 | 50.302 |
| 2 | 52.579 | 21435894 | 120133 | 49.698 |
| Total | | 43132658 | 311813 | 100.000 |

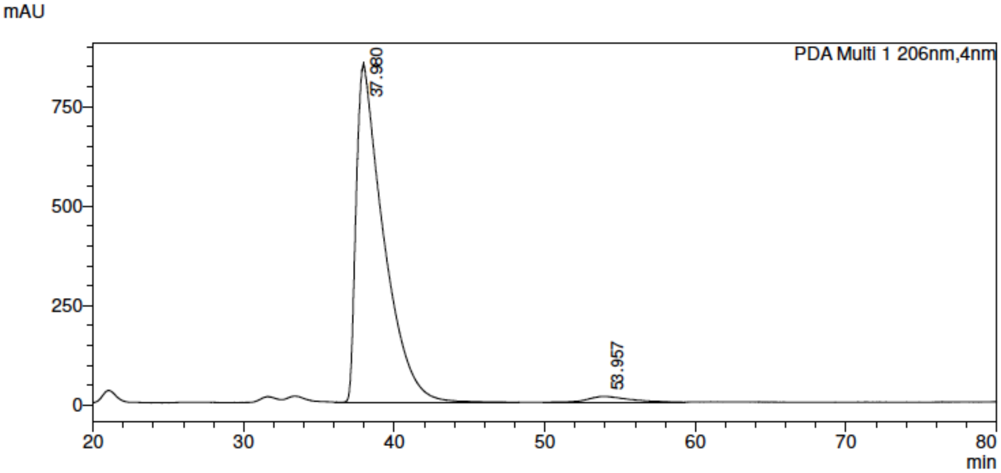


PDA Ch1 206nm

| Peak# | Ret. Time | Area | Height | Area% |
|---|---|---|---|---|
| 1 | 37.980 | 106654378 | 849883 | 97.624 |
| 2 | 53.957 | 2596321 | 14115 | 2.376 |
| Total | | 109250699 | 863998 | 100.000 |

**Supplementary Figure 24.** HPLC spectra for racemic and chiral **5h**

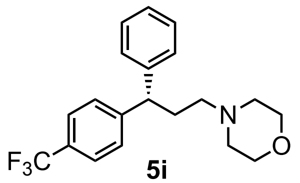

**5i**

50% (95% hexanes, 5% EtOH, 0.2%TFA, 0.1%DEA), 50% hexanes, 0.8 mL/min, CHIRALPAK® IA3

er = 98.6 : 1.4

$[\alpha]_D^{23}$ = -1.38 ($c$ = 1.97)

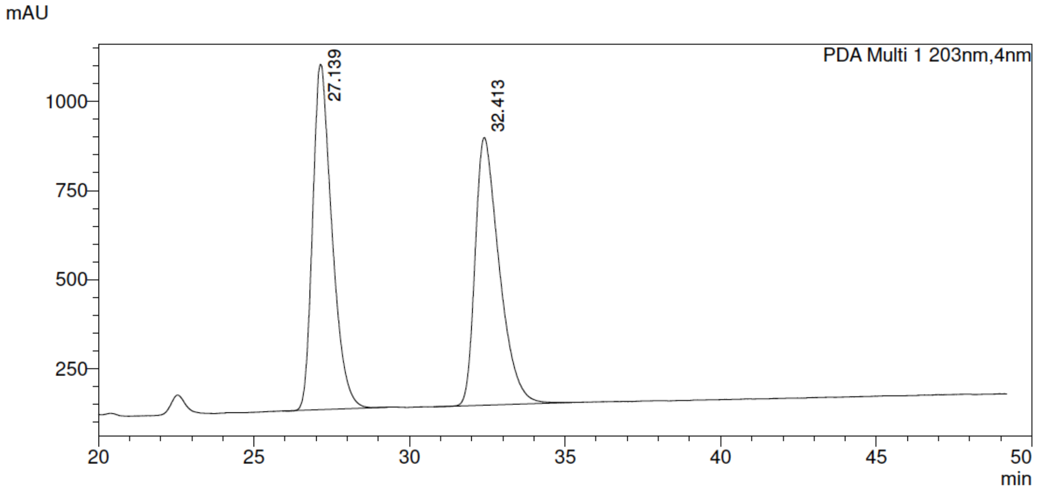


PDA Ch1 203nm

| Peak# | Ret. Time | Area | Height | Area% |
|---|---|---|---|---|
| 1 | 27.139 | 41885612 | 968486 | 50.790 |
| 2 | 32.413 | 40583421 | 750623 | 49.210 |
| Total | | 82469033 | 1719109 | 100.000 |

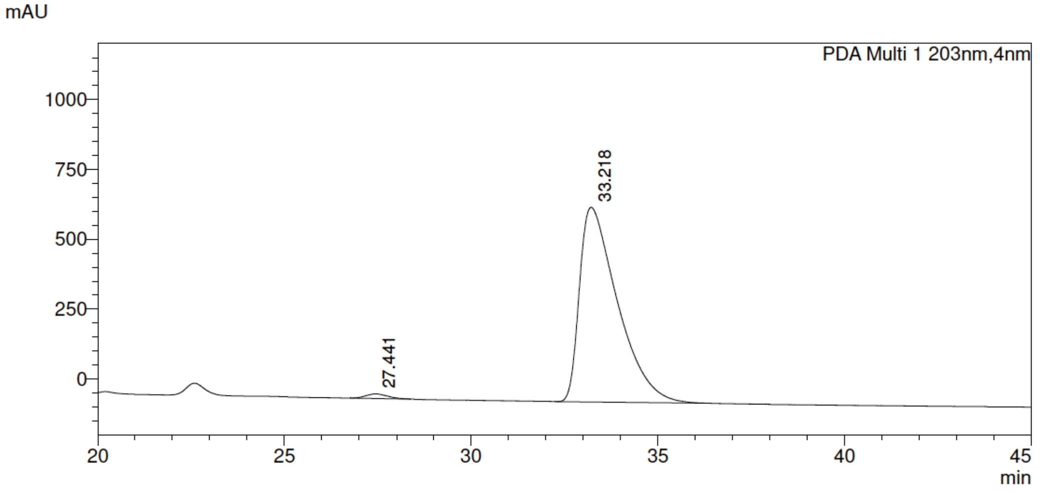


PDA Ch1 203nm

| Peak# | Ret. Time | Area | Height | Area% |
|---|---|---|---|---|
| 1 | 27.441 | 677383 | 16481 | 1.352 |
| 2 | 33.218 | 49413810 | 697168 | 98.648 |
| Total | | 50091192 | 713649 | 100.000 |

**Supplementary Figure 25.** HPLC spectra for racemic and chiral **5i**

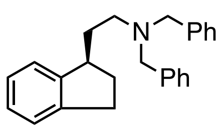

**5j**

100% (95% hexanes, 5% EtOH, 0.2%TFA, 0.1%DEA), 0.8 mL/min, CHIRALPAK® IB3

er = 92.5 : 7.5

$[\alpha]_D^{23}$ = 1.70 ($c$ = 1.80)

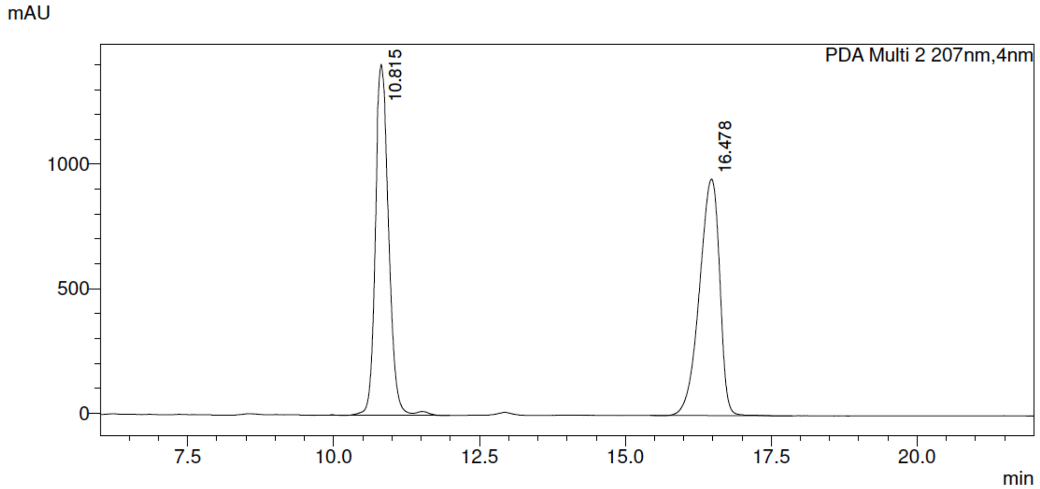


PDA Ch2 207nm

| Peak# | Ret. Time | Area | Height | Area% |
|---|---|---|---|---|
| 1 | 10.815 | 22292584 | 1408843 | 49.391 |
| 2 | 16.478 | 22841896 | 949498 | 50.609 |
| Total | | 45134480 | 2358341 | 100.000 |

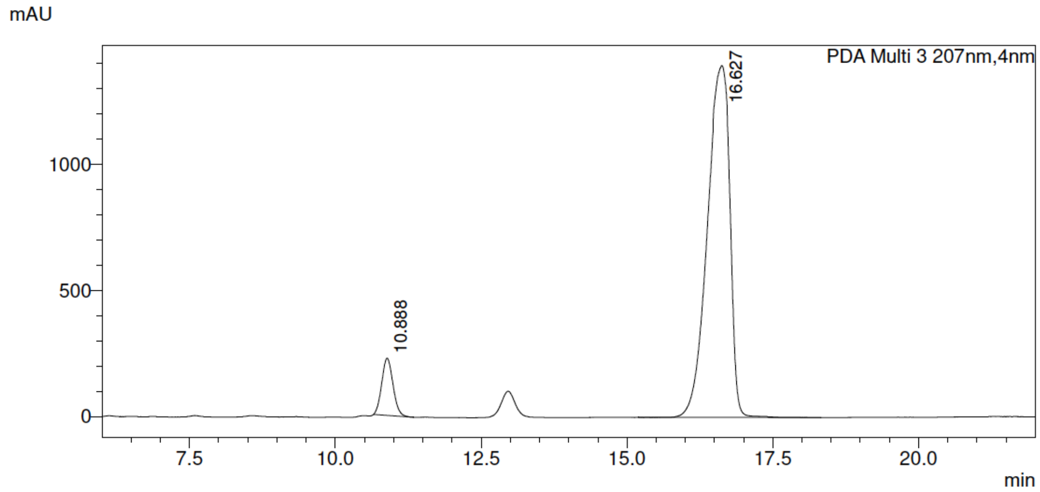


PDA Ch3 207nm

| Peak# | Ret. Time | Area | Height | Area% |
|---|---|---|---|---|
| 1 | 10.888 | 3039350 | 226339 | 7.505 |
| 2 | 16.627 | 37459855 | 1394936 | 92.495 |
| Total | | 40499205 | 1621275 | 100.000 |

**Supplementary Figure 26.** HPLC spectra for racemic and chiral **5j**

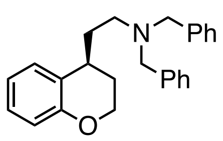

**5k**

100% (95% hexanes, 5% EtOH, 0.2%TFA, 0.1%DEA), 0.8 mL/min, CHIRALPAK® IB3

er = 97.2 : 2.8

$[\alpha]_D^{23}$ = -13.51 ($c$ = 2.32)

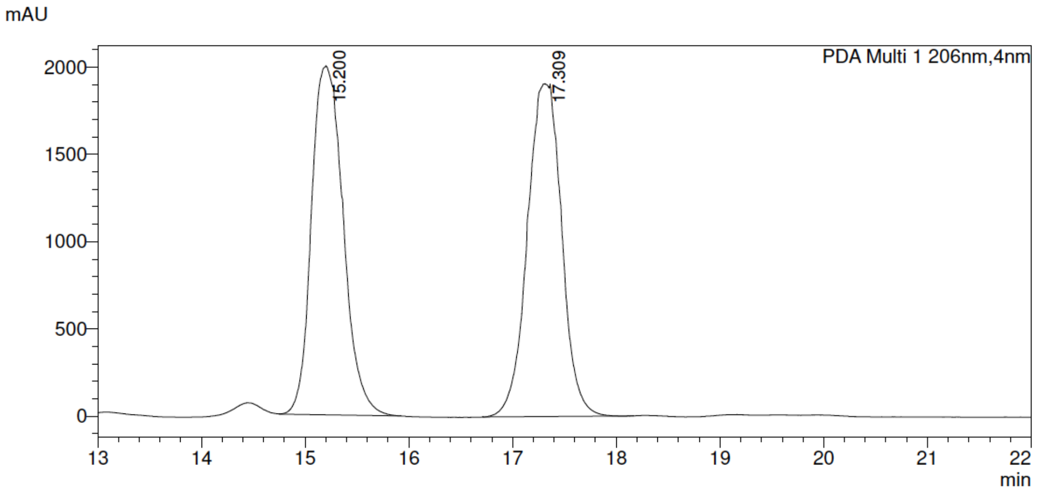


PDA Ch1 206nm

| Peak# | Ret. Time | Area | Height | Area% |
|---|---|---|---|---|
| 1 | 15.200 | 42613580 | 1997405 | 49.327 |
| 2 | 17.309 | 43777091 | 1907050 | 50.673 |
| Total | | 86390672 | 3904456 | 100.000 |

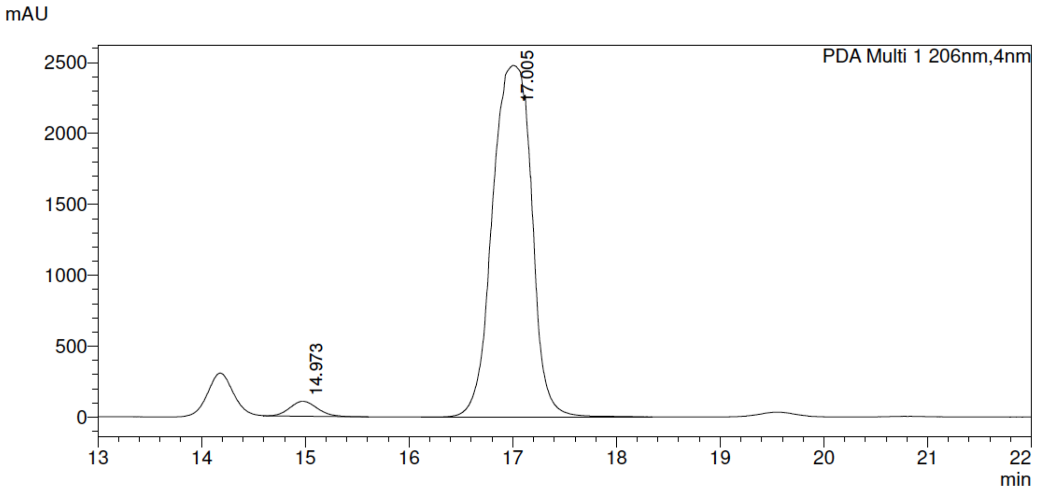


PDA Ch1 206nm

| Peak# | Ret. Time | Area | Height | Area% |
|---|---|---|---|---|
| 1 | 14.973 | 1902188 | 104915 | 2.842 |
| 2 | 17.005 | 65017948 | 2477029 | 97.158 |
| Total | | 66920135 | 2581944 | 100.000 |

**Supplementary Figure 27.** HPLC spectra for racemic and chiral **5k**

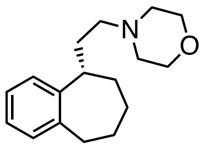

**5l**

50% (95% hexanes, 5% EtOH, 0.2%TFA, 0.1%DEA), 50% hexanes, 0.8 mL/min, CHIRALPAK® IB3

er = 96.0 : 4.0

$[\alpha]_D^{23}$ = 16.81 ($c$ = 1.16)

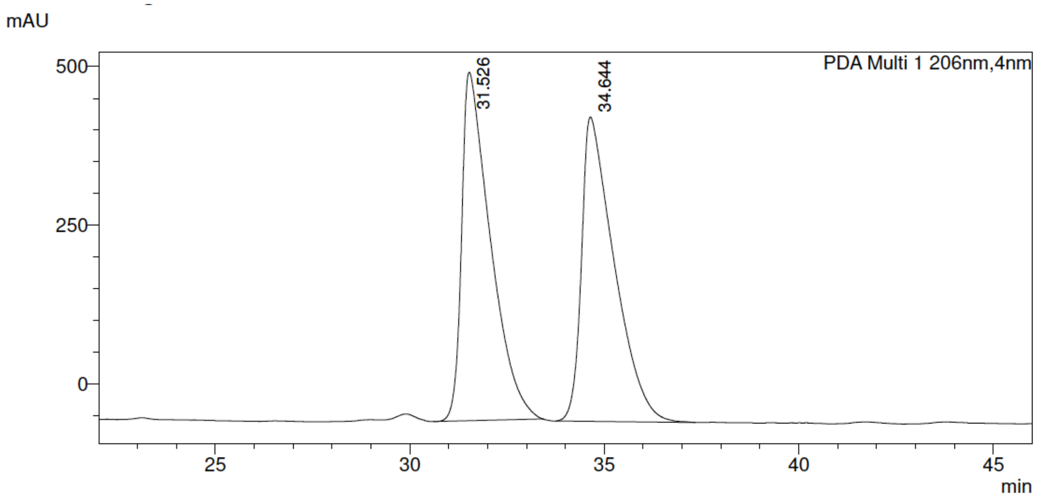


PDA Ch1 206nm

| Peak# | Ret. Time | Area | Height | Area% |
|---|---|---|---|---|
| 1 | 31.526 | 28259562 | 548678 | 50.192 |
| 2 | 34.644 | 28042812 | 478896 | 49.808 |
| Total | | 56302374 | 1027574 | 100.000 |

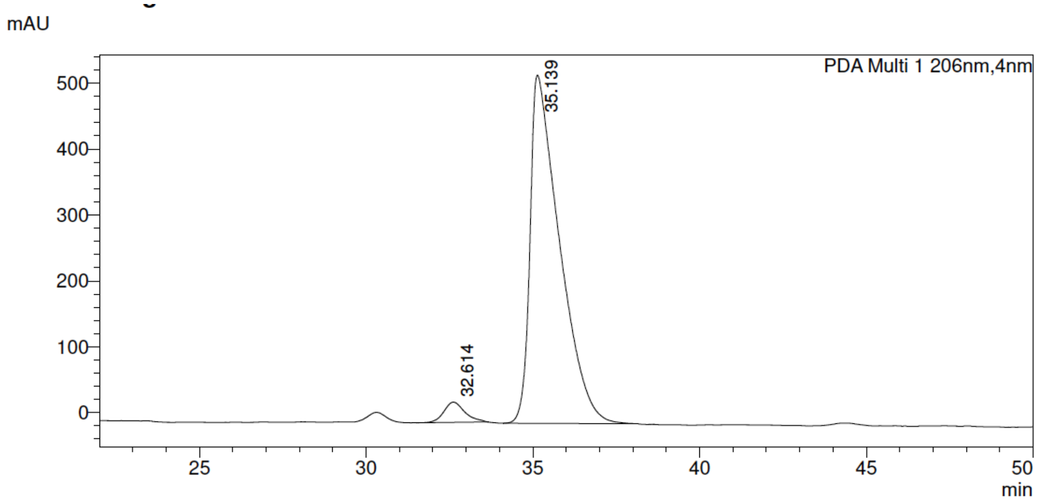


PDA Ch1 206nm

| Peak# | Ret. Time | Area | Height | Area% |
|---|---|---|---|---|
| 1 | 32.614 | 1343915 | 30711 | 3.967 |
| 2 | 35.139 | 32533565 | 528469 | 96.033 |
| Total | | 33877480 | 559180 | 100.000 |

**Supplementary Figure 28.** HPLC spectra for racemic and chiral **5l**

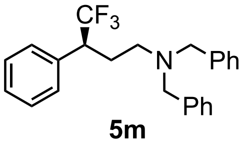


**5m**

100% (95% hexanes, 5% EtOH, 0.2%TFA, 0.1%DEA), 0.8 mL/min, CHIRALCEL® OJ-H

er = 98.6 : 1.4

$[\alpha]_D^{23}$ = 85.78 ($c$ = 2.12)

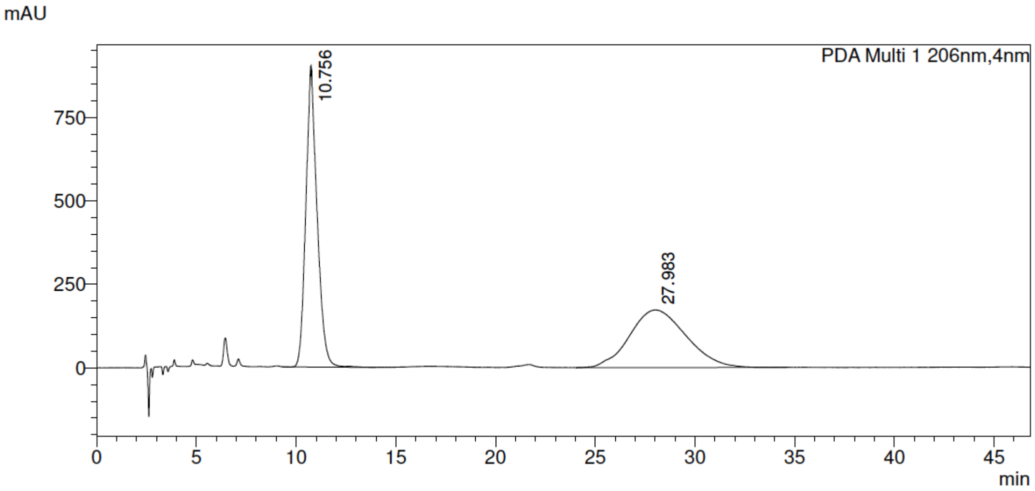


PDA Ch1 206nm

| Peak# | Ret. Time | Area | Height | Area% |
|---|---|---|---|---|
| 1 | 10.756 | 35119358 | 899382 | 50.822 |
| 2 | 27.983 | 33983624 | 172707 | 49.178 |
| Total | | 69102982 | 1072088 | 100.000 |

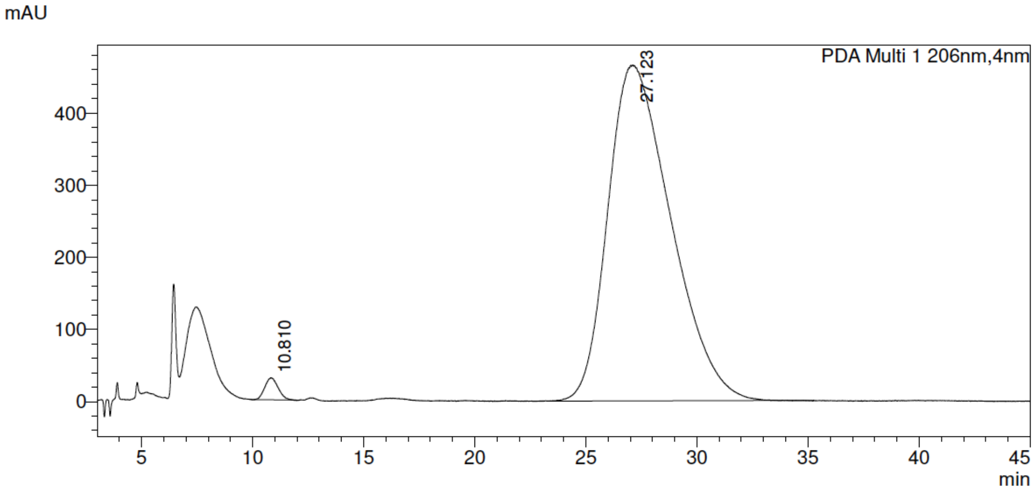


PDA Ch1 206nm

| Peak# | Ret. Time | Area | Height | Area% |
|---|---|---|---|---|
| 1 | 10.810 | 1258992 | 30385 | 1.352 |
| 2 | 27.123 | 91873672 | 465456 | 98.648 |
| Total | | 93132664 | 495840 | 100.000 |

**Supplementary Figure 29.** HPLC spectra for racemic and chiral **5m**

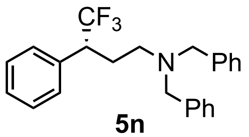

**5n**

100% (95% hexanes, 5% EtOH, 0.2%TFA, 0.1%DEA), 0.8 mL/min, CHIRALCEL® OJ-H

er = 98.2 : 1.8

$[\alpha]_D^{23}$ = -79.65 ($c$ = 1.94)

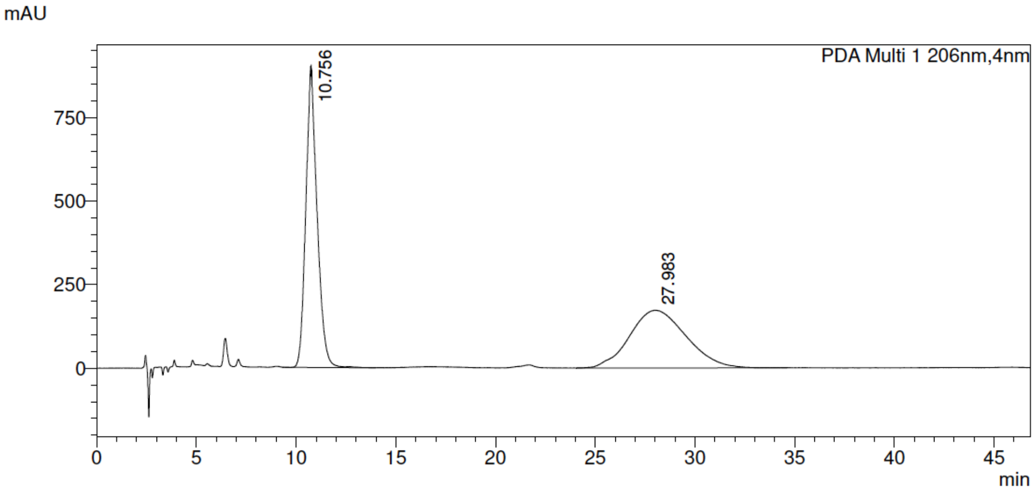


PDA Ch1 206nm

| Peak# | Ret. Time | Area | Height | Area% |
|---|---|---|---|---|
| 1 | 10.756 | 35119358 | 899382 | 50.822 |
| 2 | 27.983 | 33983624 | 172707 | 49.178 |
| Total | | 69102982 | 1072088 | 100.000 |

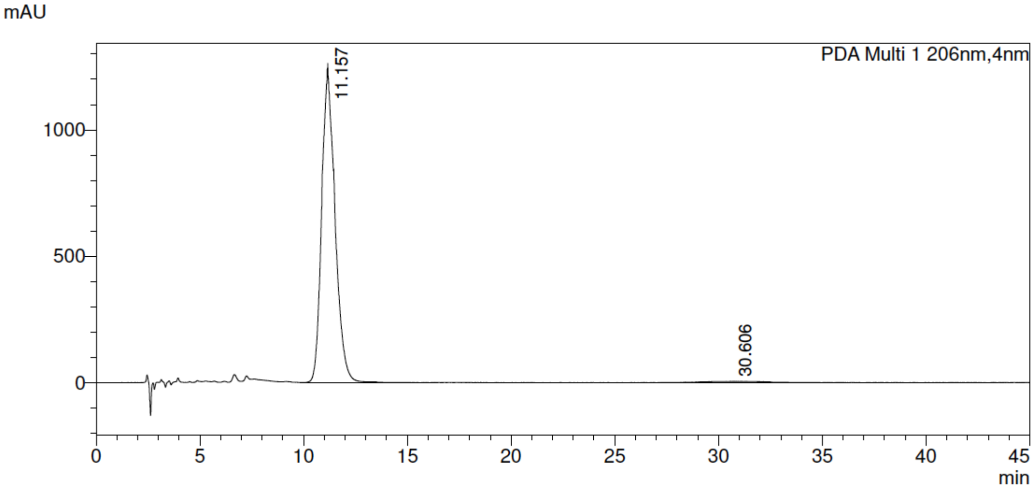


PDA Ch1 206nm

| Peak# | Ret. Time | Area | Height | Area% |
|---|---|---|---|---|
| 1 | 11.157 | 55511726 | 1243949 | 98.175 |
| 2 | 30.606 | 1032044 | 4753 | 1.825 |
| Total | | 56543770 | 1248701 | 100.000 |

**Supplementary Figure 30.** HPLC spectra for racemic and chiral **5n**

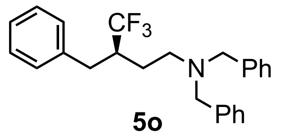

**5o**

25% (95% hexanes, 5% EtOH, 0.2%TFA, 0.1%DEA), 75% hexanes, 0.8 mL/min, CHIRALCEL® OJ-H

er = 97.1 : 2.9

$[\alpha]_D^{23} = -2.22$ ($c$ = 1.71)

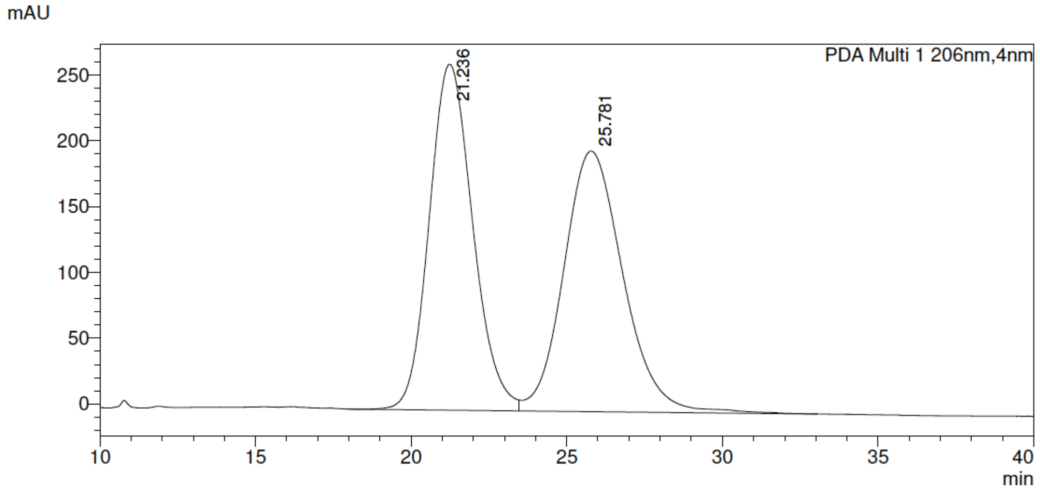


PDA Ch1 206nm

| Peak# | Ret. Time | Area | Height | Conc. | Area% |
|---|---|---|---|---|---|
| 1 | 21.236 | 25592581 | 262863 | 0.000 | 49.351 |
| 2 | 25.781 | 26265245 | 198023 | 0.000 | 50.649 |
| Total | | 51857827 | 460887 | | 100.000 |

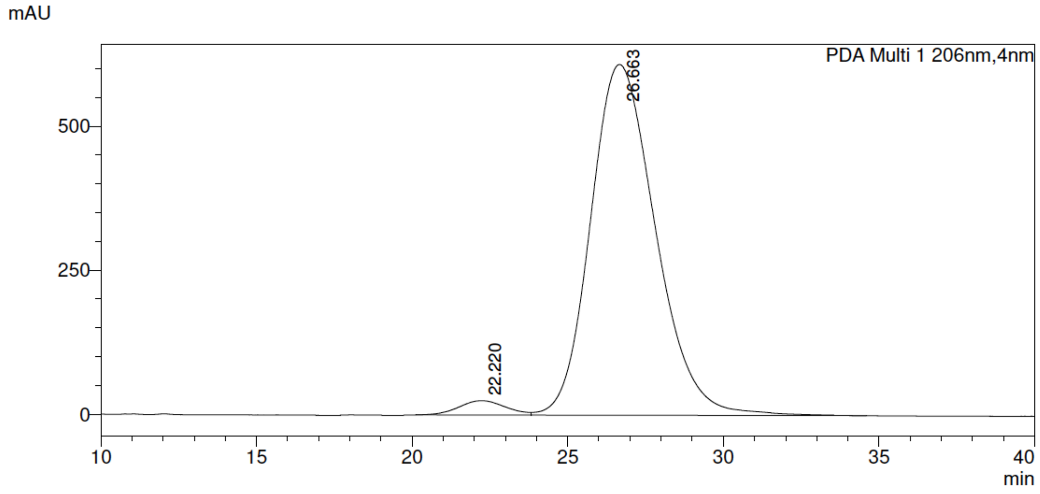


PDA Ch1 206nm

| Peak# | Ret. Time | Area | Height | Area% |
|---|---|---|---|---|
| 1 | 22.220 | 2608624 | 25026 | 2.867 |
| 2 | 26.663 | 88365204 | 608469 | 97.133 |
| Total | | 90973828 | 633496 | 100.000 |

**Supplementary Figure 31.** HPLC spectra for racemic and chiral **5o**

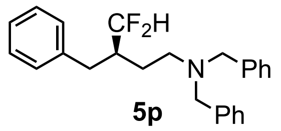


100% (95% hexanes, 5% EtOH, 0.2%TFA, 0.1%DEA), 0.8 mL/min, CHIRALPAK® IB3

er = 97.2 : 2.8

$[\alpha]_D^{23} = 0.96$ ($c$ = 1.68)

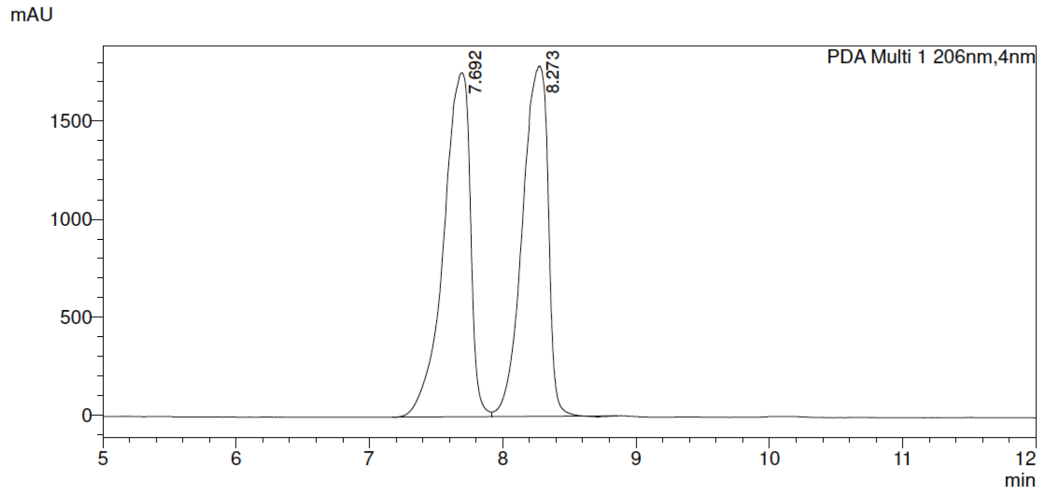


PDA Ch1 206nm

| Peak# | Ret. Time | Area | Height | Area% |
|---|---|---|---|---|
| 1 | 7.692 | 24454936 | 1752397 | 50.618 |
| 2 | 8.273 | 23857665 | 1782768 | 49.382 |
| Total | | 48312601 | 3535164 | 100.000 |

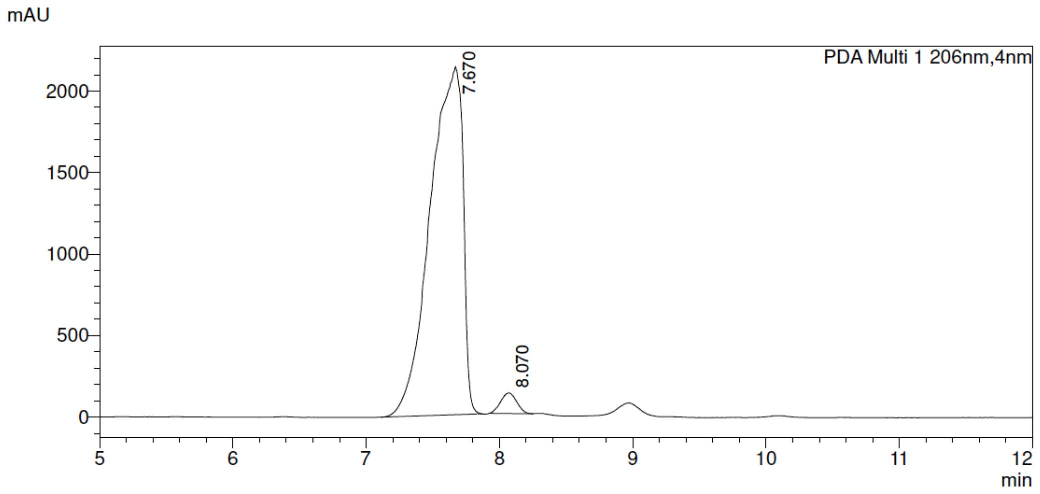


PDA Ch1 206nm

| Peak# | Ret. Time | Area | Height | Area% |
|---|---|---|---|---|
| 1 | 7.670 | 36625215 | 2132341 | 97.209 |
| 2 | 8.070 | 1051701 | 125602 | 2.791 |
| Total | | 37676917 | 2257942 | 100.000 |

**Supplementary Figure 32.** HPLC spectra for racemic and chiral **5p**

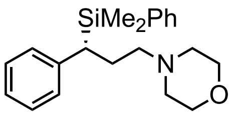


**5q**

50% (95% hexanes, 5% EtOH, 0.2%TFA, 0.1%DEA), 50% hexanes, 0.8 mL/min, CHIRALPAK® IA3

er = 98.8 : 1.2

$[\alpha]_D^{23}$ = 8.10 ($c$ = 1.98)

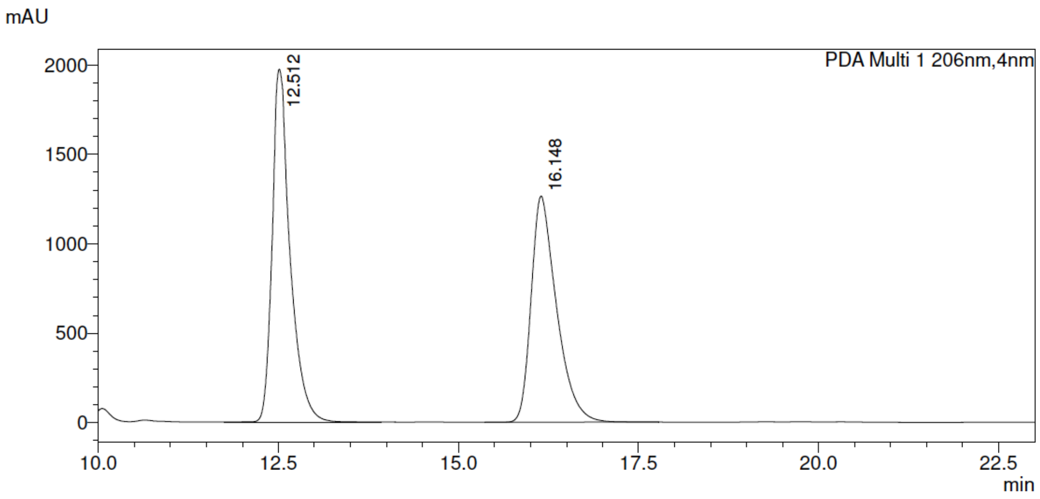


PDA Ch1 206nm

| Peak# | Ret. Time | Area | Height | Area% |
|---|---|---|---|---|
| 1 | 12.512 | 34134234 | 1972601 | 52.320 |
| 2 | 16.148 | 31106916 | 1265412 | 47.680 |
| Total | | 65241150 | 3238013 | 100.000 |

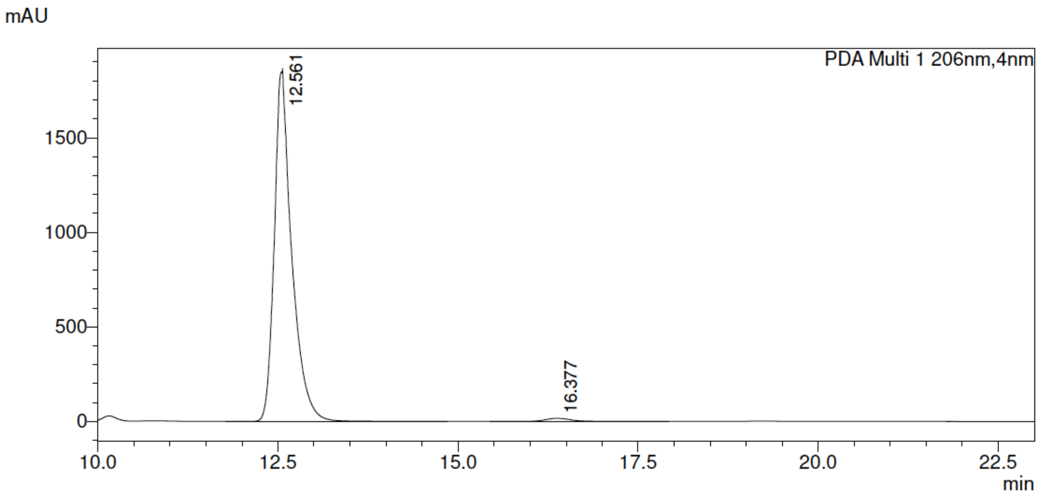


PDA Ch1 206nm

| Peak# | Ret. Time | Area | Height | Area% |
|---|---|---|---|---|
| 1 | 12.561 | 31258653 | 1846980 | 98.776 |
| 2 | 16.377 | 387503 | 16316 | 1.224 |
| Total | | 31646156 | 1863296 | 100.000 |

**Supplementary Figure 33.** HPLC spectra for racemic and chiral **5q**

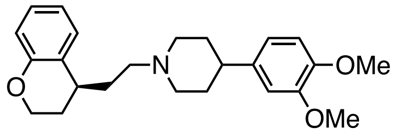


***Terikalant***

100% (95% hexanes, 5% EtOH, 0.2%TFA, 0.1%DEA), 0.8 mL/min, CHIRALPAK® IB3

er = 96.7 : 3.3

$[\alpha]_D^{23} = -8.85$ ($c$ = 2.68)

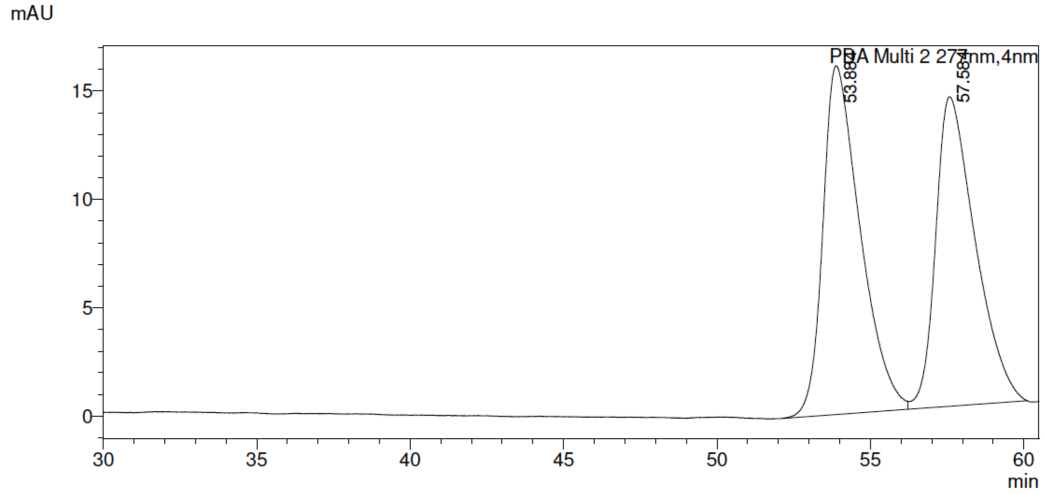


PDA Ch2 277nm

| Peak# | Ret. Time | Area | Height | Conc. | Unit |
|---|---|---|---|---|---|
| 1 | 53.884 | 1362921 | 16087 | 51.561 | |
| 2 | 57.584 | 1280383 | 14280 | 48.439 | |
| Total | | 2643304 | 30367 | | |

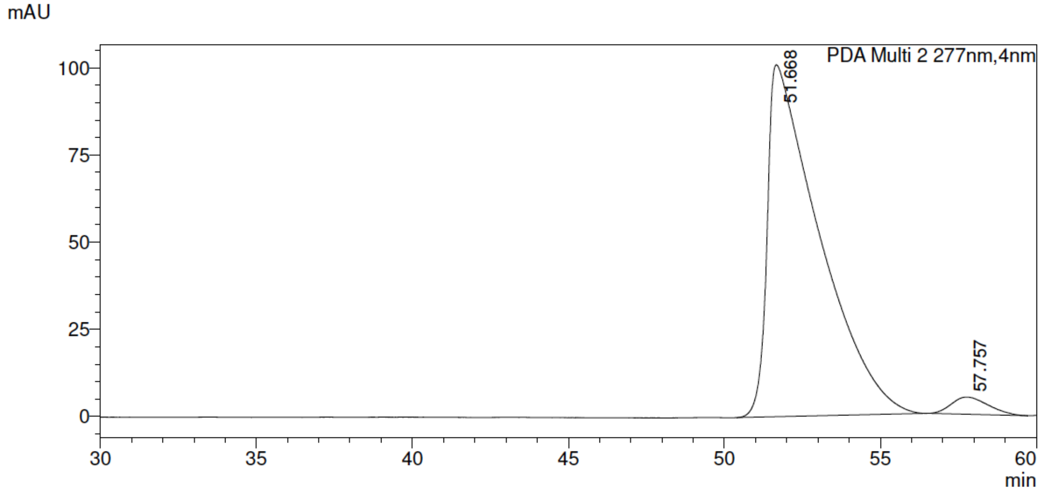


PDA Ch2 277nm

| Peak# | Ret. Time | Area | Height | Area% |
|---|---|---|---|---|
| 1 | 51.668 | 11746301 | 100998 | 96.657 |
| 2 | 57.757 | 406200 | 4951 | 3.343 |
| Total | | 12152501 | 105950 | 100.000 |

**Supplementary Figure 34.** HPLC spectra for racemic and chiral **Terikalant**

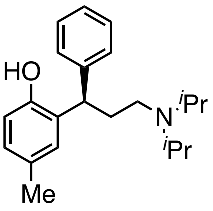


***(R)*-Tolterodine**

50% (95% hexanes, 5% EtOH, 0.2%TFA, 0.1%DEA), 50% hexanes, 1.0 mL/min, CHIRALCEL® OJ-H

er = 96.0 : 4.0

$[\alpha]_D^{23}$ = 114.94 ($c$ = 2.99)

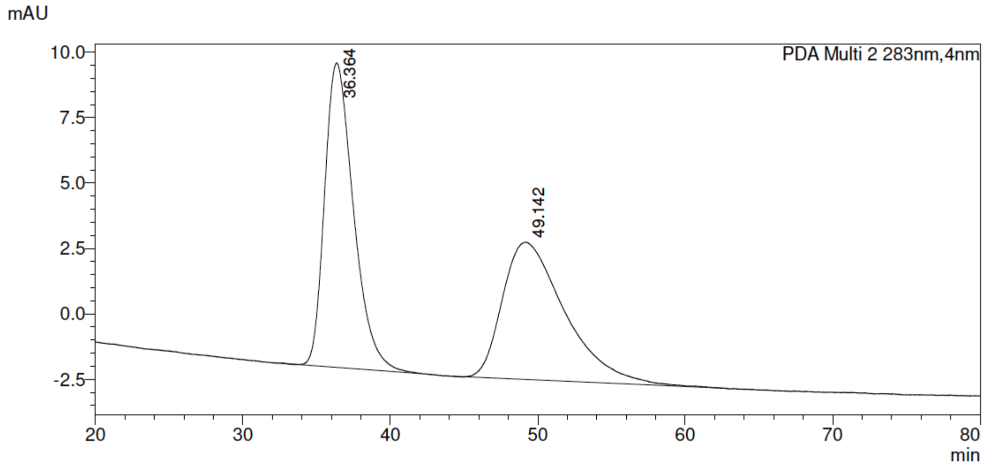


PDA Ch2 283nm

| Peak# | Ret. Time | Area | Height | Area% |
|---|---|---|---|---|
| 1 | 36.364 | 1589118 | 11634 | 50.698 |
| 2 | 49.142 | 1545382 | 5250 | 49.302 |
| Total | | 3134500 | 16883 | 100.000 |

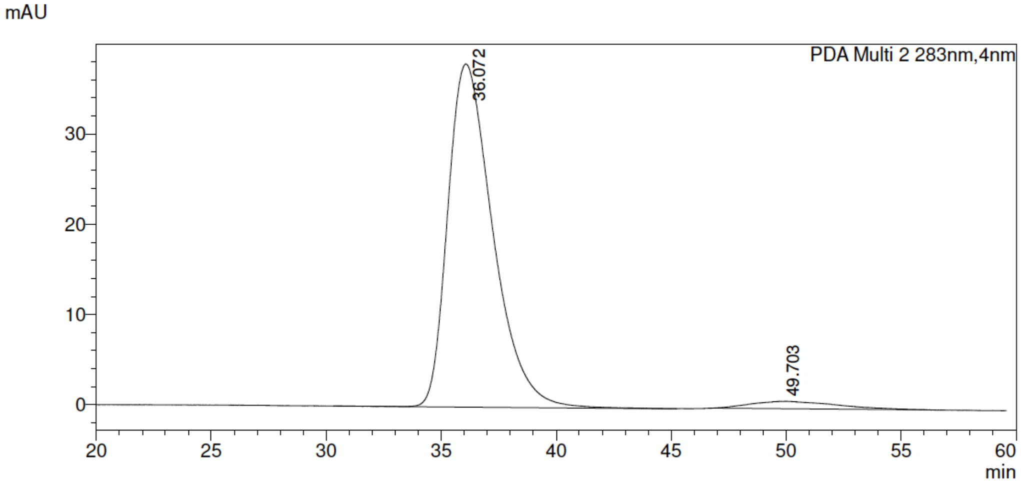


PDA Ch2 283nm

| Peak# | Ret. Time | Area | Height | Area% |
|---|---|---|---|---|
| 1 | 36.072 | 5179099 | 38025 | 95.994 |
| 2 | 49.703 | 216152 | 836 | 4.006 |
| Total | | 5395251 | 38861 | 100.000 |

**Supplementary Figure 35.** HPLC spectra for racemic and chiral **Tolterodine**

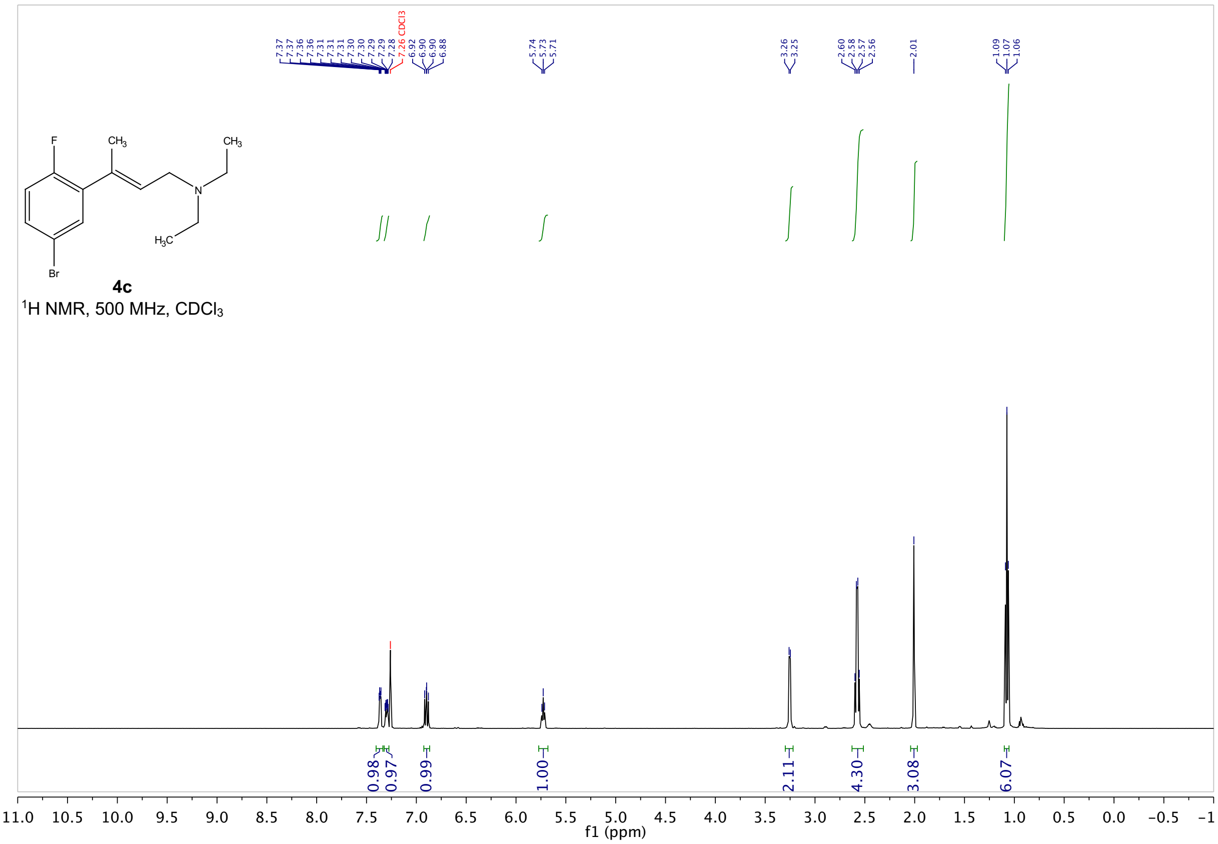


**Supplementary Figure 36.** $^{1}$H NMR spectra of compound **4c**

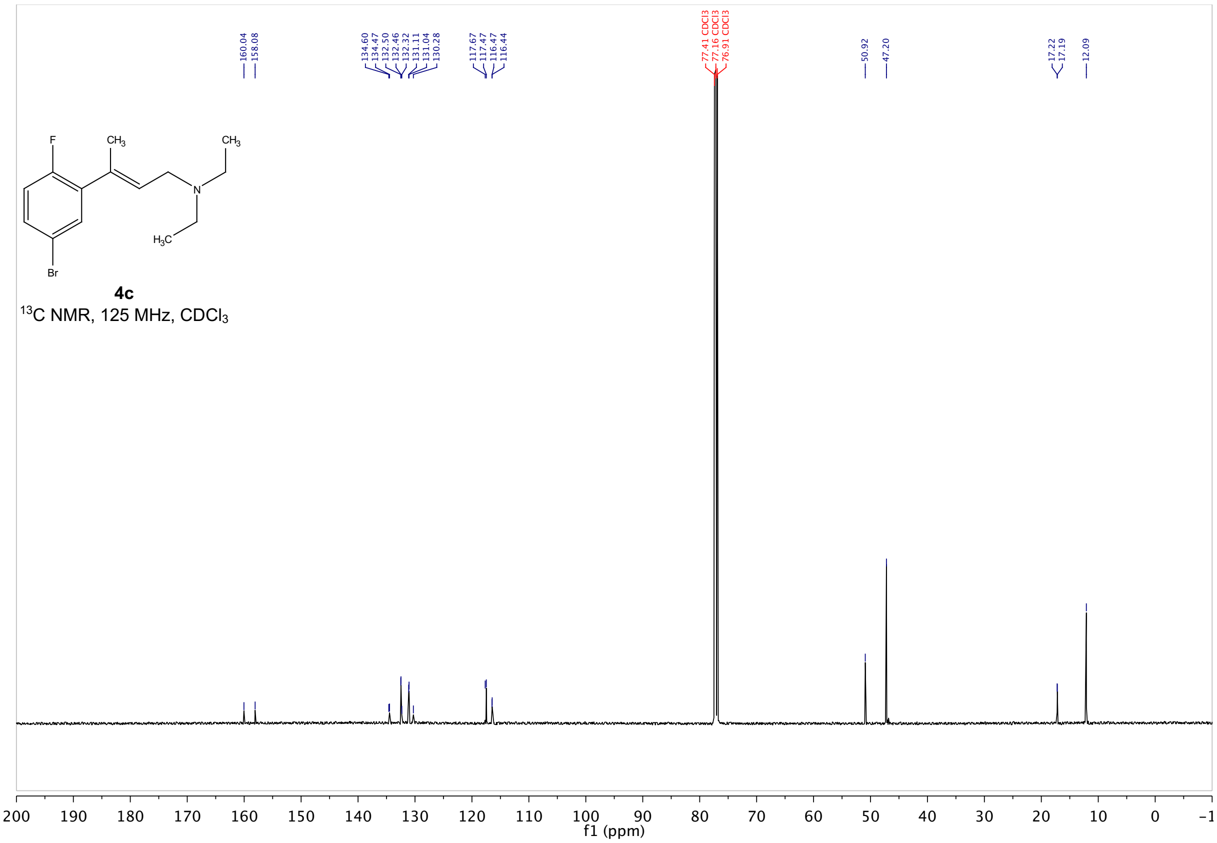


**Supplementary Figure 37.** $^{13}$C NMR spectra of compound **4c**

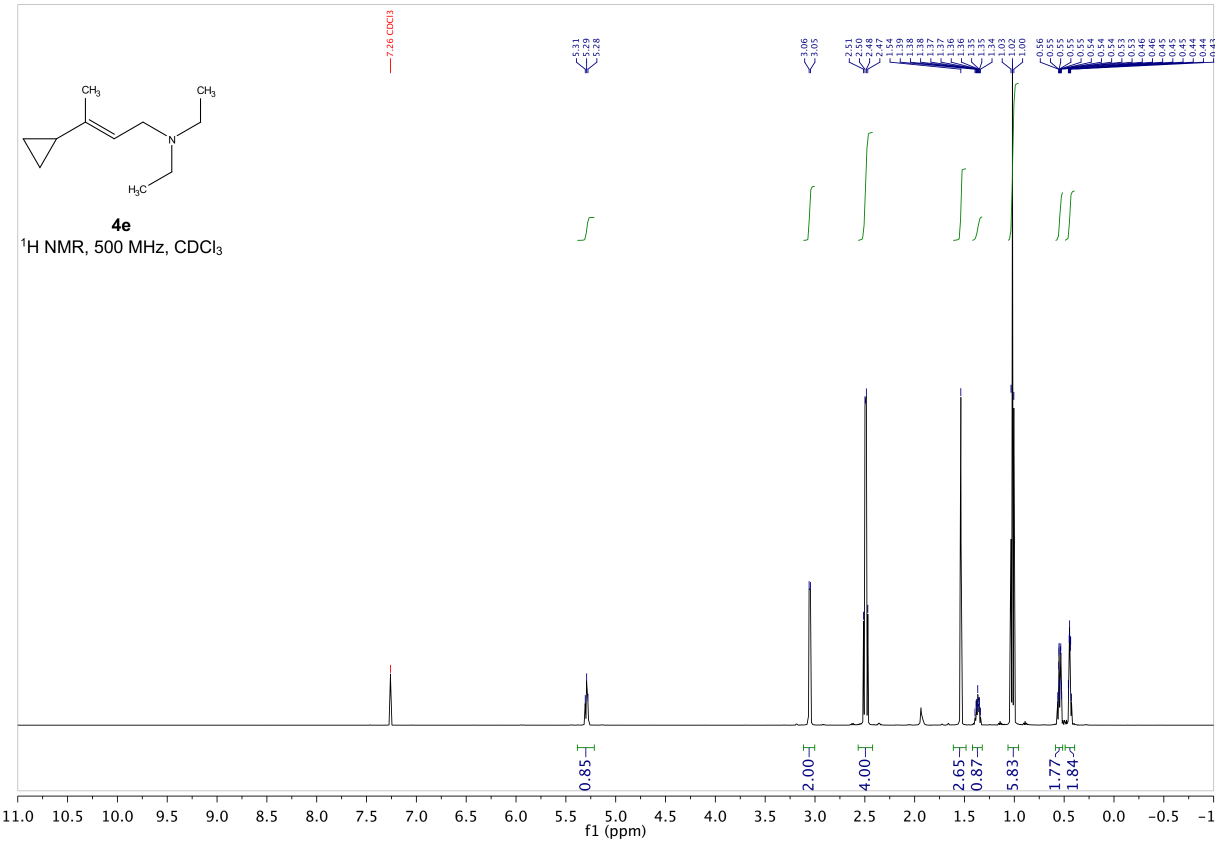


**Supplementary Figure 38.** $^1$H NMR spectra of compound **4e**

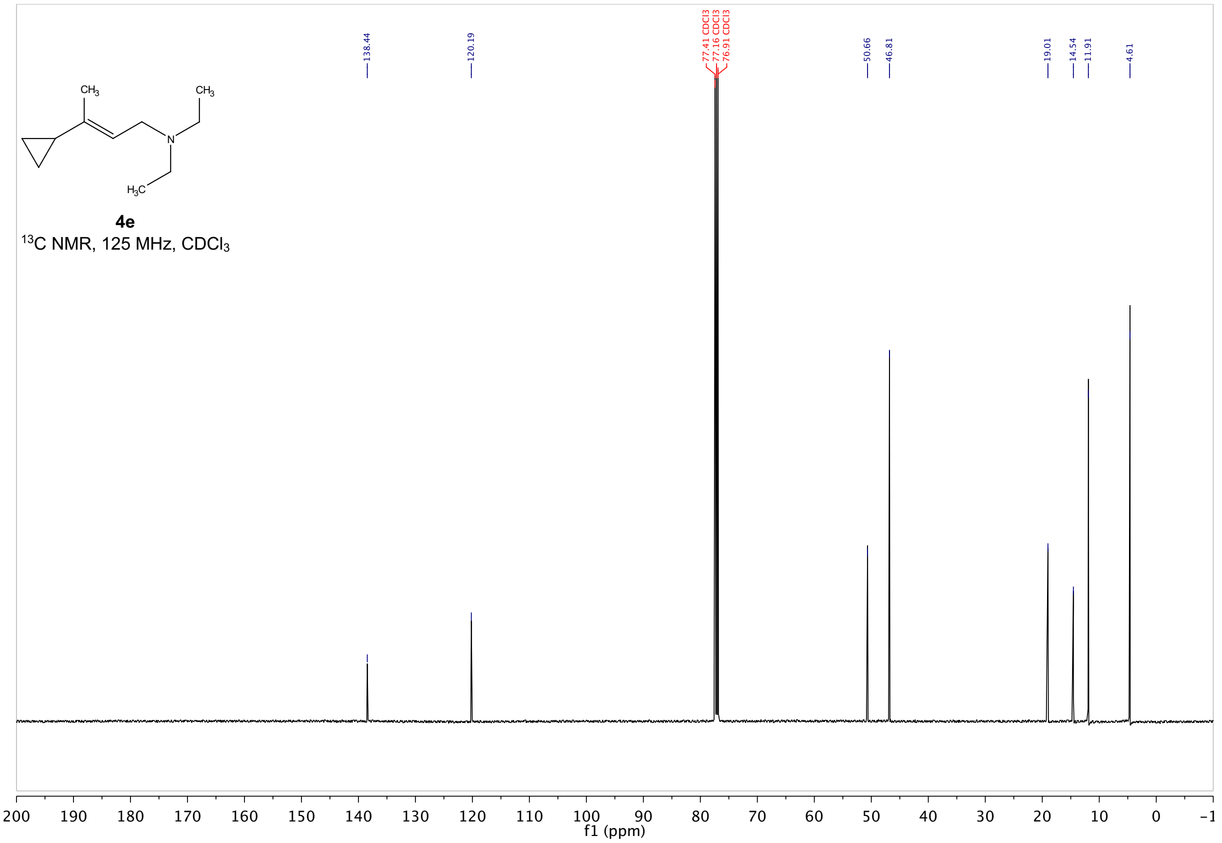


**Supplementary Figure 39.** $^{13}$C NMR spectra of compound **4e**

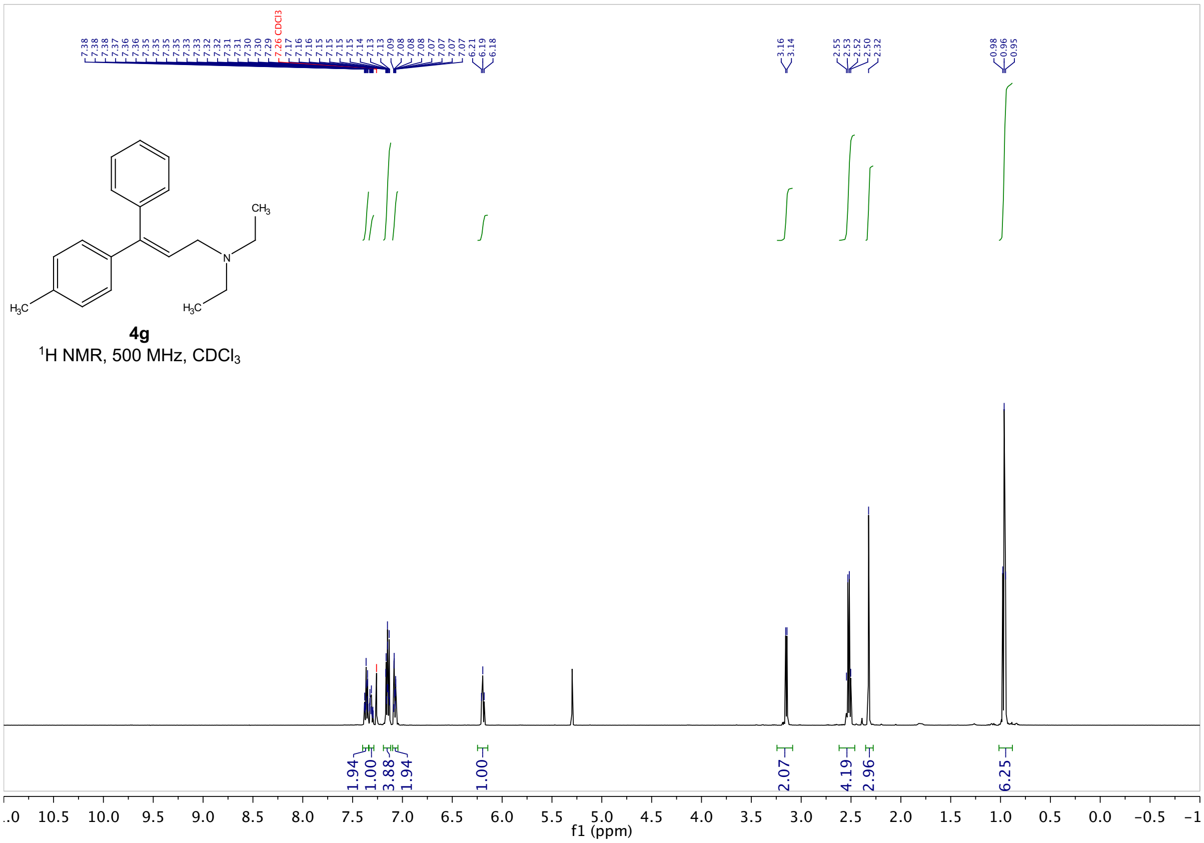


**Supplementary Figure 40.** $^{1}$H NMR spectra of compound **4g**

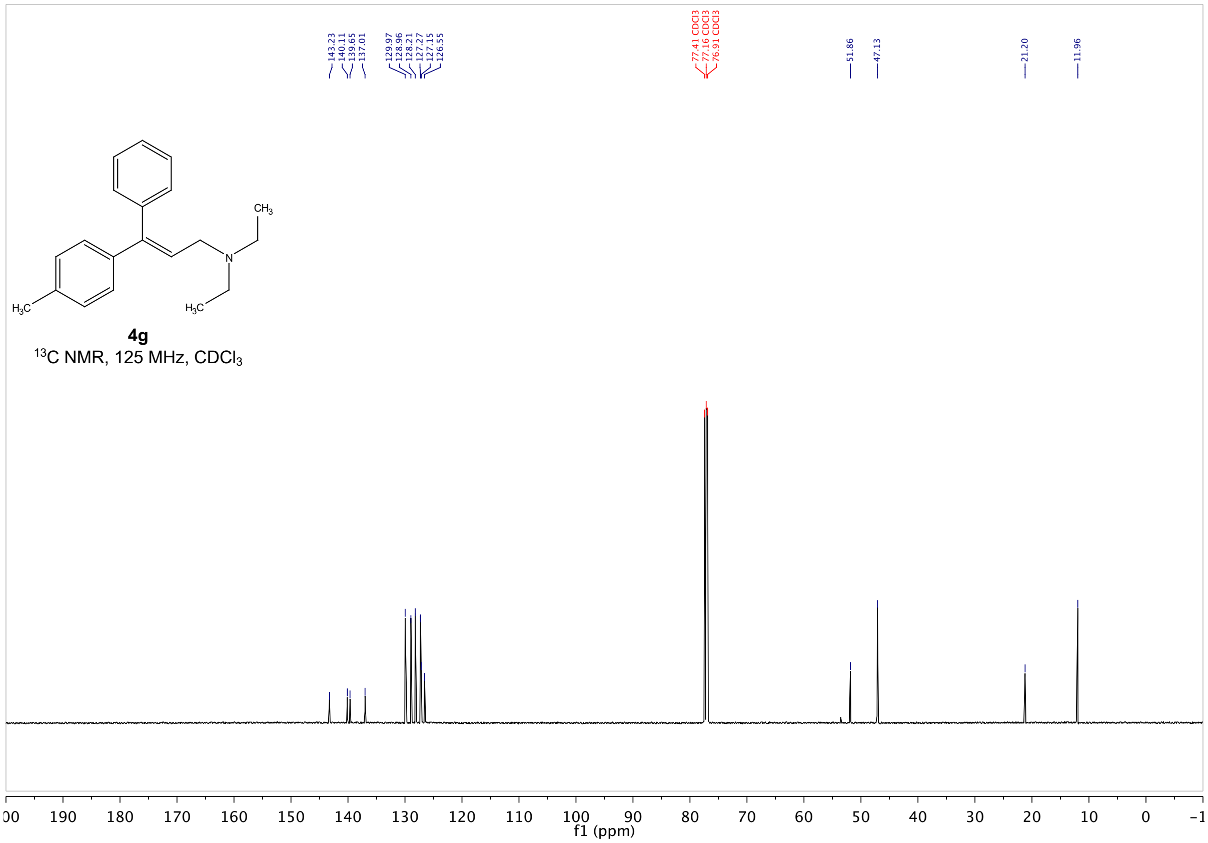


**Supplementary Figure 41.** $^{13}$C NMR spectra of compound **4g**

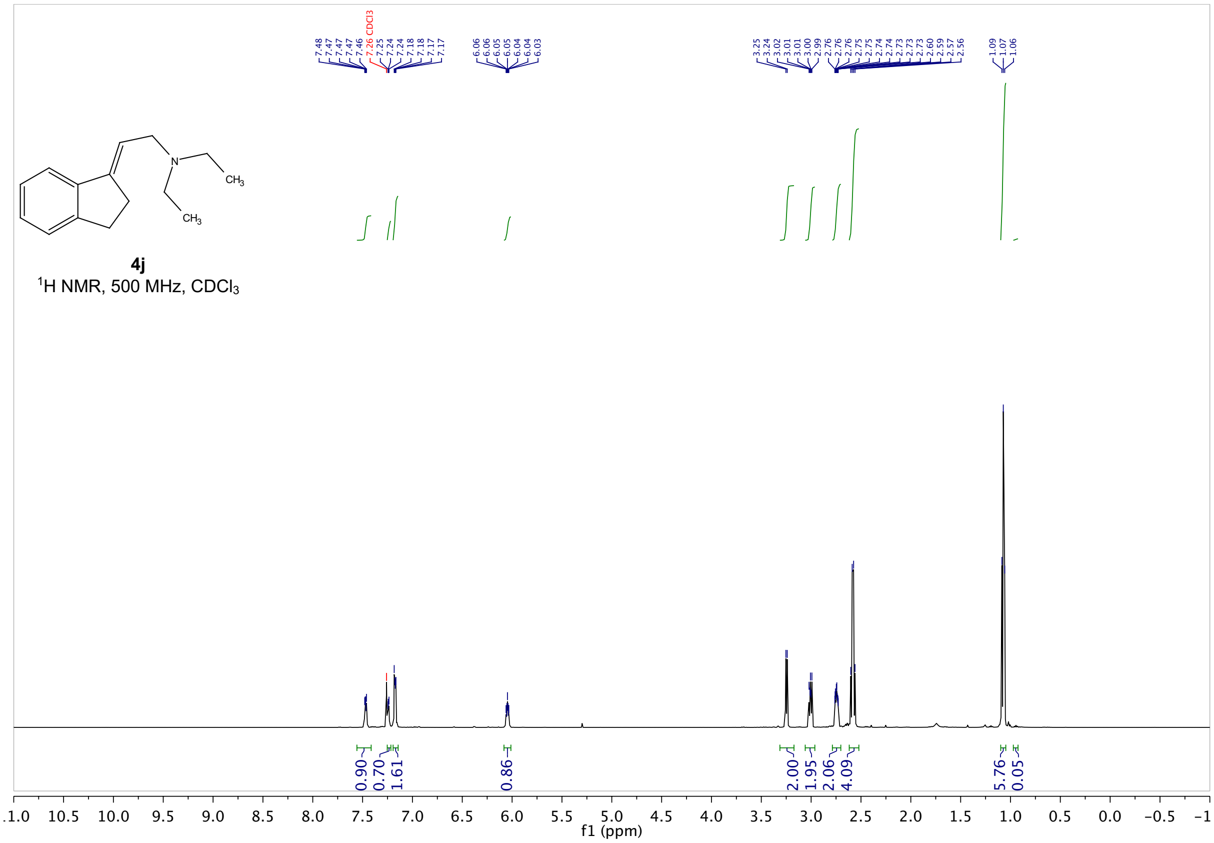


**Supplementary Figure 42.** $^1$H NMR spectra of compound **4j**

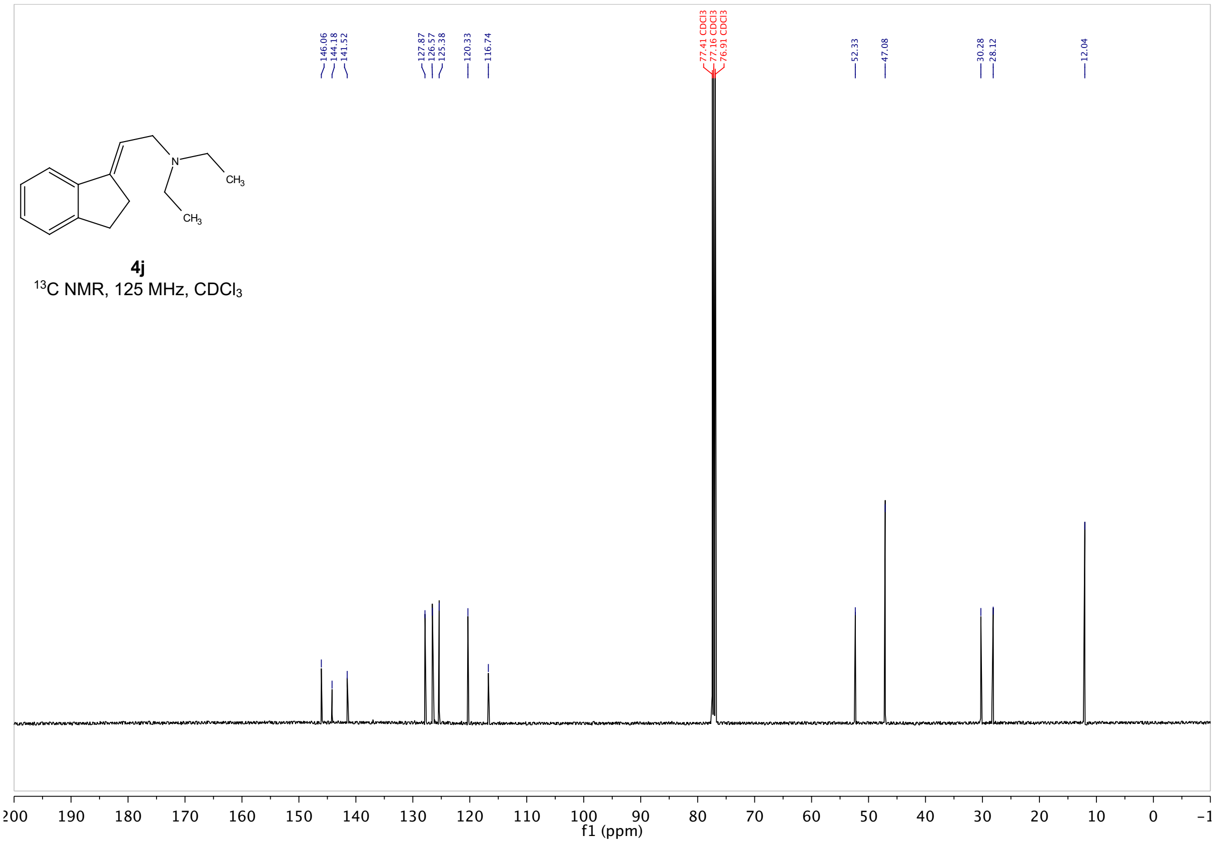


**Supplementary Figure 43.** $^{13}$C NMR spectra of compound **4j**

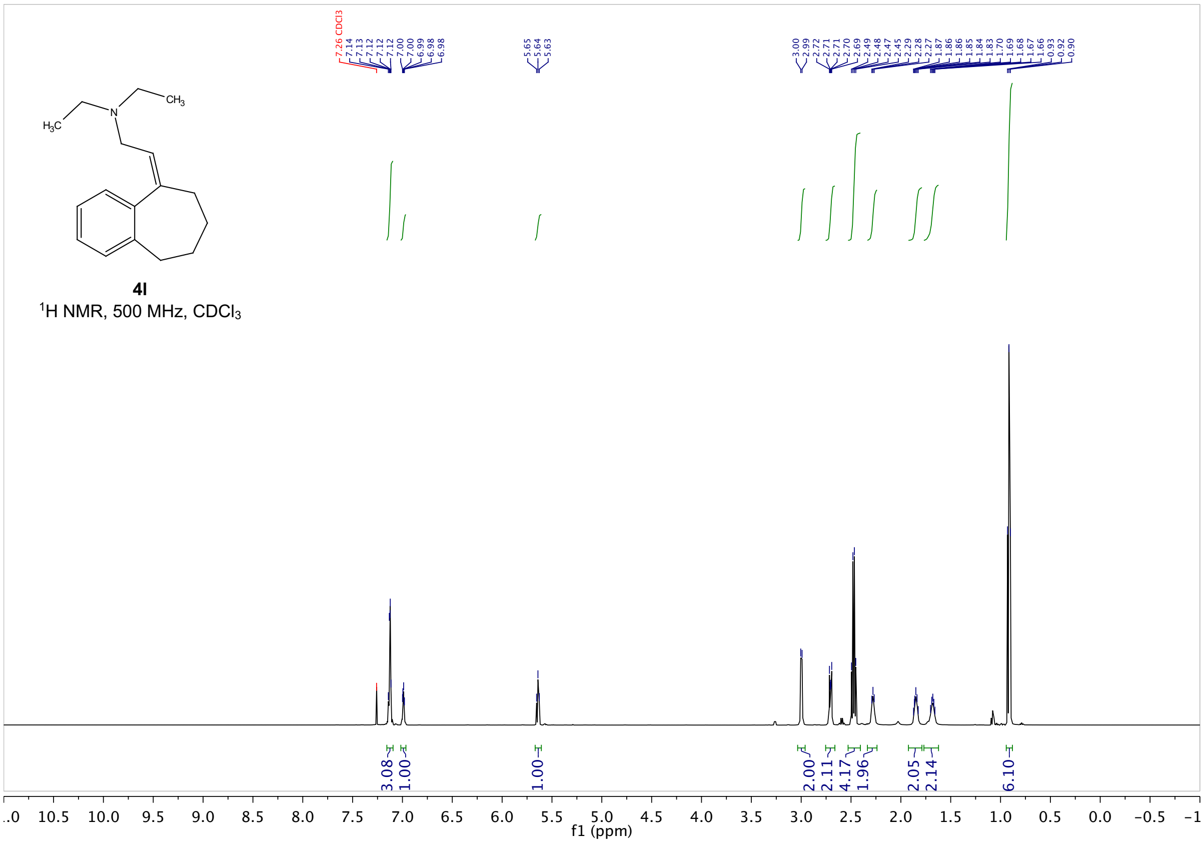


**Supplementary Figure 44.** $^1$H NMR spectra of compound **4l**

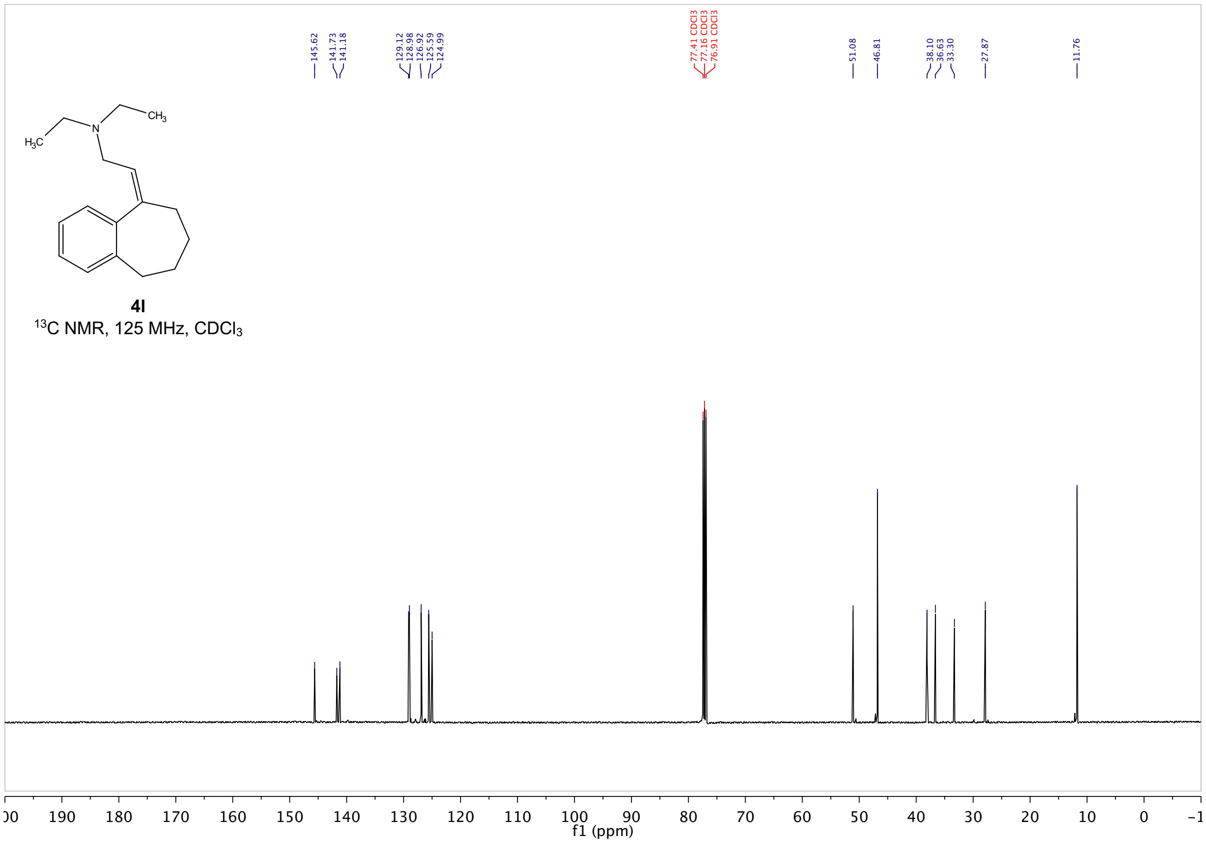


**Supplementary Figure 45.** $^{13}$C NMR spectra of compound **4l**

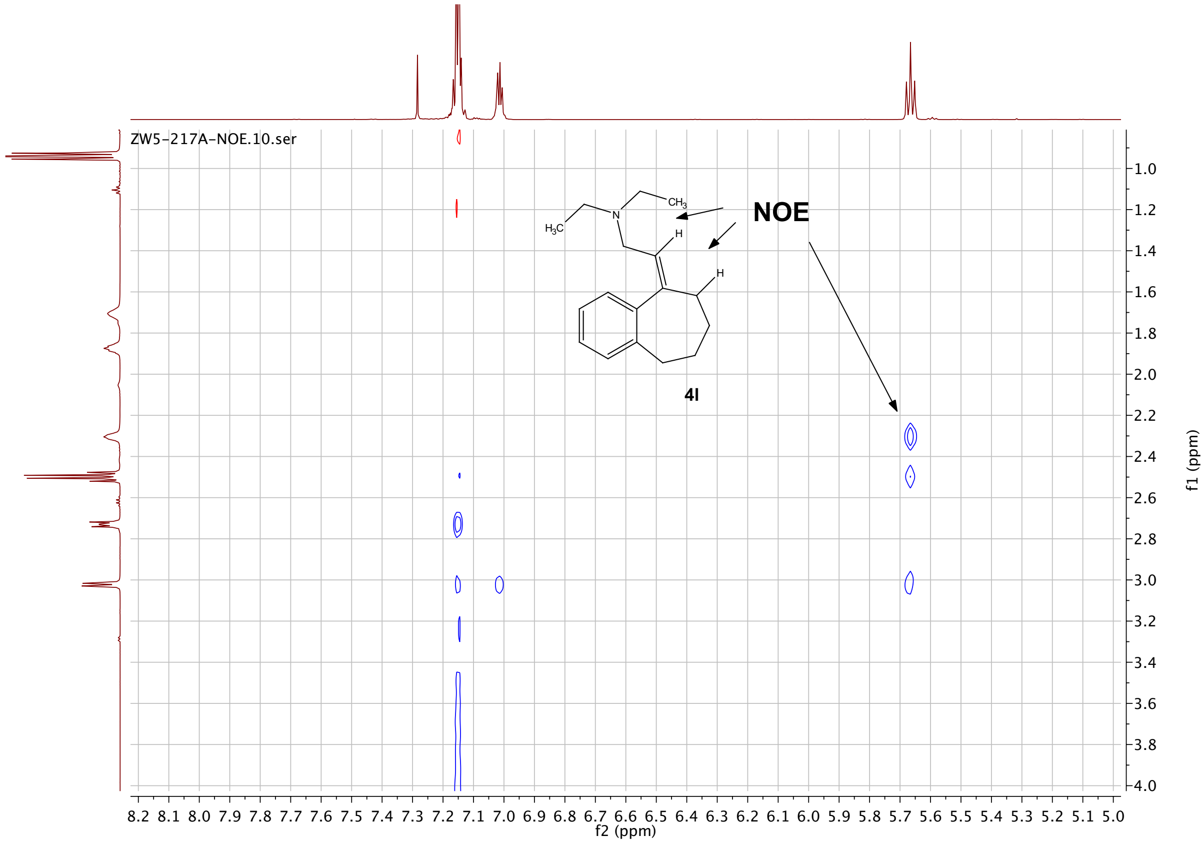


**Supplementary Figure 46.** NOE spectra of compound **4l**

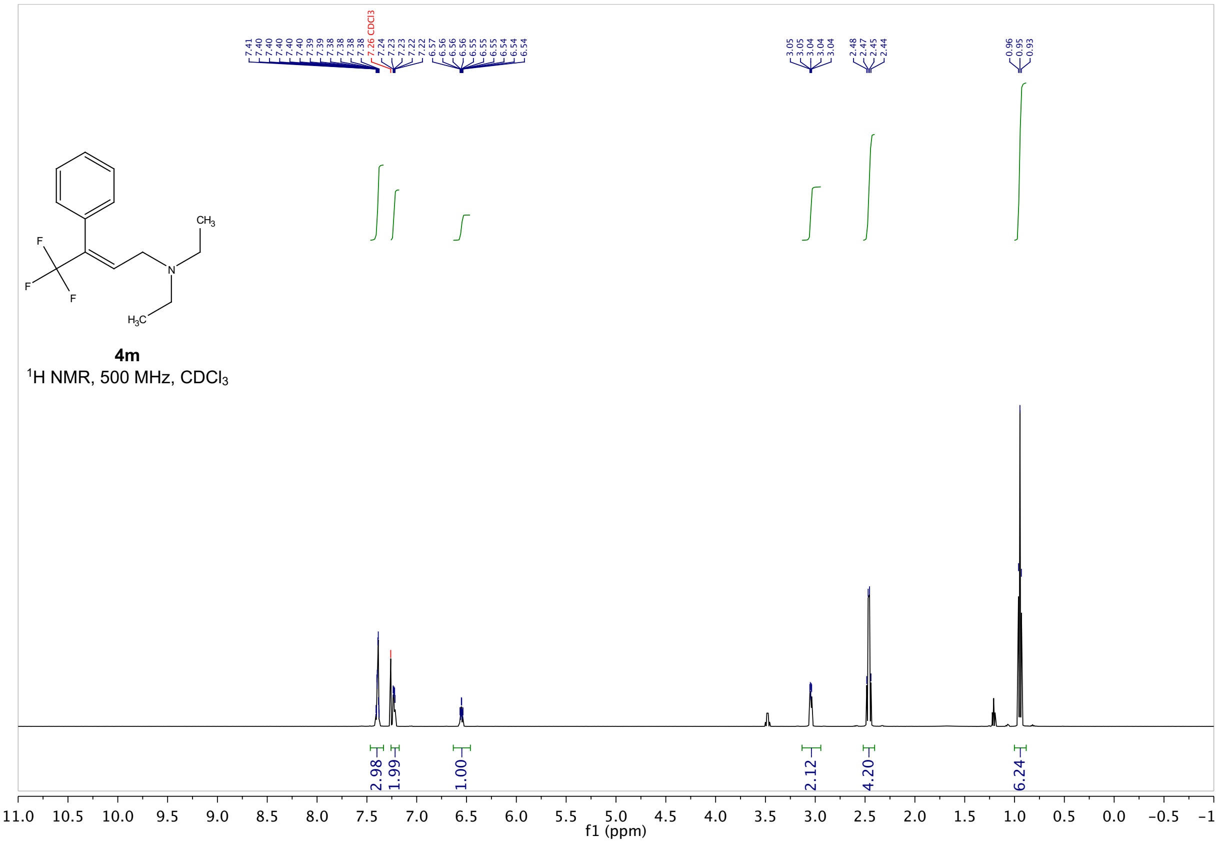


**Supplementary Figure 47.** ¹H NMR spectra of compound **4m**

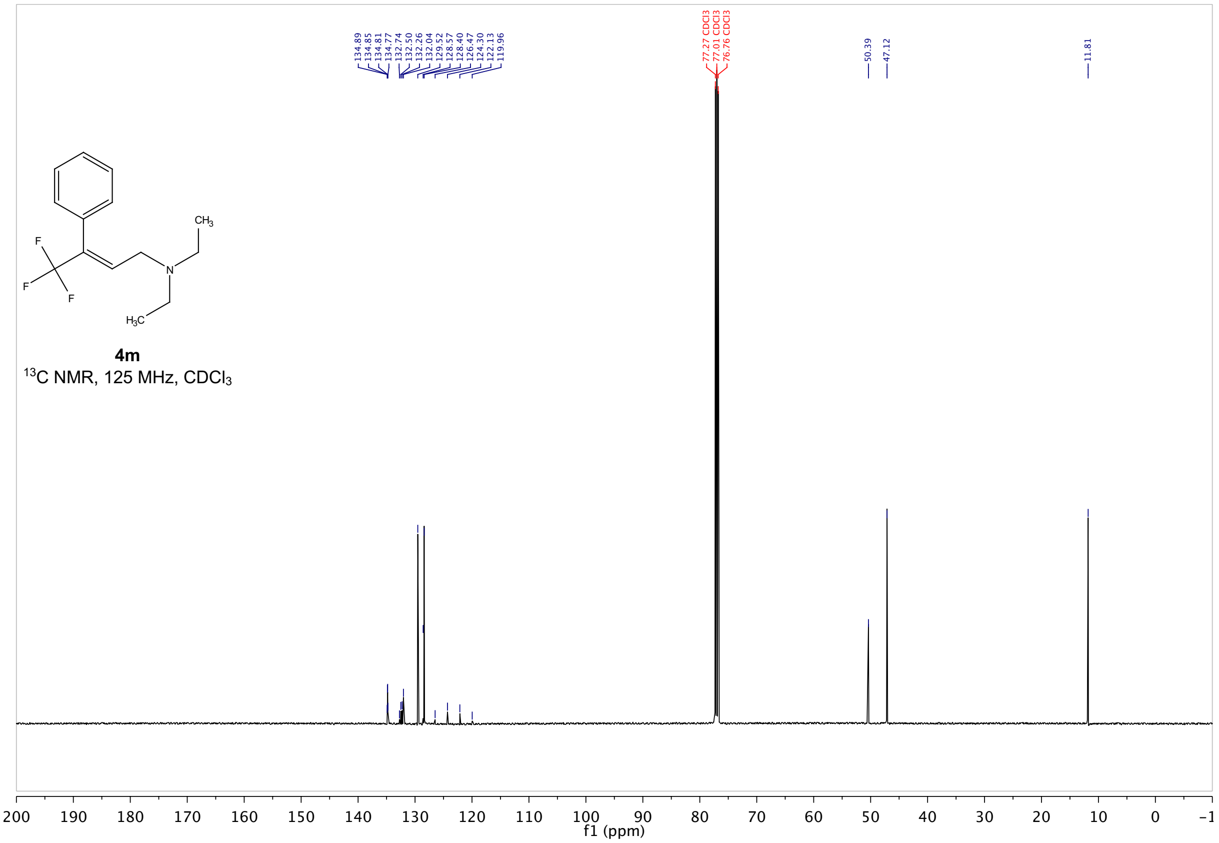


**Supplementary Figure 48.** $^{13}C$ NMR spectra of compound **4m**

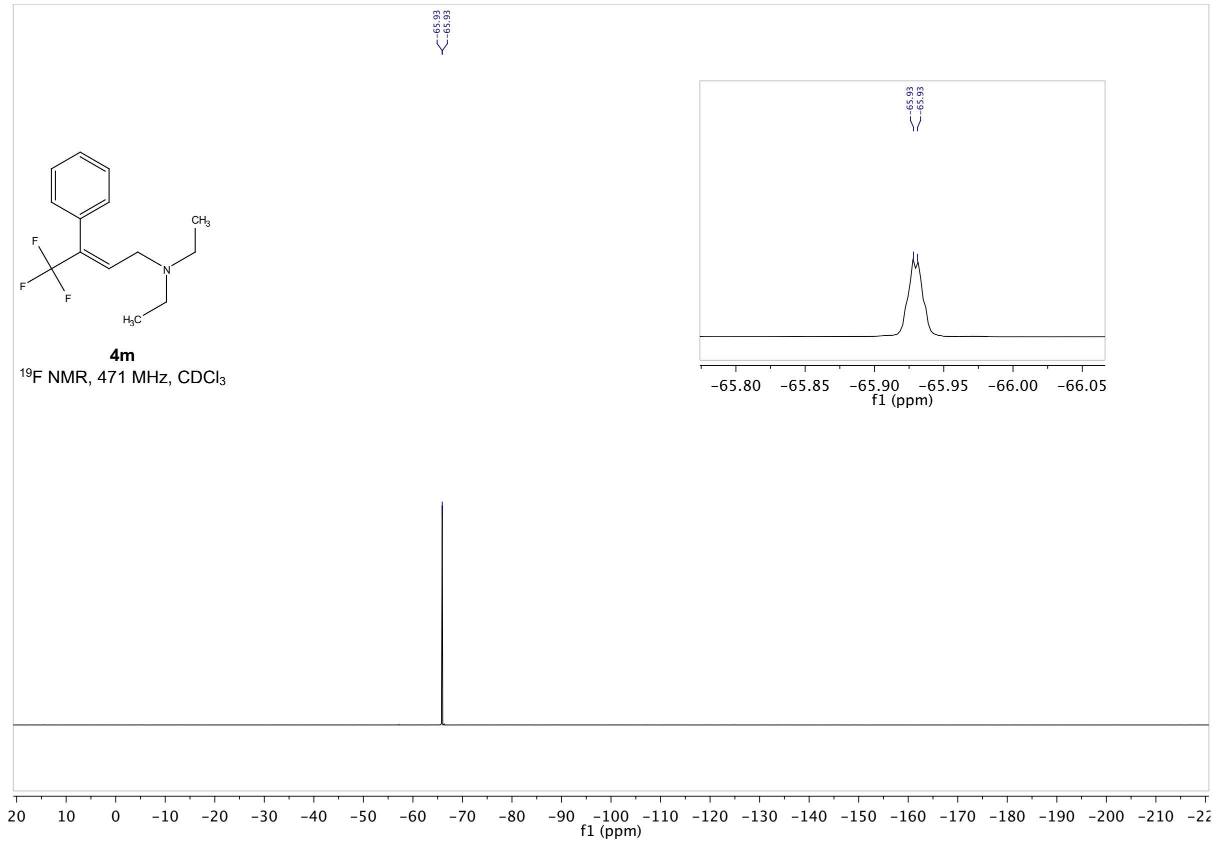


**Supplementary Figure 49.** $^{19}F$ NMR spectra of compound **4m**

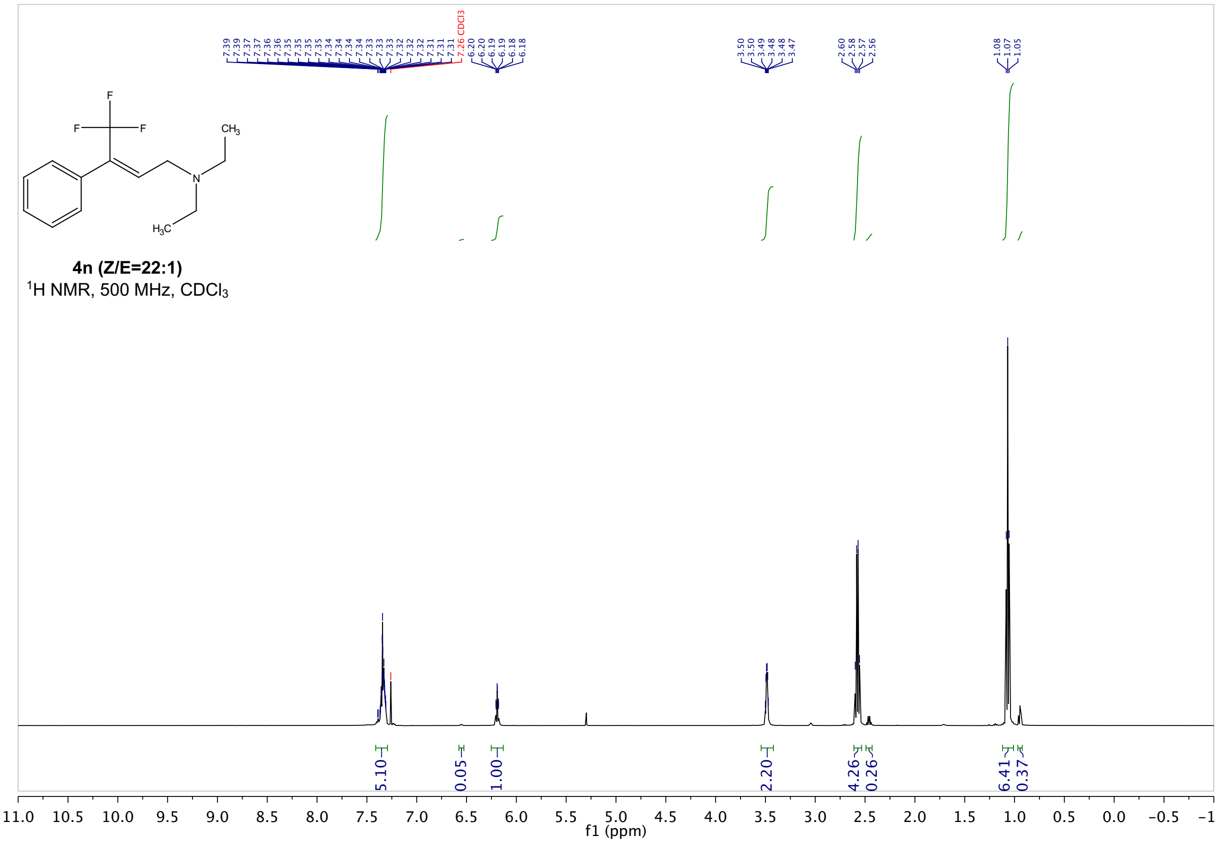


**Supplementary Figure 50.** $^1$H NMR spectra of compound **4n**

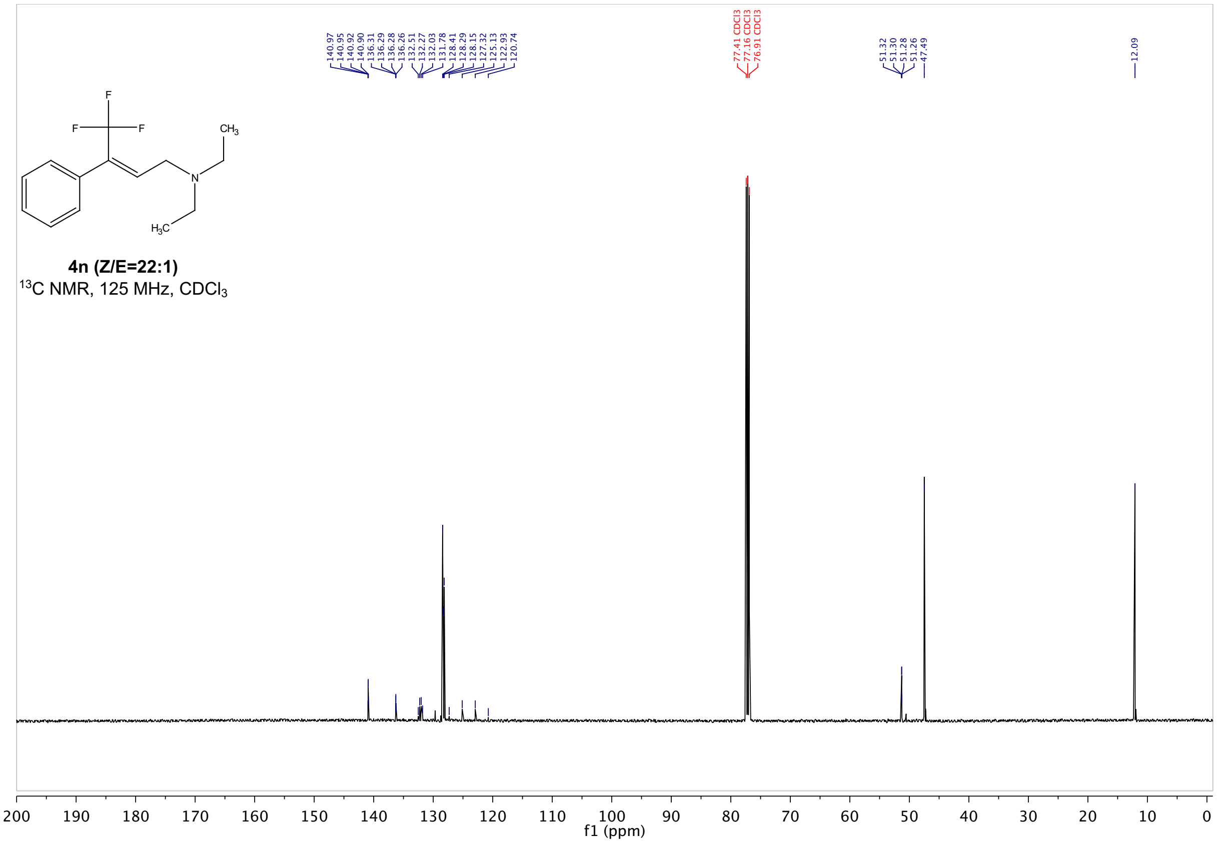


**Supplementary Figure 51.** $^{13}$C NMR spectra of compound **4n**

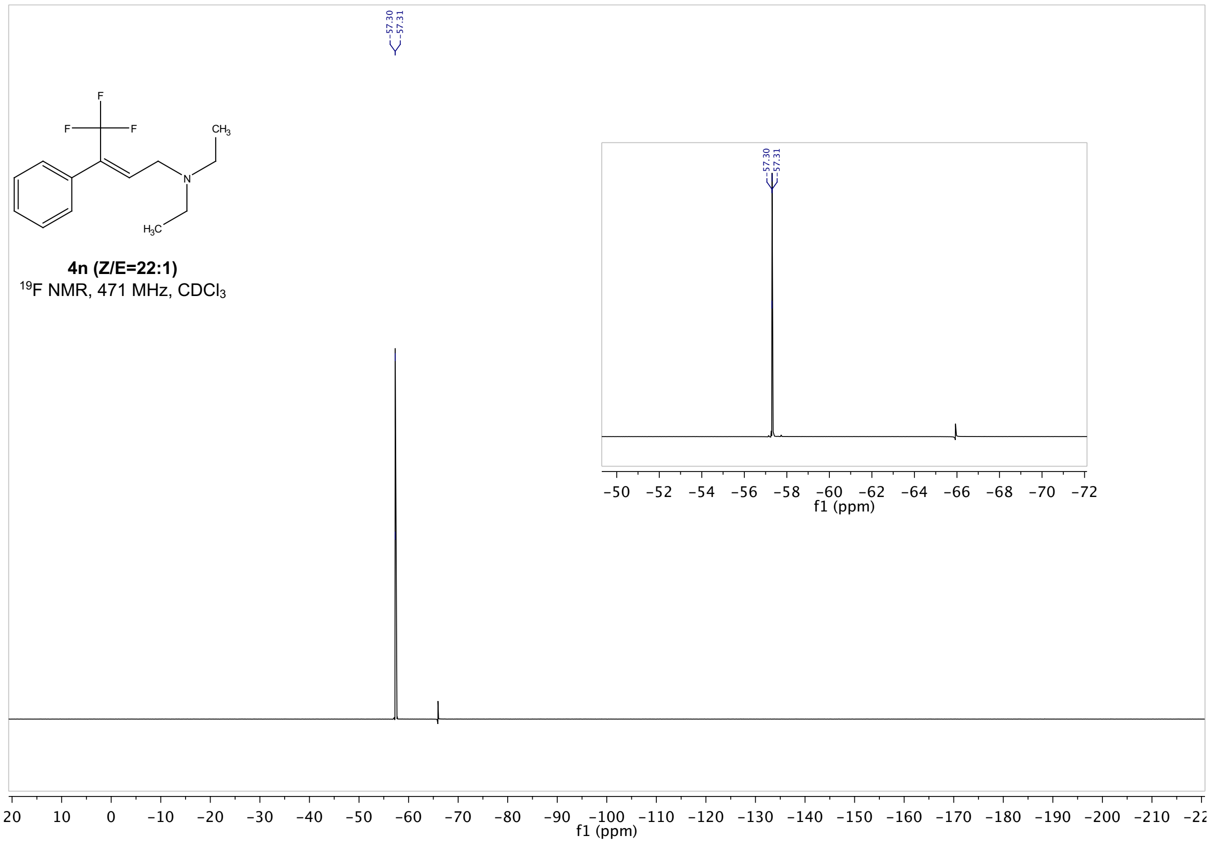


**Supplementary Figure 52.** $^{19}F$ NMR spectra of compound **4n**

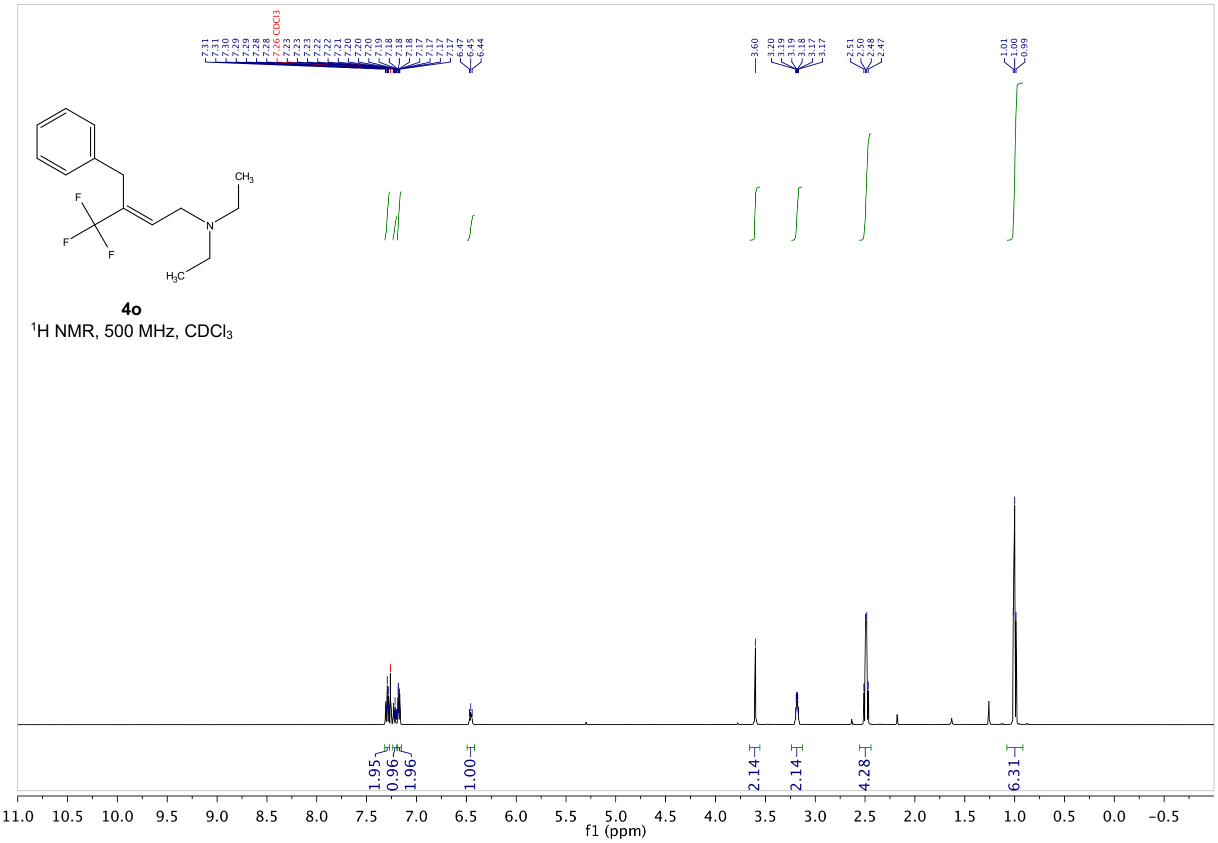


**Supplementary Figure 53.** $^{1}H$ NMR spectra of compound **4o**

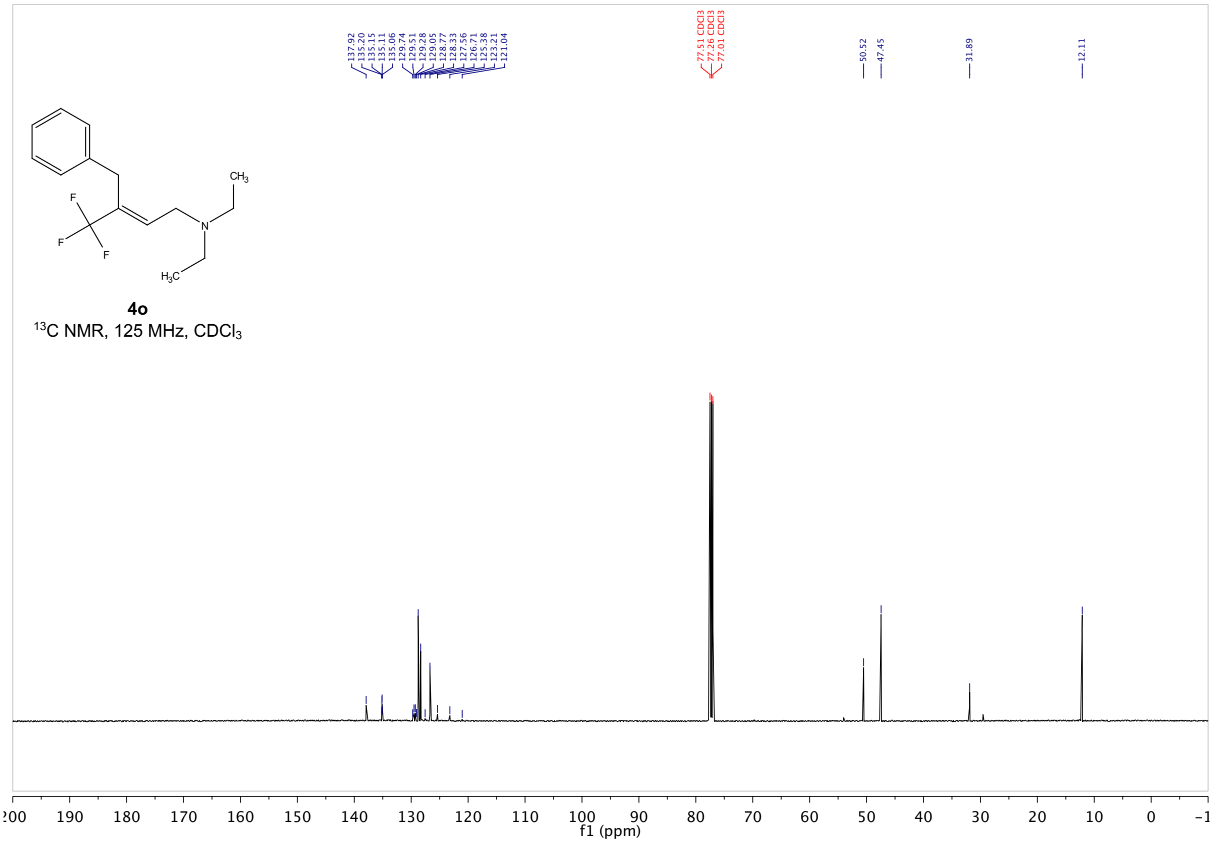


**Supplementary Figure 54.** $^{13}$C NMR spectra of compound **4o**

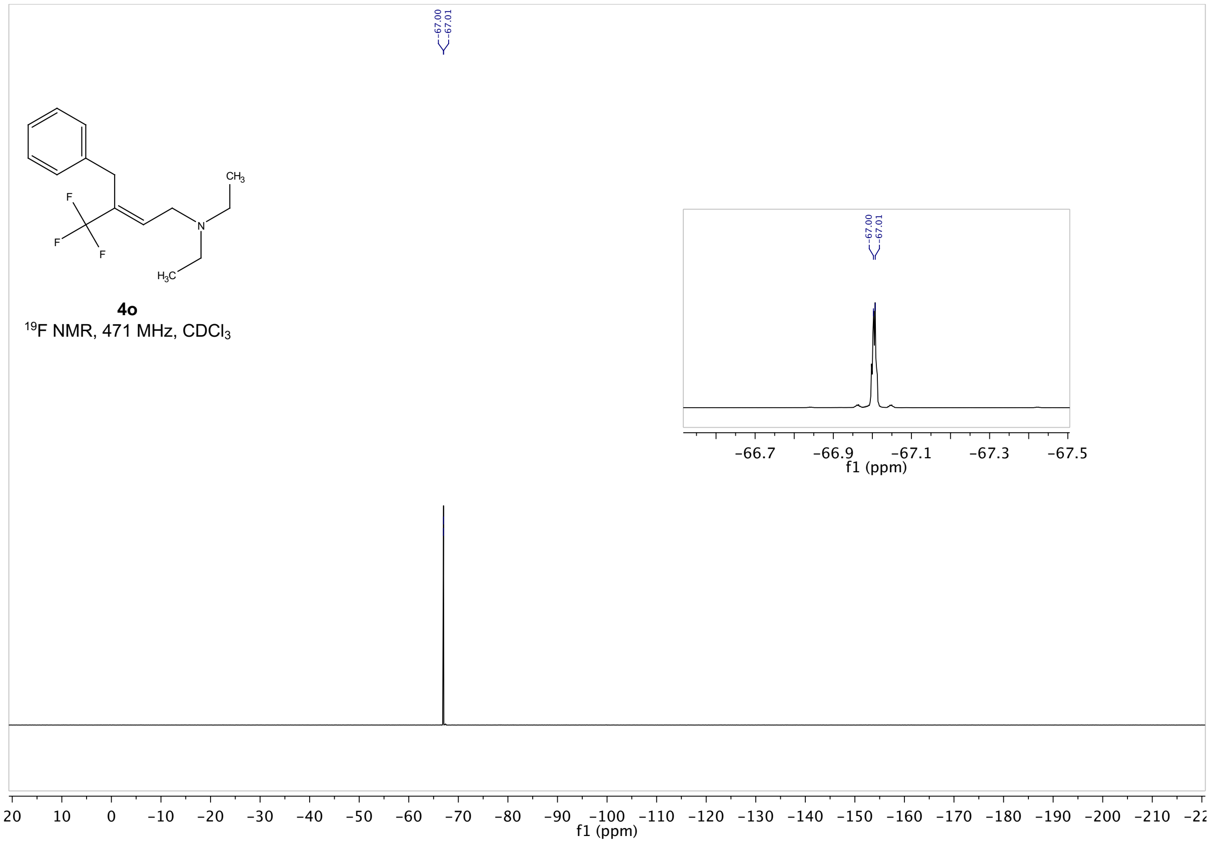


**Supplementary Figure 55.** $^{19}$F NMR spectra of compound **4o**

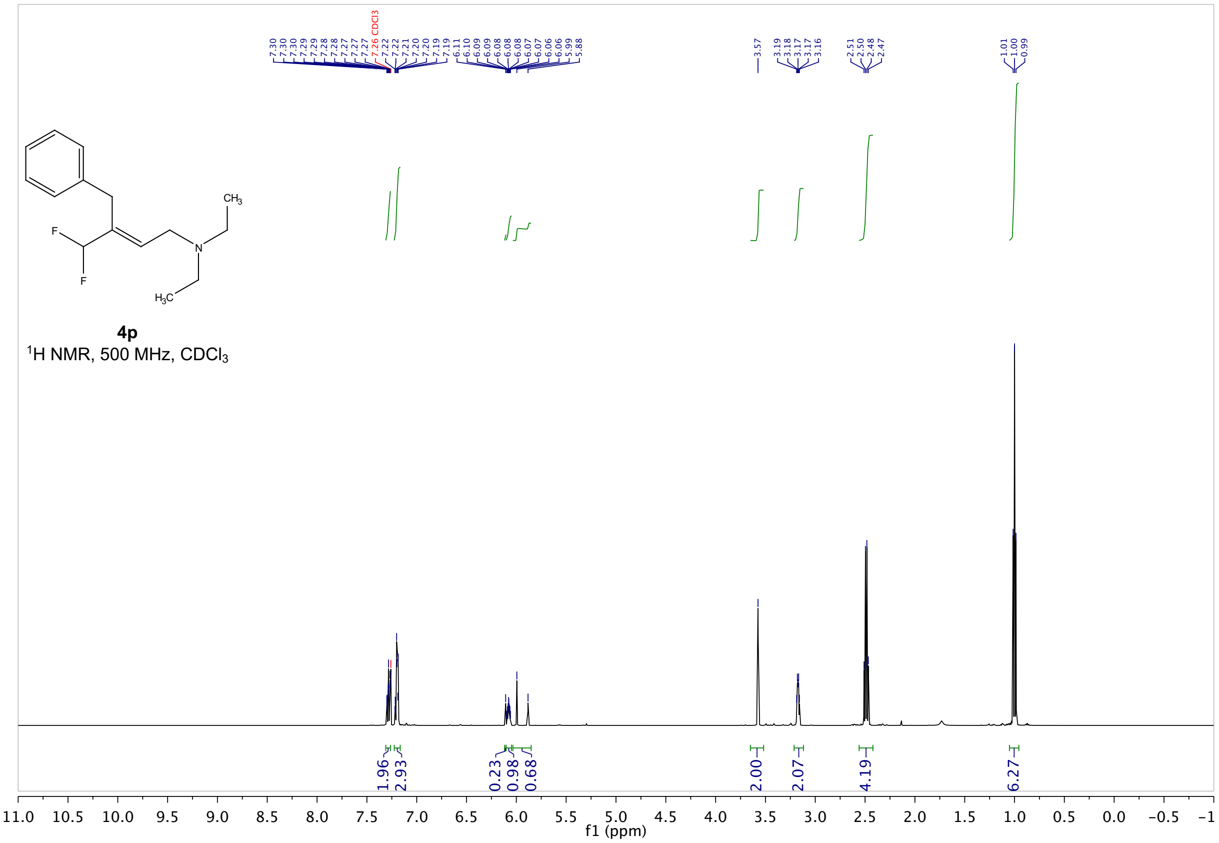


**Supplementary Figure 56.** $^1$H NMR spectra of compound **4p**

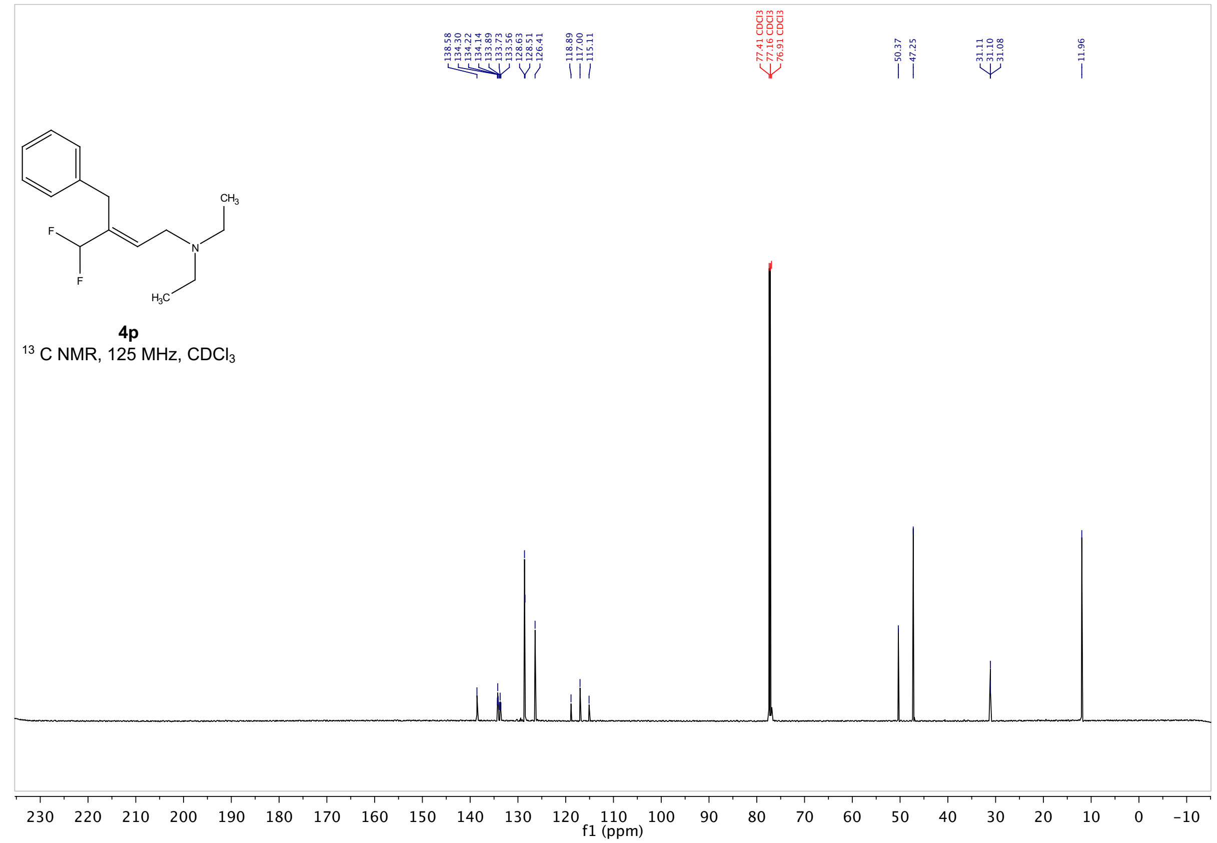


**Supplementary Figure 57.** $^{13}$C NMR spectra of compound **4p**

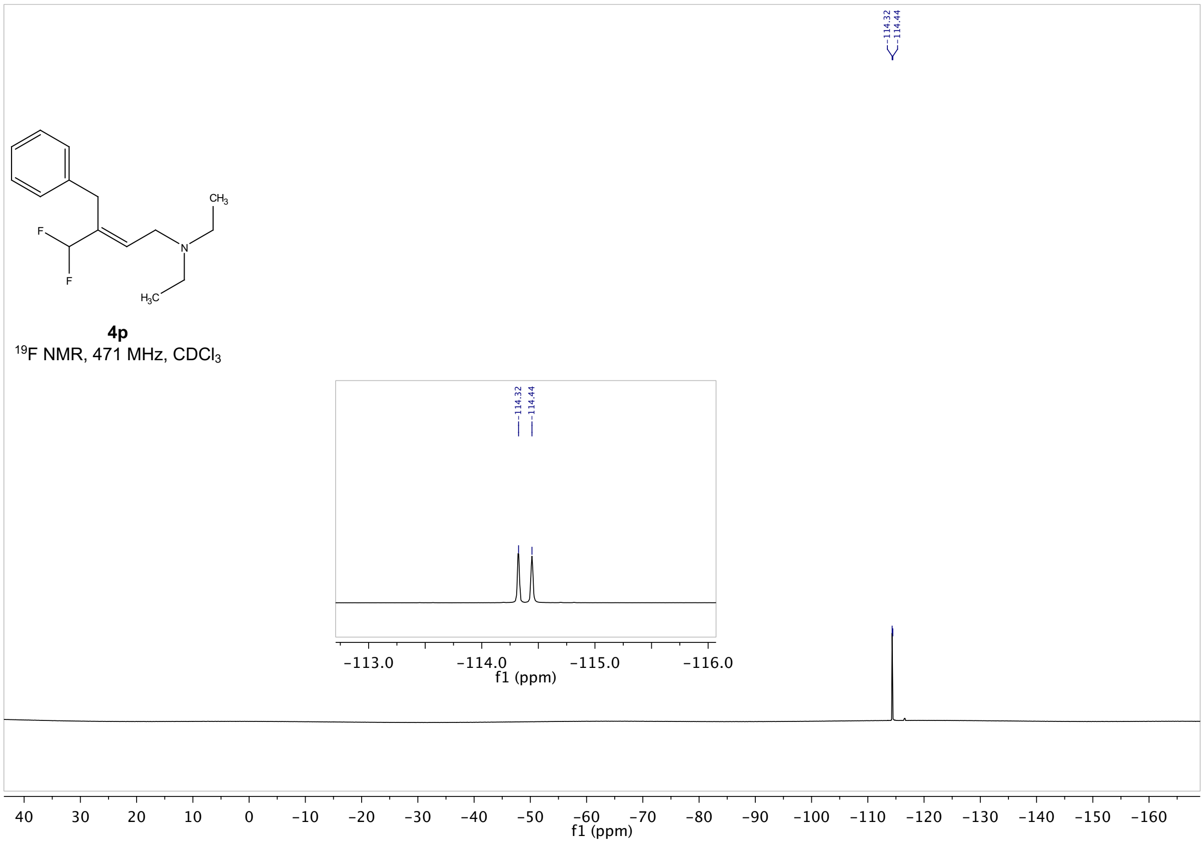


**Supplementary Figure 58.** $^{19}$F NMR spectra of compound **4p**

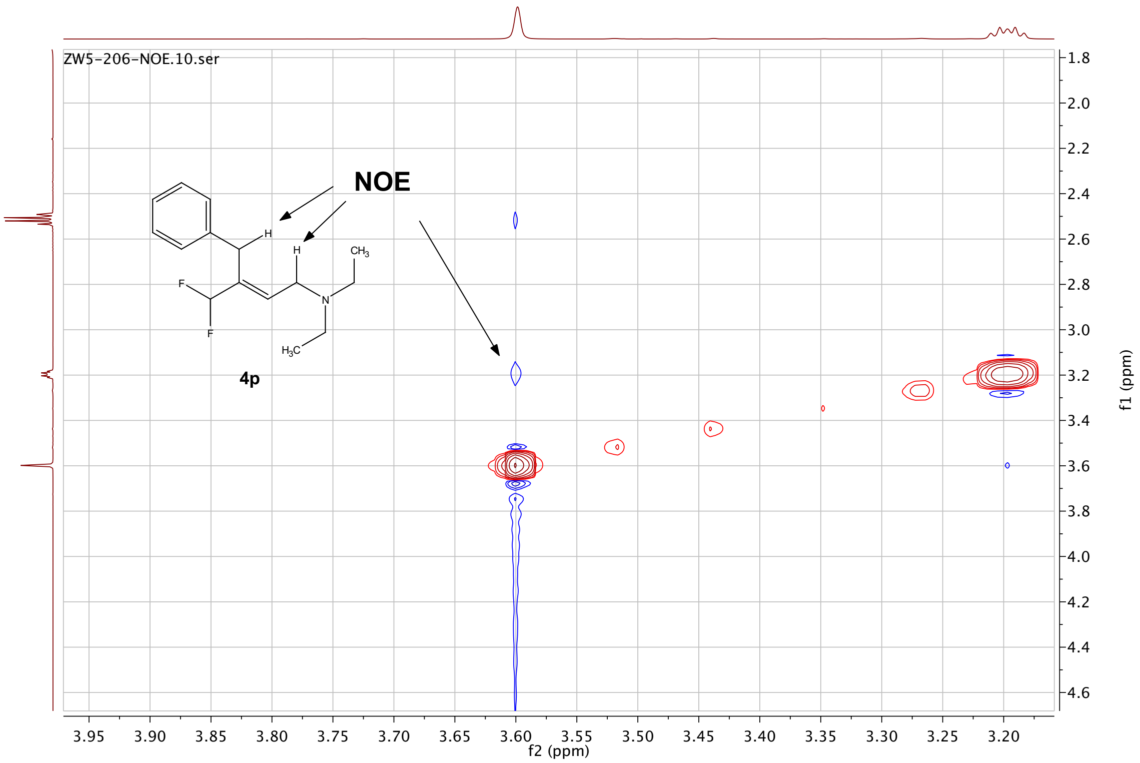


**Supplementary Figure 59.** NOE spectra of compound **4p**

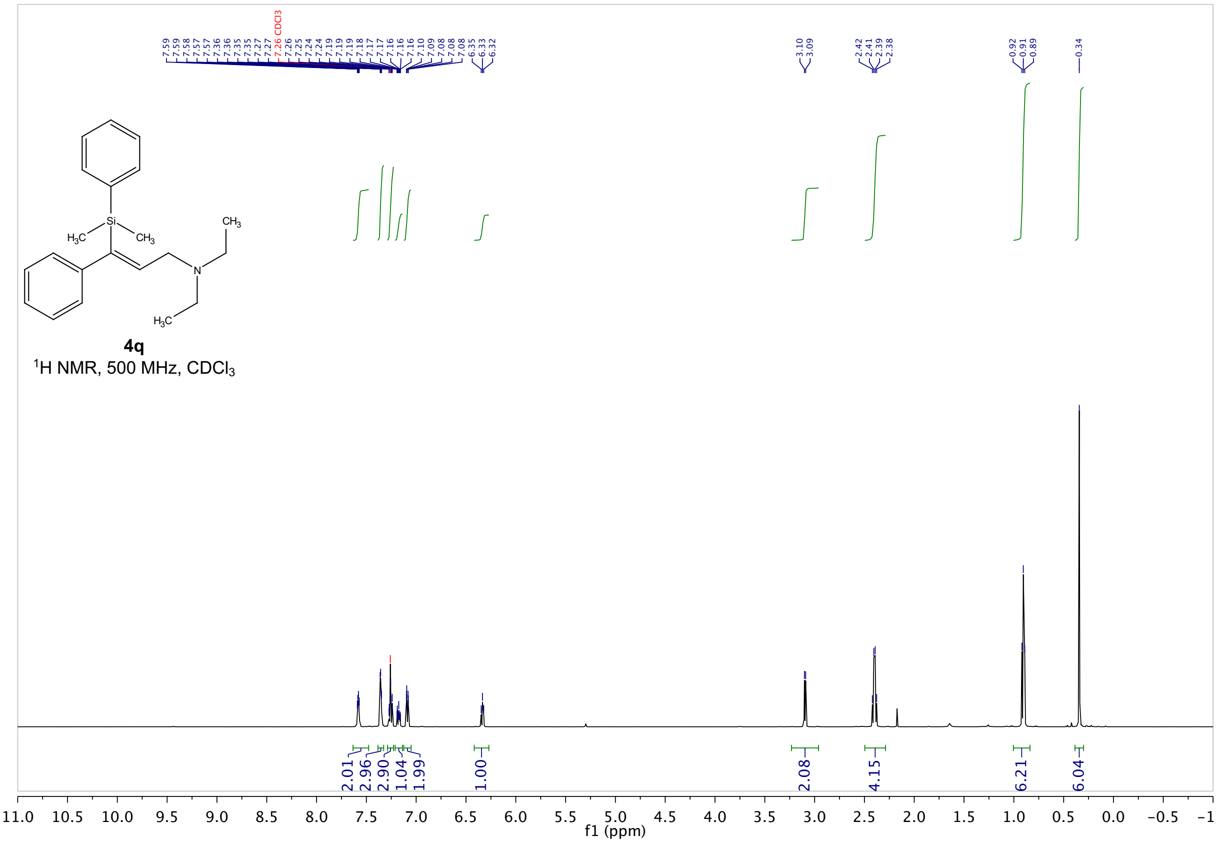


**Supplementary Figure 60.** $^{1}$H NMR spectra of compound **4q**

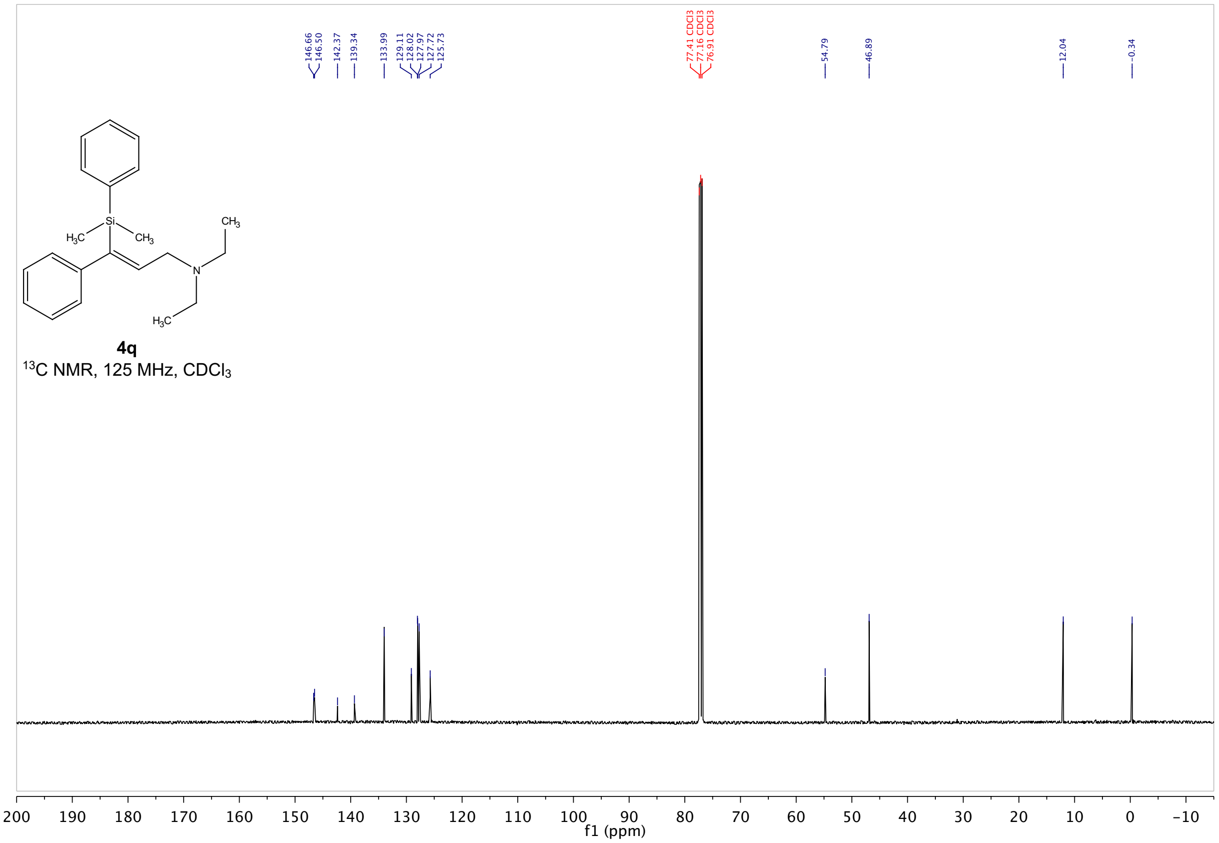


**Supplementary Figure 61.** $^{13}$C NMR spectra of compound **4q**

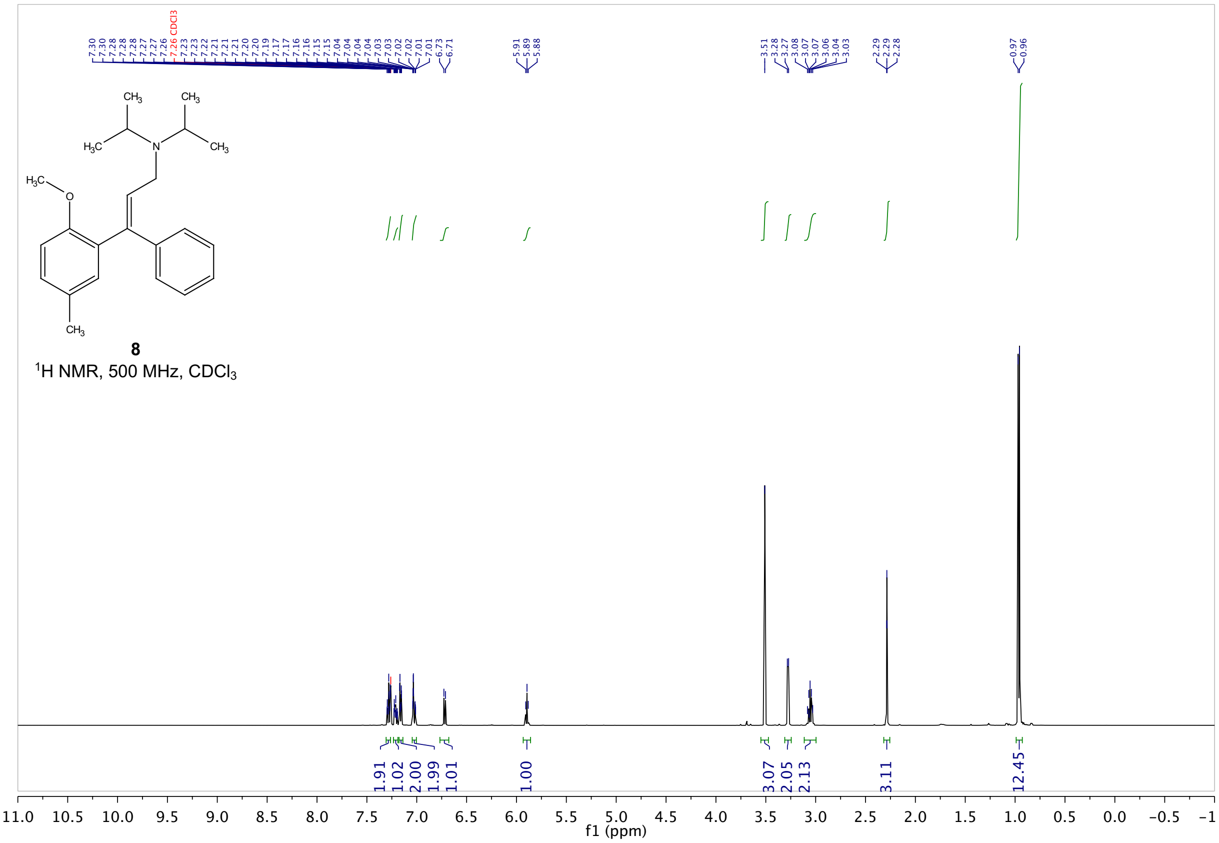

**Supplementary Figure 62.** $^{1}$H NMR spectra of compound **8**

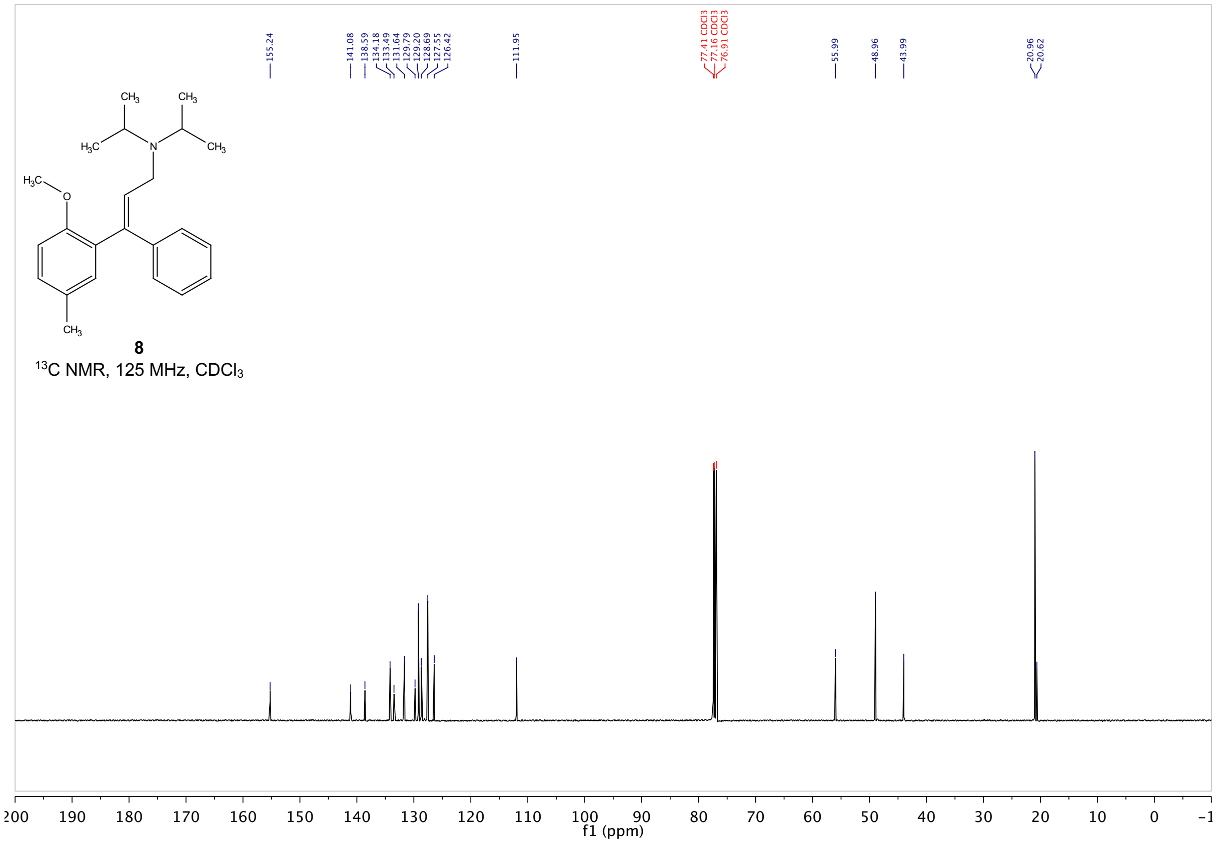

**Supplementary Figure 63.** $^{13}$C NMR spectra of compound **8**

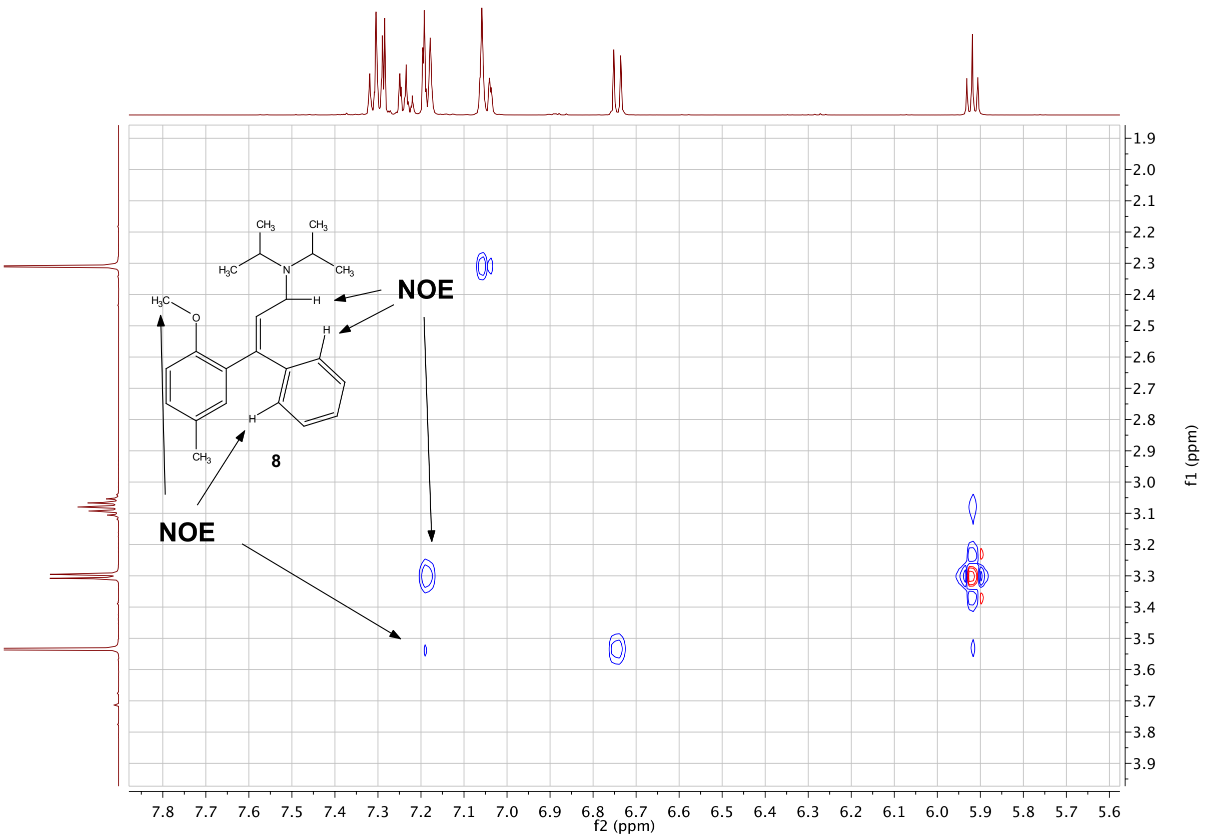


**Supplementary Figure 64.** NOE spectra of compound **8**

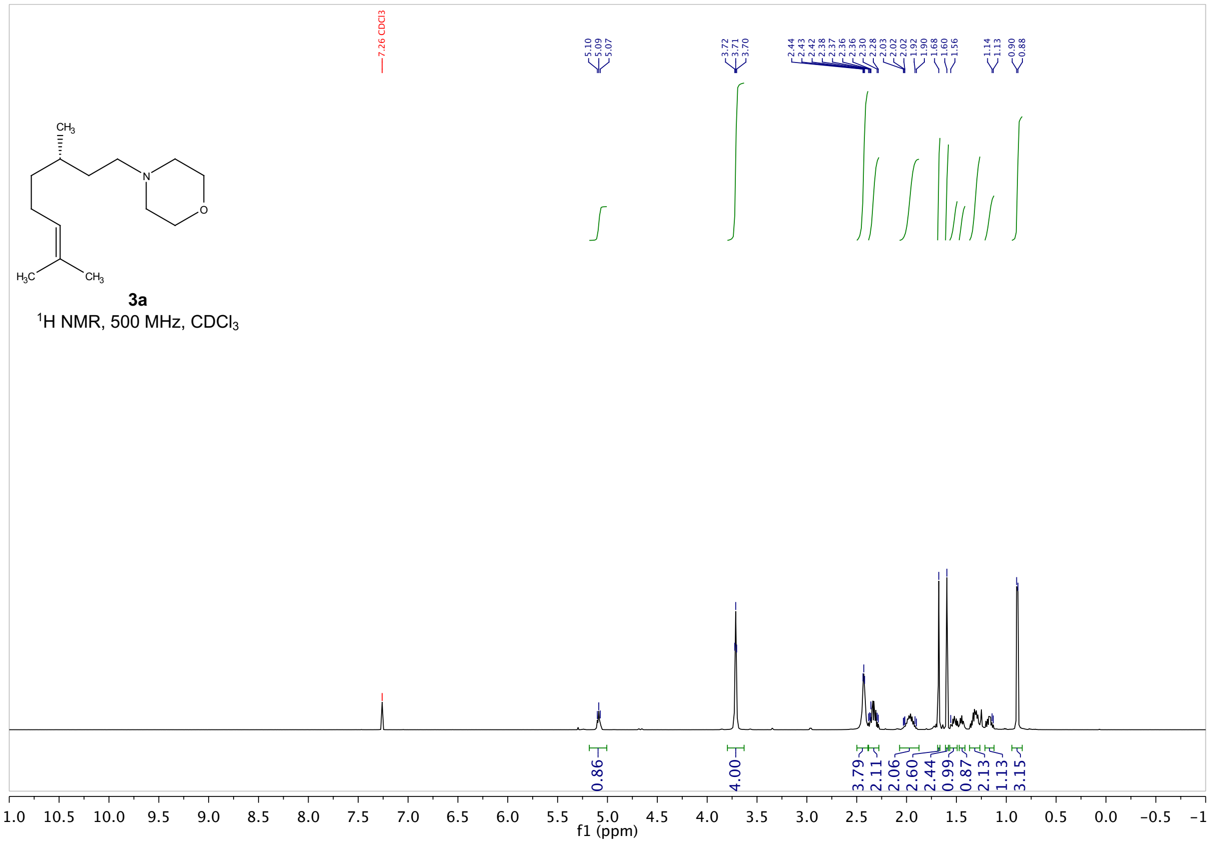


**Supplementary Figure 65.** $^{1}$H NMR spectra of compound **3a**

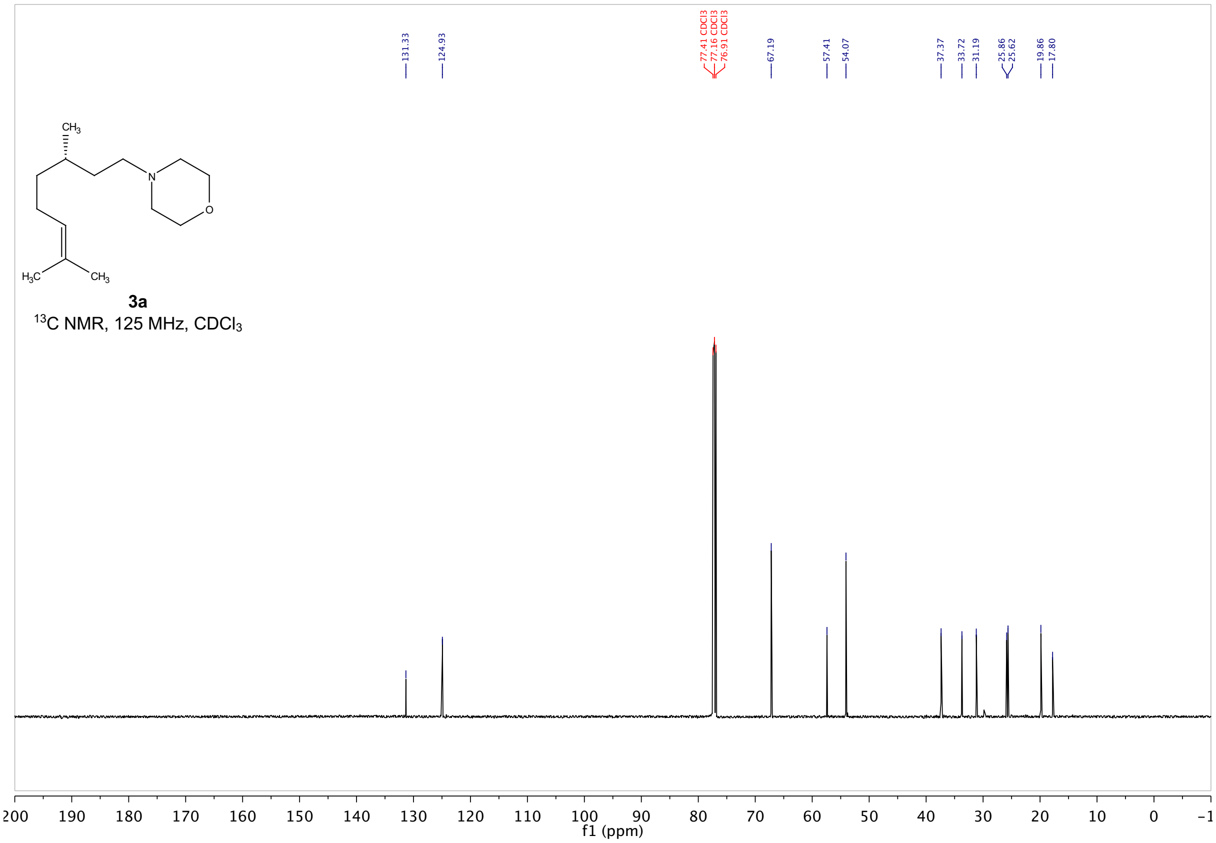


**Supplementary Figure 66.** $^{13}$C NMR spectra of compound **3a**

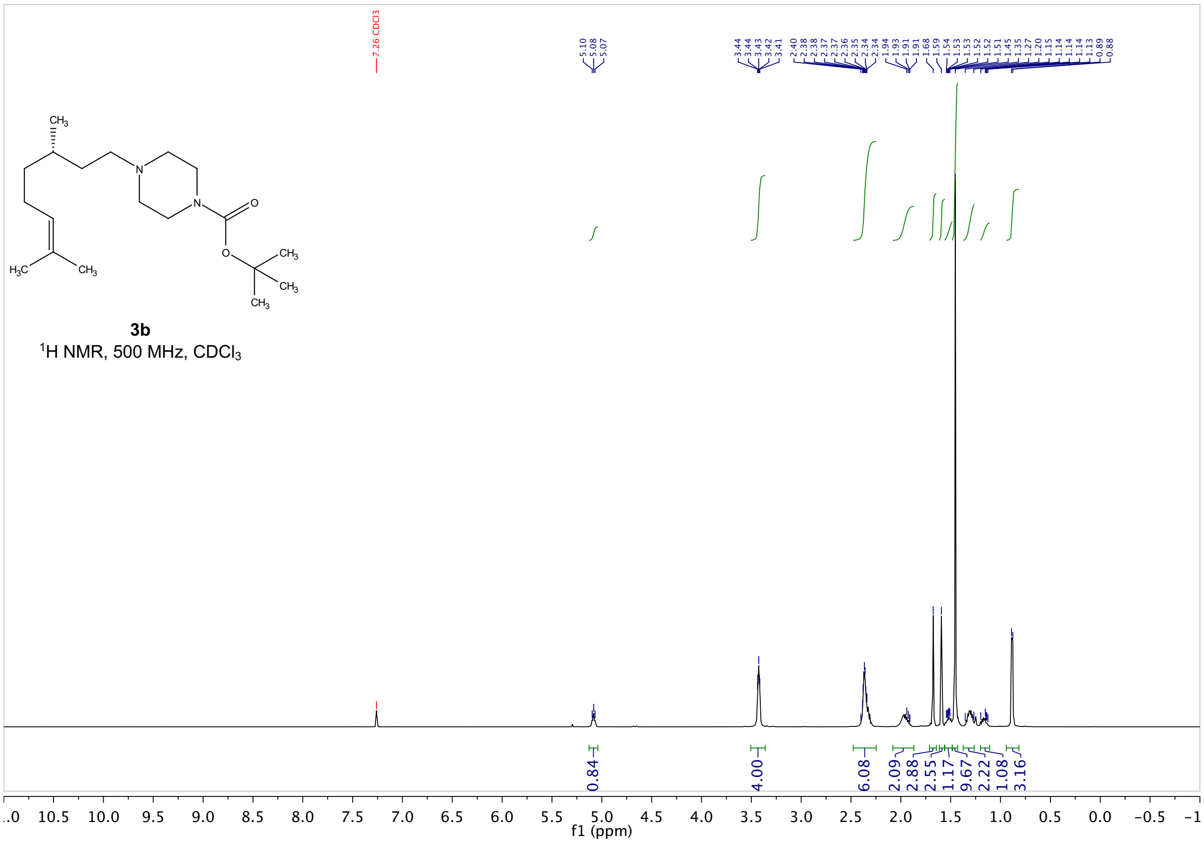

**Supplementary Figure 67.** $^{1}$H NMR spectra of compound **3b**

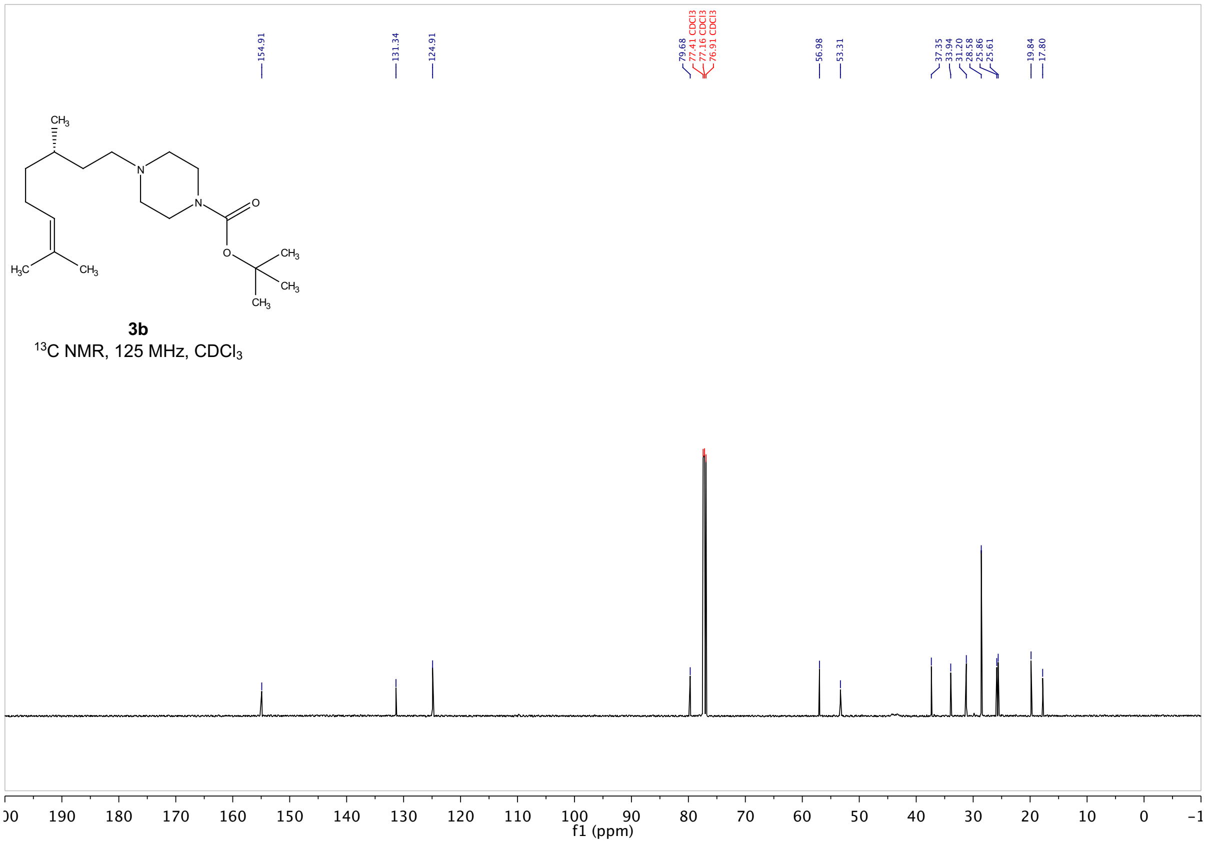

**Supplementary Figure 68.** $^{13}$C NMR spectra of compound **3b**

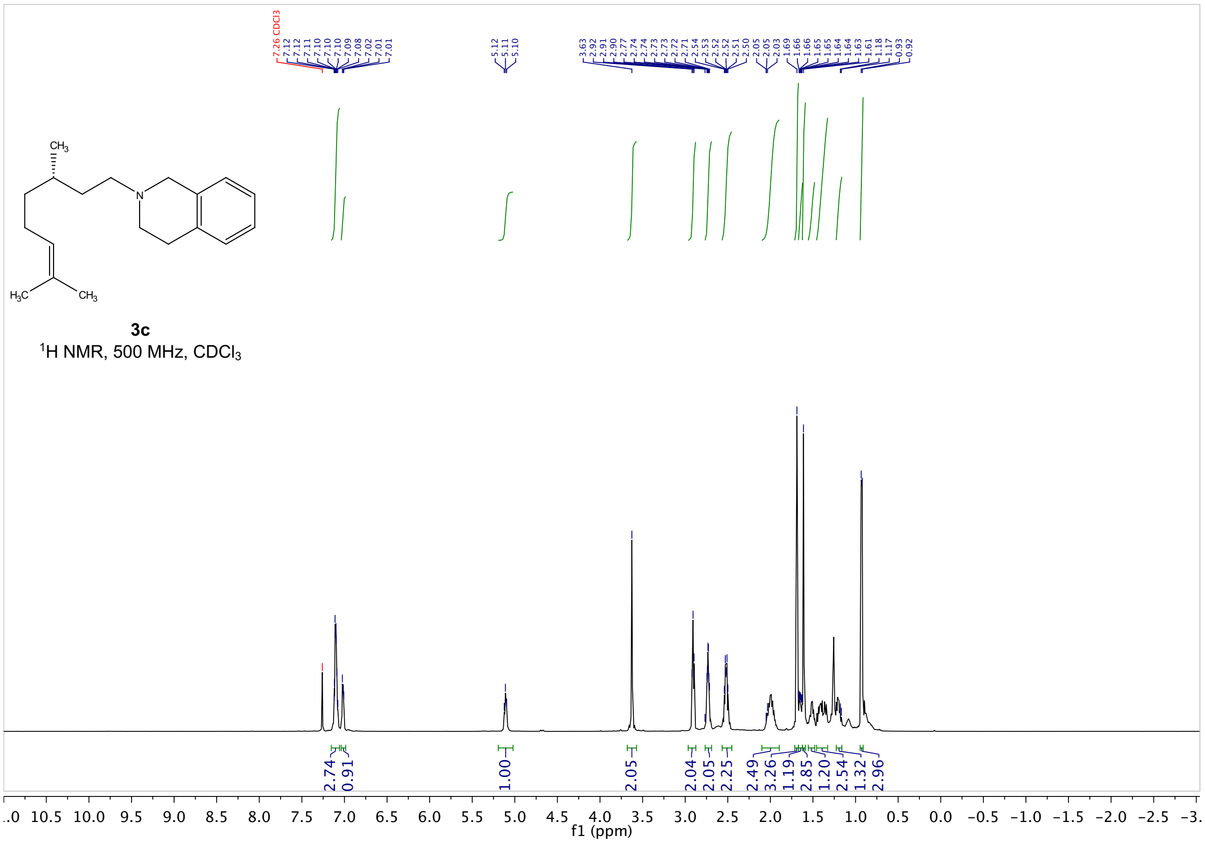


**Supplementary Figure 69.** $^{1}$H NMR spectra of compound **3c**

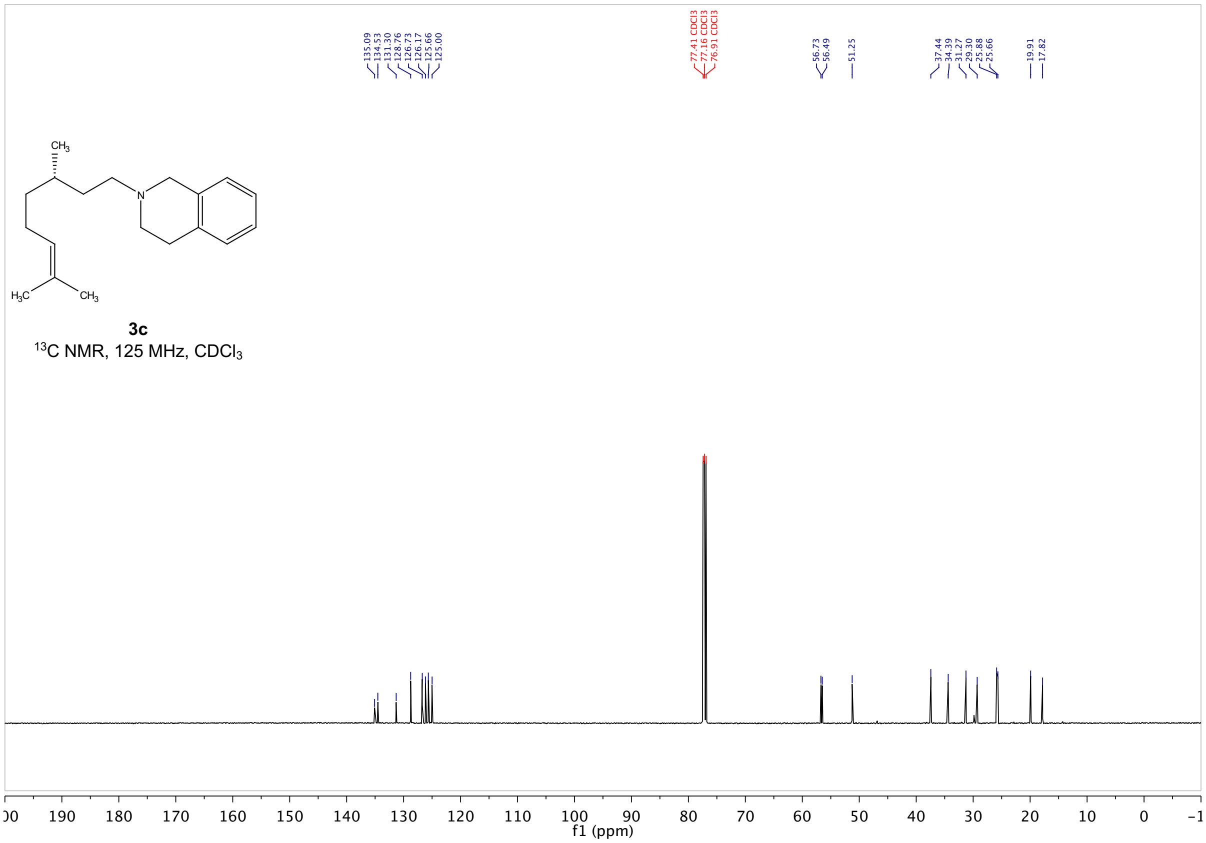


**Supplementary Figure 70.** $^{13}$C NMR spectra of compound **3c**

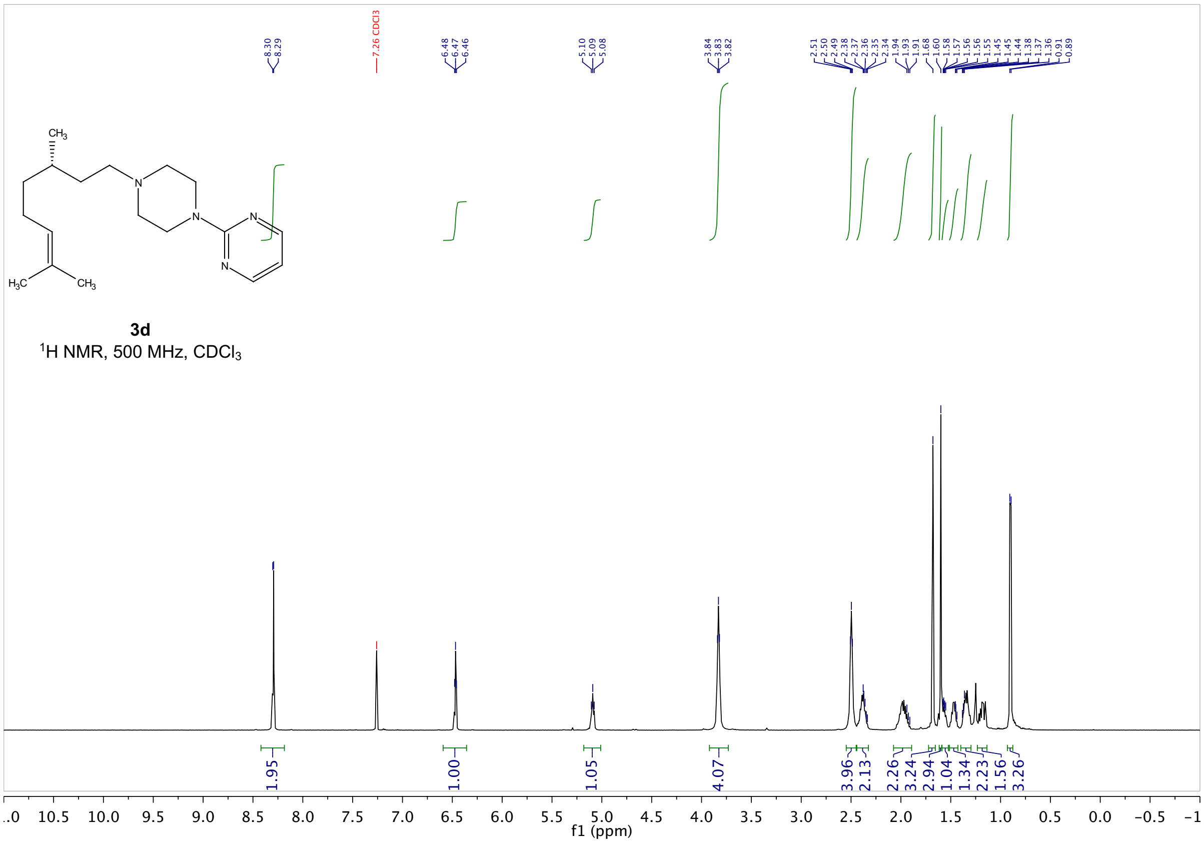

**Supplementary Figure 71.** $^{1}$H NMR spectra of compound **3d**

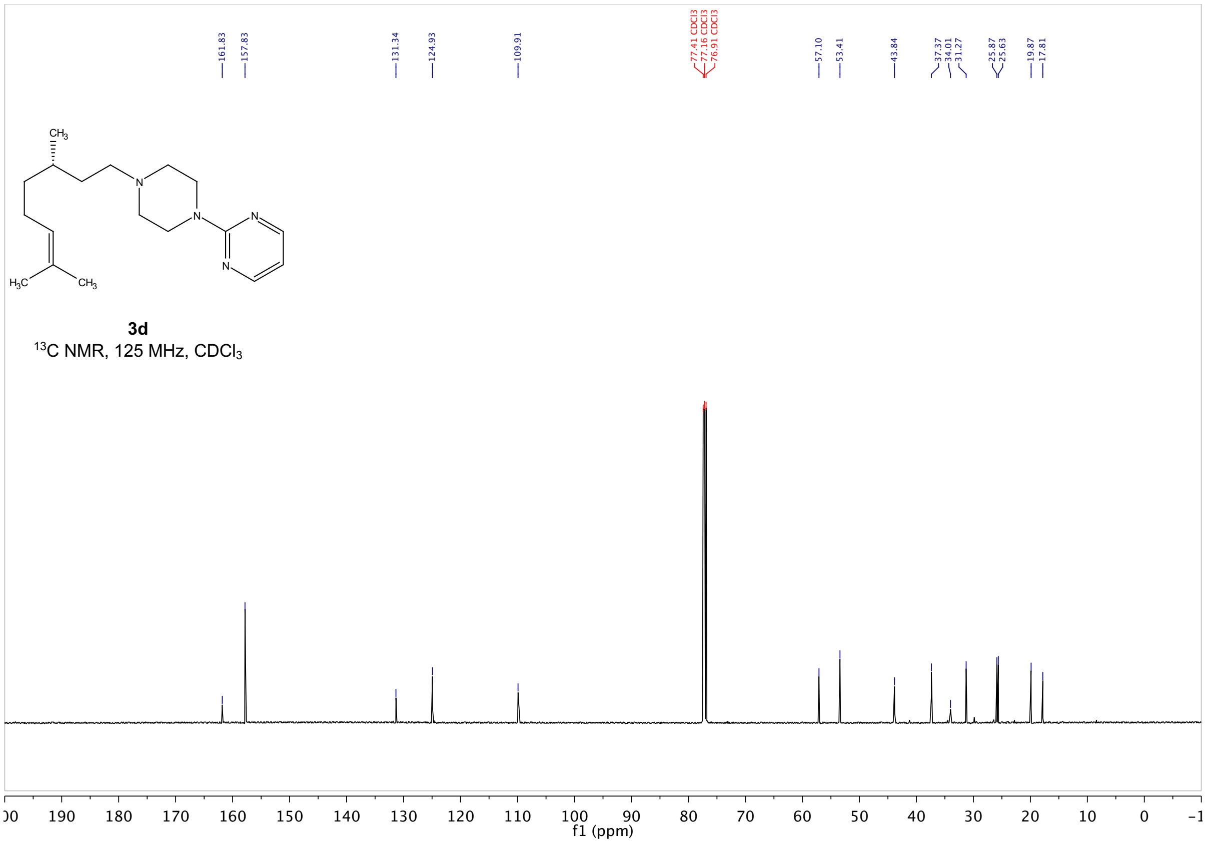

**Supplementary Figure 72.** $^{13}$C NMR spectra of compound **3d**

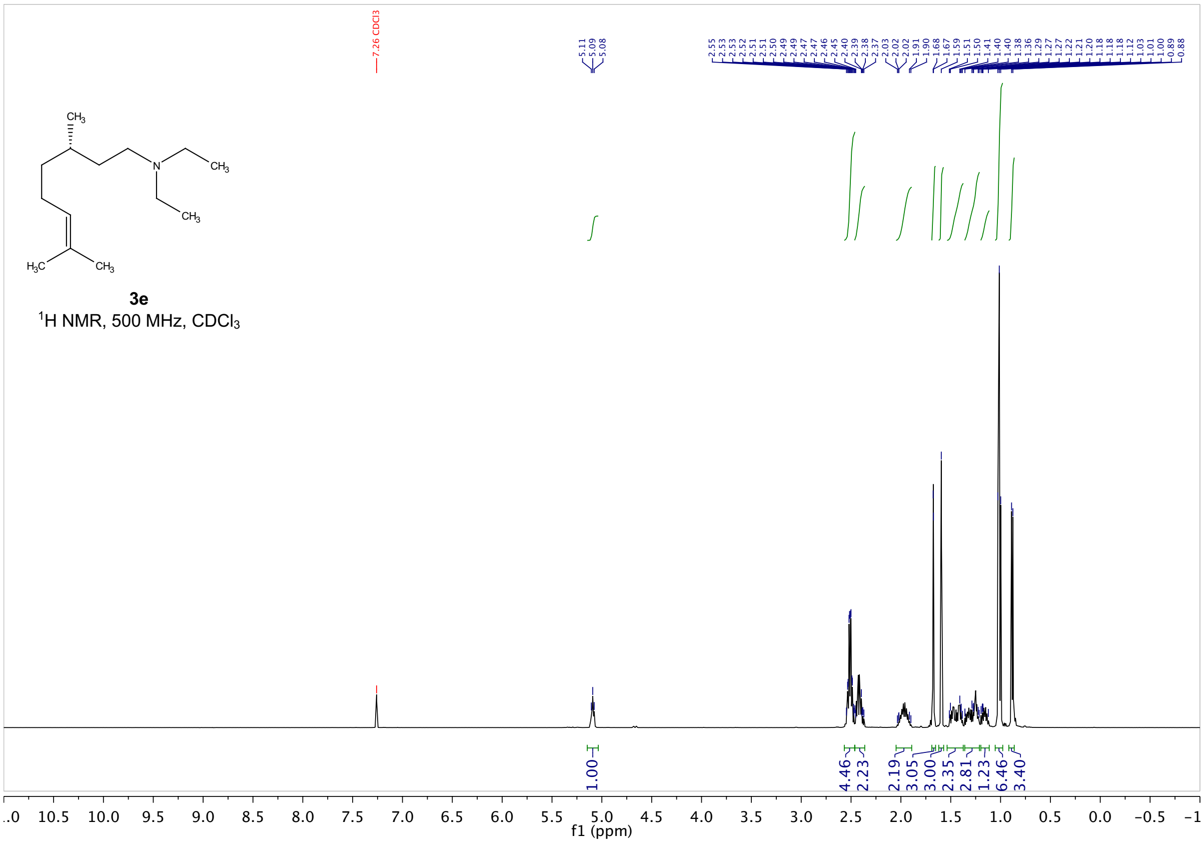

**Supplementary Figure 73.** $^{1}$H NMR spectra of compound **3e**

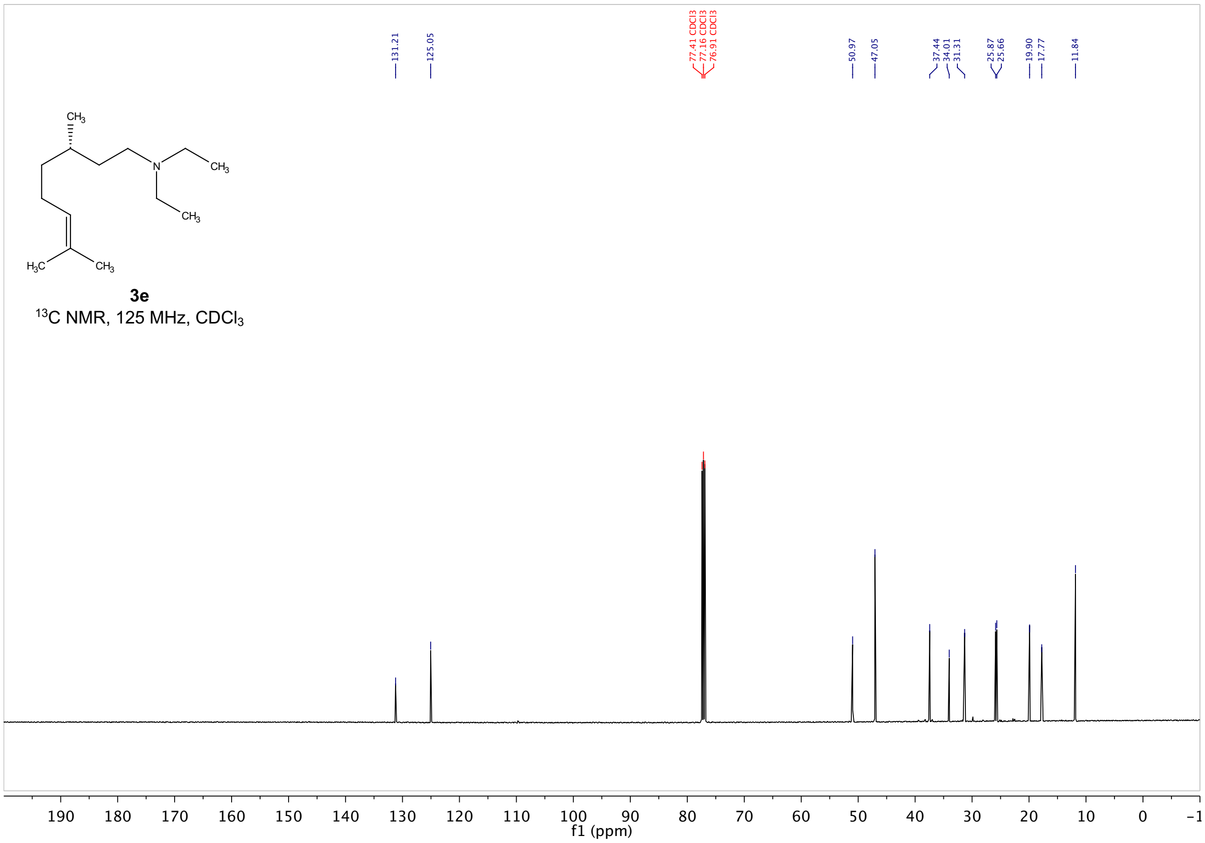

**Supplementary Figure 74.** $^{13}$C NMR spectra of compound **3e**

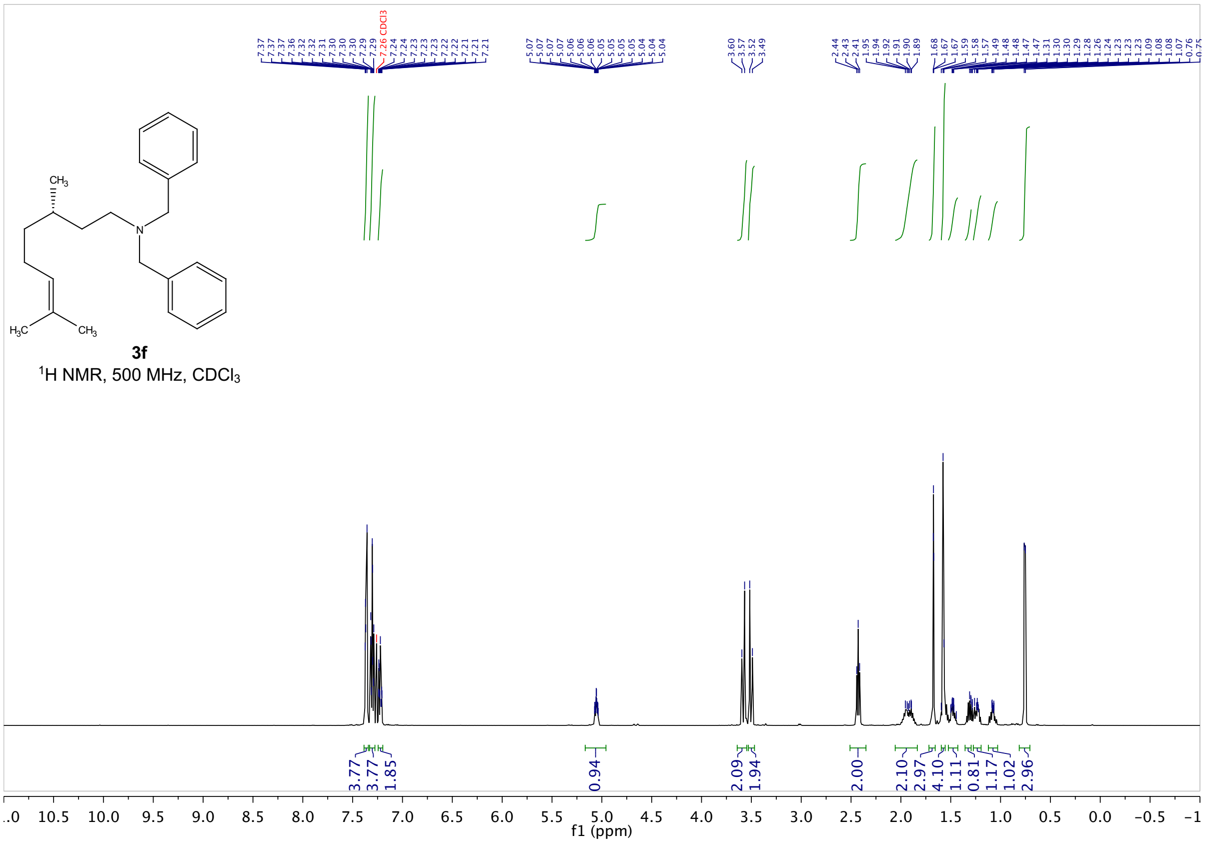

**Supplementary Figure 75.** $^{1}$H NMR spectra of compound **3f**

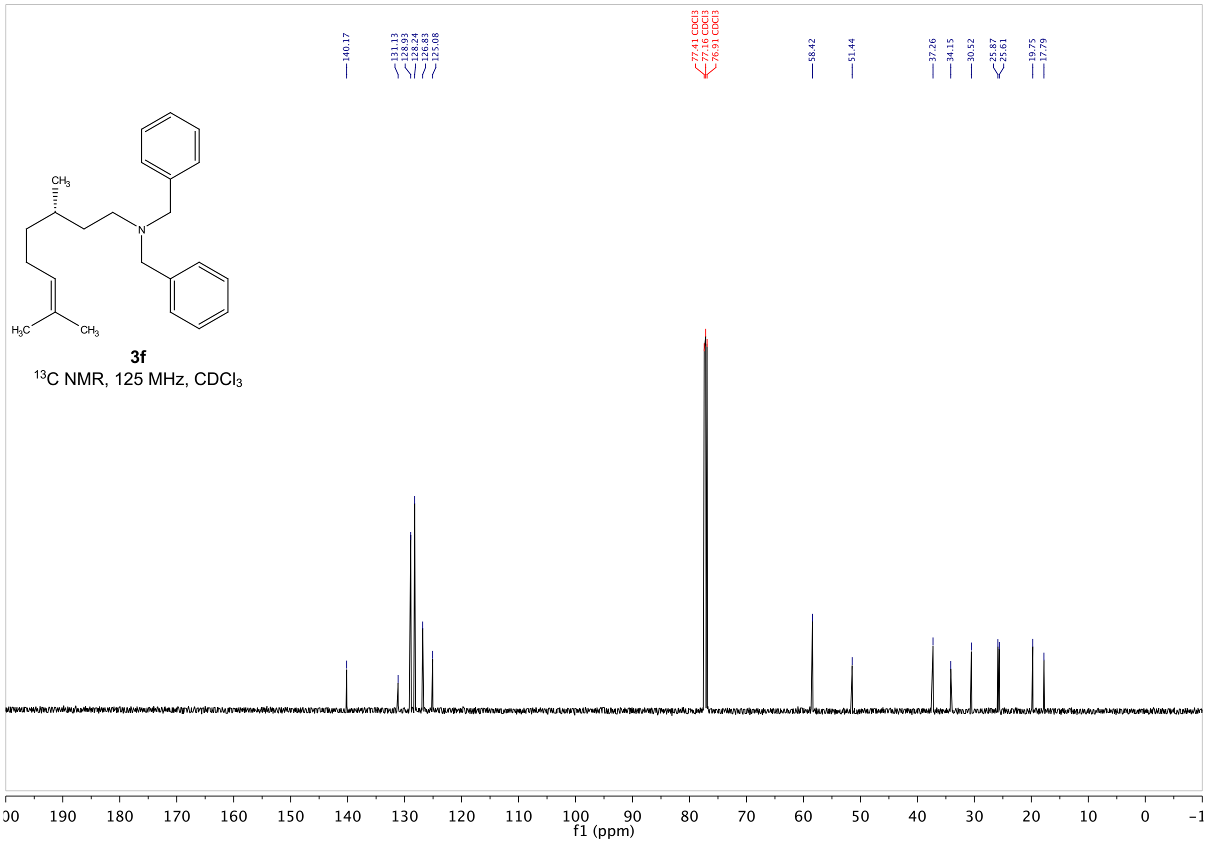

**Supplementary Figure 76.** $^{13}$C NMR spectra of compound **3f**

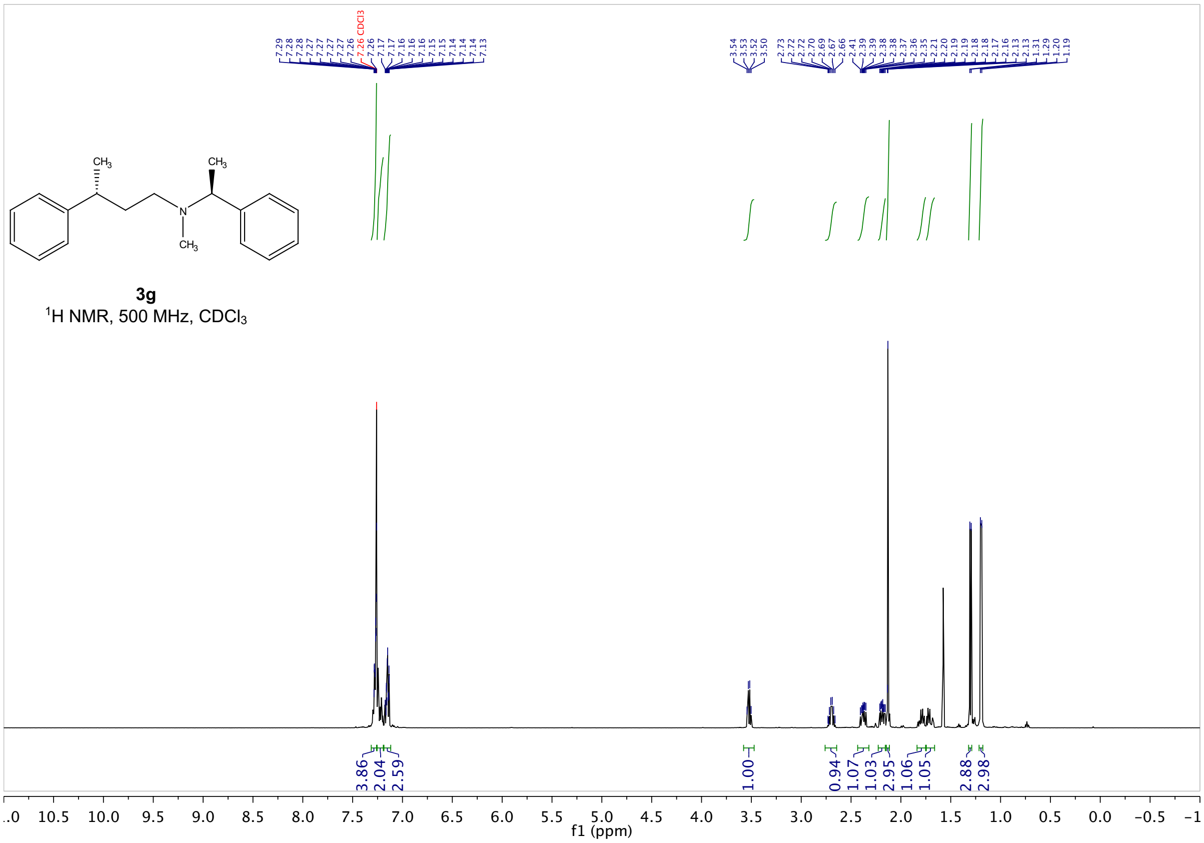


**Supplementary Figure 77.** $^{1}$H NMR spectra of compound **3g**

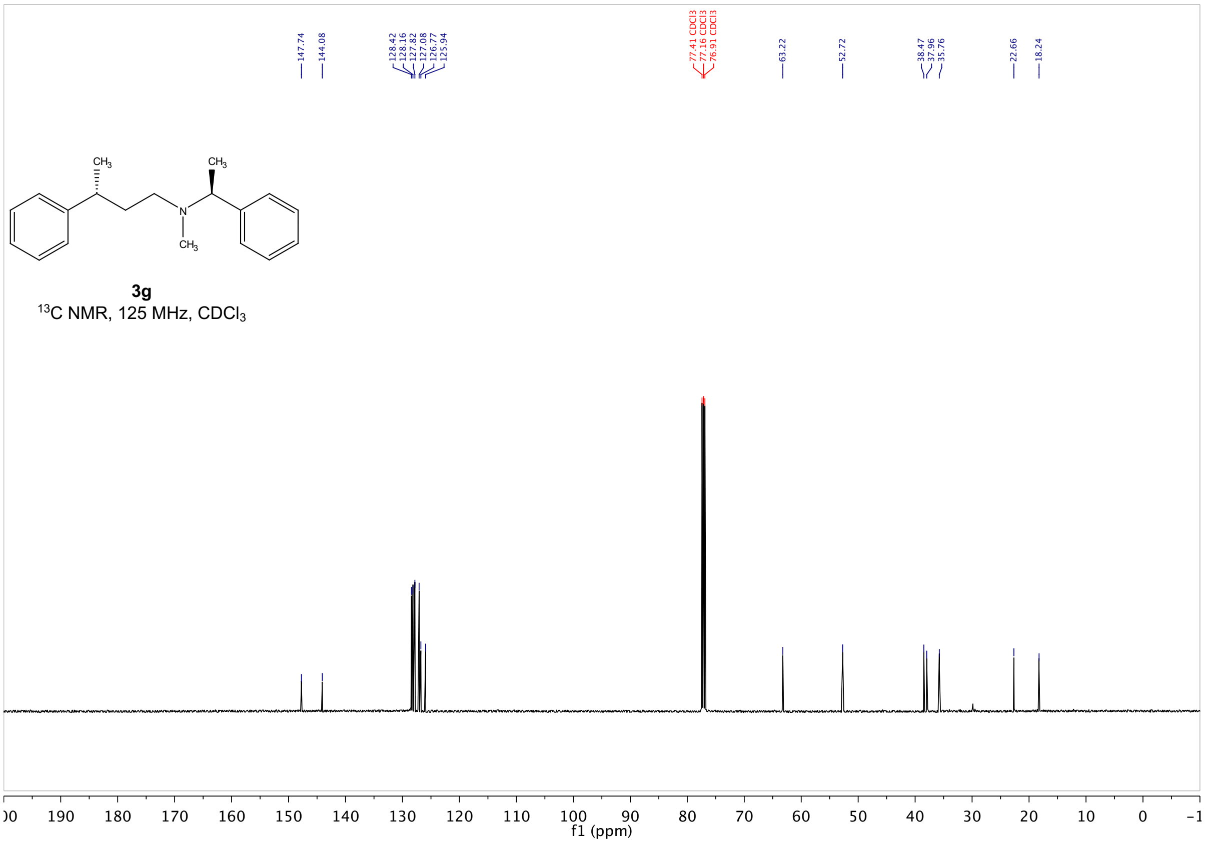


**Supplementary Figure 78.** $^{13}$C NMR spectra of compound **3g**

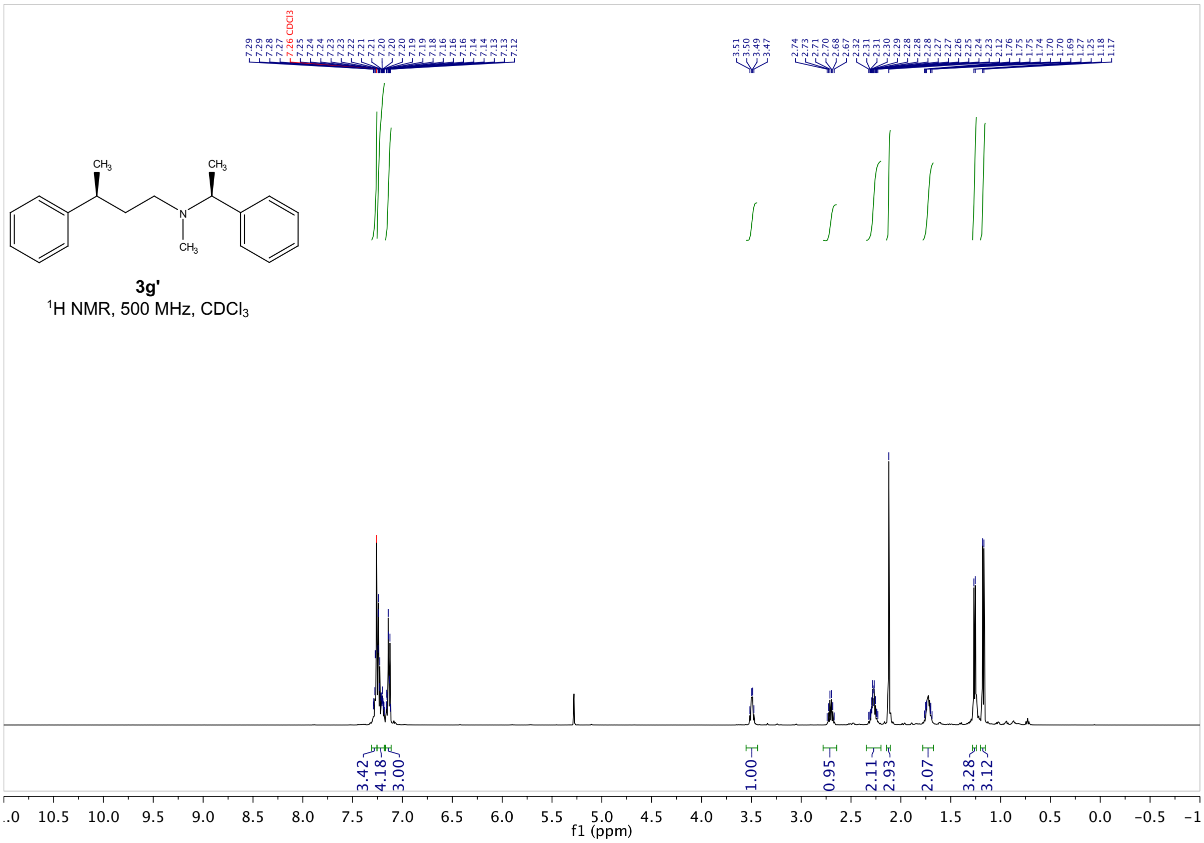


**Supplementary Figure 79.** $^{1}$H NMR spectra of compound **3g'**

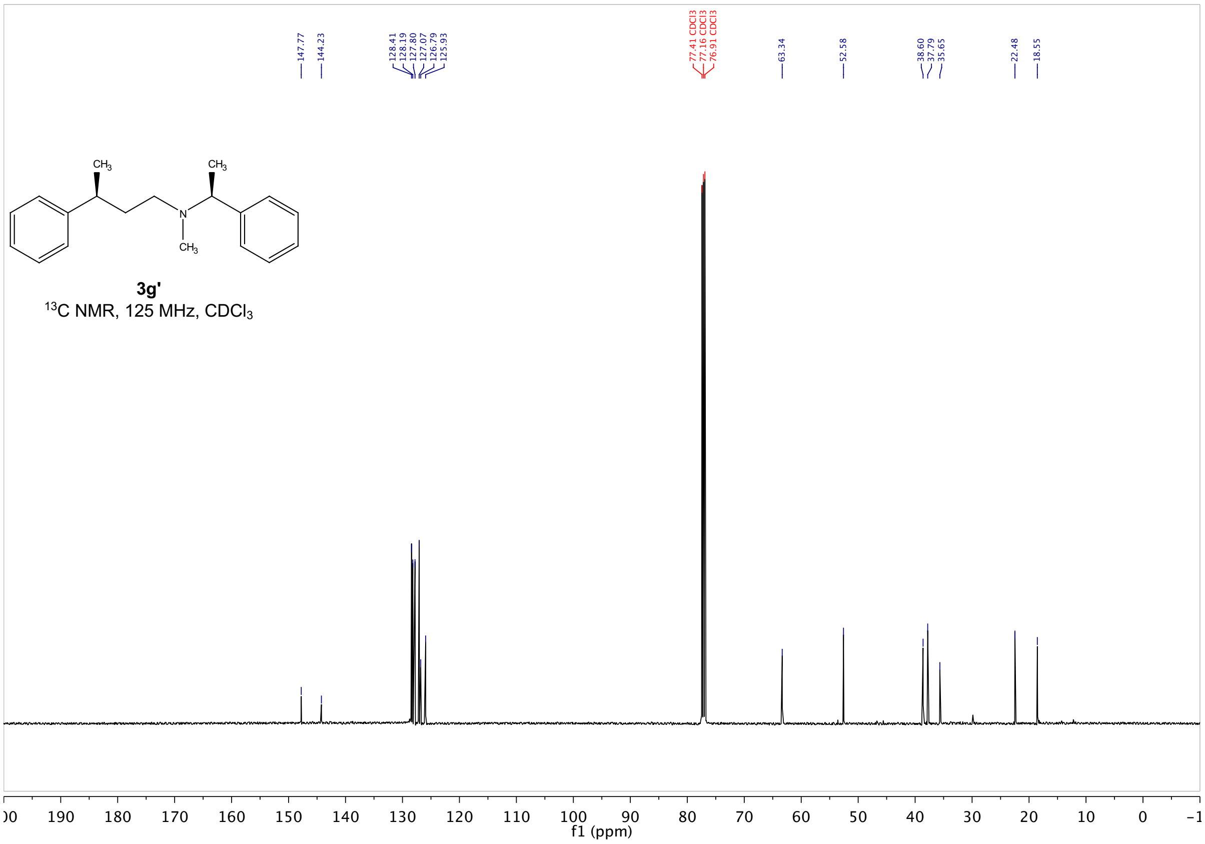


**Supplementary Figure 80.** $^{13}$C NMR spectra of compound **3g'**

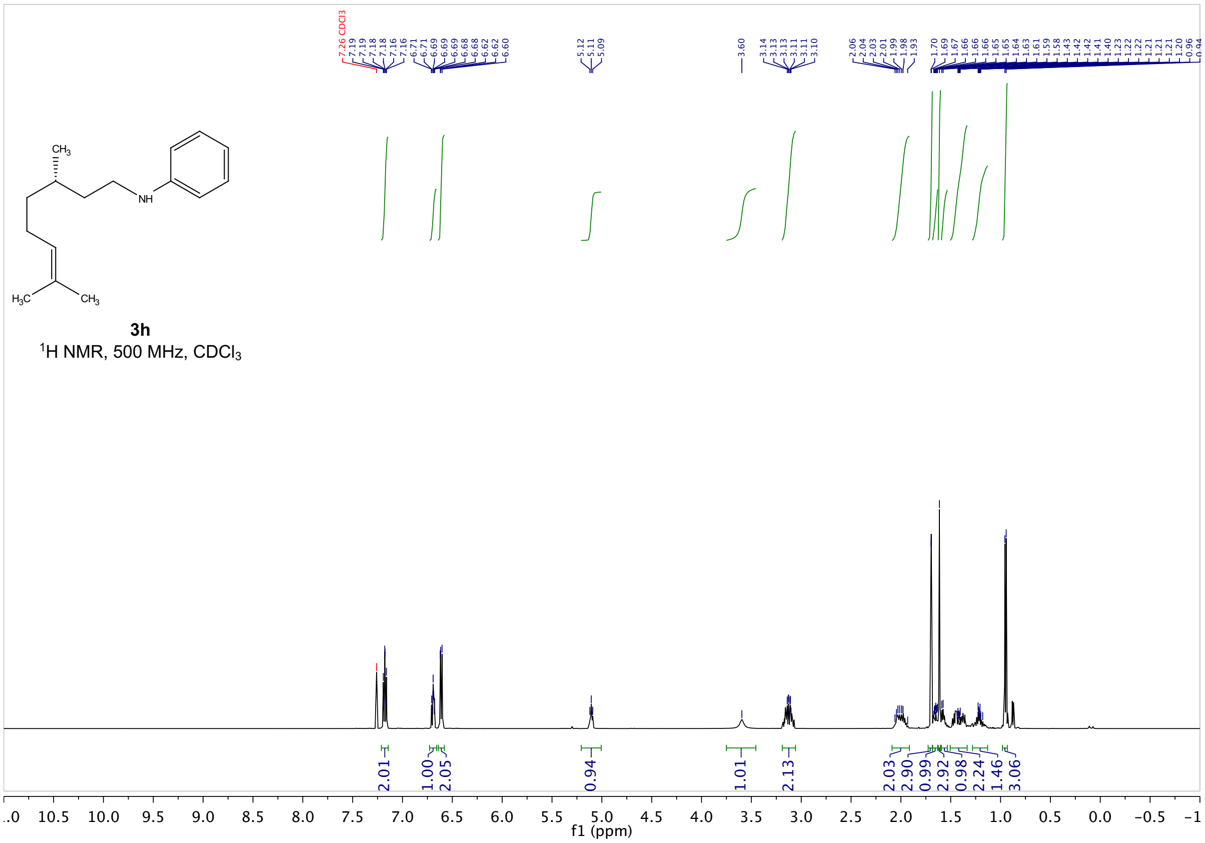


**Supplementary Figure 81.** $^{1}$H NMR spectra of compound **3h**

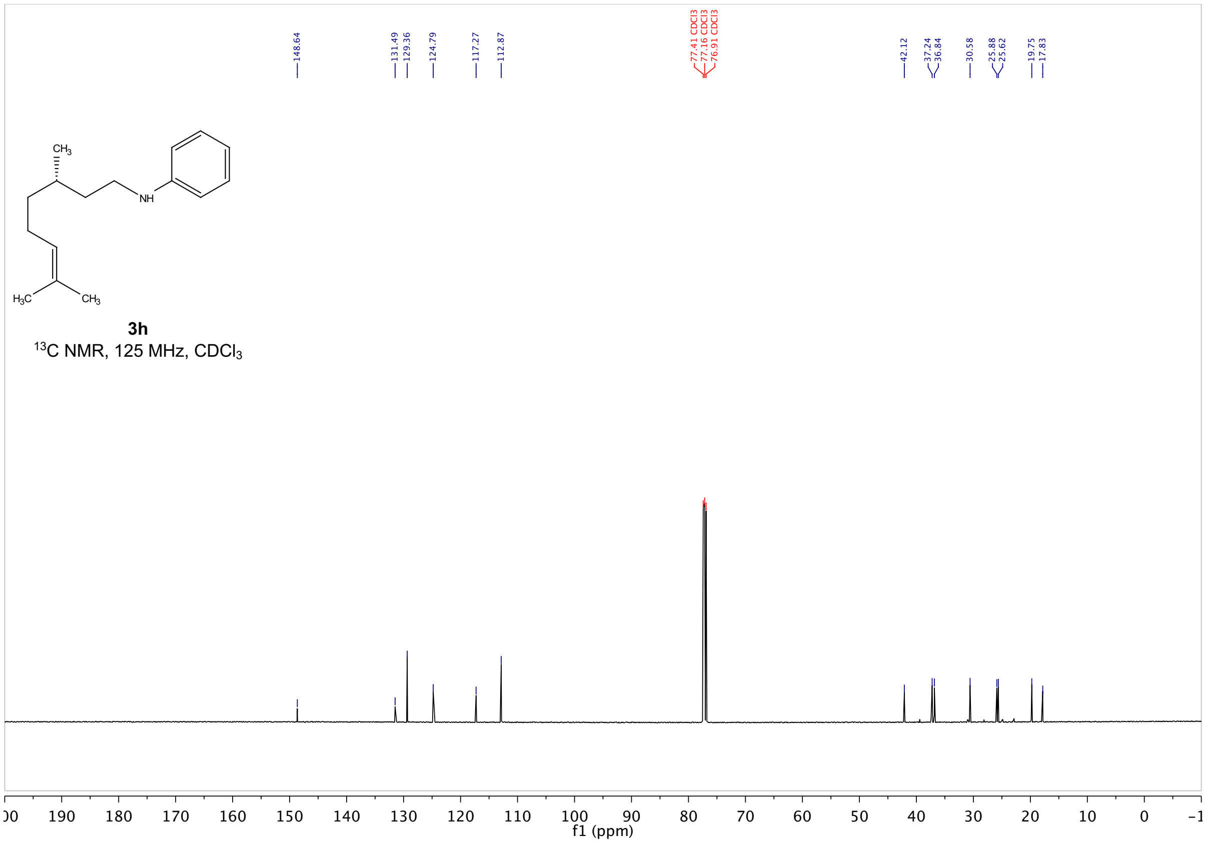


**Supplementary Figure 82.** $^{13}$C NMR spectra of compound **3h**

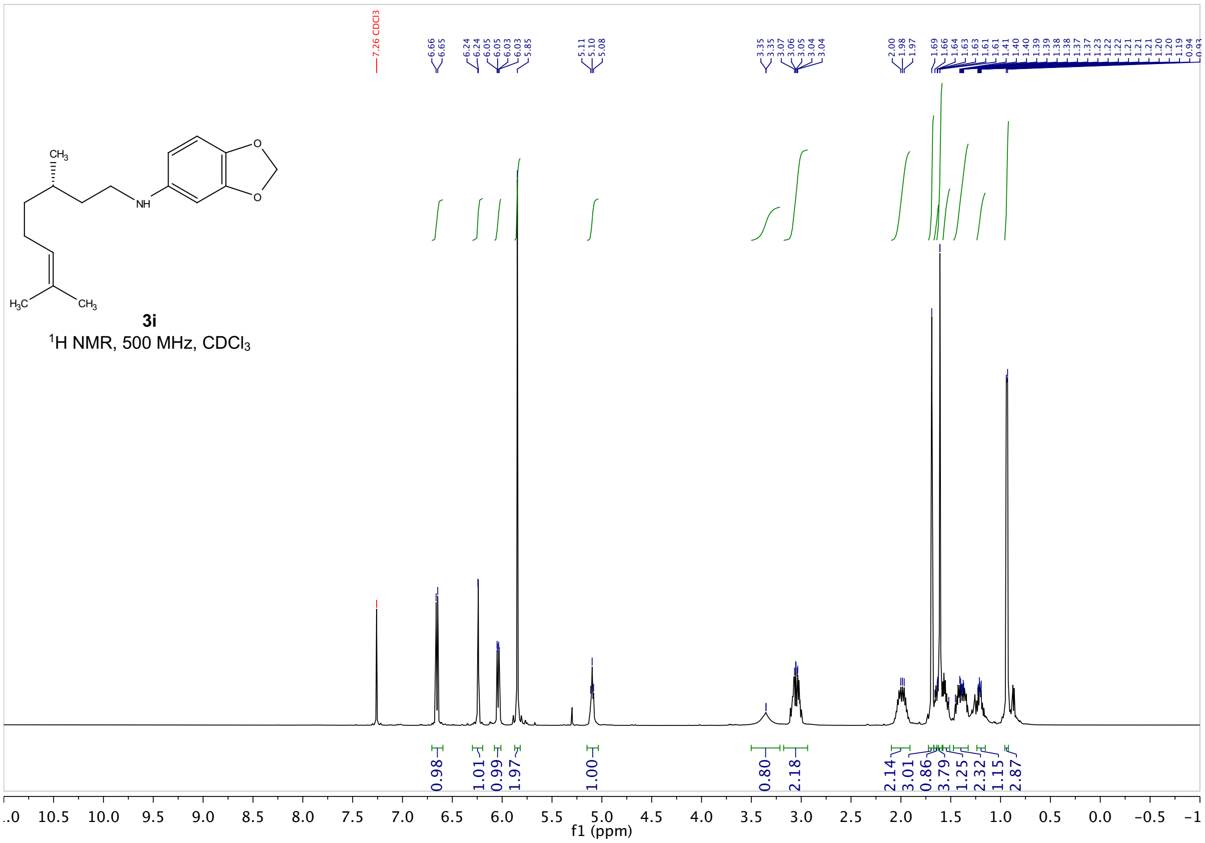


**Supplementary Figure 83.** $^{1}H$ NMR spectra of compound **3i**

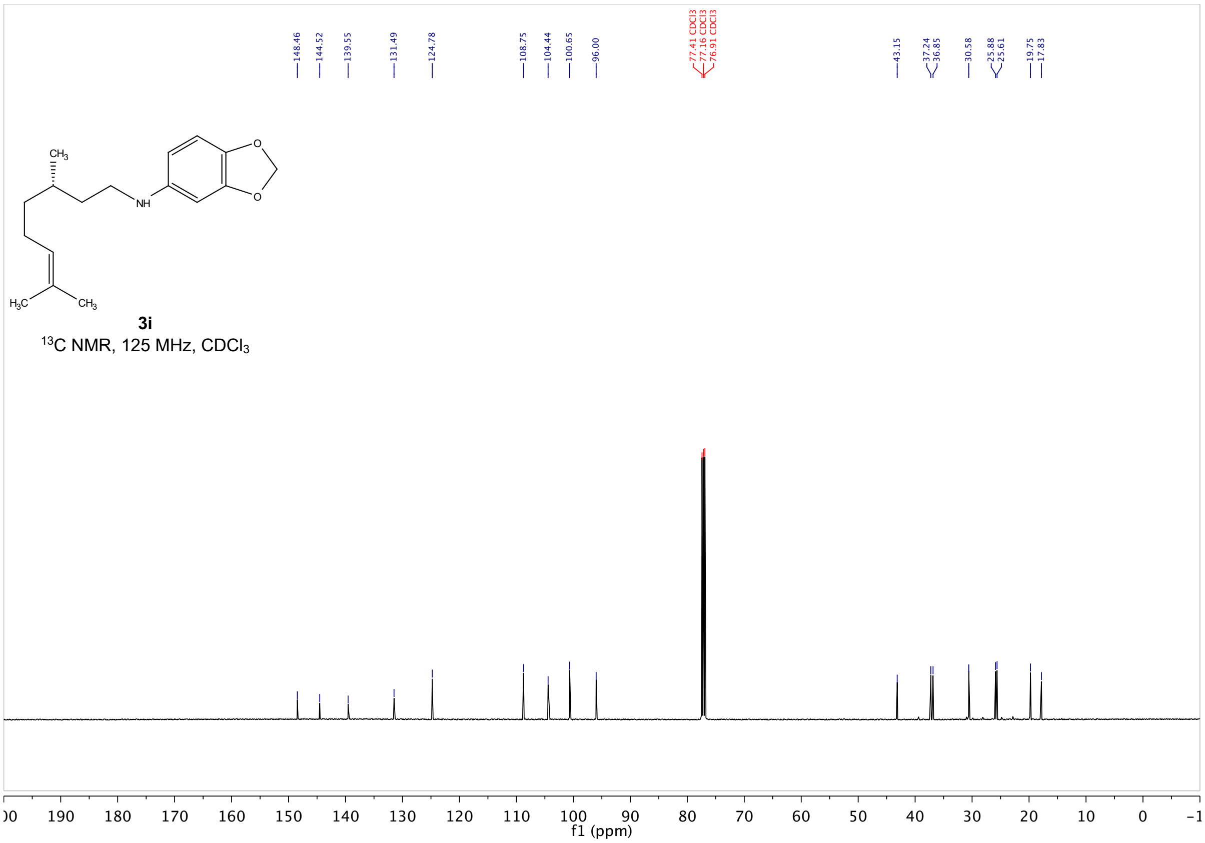


**Supplementary Figure 84.** $^{13}C$ NMR spectra of compound **3i**

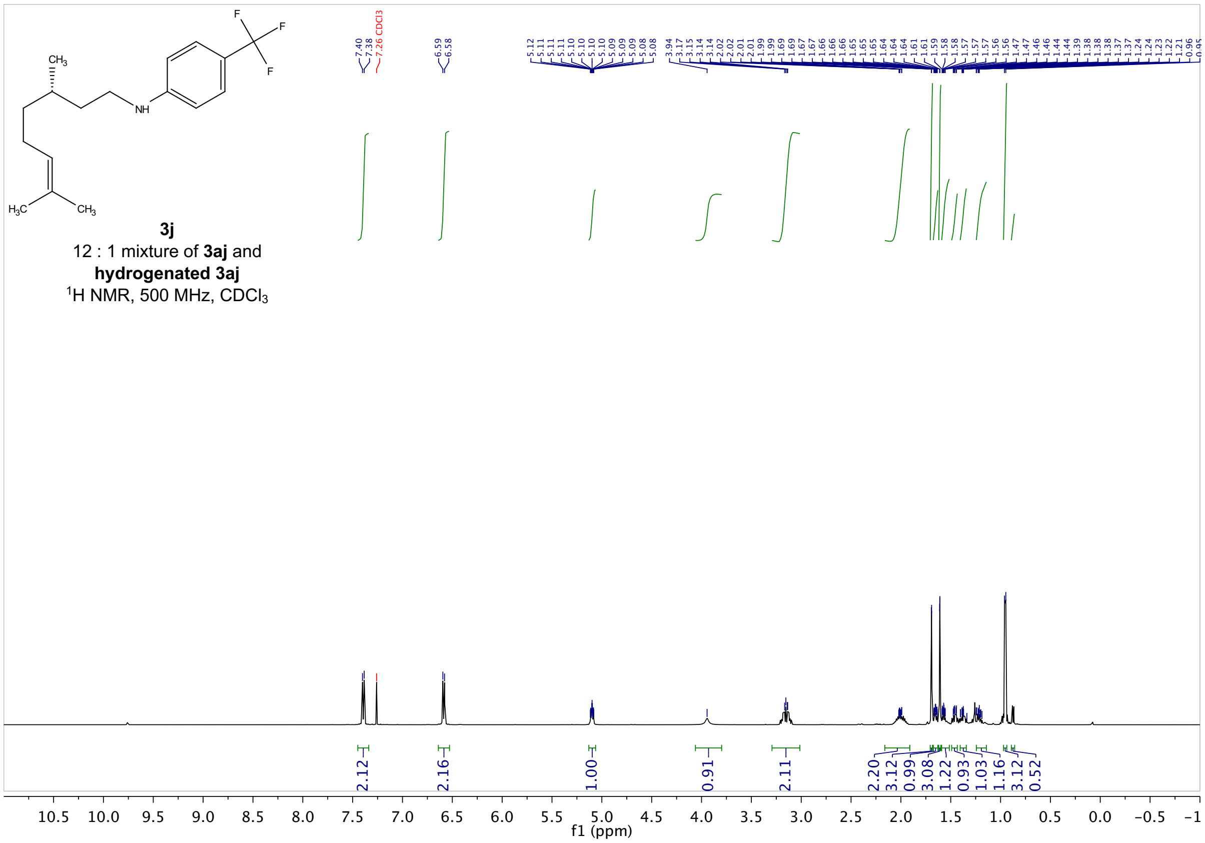


**Supplementary Figure 85.** $^{1}$H NMR spectra of compound **3j**

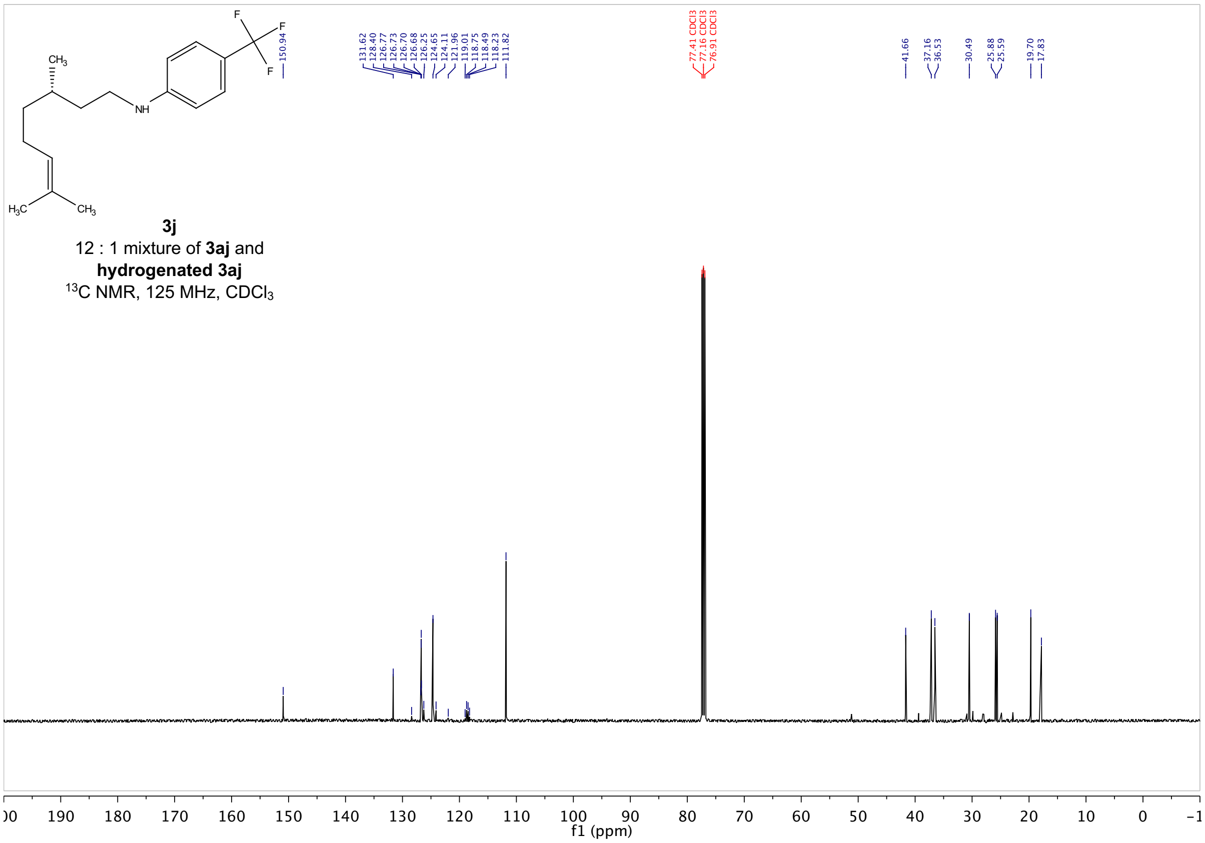


**Supplementary Figure 86.** $^{13}$C NMR spectra of compound **3j**

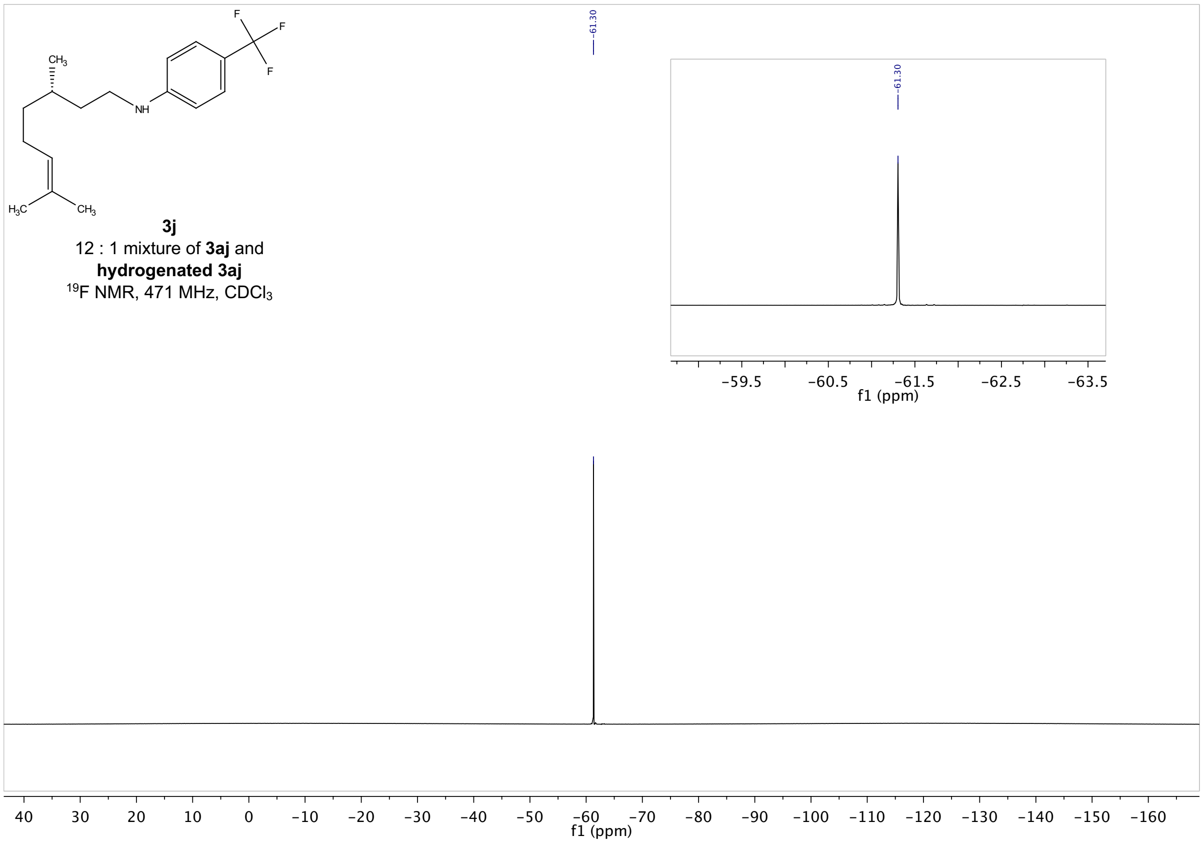


**Supplementary Figure 87.** $^{19}F$ NMR spectra of compound **3j**

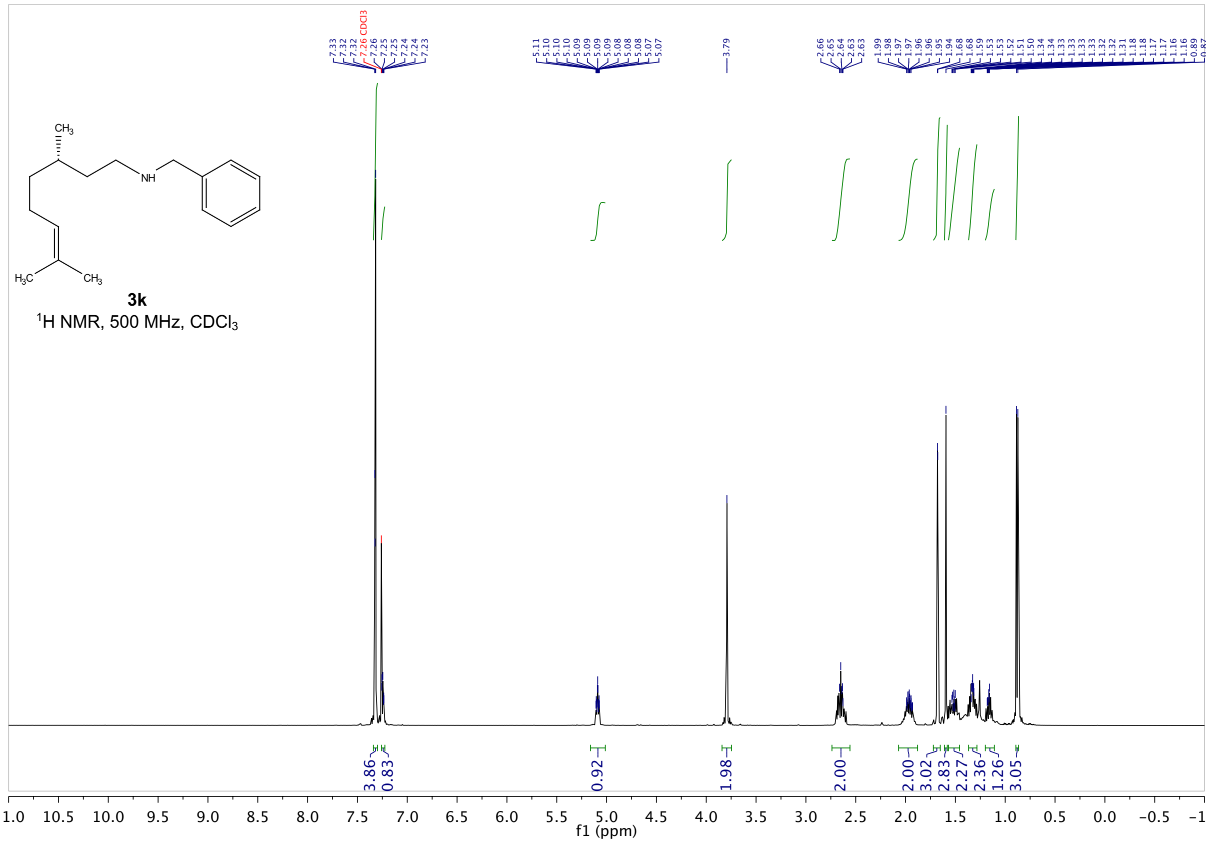


**Supplementary Figure 88.** $^1$H NMR spectra of compound **3k**

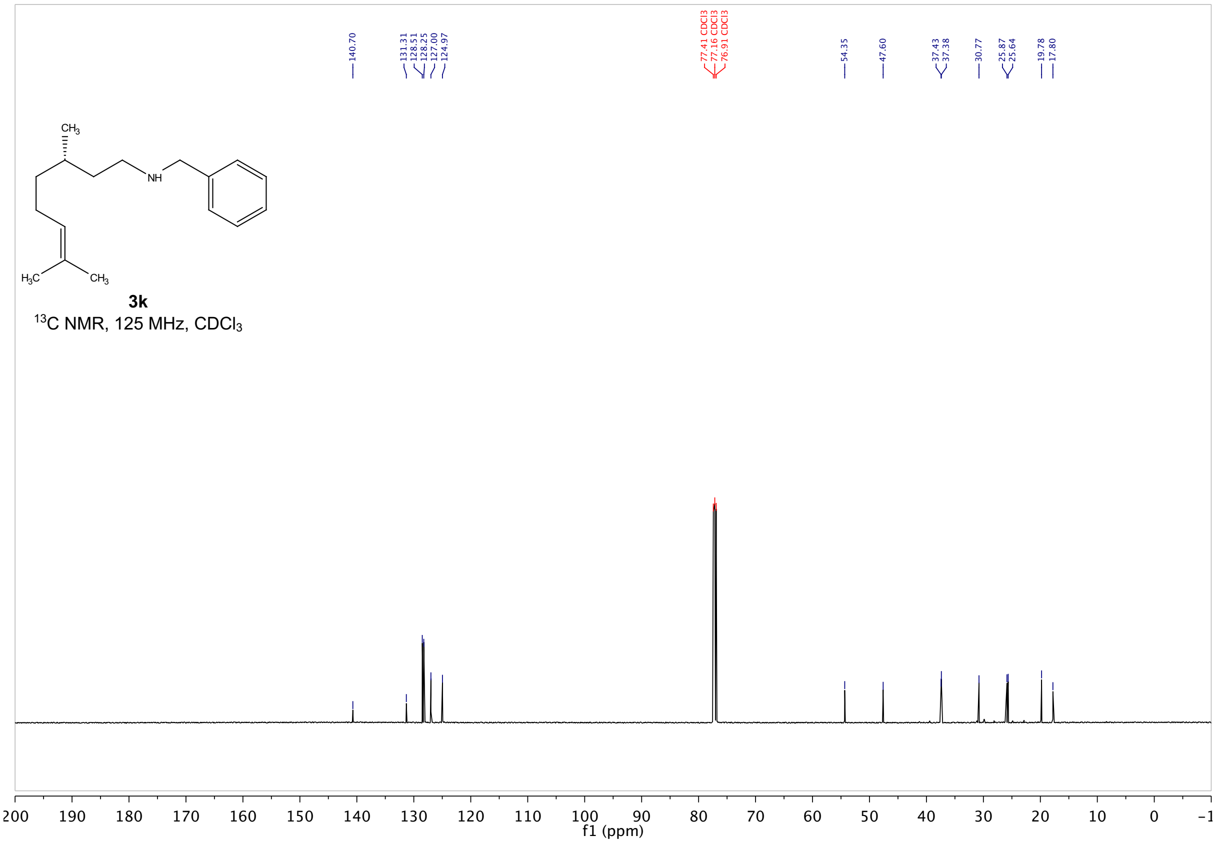


**Supplementary Figure 89.** $^{13}$C NMR spectra of compound **3k**

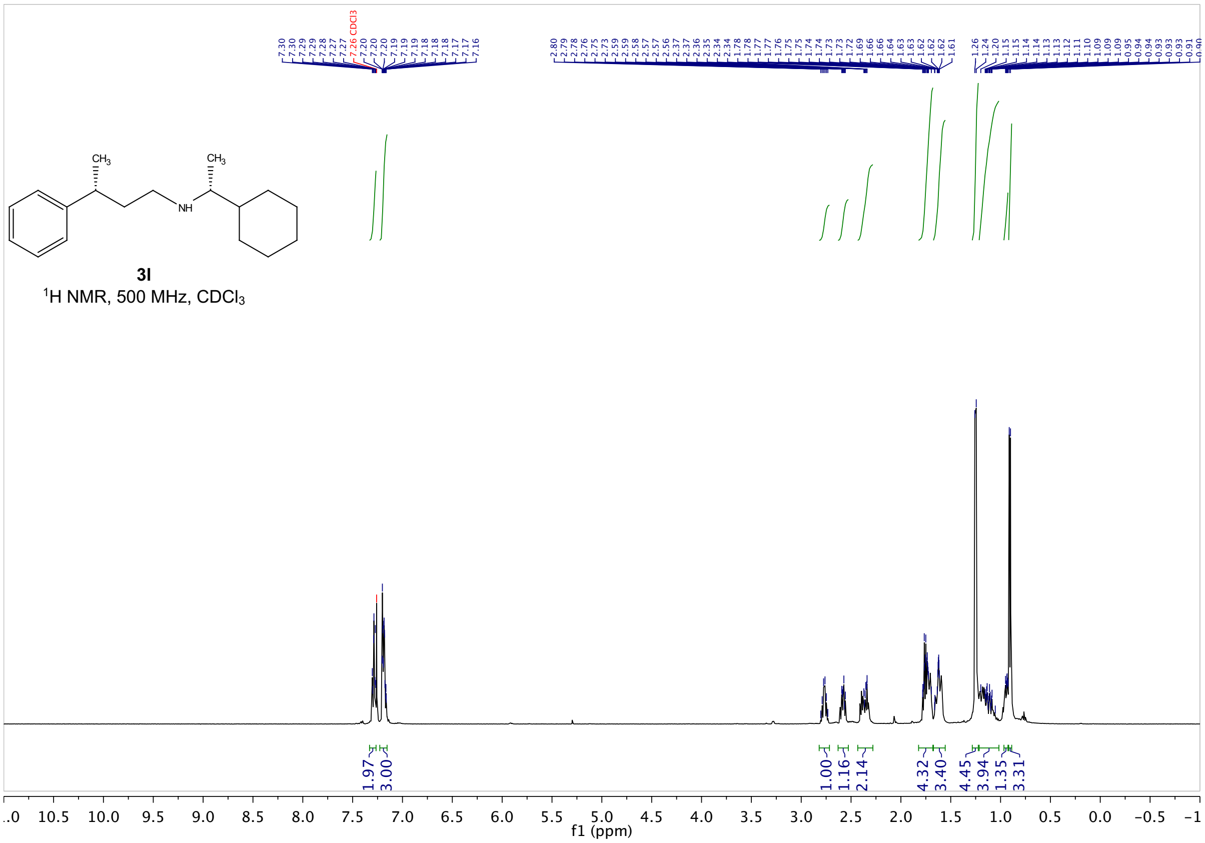


**Supplementary Figure 90.** $^{1}$H NMR spectra of compound **3l**

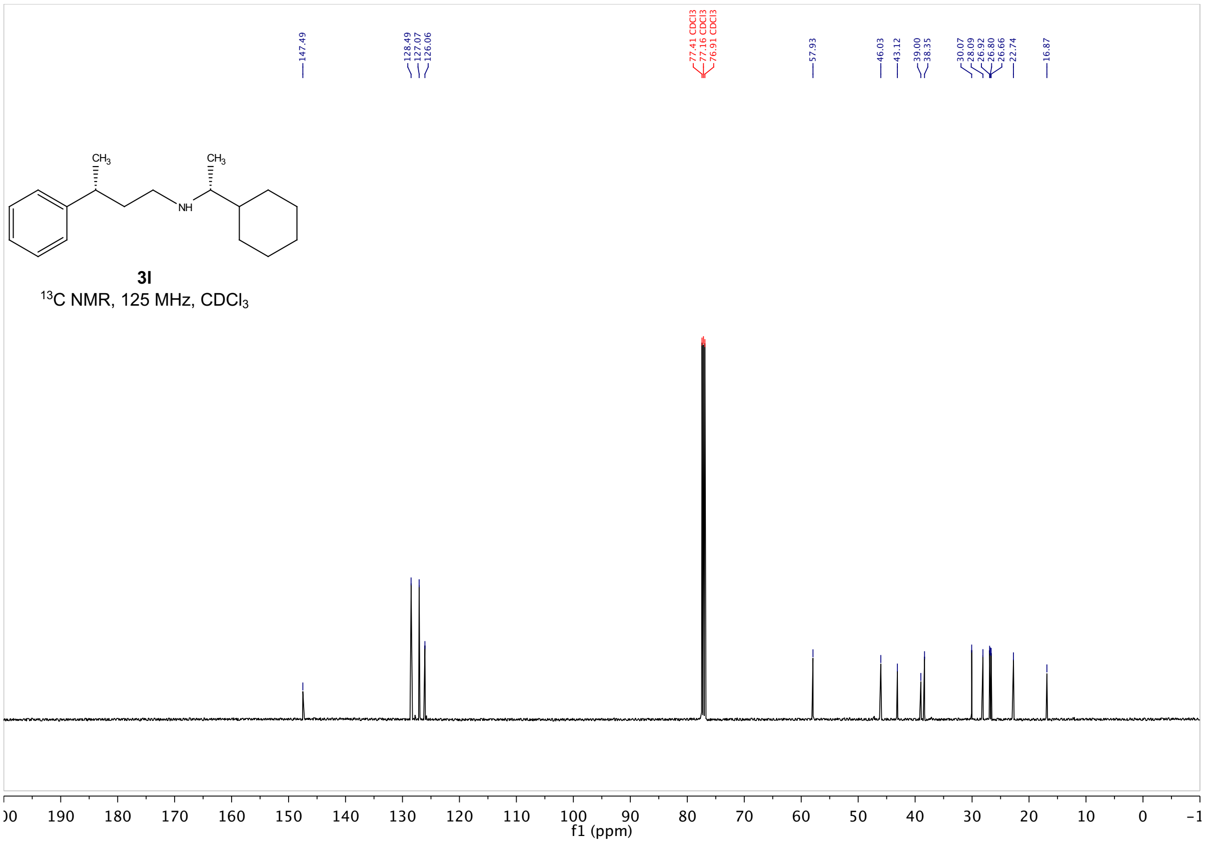


**Supplementary Figure 91.** $^{13}$C NMR spectra of compound **3l**

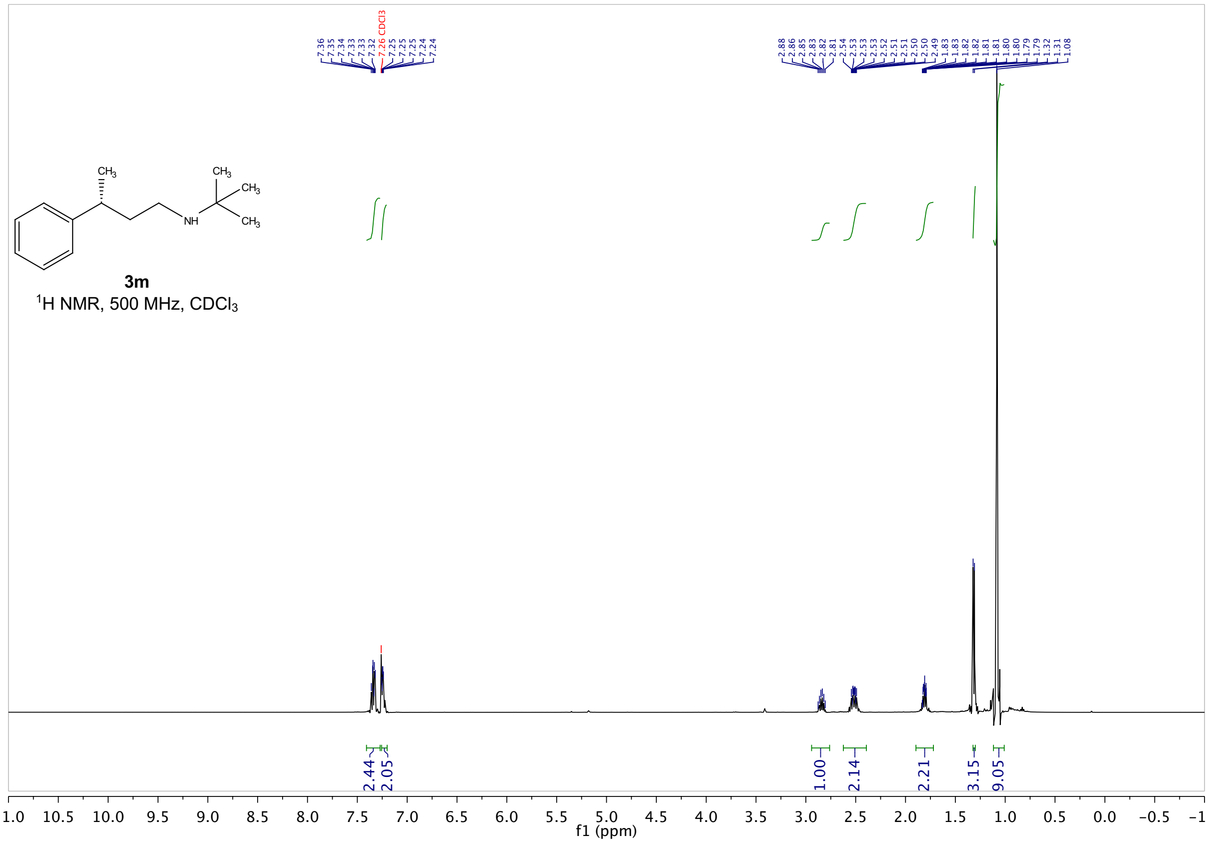


**Supplementary Figure 92.** $^{1}$H NMR spectra of compound **3m**

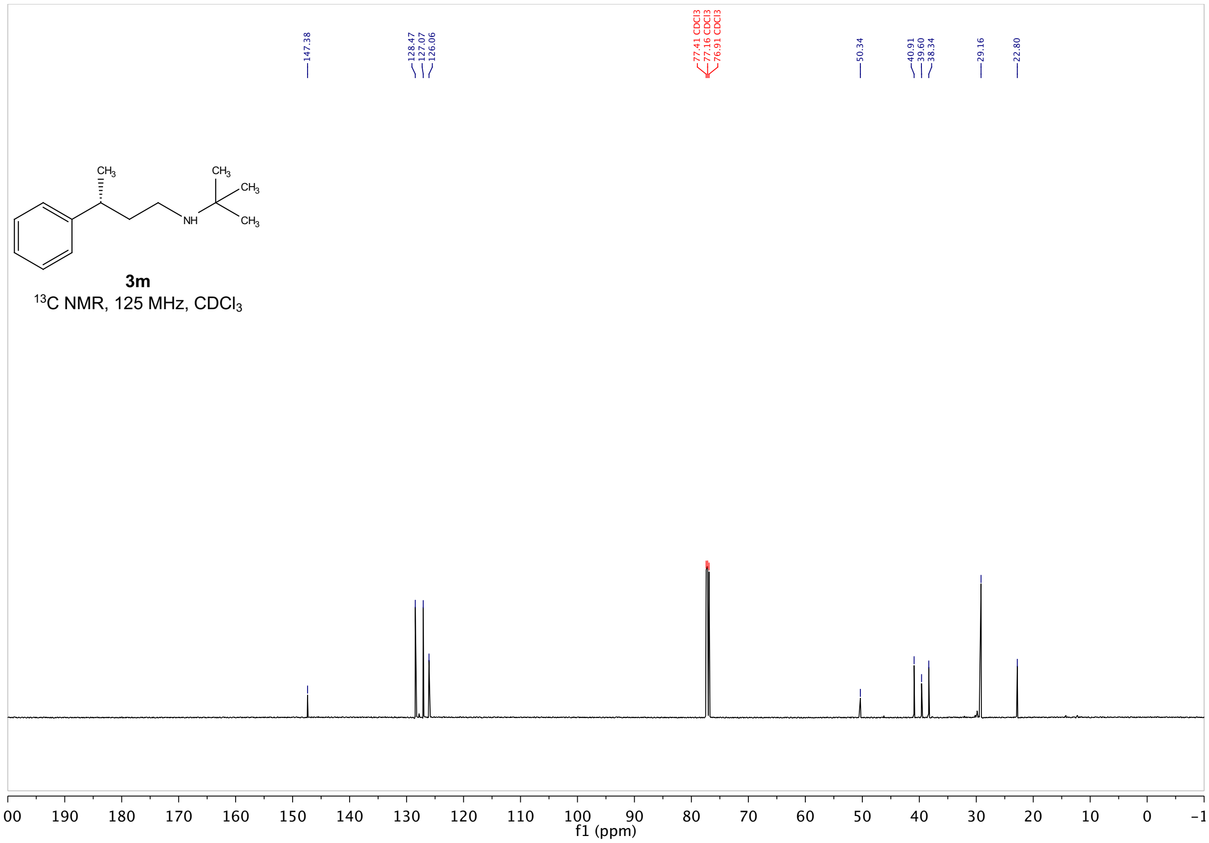


**Supplementary Figure 93.** $^{13}$C NMR spectra of compound **3m**

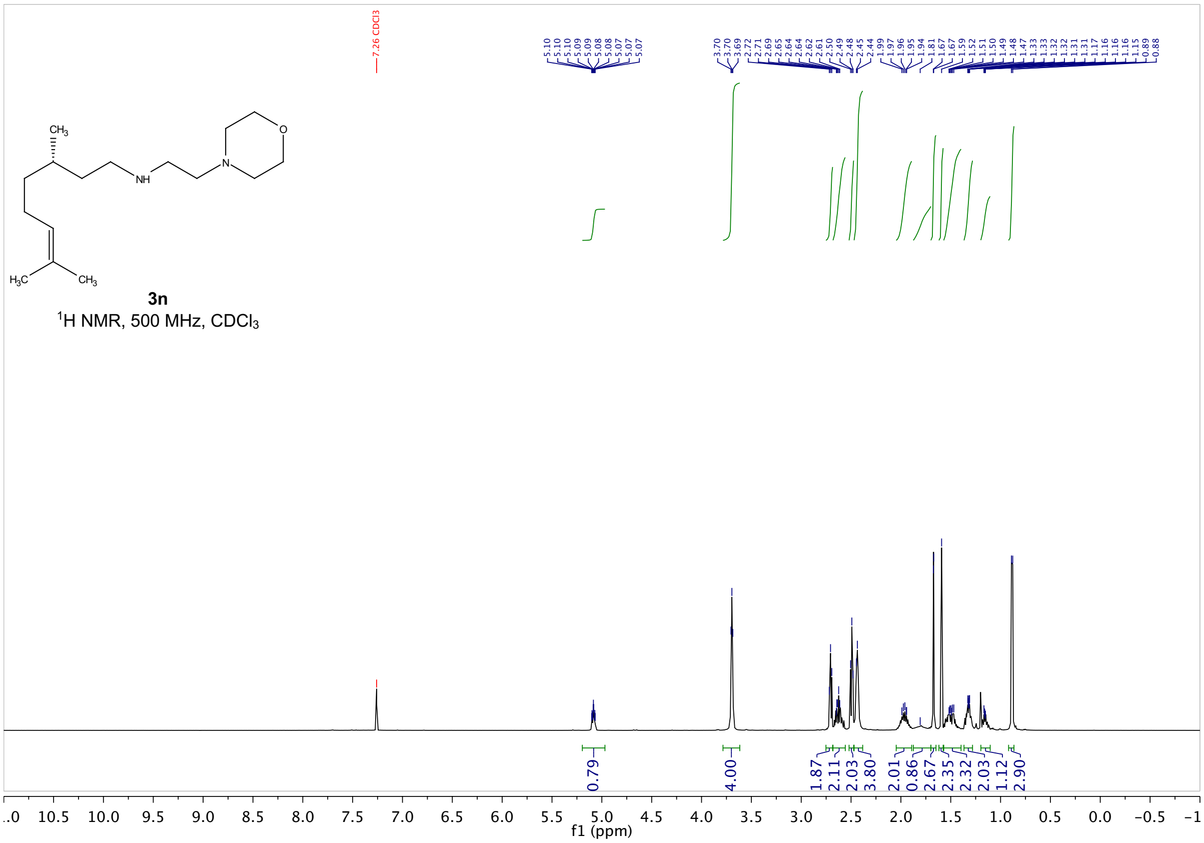


**Supplementary Figure 94.** $^{1}$H NMR spectra of compound **3n**

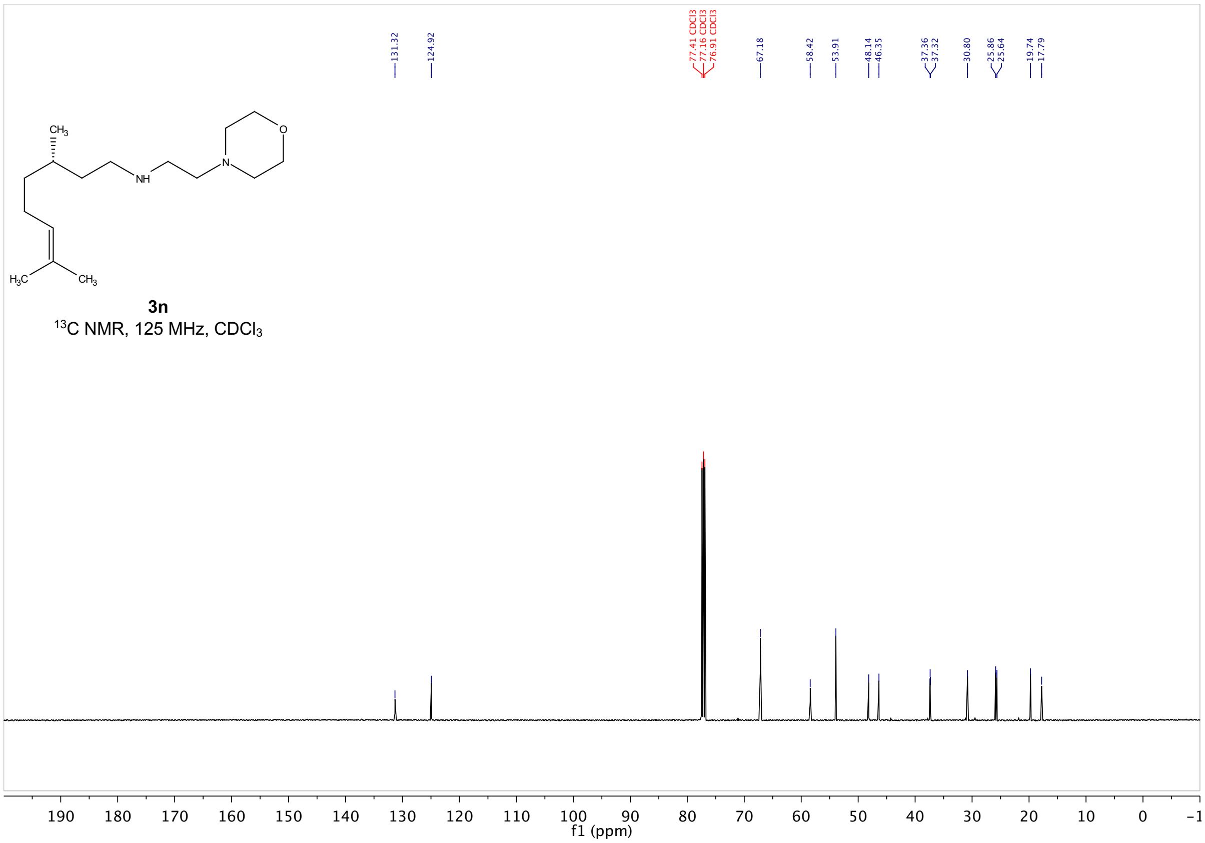


**Supplementary Figure 95.** $^{13}$C NMR spectra of compound **3n**

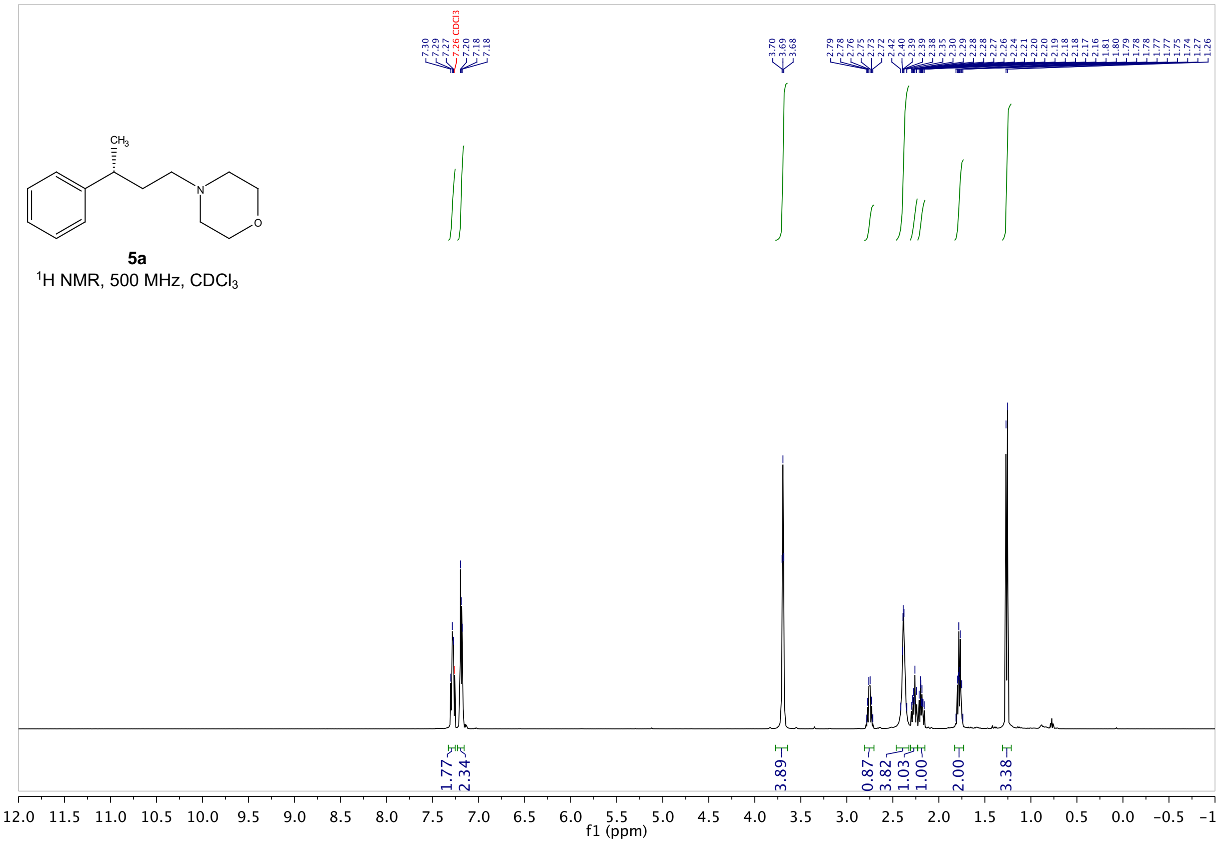


**Supplementary Figure 95.** $^{1}$H NMR spectra of compound **5a**

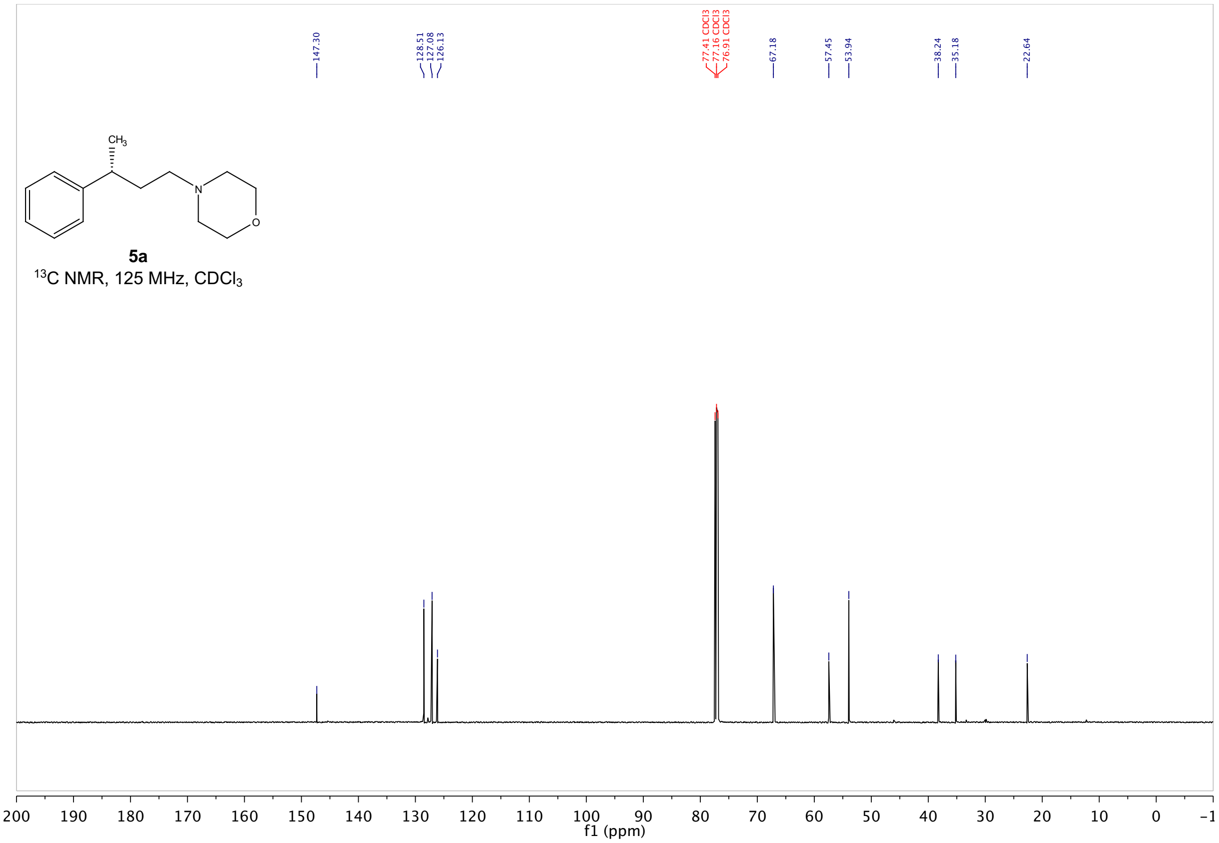


**Supplementary Figure 96.** $^{13}$C NMR spectra of compound **5a**

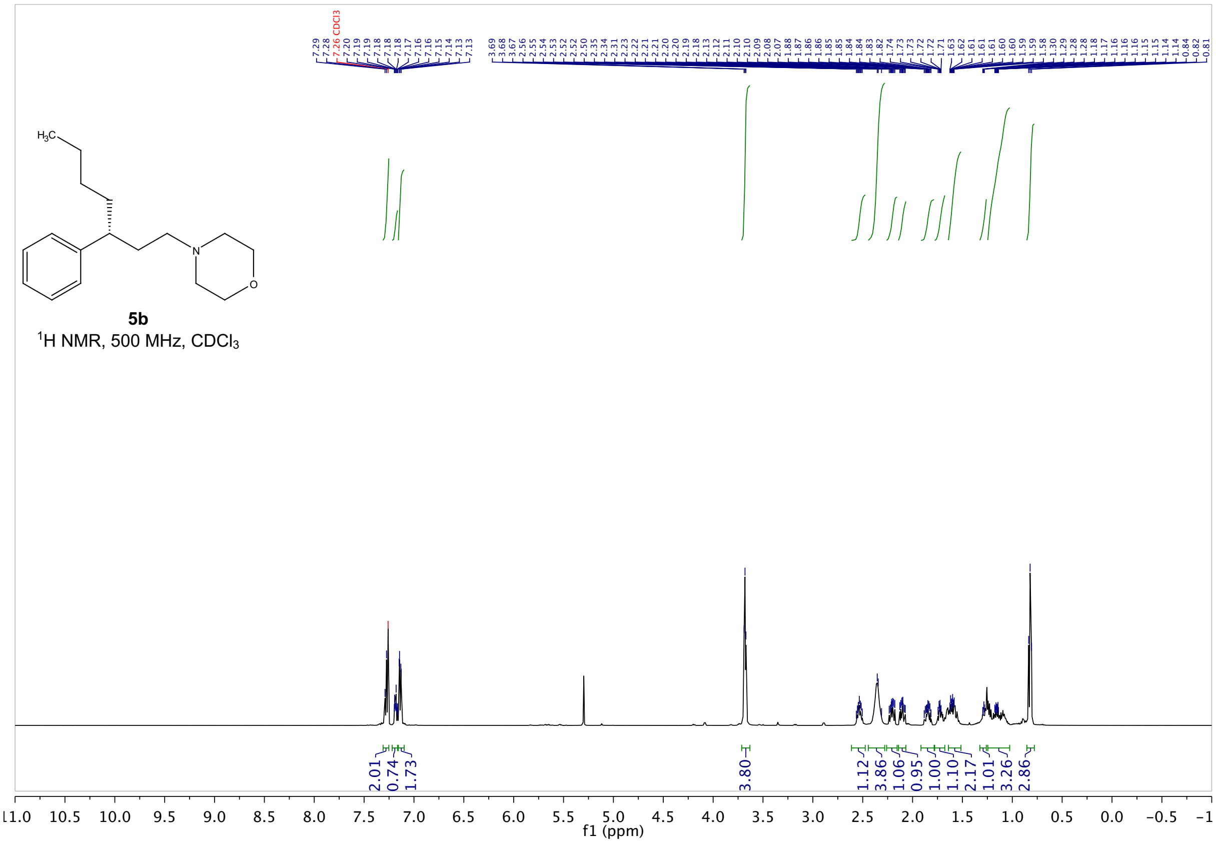


**Supplementary Figure 97.** $^{1}$H NMR spectra of compound **5b**

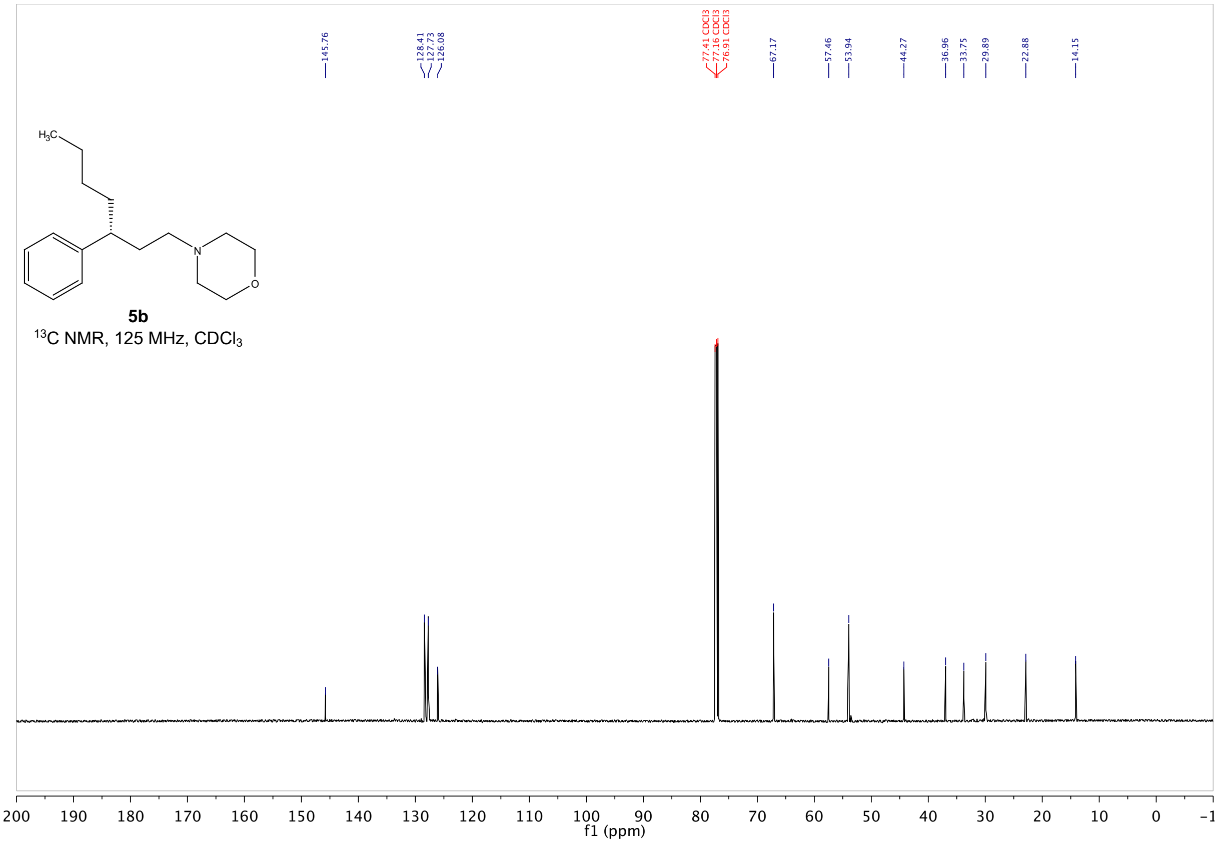


**Supplementary Figure 98.** $^{13}$C NMR spectra of compound **5b**

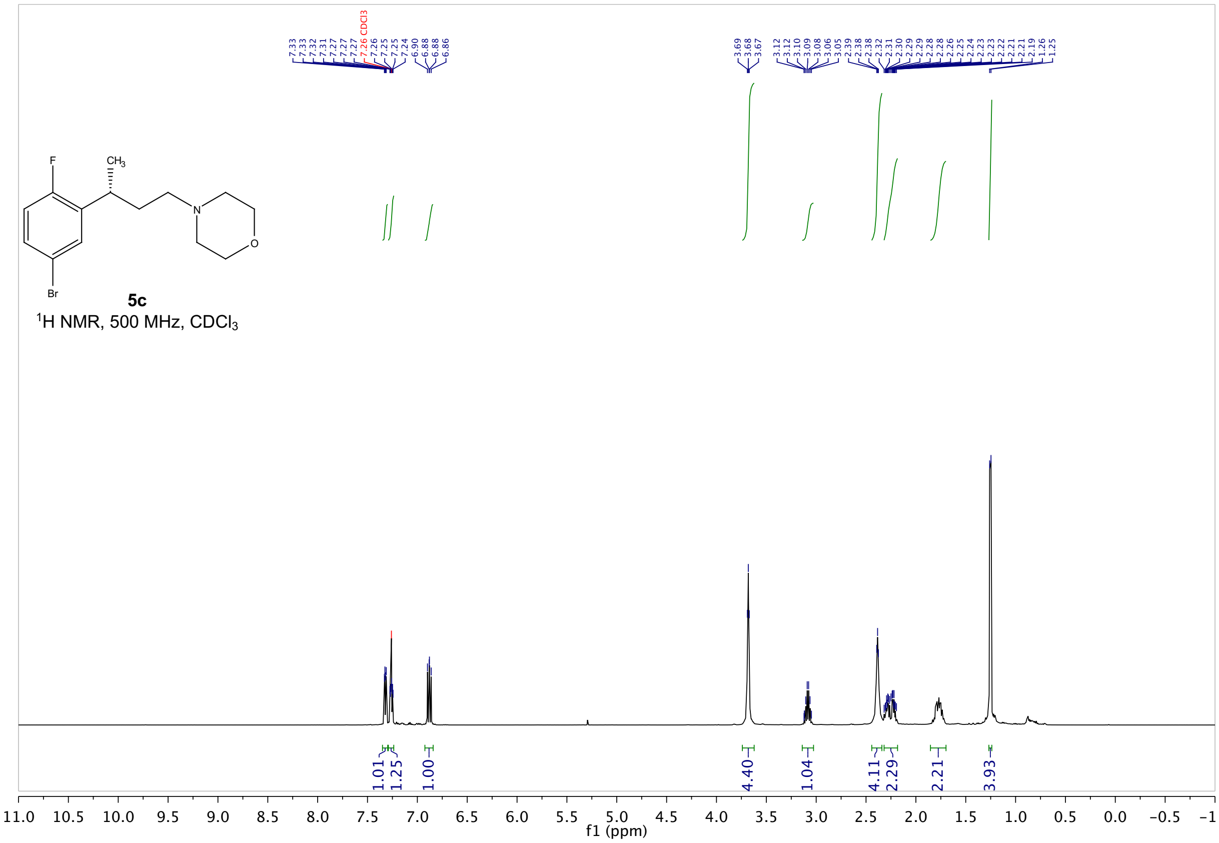


**Supplementary Figure 99.** $^1$H NMR spectra of compound **5c**

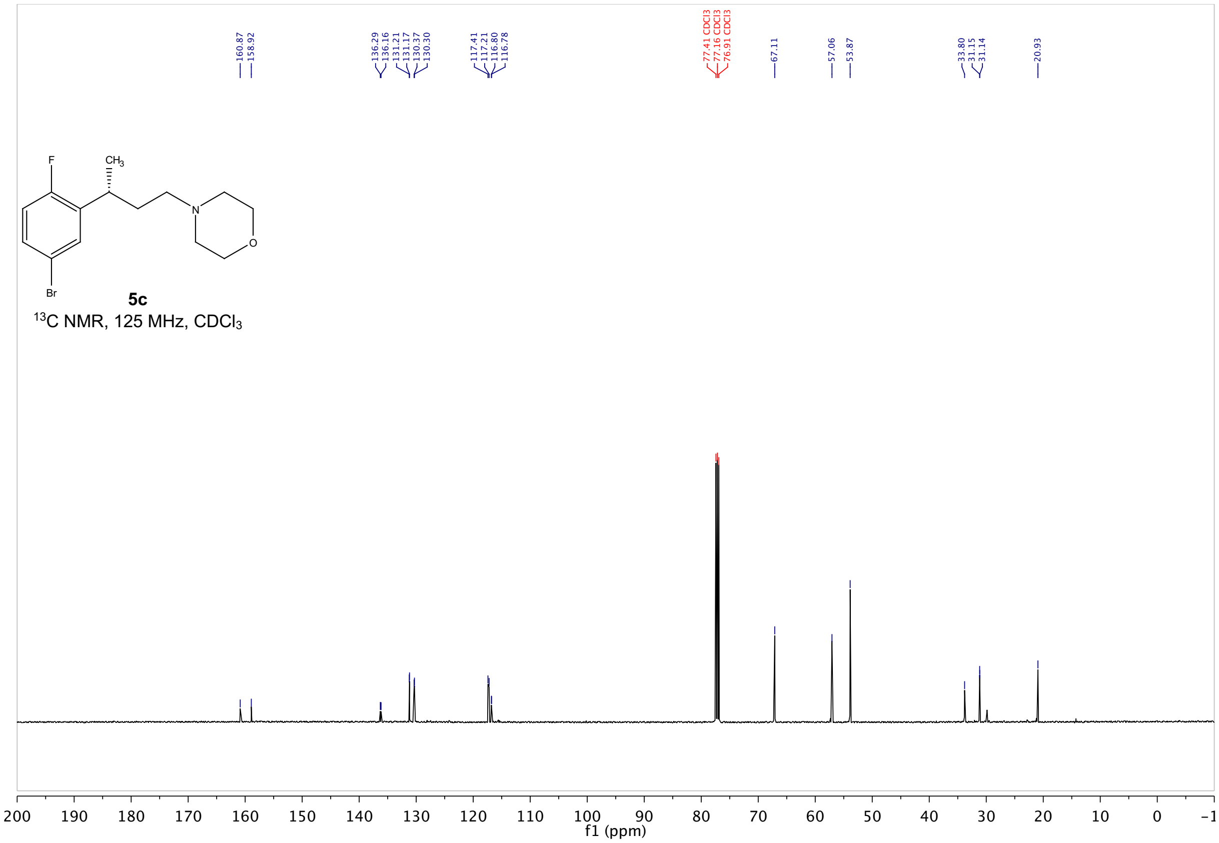


**Supplementary Figure 100.** $^{13}$C NMR spectra of compound **5c**

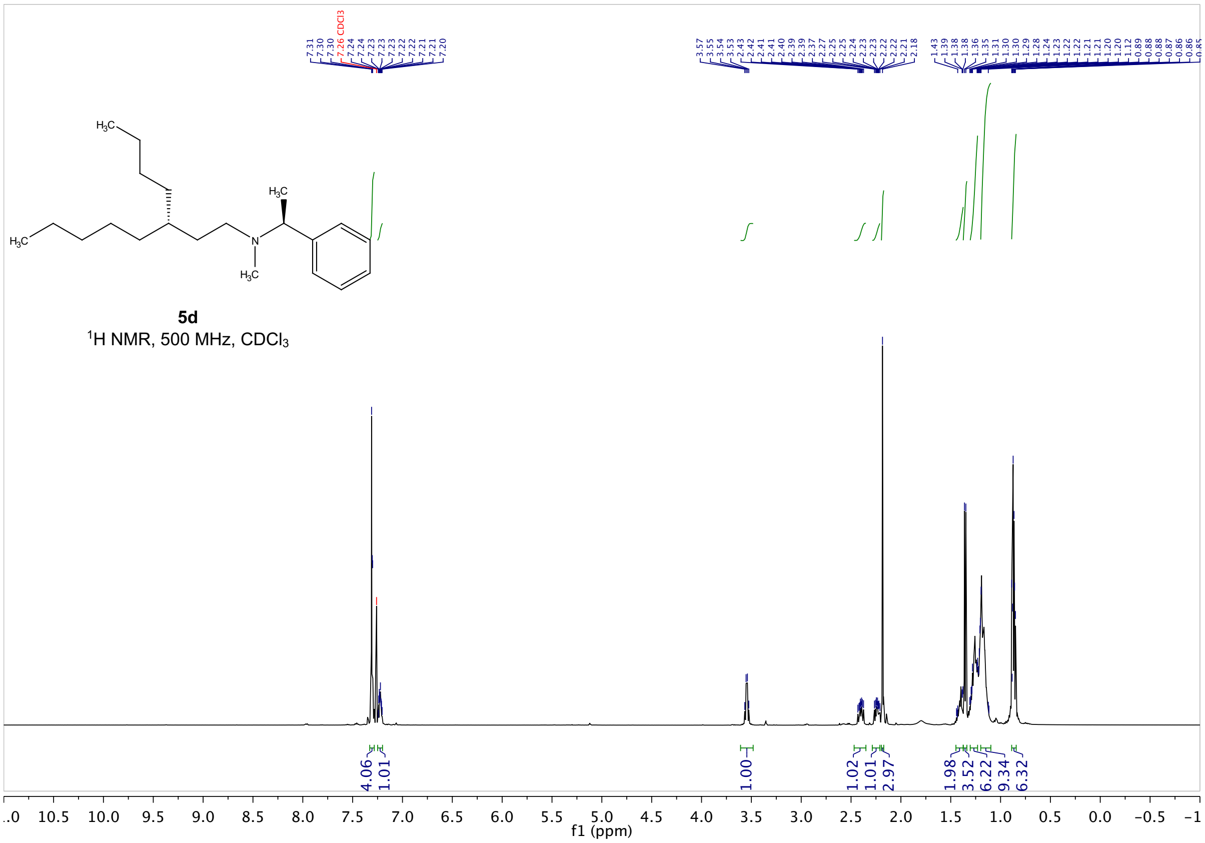


**Supplementary Figure 101.** $^{1}$H NMR spectra of compound **5d**

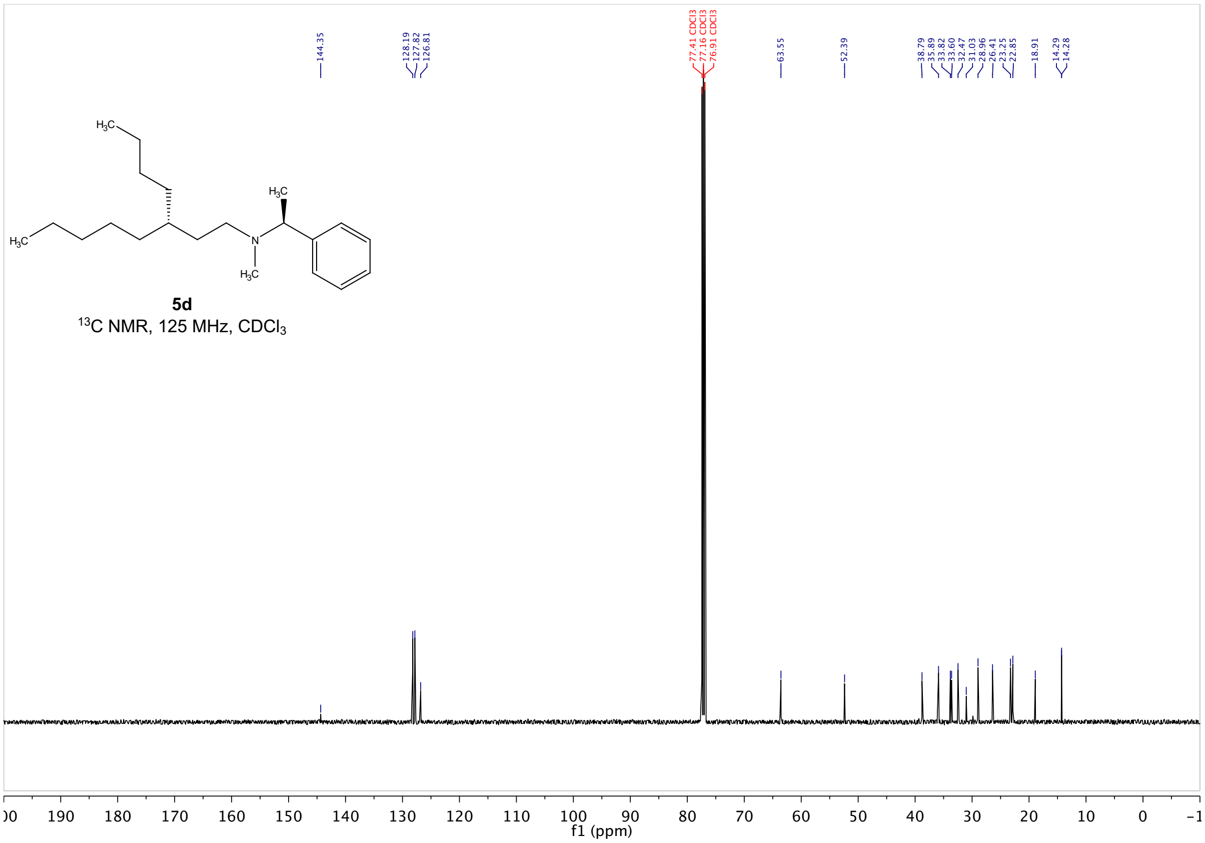


**Supplementary Figure 102.** $^{13}$C NMR spectra of compound **5d**

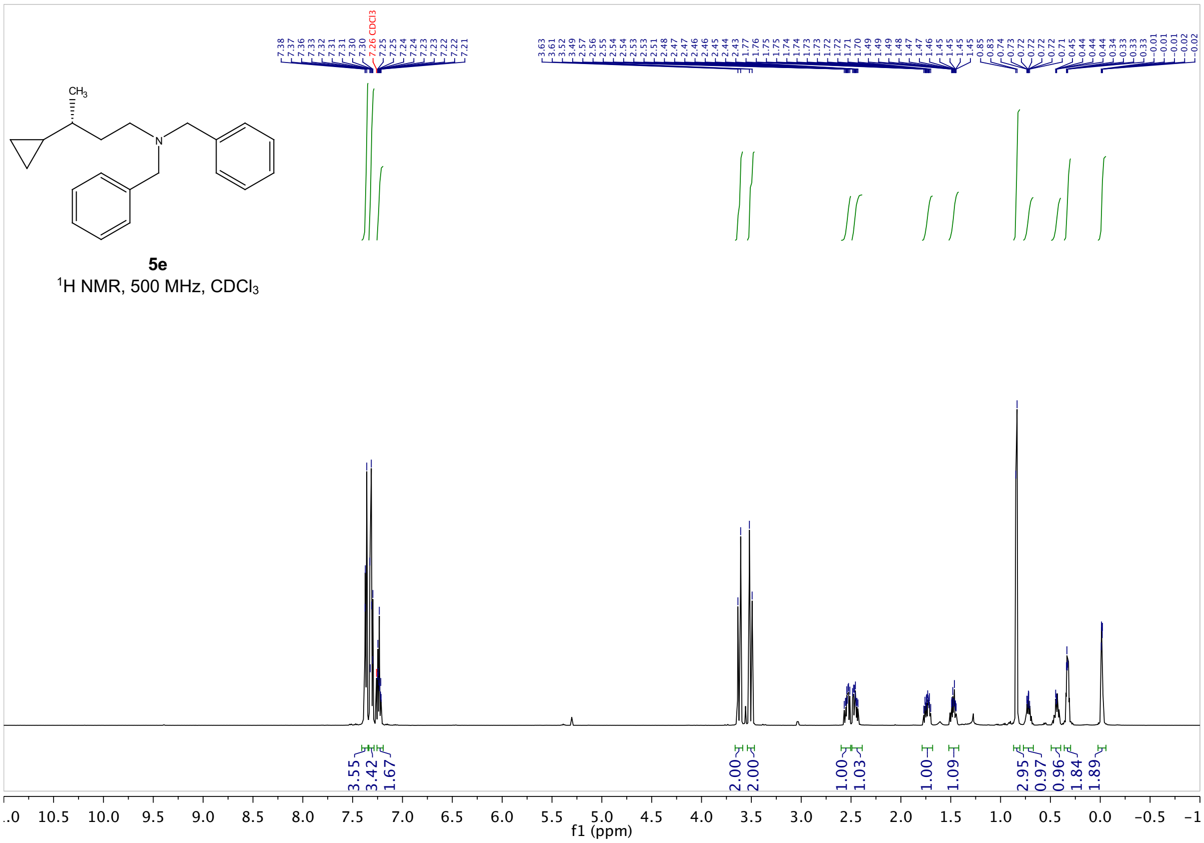

**Supplementary Figure 103.** $^{1}$H NMR spectra of compound **5e**

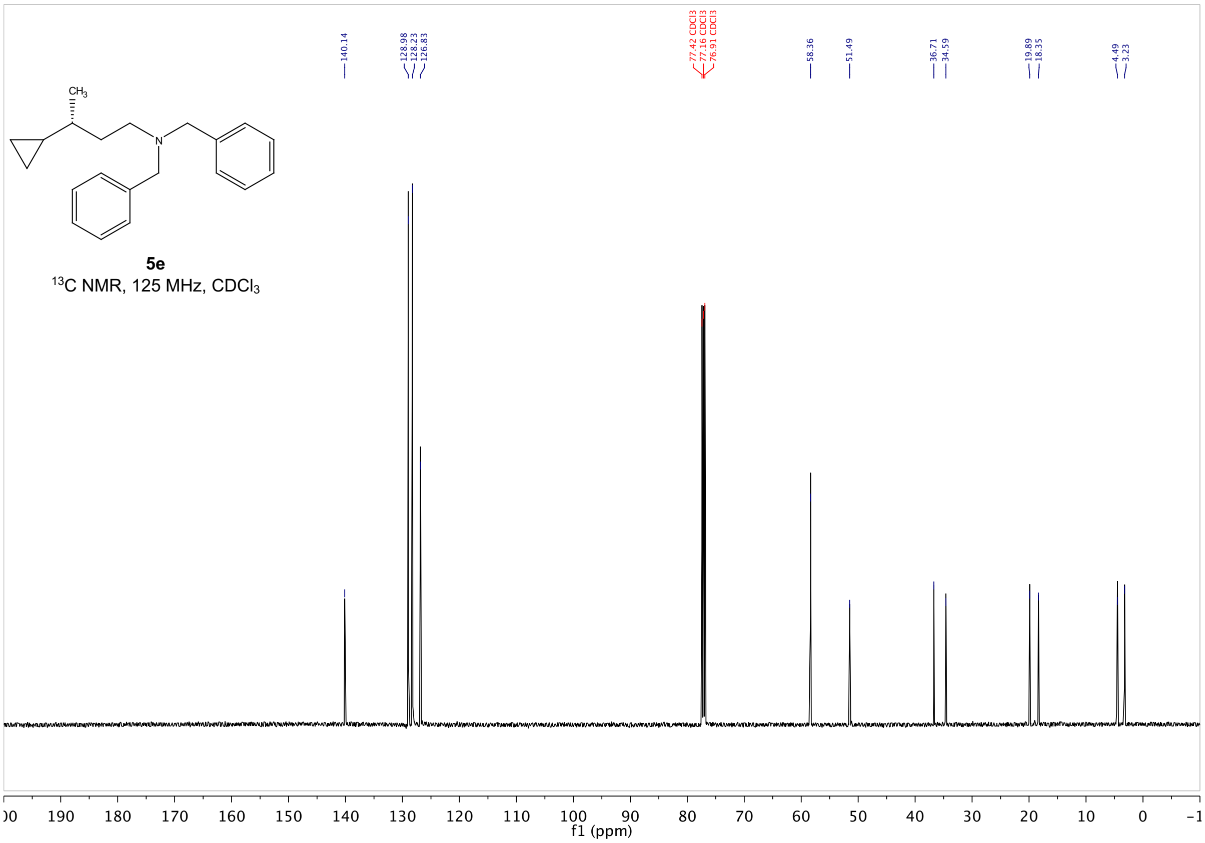

**Supplementary Figure 104.** $^{13}$C NMR spectra of compound **5e**

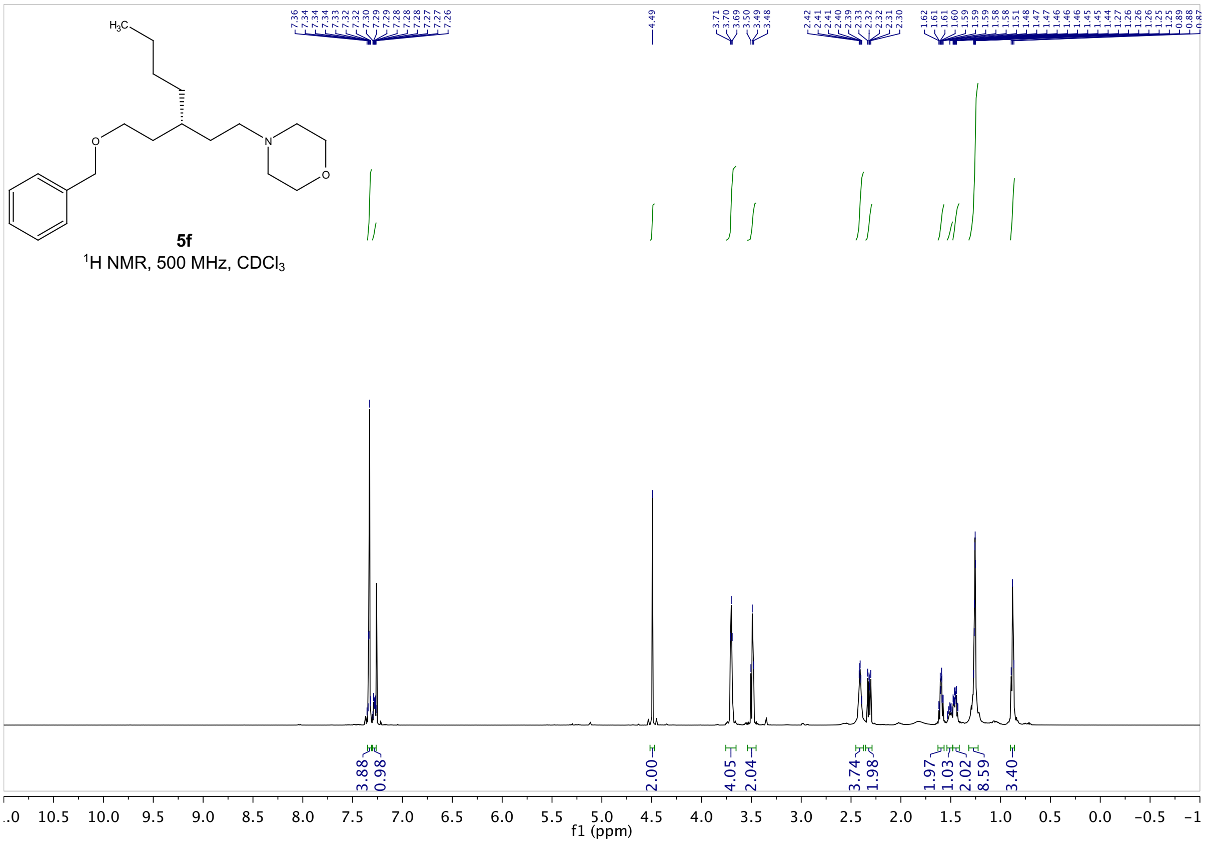


**Supplementary Figure 105.** $^{1}$H NMR spectra of compound **5f**

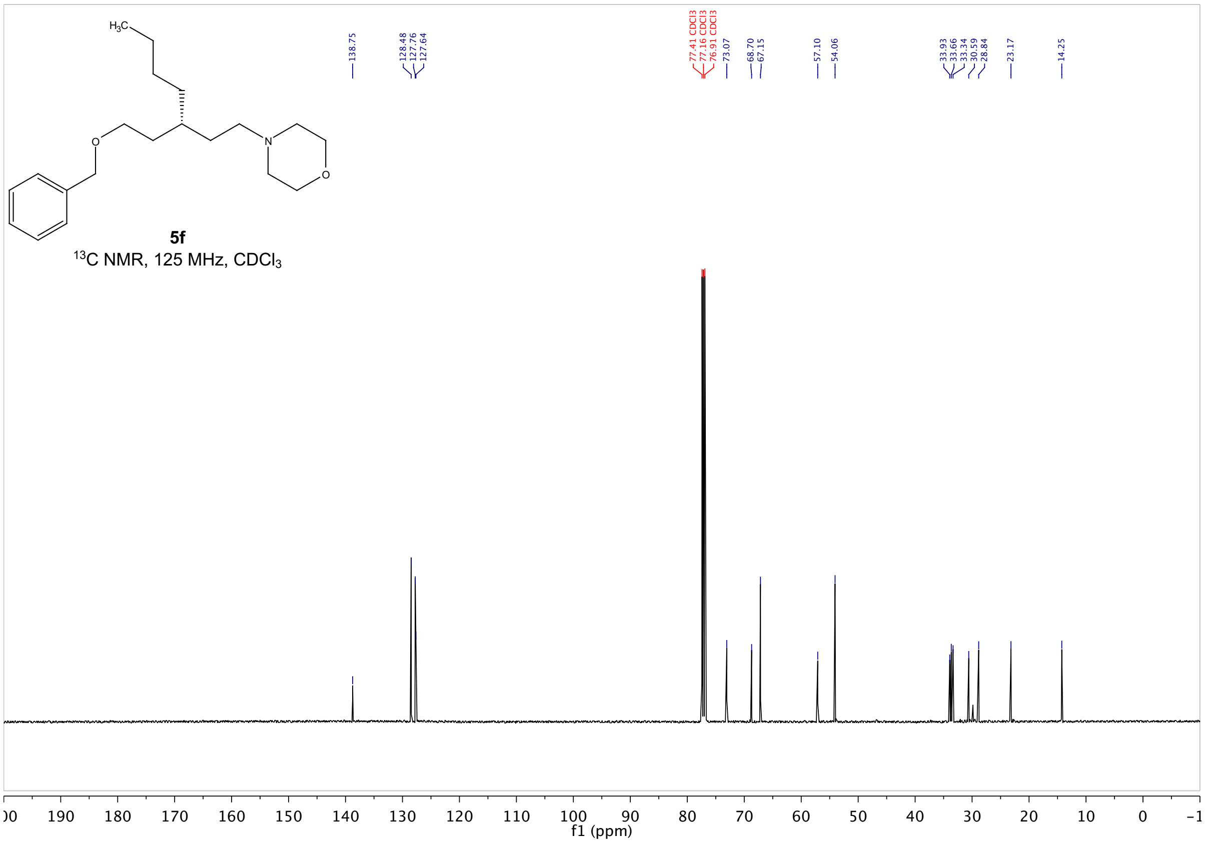


**Supplementary Figure 106.** $^{13}$C NMR spectra of compound **5f**

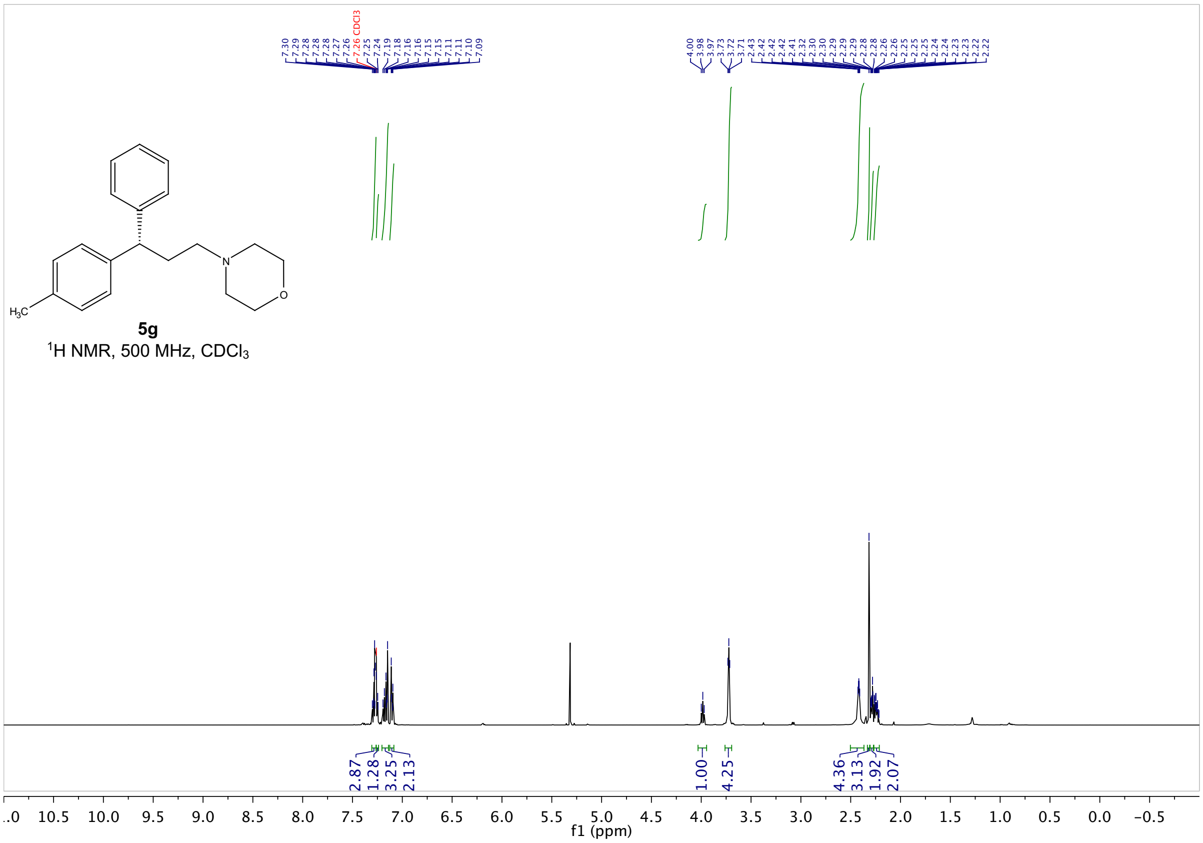


**Supplementary Figure 107.** $^1$H NMR spectra of compound **5g**

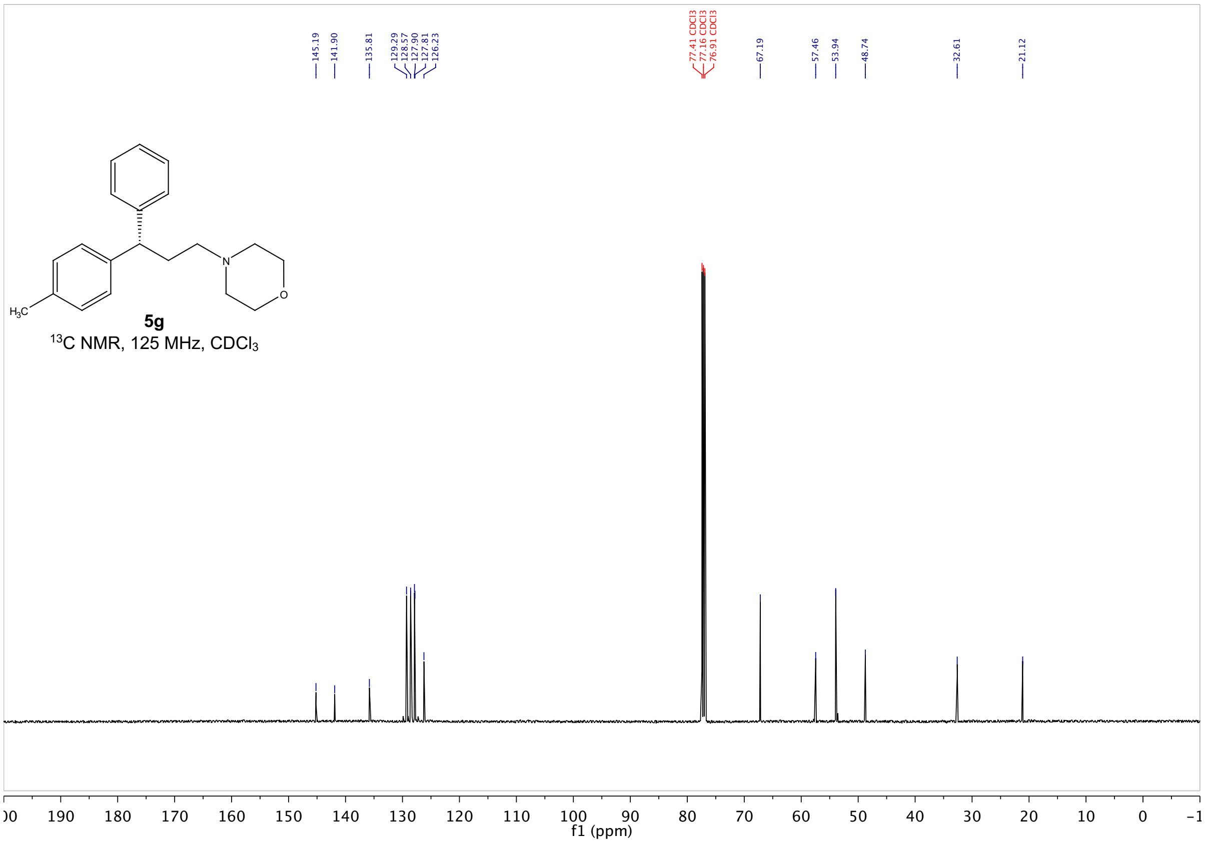


**Supplementary Figure 108.** $^{13}$C NMR spectra of compound **5g**

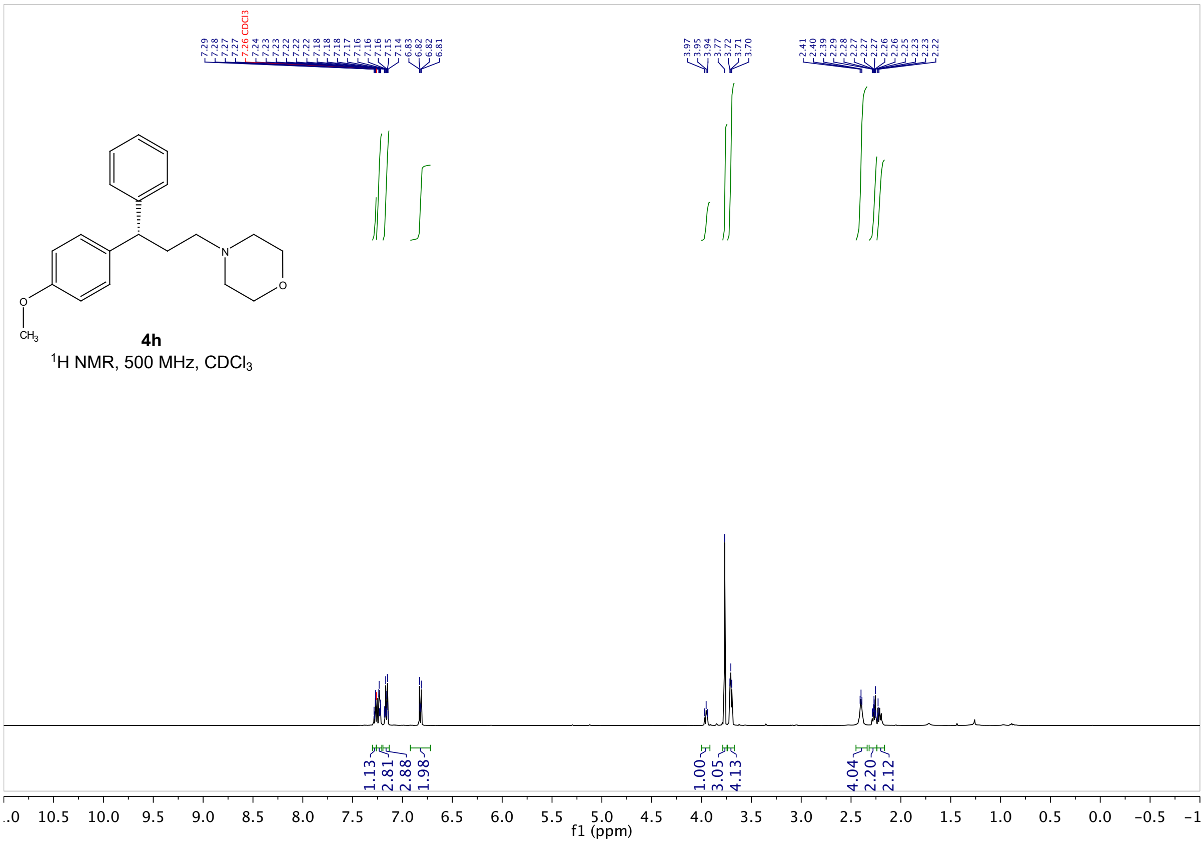


**Supplementary Figure 109.** $^1$H NMR spectra of compound **5h**

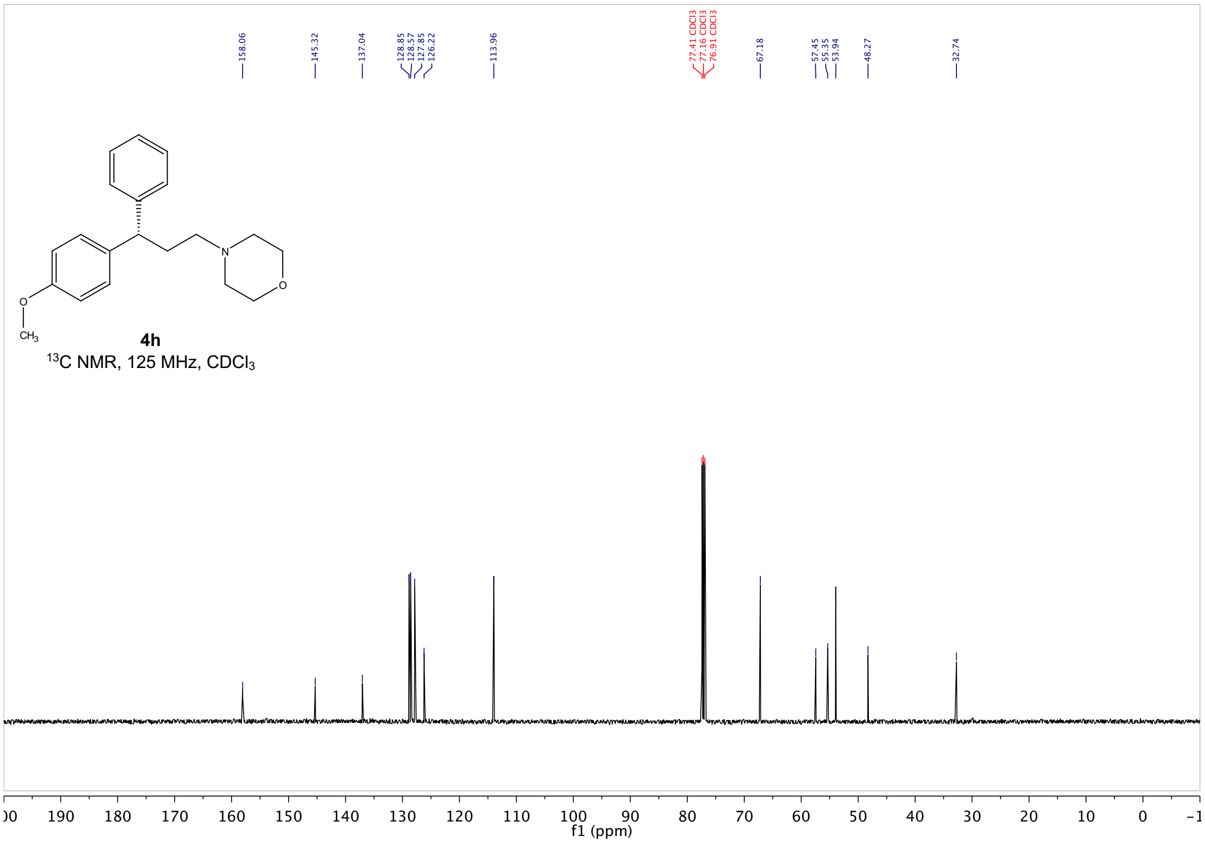


**Supplementary Figure 110.** $^{13}$C NMR spectra of compound **5h**

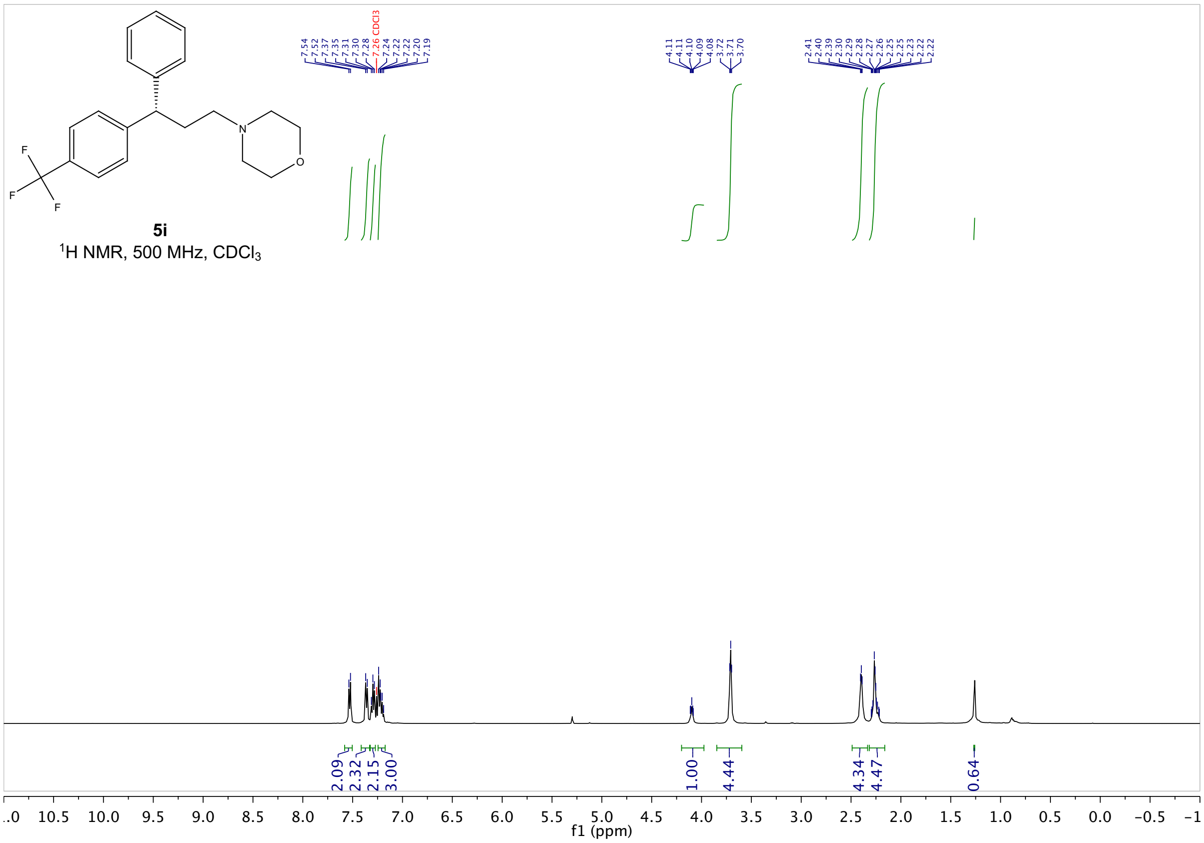


**Supplementary Figure 111.** $^{1}$H NMR spectra of compound **5i**

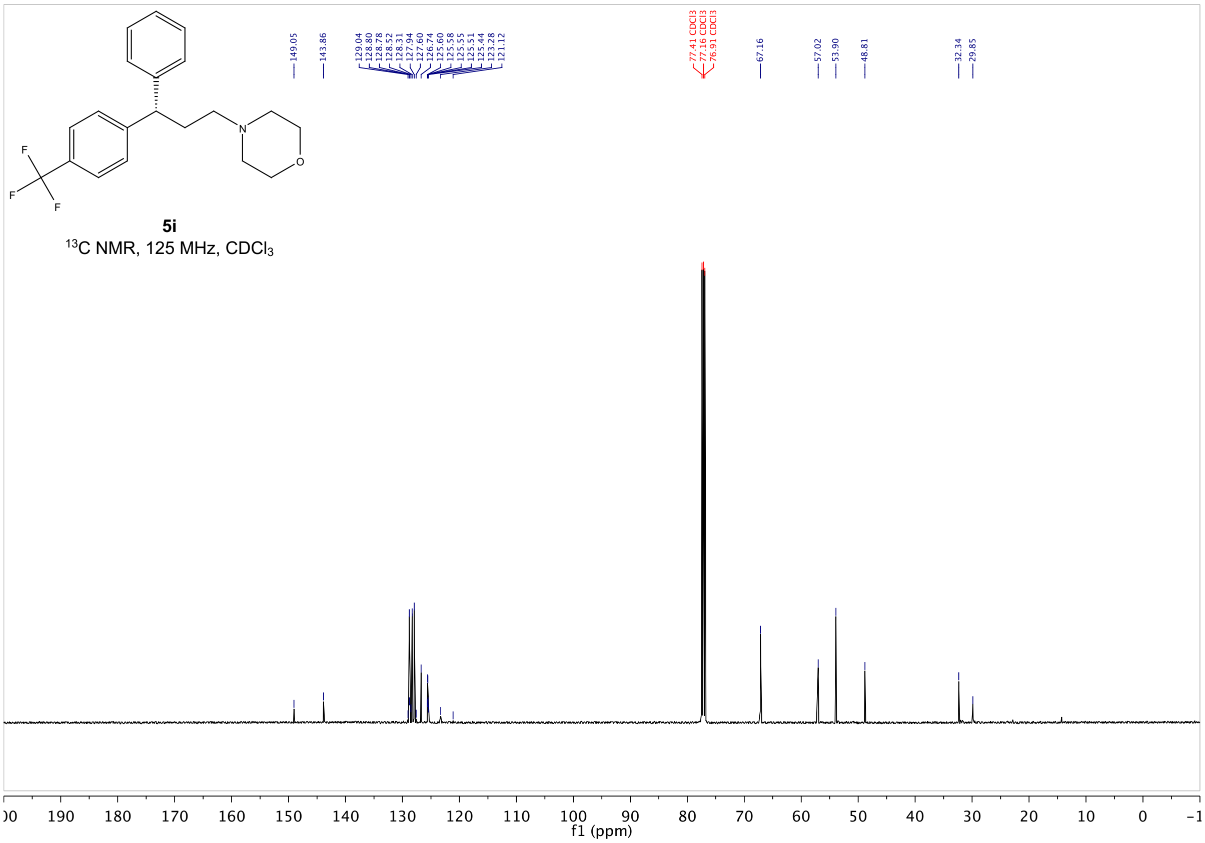


**Supplementary Figure 112.** $^{13}$C NMR spectra of compound **5i**

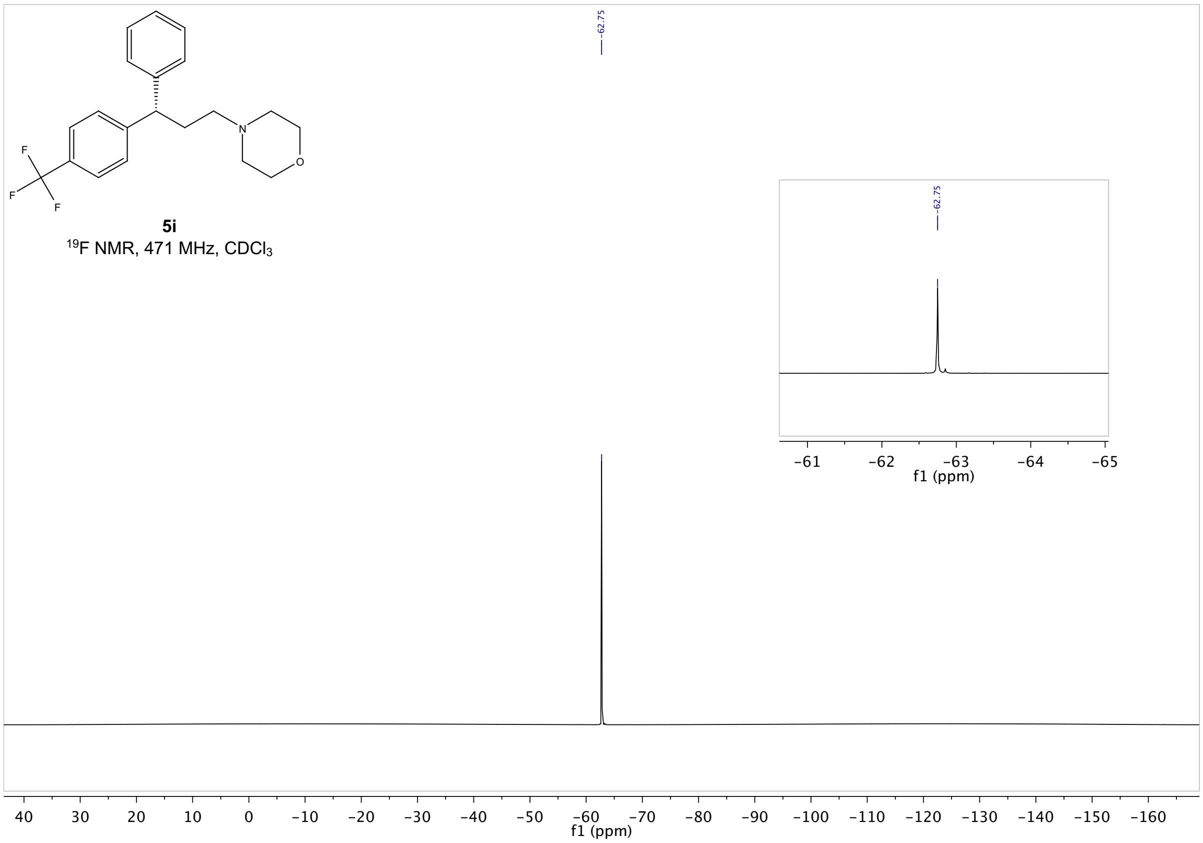


**Supplementary Figure 113.** $^{19}$F NMR spectra of compound **5i**

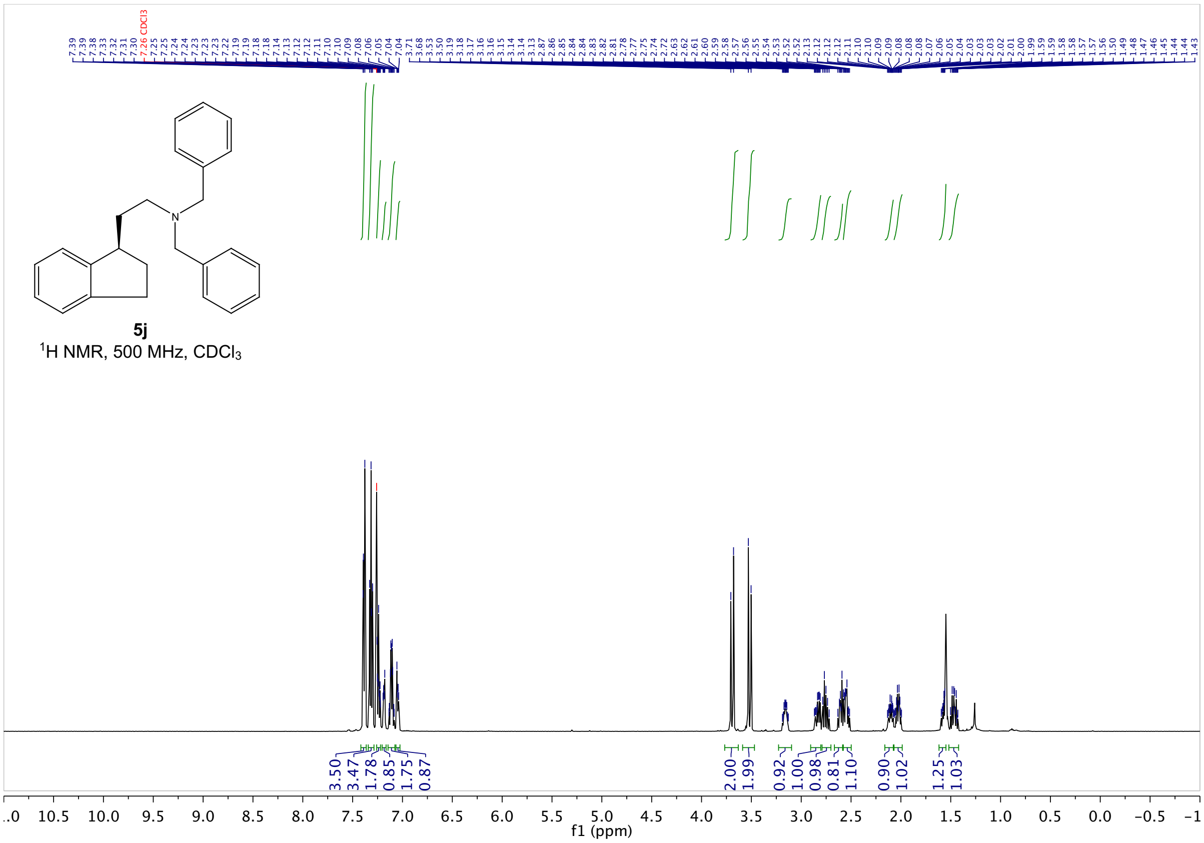


**Supplementary Figure 114.** $^{1}$H NMR spectra of compound **5j**

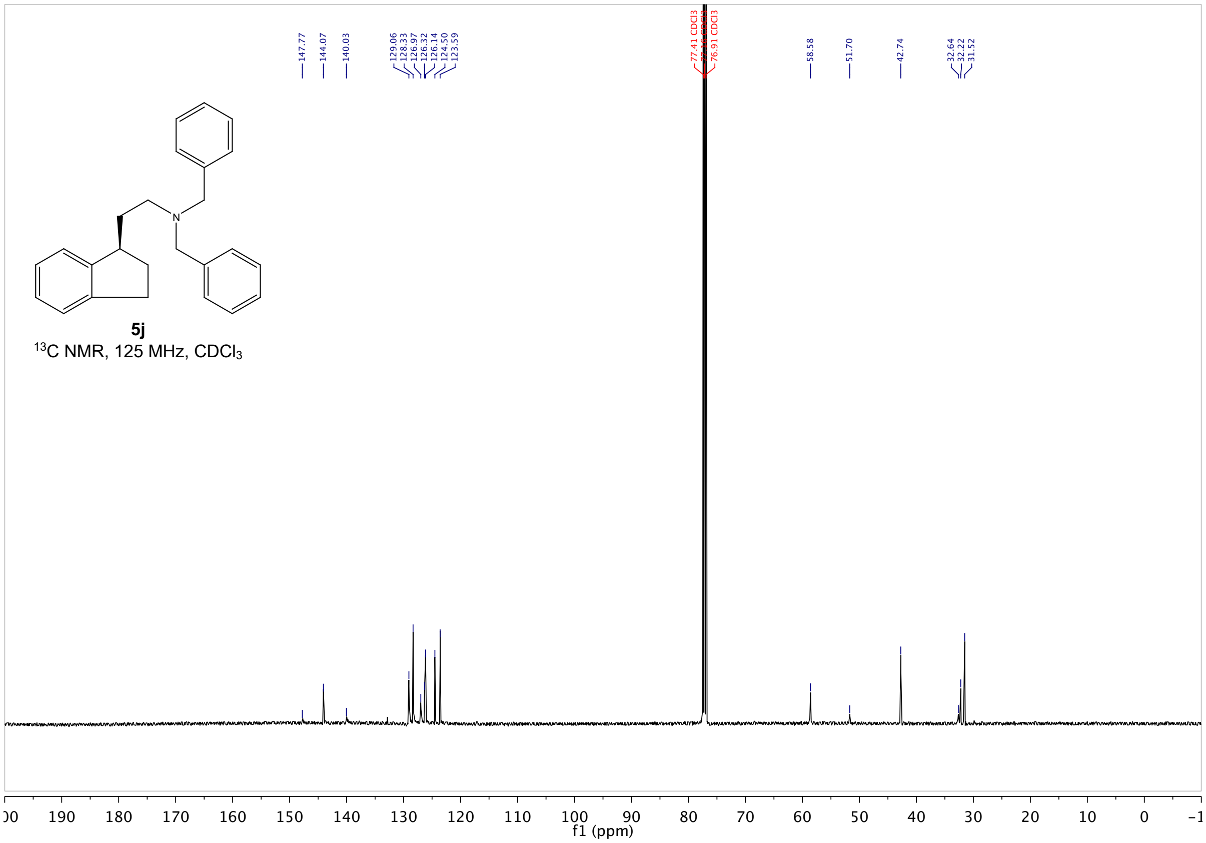


**Supplementary Figure 115.** $^{13}$C NMR spectra of compound **5j**

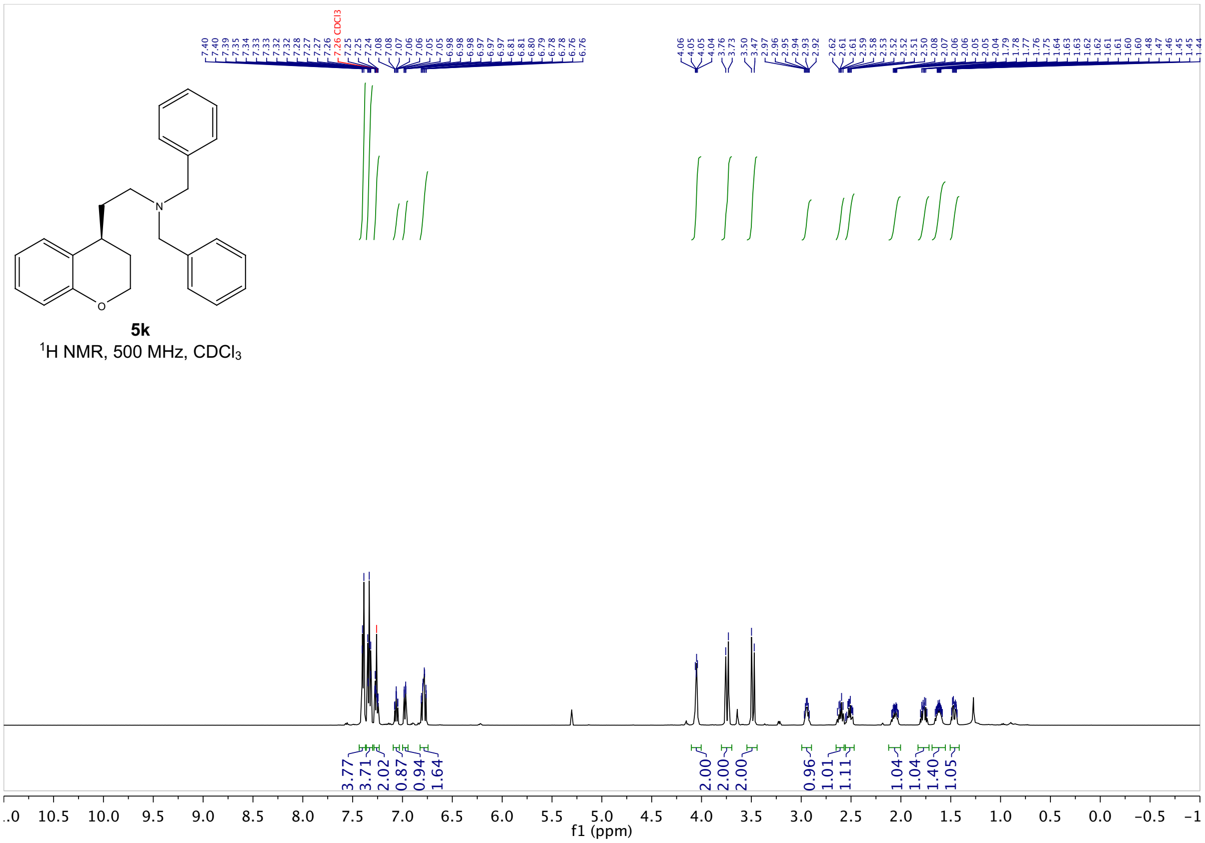


**Supplementary Figure 116.** $^{1}$H NMR spectra of compound **5k**

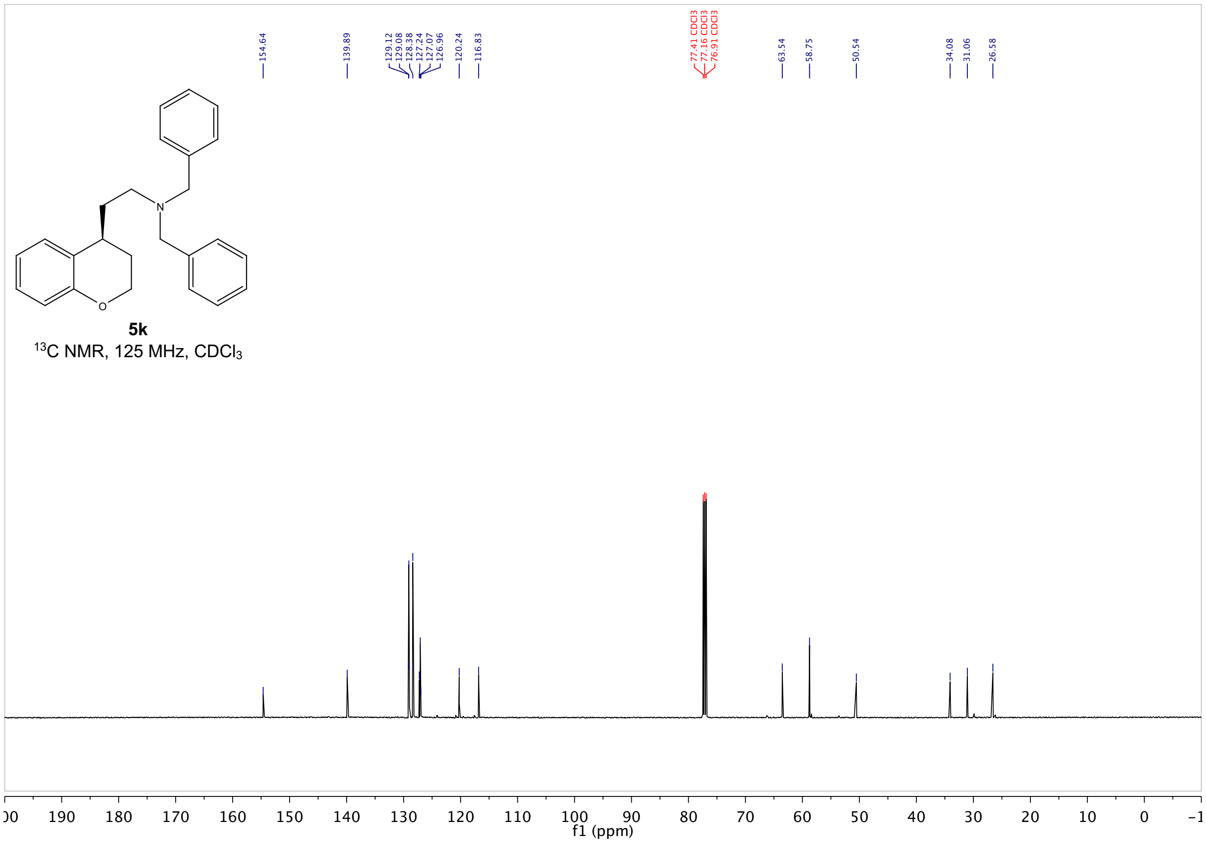


**Supplementary Figure 117.** $^{13}C$ NMR spectra of compound **5k**

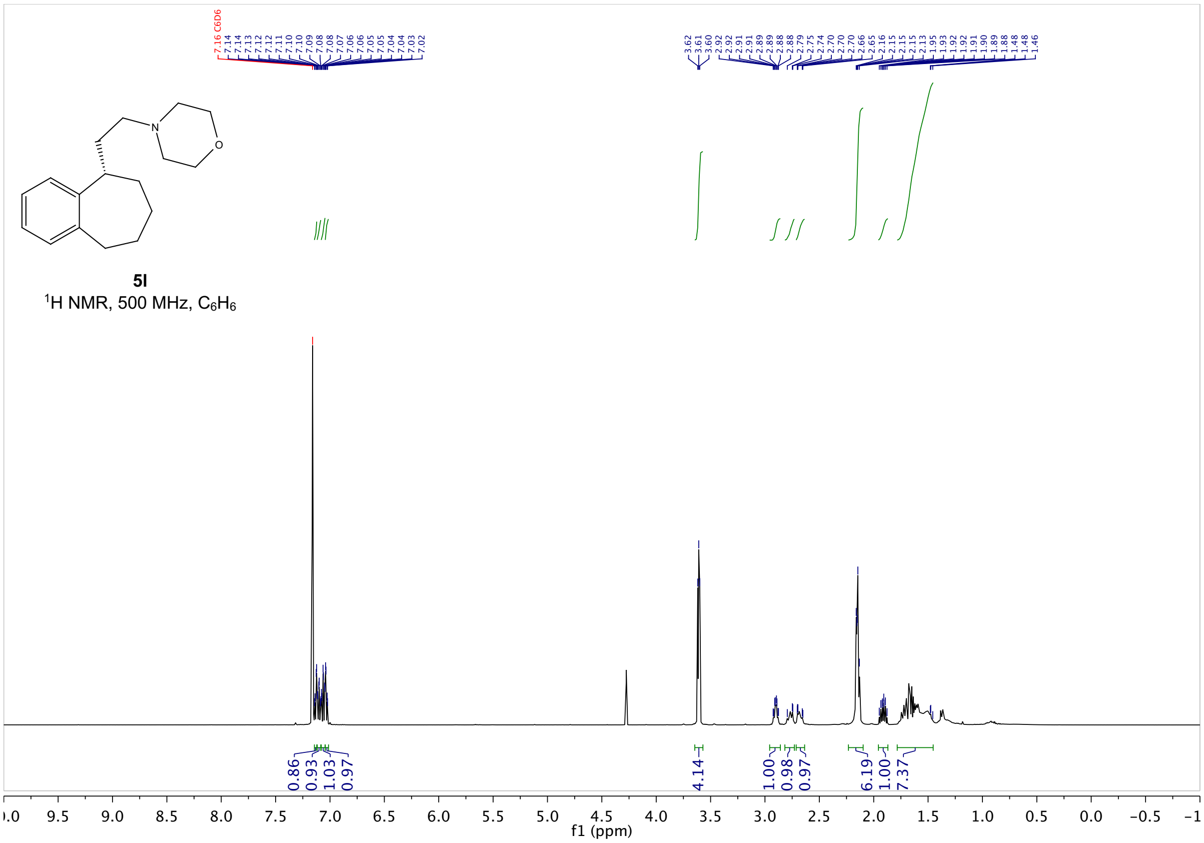


**Supplementary Figure 118.** $^{1}H$ NMR spectra of compound **5l**

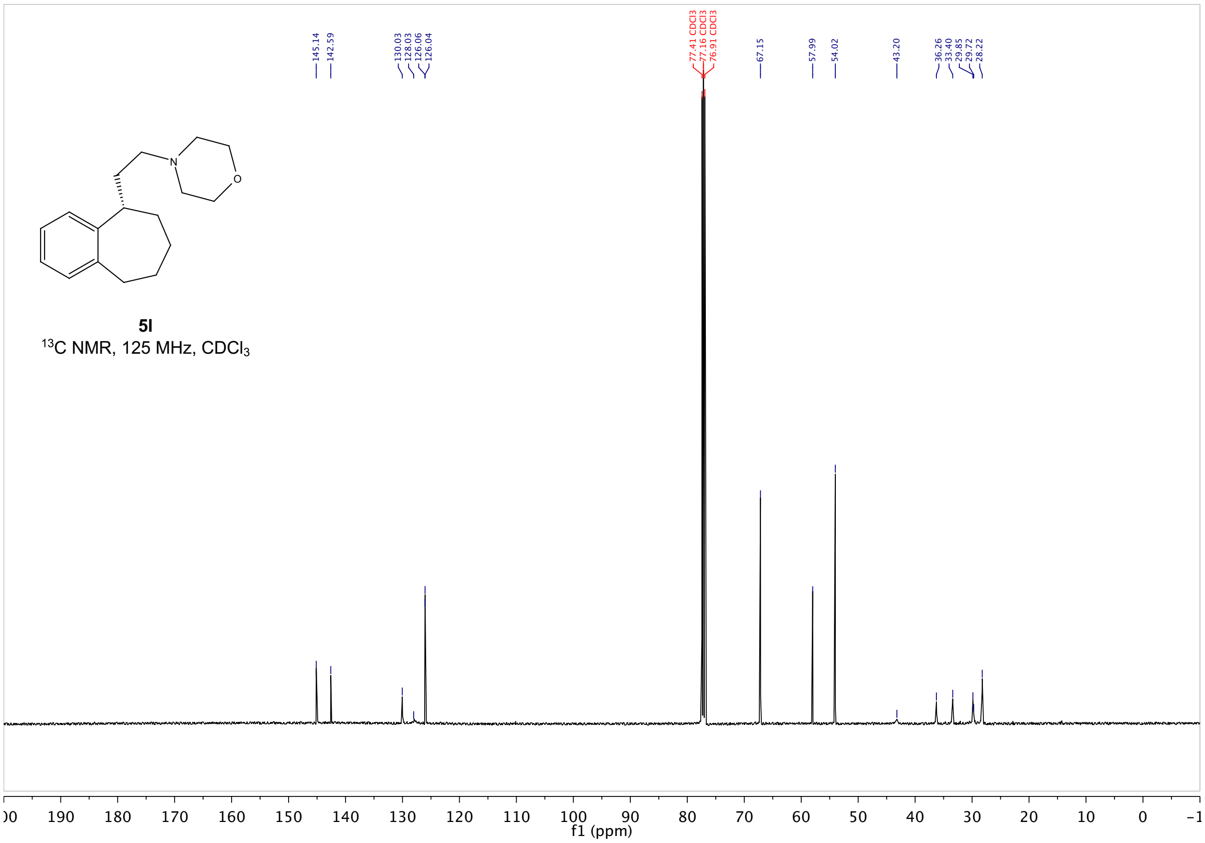


**Supplementary Figure 119.** $^{13}$C NMR spectra of compound **5l**

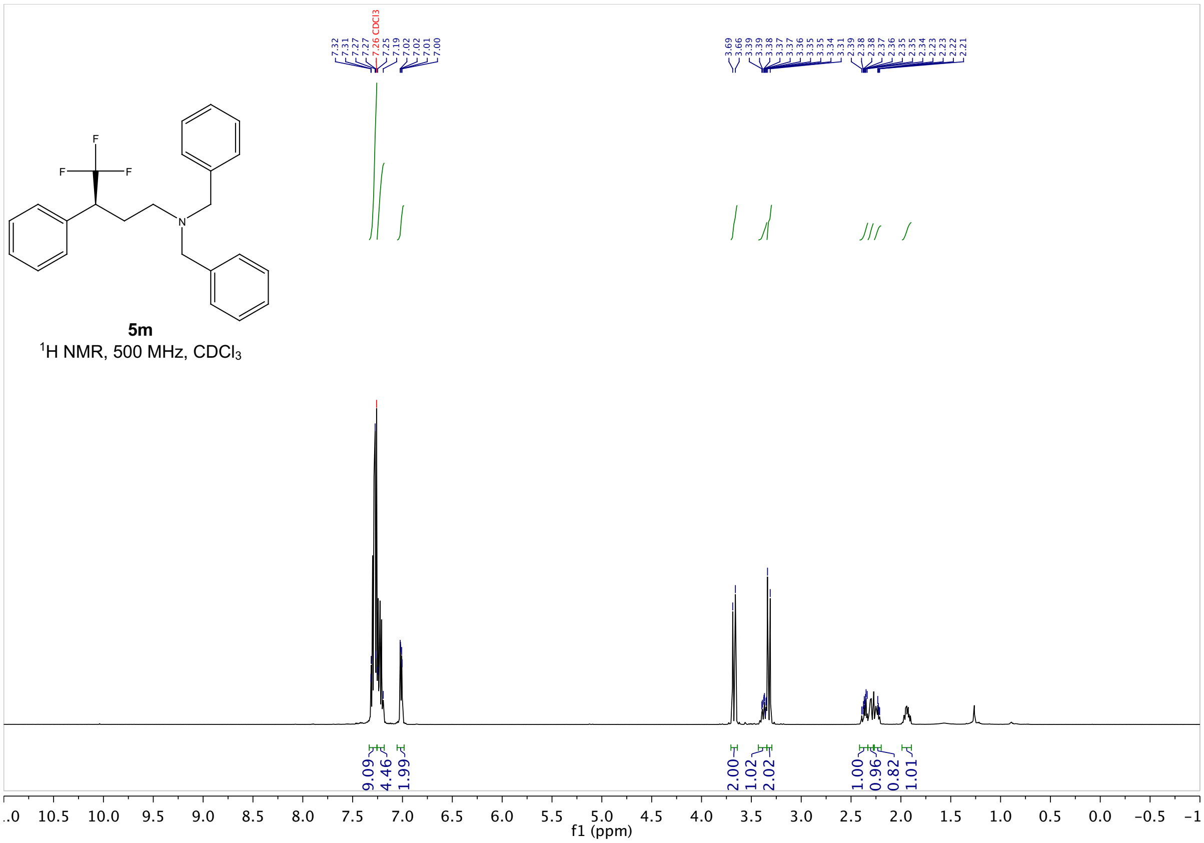


**Supplementary Figure 120.** $^{1}$H NMR spectra of compound **5m**

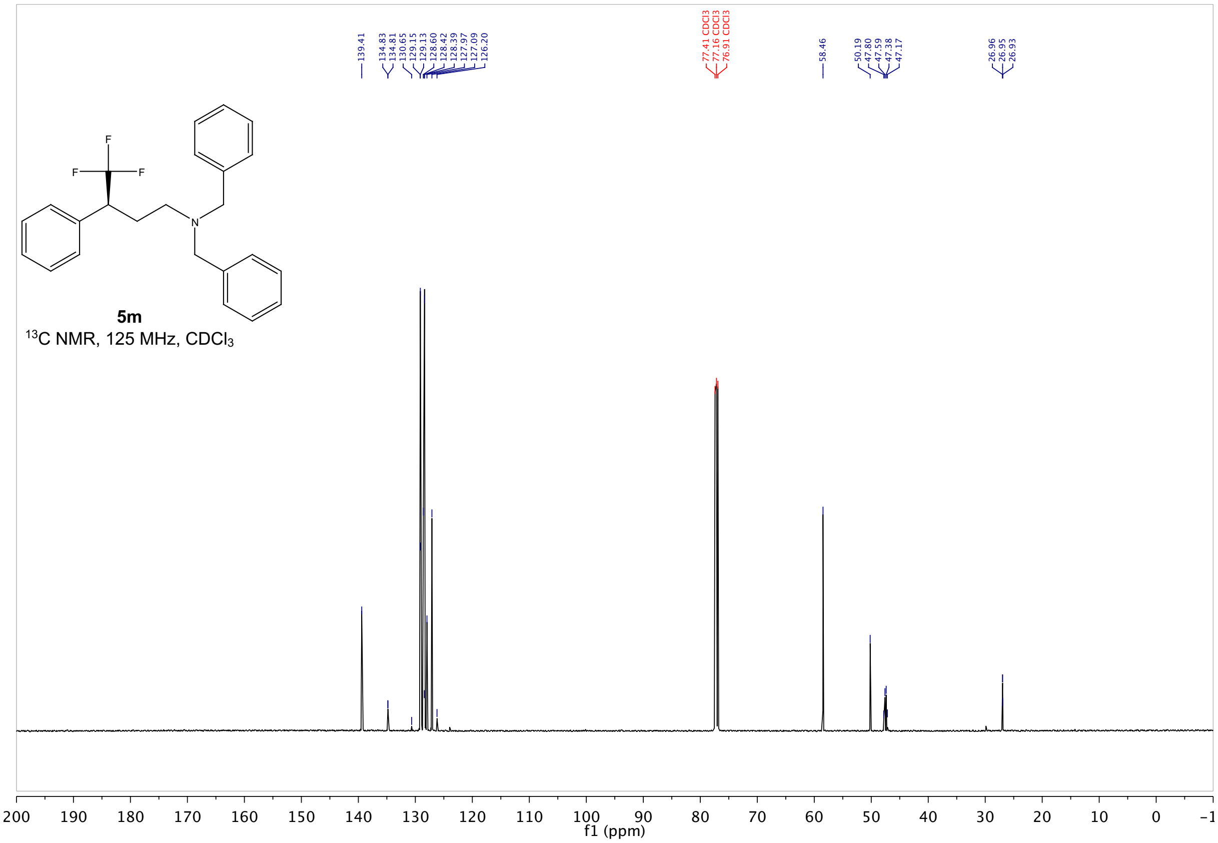


**Supplementary Figure 121.** $^{13}$C NMR spectra of compound **5m**

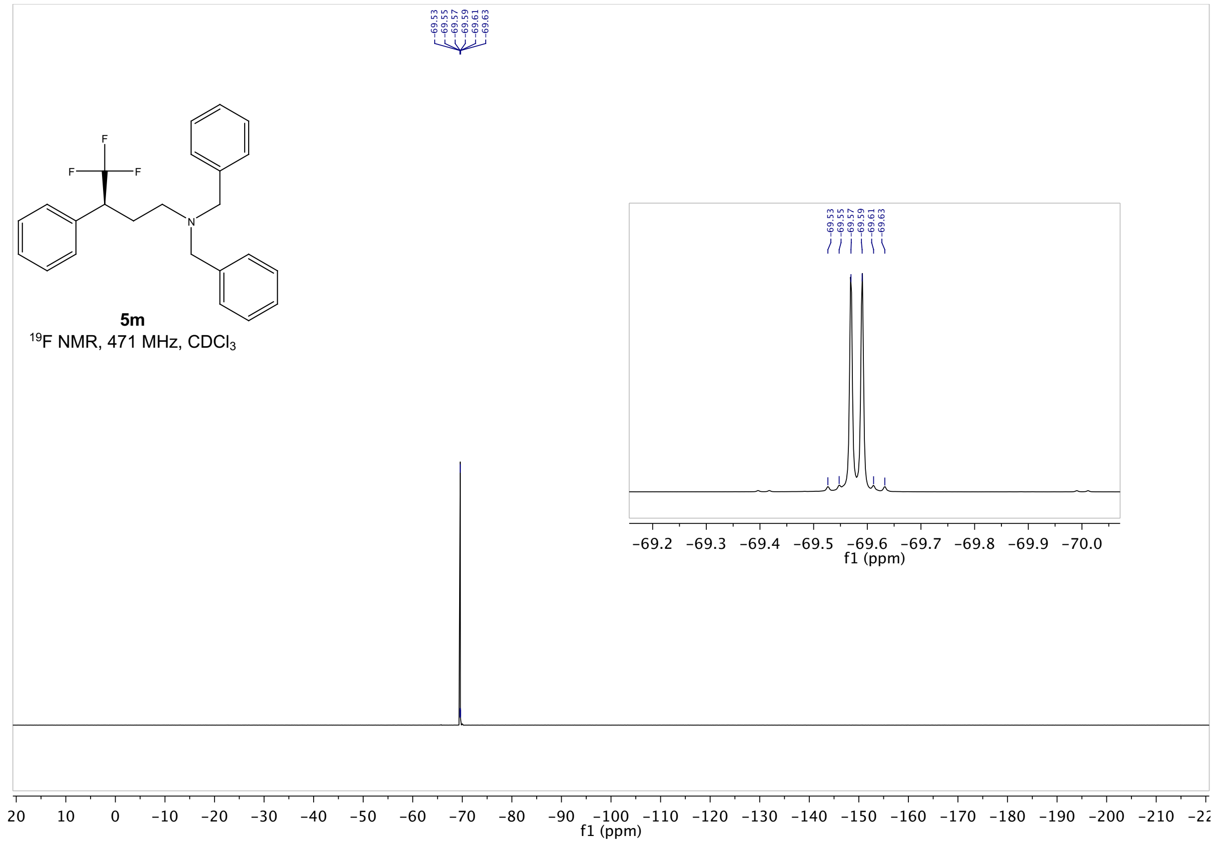


**Supplementary Figure 122.** $^{19}$F NMR spectra of compound **5m**

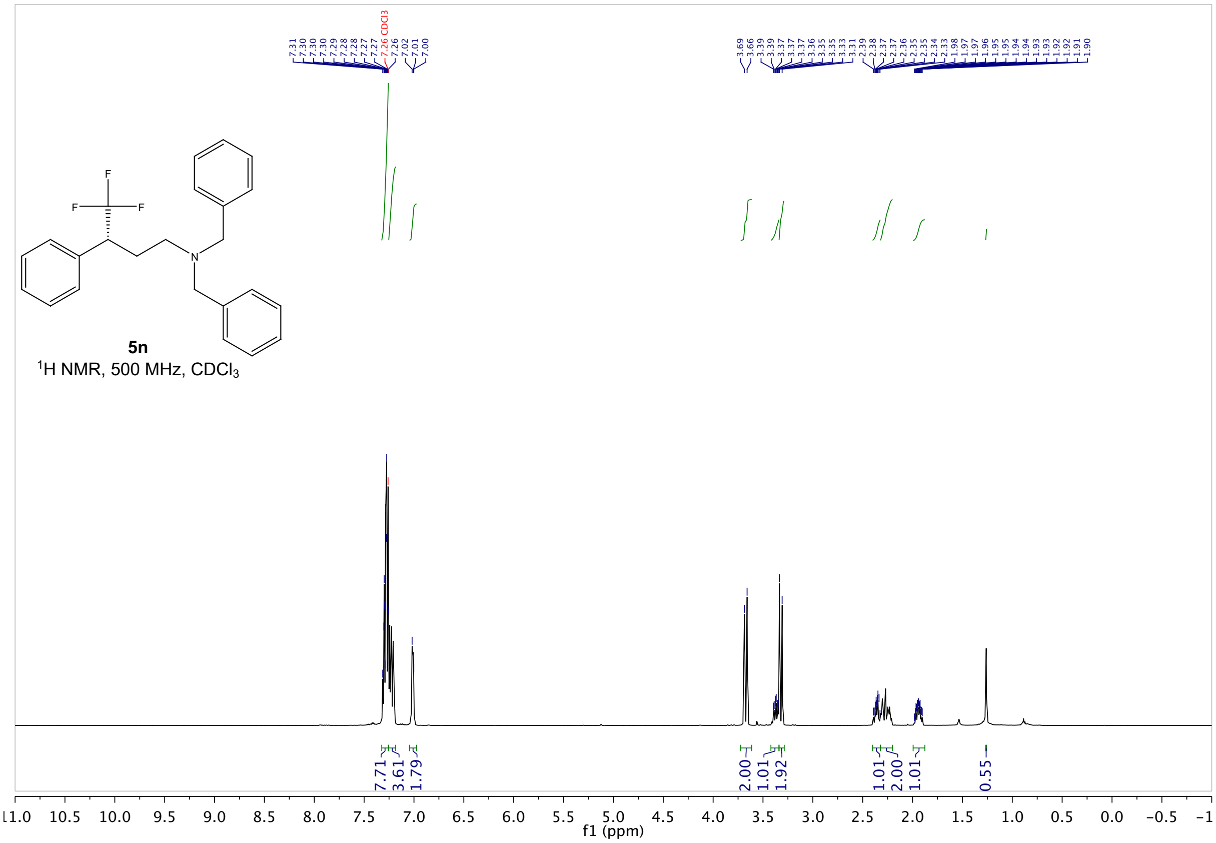

**Supplementary Figure 123.** $^{1}$H NMR spectra of compound **5n**

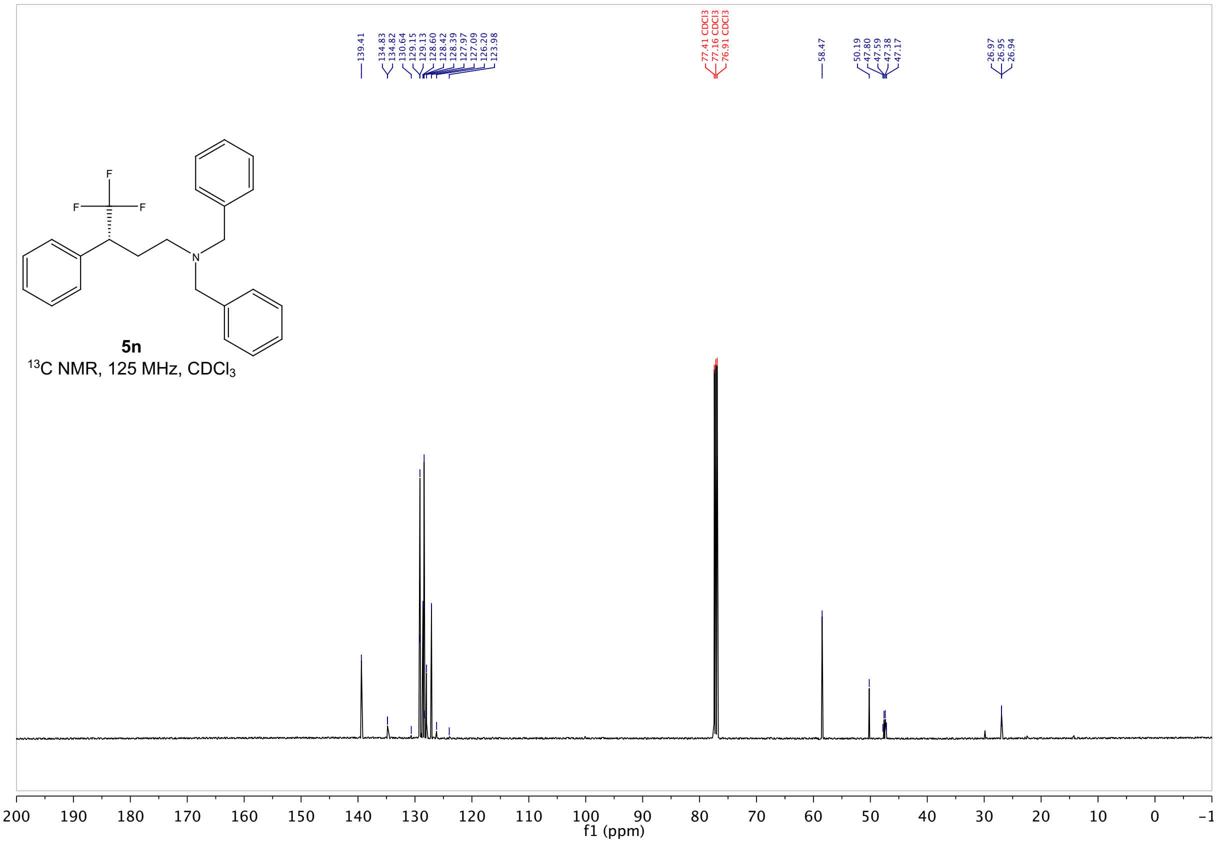

**Supplementary Figure 124.** $^{13}$C NMR spectra of compound **5n**

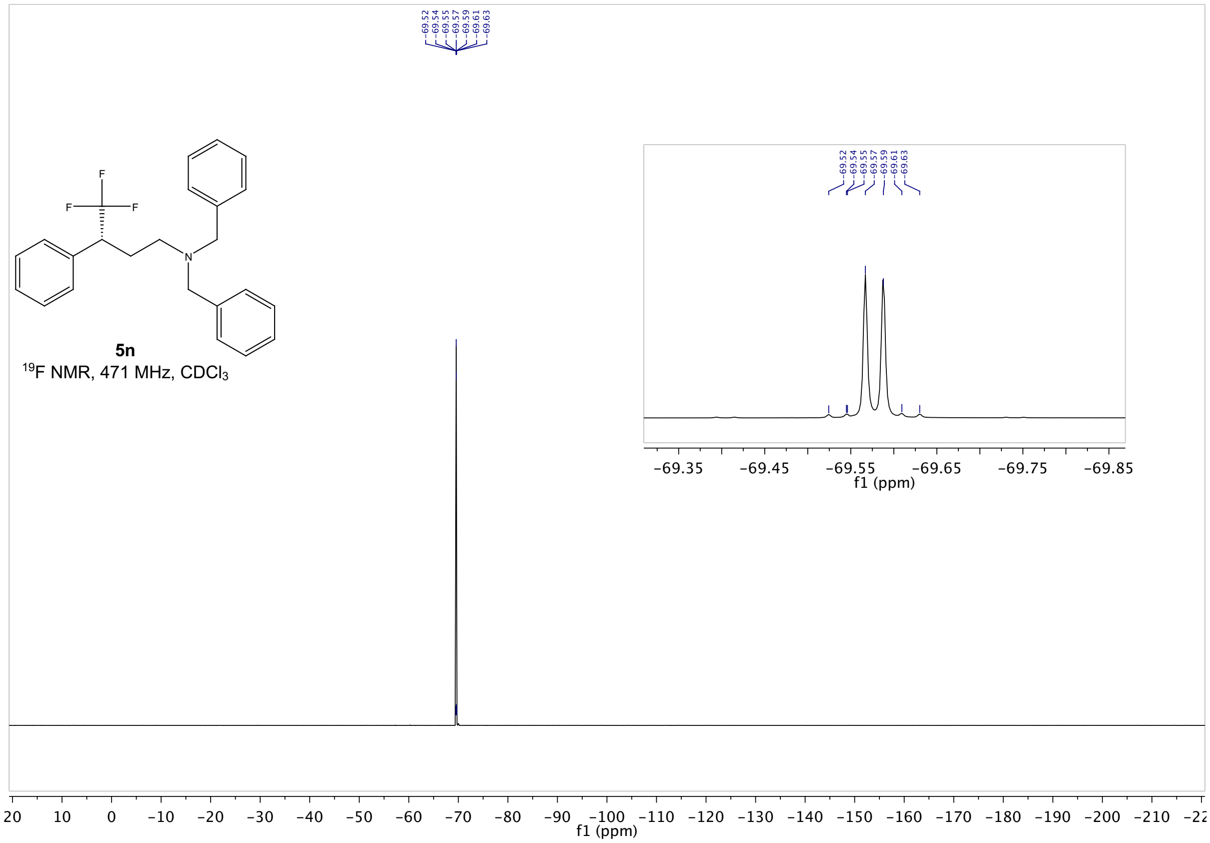

**Supplementary Figure 125.** $^{19}F$ NMR spectra of compound **5n**

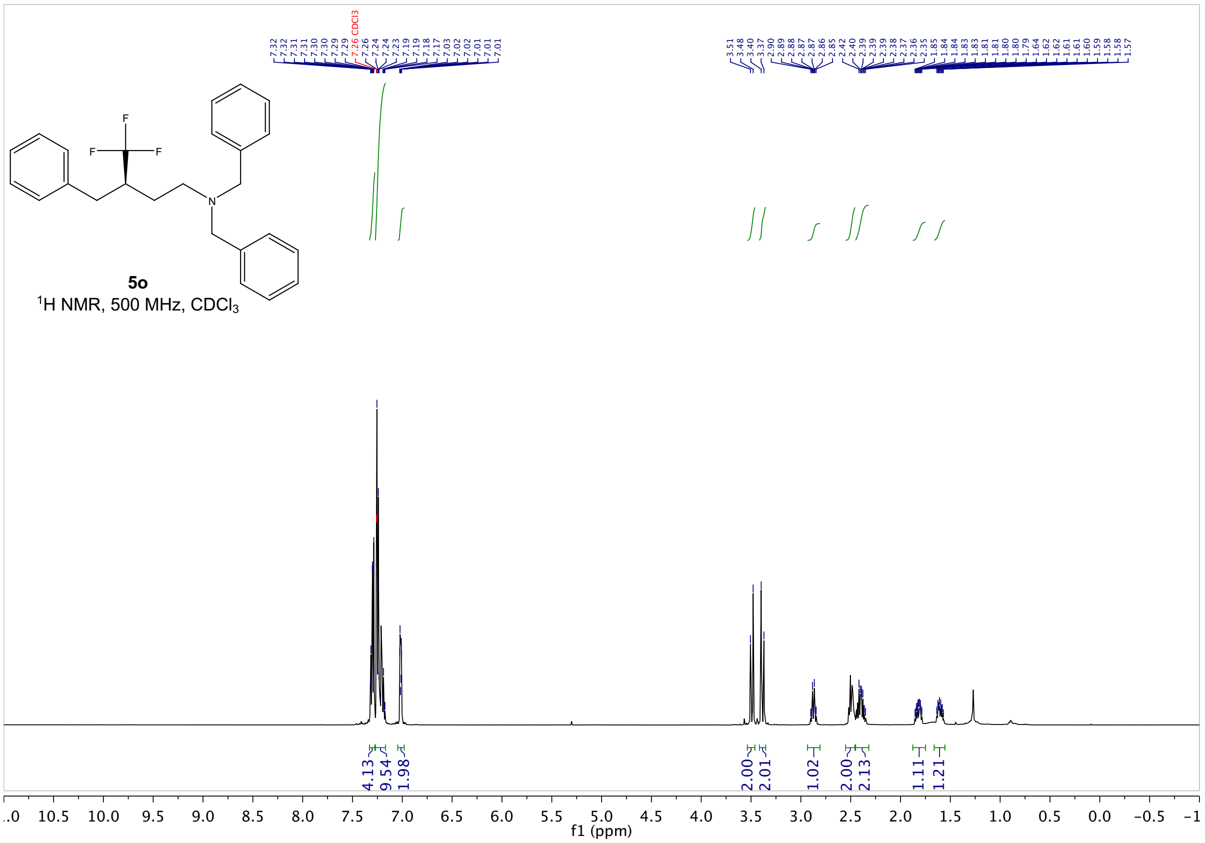

**Supplementary Figure 126.** $^{1}H$ NMR spectra of compound **5o**

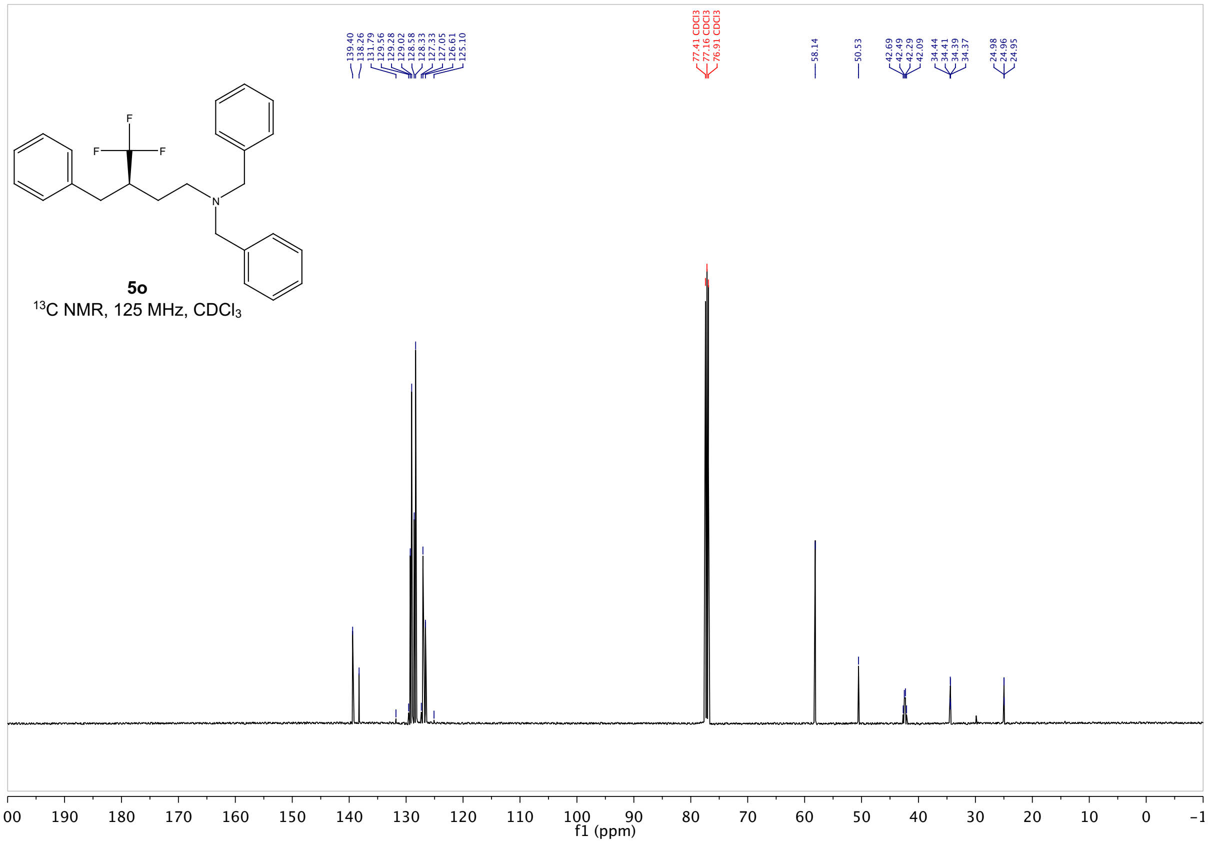


**Supplementary Figure 127.** $^{13}$C NMR spectra of compound **5o**

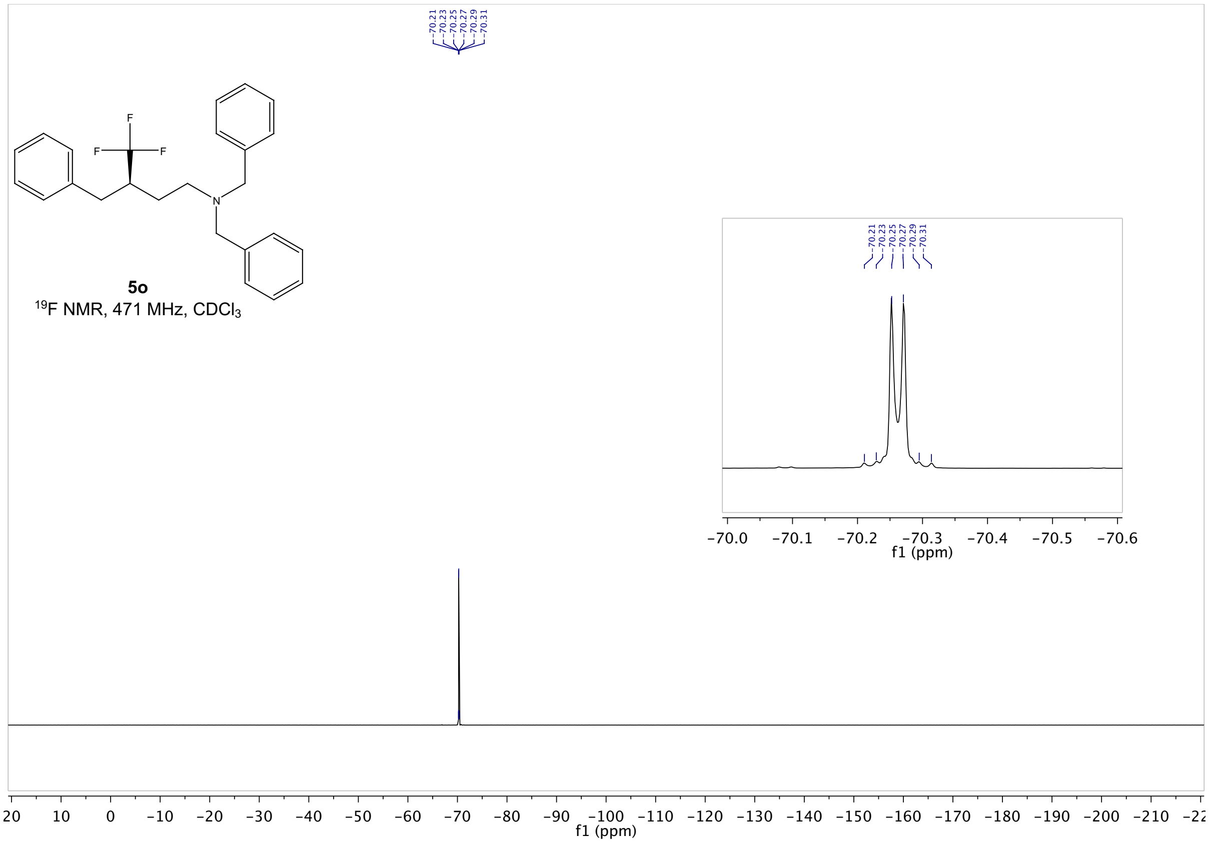


**Supplementary Figure 128.** $^{19}$F NMR spectra of compound **5o**

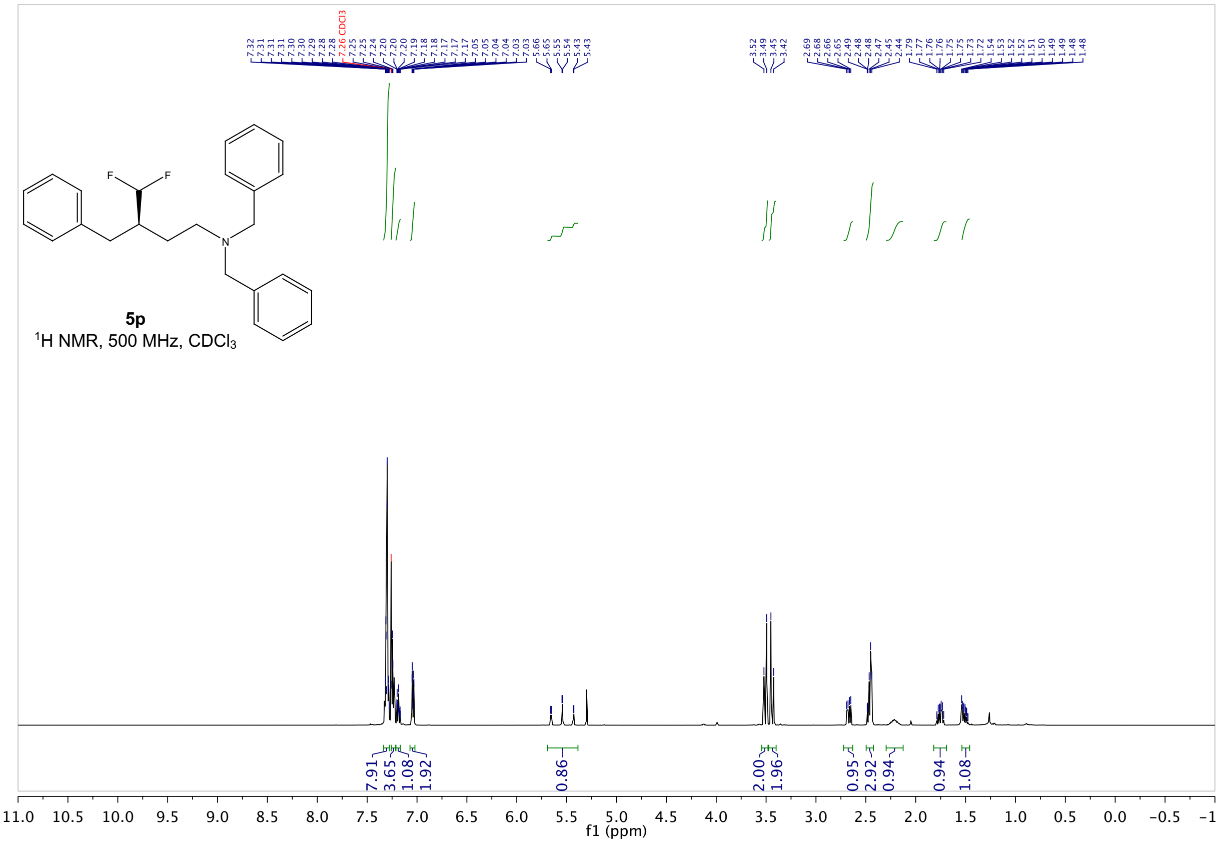


**Supplementary Figure 129.** $^{1}$H NMR spectra of compound **5p**

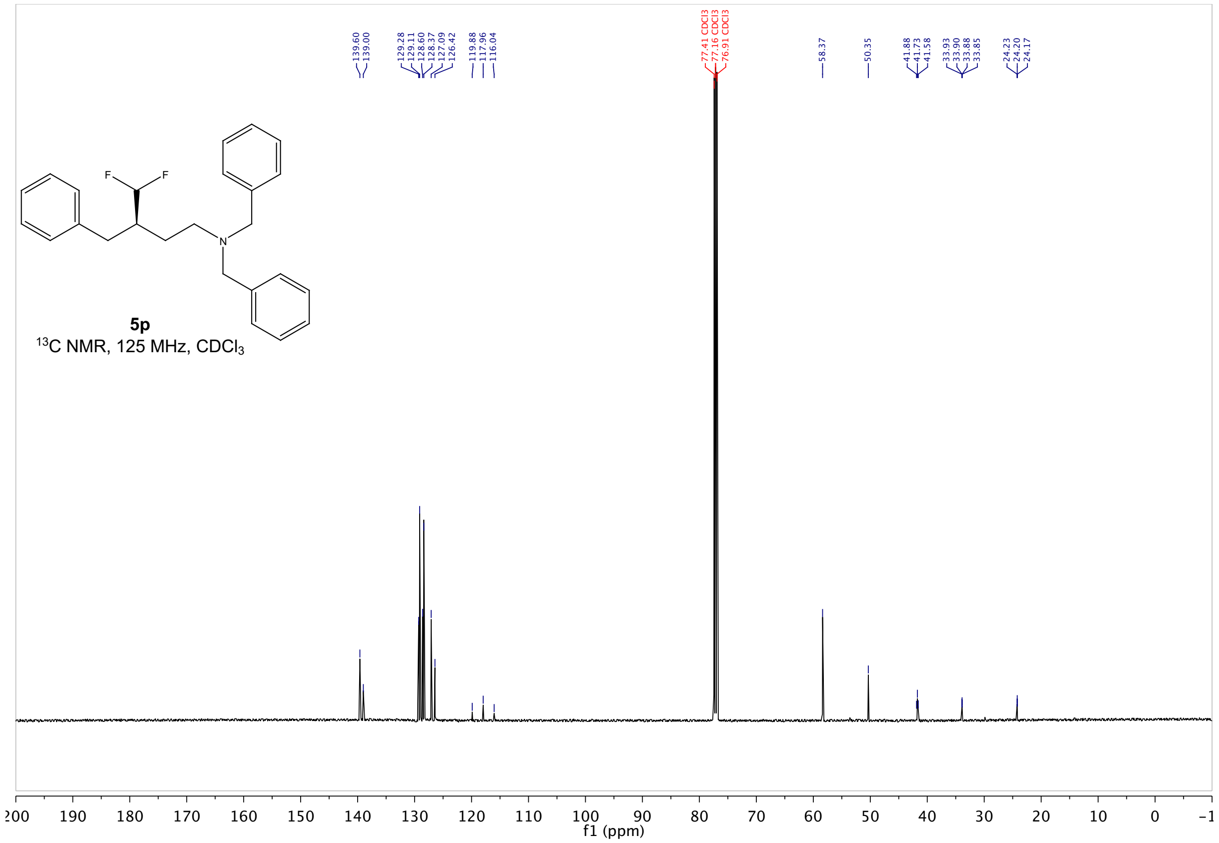


**Supplementary Figure 130.** $^{13}$C NMR spectra of compound **5p**

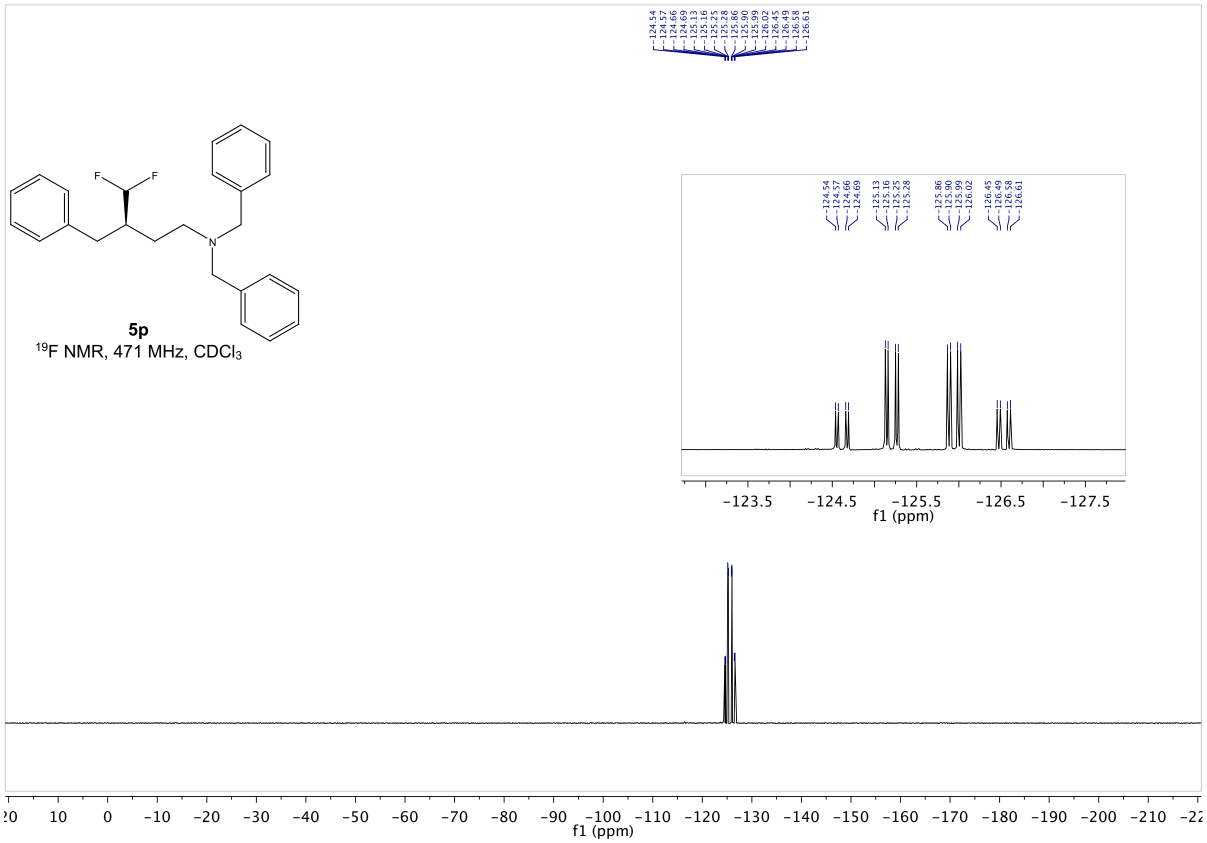


**Supplementary Figure 131.** $^{19}$F NMR spectra of compound **5p**

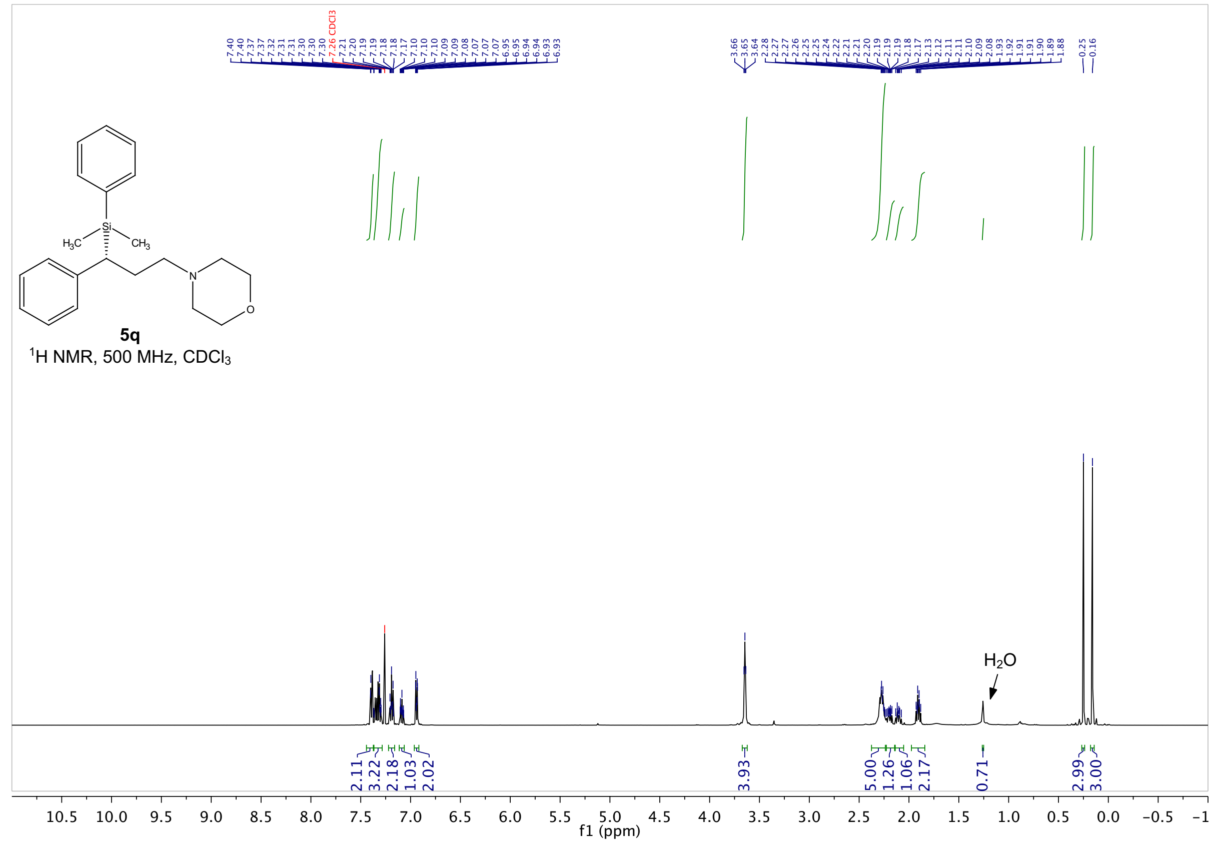


**Supplementary Figure 132.** $^1$H NMR spectra of compound **5q**

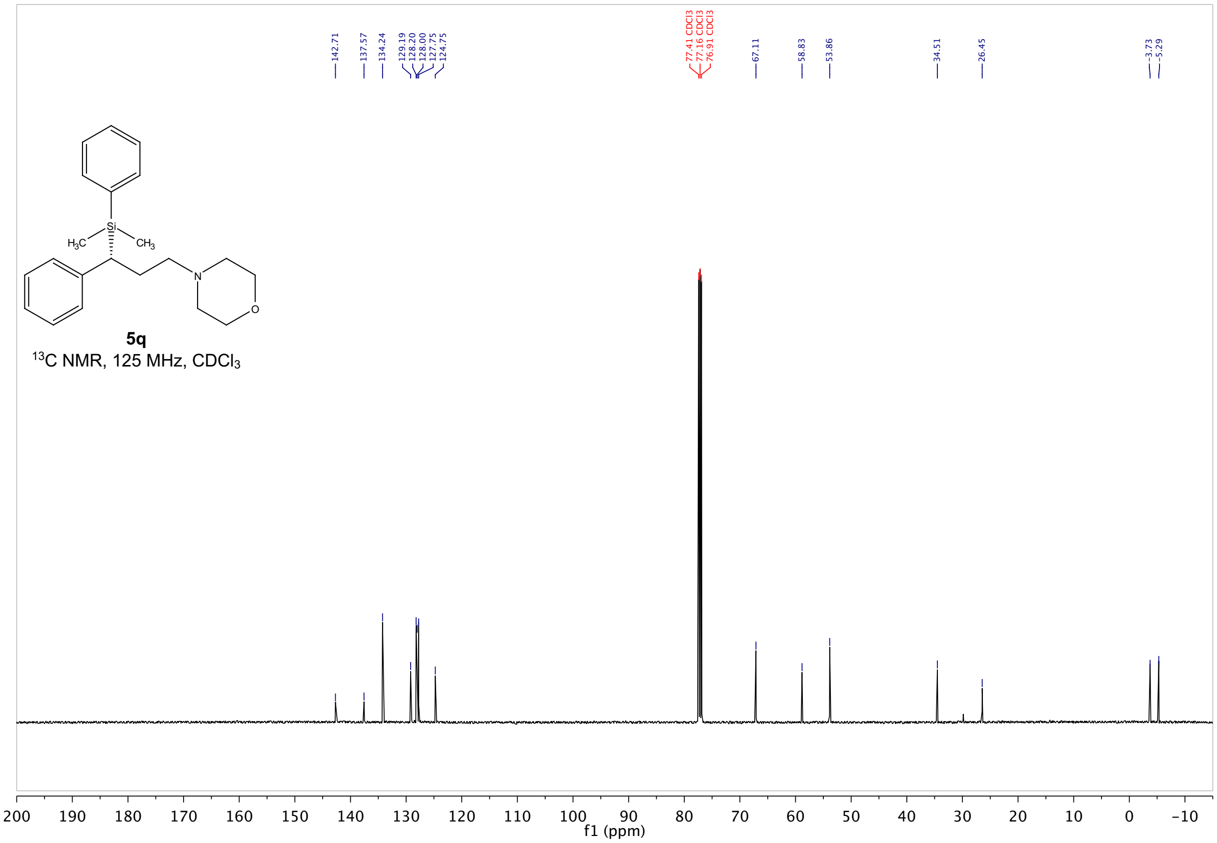


**Supplementary Figure 133.** $^{13}$C NMR spectra of compound **5q**

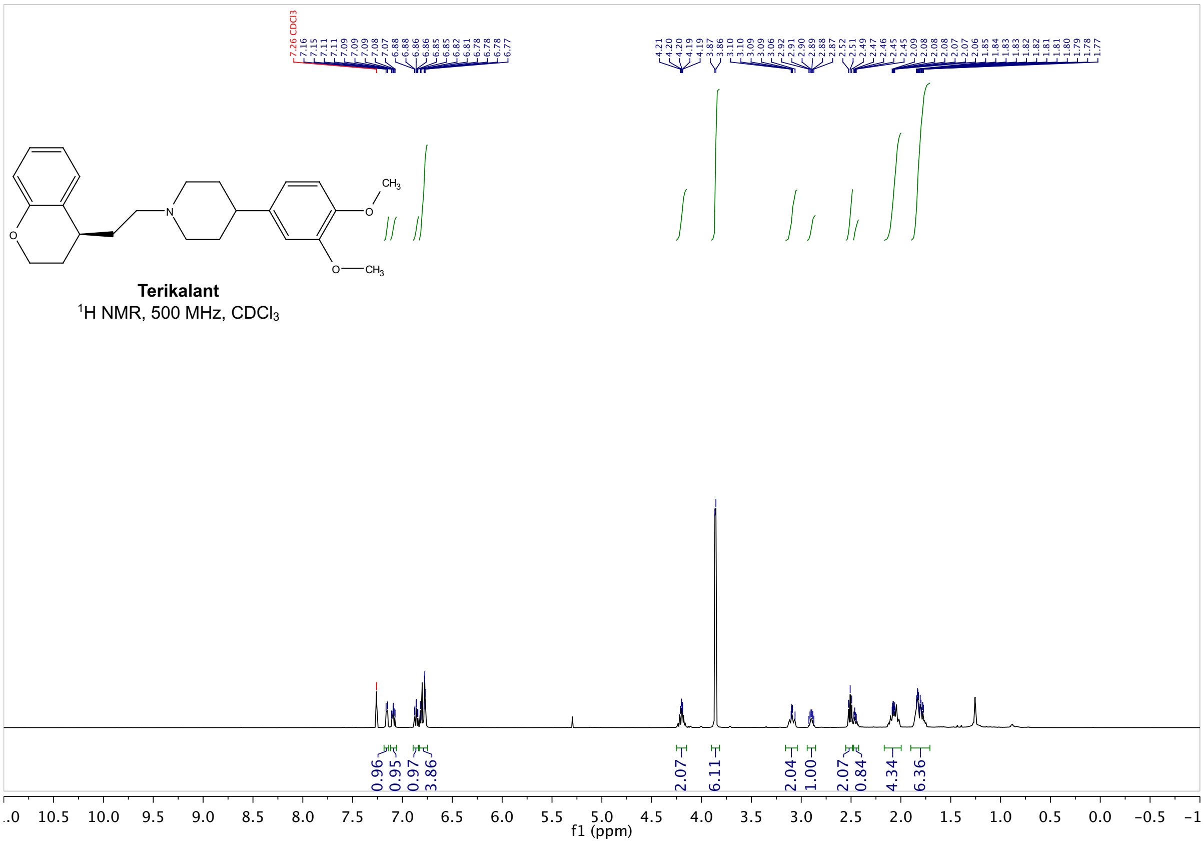

**Supplementary Figure 134.** $^{1}H$ NMR spectra of compound **Terikalant**

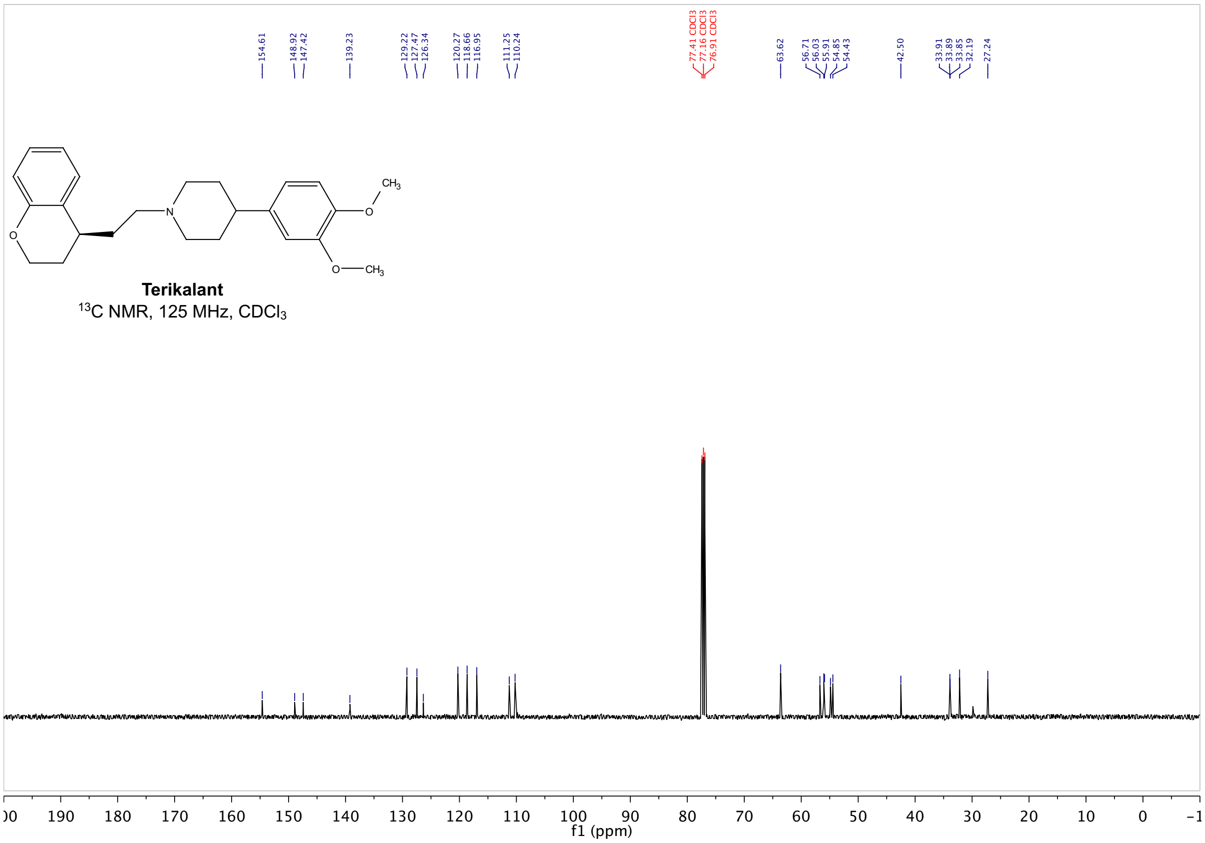

**Supplementary Figure 135.** $^{13}C$ NMR spectra of compound **Terikalant**

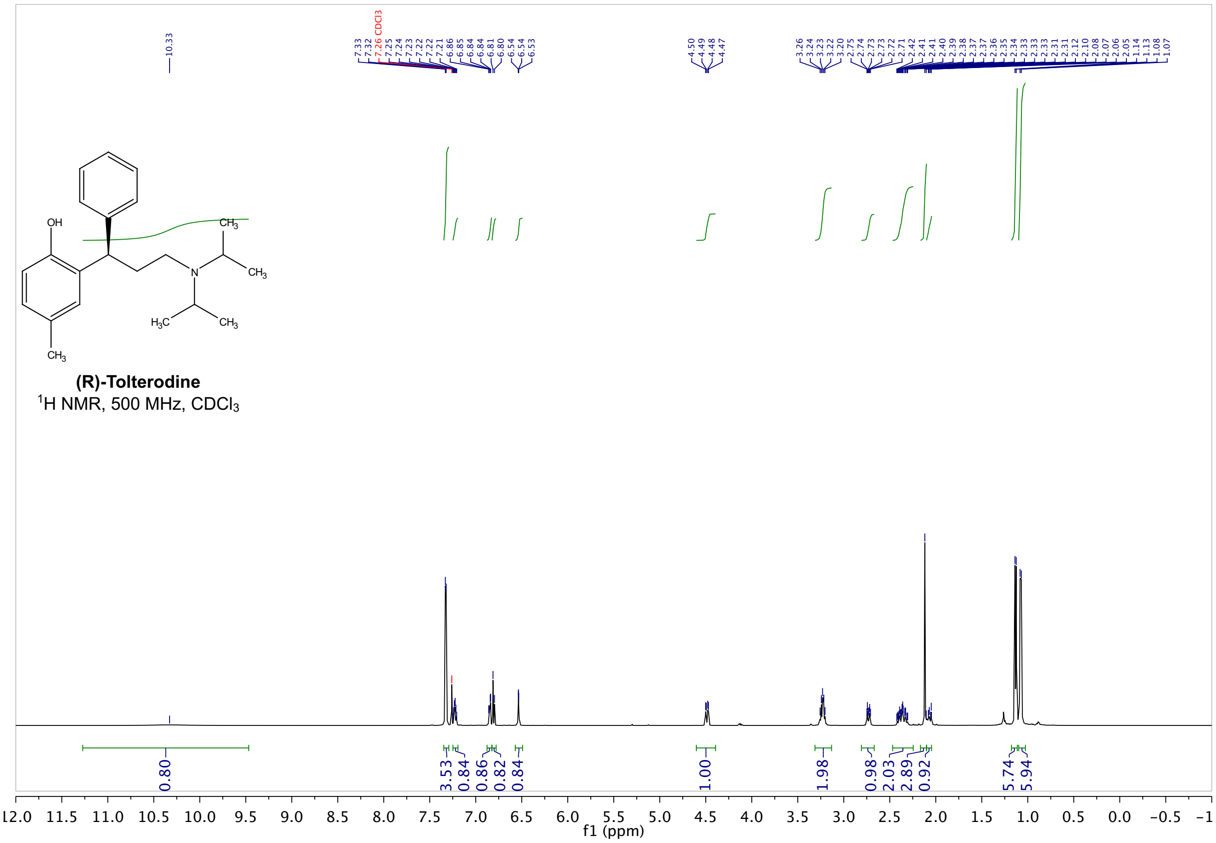


**Supplementary Figure 136.** $^{1}$H NMR spectra of compound **Tolterodine**

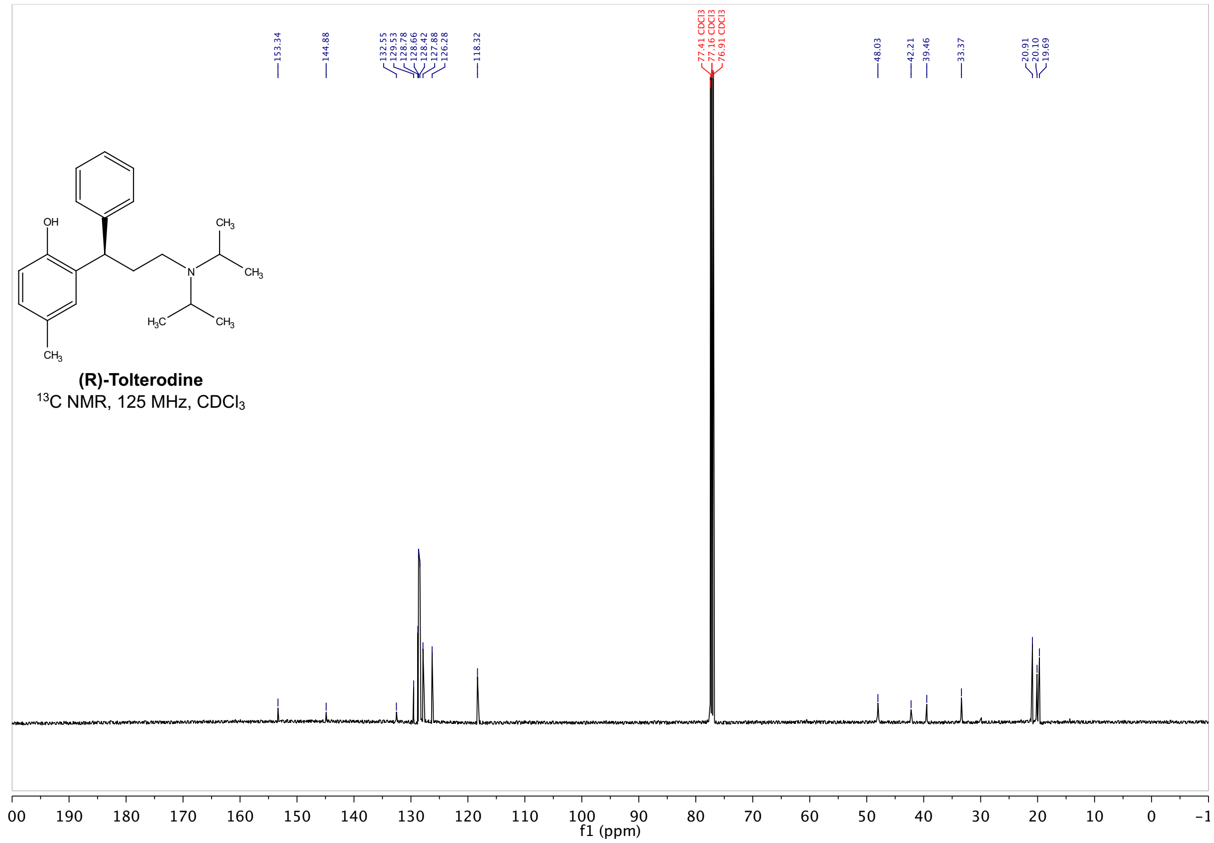


**Supplementary Figure 137.** $^{13}$C NMR spectra of compound **Tolterodine**

## Supplementary References


1. Wu, Z., Laffoon, S. D., Nguyen, T. T., McAlpin, J. D. & Hull, K. L. Rhodium-Catalyzed Asymmetric Synthesis of β-Branched Amides. *Angew. Chemie Int. Ed.* **56,** 1371–1375 (2017).

2. Limberger, J., Claudino, T. S. & Monteiro, A. L. Stereoselective synthesis of (*E*)-3,3-diaryl and (*E*)-3-aryl-3-aryloxy allylamines and allylalcohols from trans-cinnamyl chloride and alcohol. *RSC Adv.* **4,** 45558–45565 (2014).

3. Watanabe, M., Hisamatsu, S., Hotokezaka, H. & Furukawa, S. Reaction of lithiated senecioamide and related compounds with benzynes : Efficient syntheses of naphthols and naphthoquinones. *Chem. Pharm. Bull. (Tokyo).* **34,** 2810–2820 (1986).

4. Bizet, V., Pannecoucke, X., Renaud, J. L. & Cahard, D. Synthesis of β-$CF_3$ ketones from trifluoromethylated allylic alcohols by ruthenium catalyzed isomerization. *J. Fluor. Chem.* **152,** 56–61 (2013).

5. Eguchi, T., Aoyama, T. & Kakinuma, K. Remarkable Reversal of Stereoselectivity Olefinations of a-Fluorinated in Wittig-Type Alkyl Aryl Ketones. *Tetrahedron Lett.* **33,** 5545–5546 (1992).

6. Corey, E. J., Katzenellenbogen, J. A. & Posner, G. H. A new stereospecific synthesis of trisubstituted olefins. stereospecific synthesis of farnesol. *J. Am. Chem. Soc.* **89,** 4245–4247 (1967).

7. Ohkawa, S. et al. Benzo-fused 5-membered heterocyclic compounds, their production and use. *U. S. Pat. Appl. Publ.* 20070149558 (2007).
